# Rate and equilibrium constants for the addition of triazolium salt derived N-heterocyclic carbenes to heteroaromatic aldehydes†

**DOI:** 10.1039/d2sc05704b

**Published:** 2022-11-14

**Authors:** Zhuan Duan, Claire M. Young, Jiayun Zhu, Alexandra M. Z. Slawin, AnnMarie C. O'Donoghue, Andrew D. Smith

**Affiliations:** EaStCHEM, School of Chemistry, University of St Andrews North Haugh St Andrews Fife KY16 9ST UK ads10@st-andrews.ac.uk; Department of Chemistry, Durham University South Road Durham DH1 3LE UK annmarie.odonoghue@durham.ac.uk

## Abstract

Heteroaromatic aldehydes are often used preferentially or exclusively in a range of NHC-catalysed processes that proceed through the generation of a reactive diaminoenol or Breslow Intermediate (BI), with the reason for their unique reactivity currently underexplored. This manuscript reports measurement of rate and equilibrium constants for the reaction between *N*-aryl triazolium NHCs and heteroaromatic aldehydes, providing insight into the effect of the NHC and heteroaromatic aldehyde structure up to formation of the BI. Variation in NHC catalyst and heteroaromatic aldehyde structure markedly affect the observed kinetic parameters of adduct formation, decay to starting materials and onward reaction to BI. In particular, large effects are observed with both 3-halogen (Br, F) and 3-methyl substituted pyridine-2-carboxaldehyde derivatives which substantially favour formation of the tetrahedral intermediate relative to benzaldehyde derivatives. Key observations indicate that increased steric hindrance leads to a reduction in both *k*_2_ and *k*_−1_ for large (2,6-disubstituted)-*N*-Ar groups within the triazolium scaffold, and sterically demanding aldehyde substituents in the 3-position, but not in the 6-position of the pyridine-2-carboxaldehyde derivatives. As part of this study, the isolation and characterisation of twenty tetrahedral adducts formed upon addition of *N*-aryl triazolium derived NHCs into heteroaromatic aldehydes are described. These adducts are key intermediates in NHC-catalysed umpolung addition of heteroaromatic aldehydes and are BI precursors.

## Abstract 2

The combined effect of the heteroatom and an additional substituent both ortho to the aldehydic position in heteroaromatic aldehydes result in exceptionally large enhancements in equilibrium constants for tetrahedral adduct formation on NHC addition.

## Graphical abstract

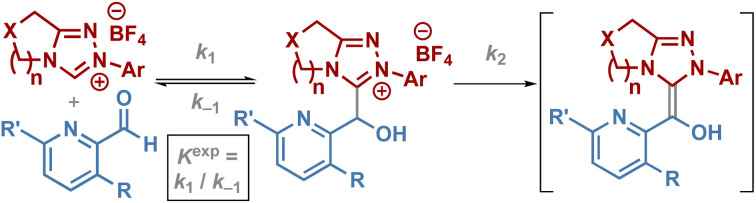

## Introduction

**Scheme 1:**
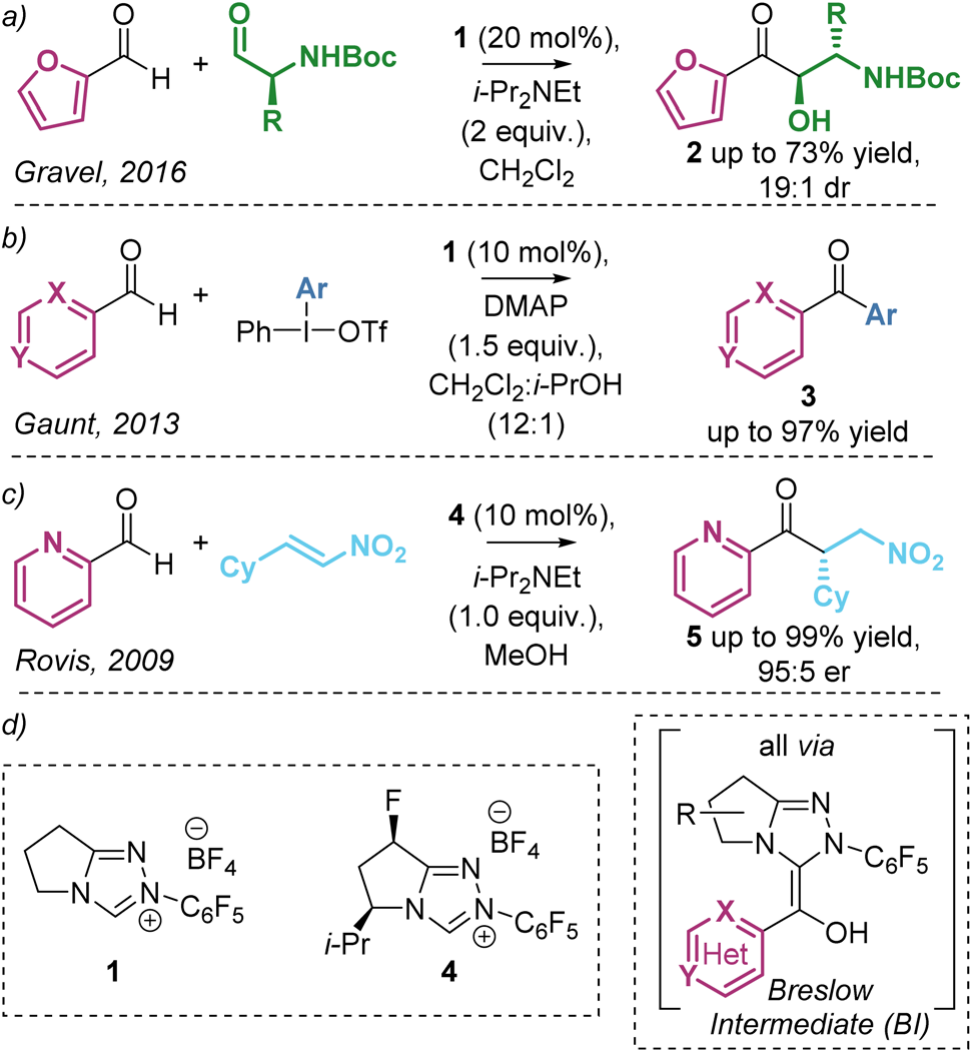
NHC-catalysed addition of heteroaromatic aldehydes to electrophiles *via* acyl anion equivalent Breslow intermediates.

The umpolung activation of aldehydes using N-heterocyclic carbene (NHC) catalysts is a widely applicable reaction mode and is generally regarded as a cornerstone of organocatalysis. These reactions harness the ability of NHCs to generate a key catalytic intermediate diaminoenol, commonly referred to as the Breslow Intermediate (BI, Scheme 1), in a remarkable range of synthetic processes.^1^ In recent reports, a significant trend has emerged in the preferred or sometimes exclusive use of heteroaromatic aldehydes as substrates, with furyl-, pyridyl- and quinolyl-derivatives commonplace despite reasons for their preferential reactivity remaining relatively unexplored.^2^ As well as providing unique reactivity, the incorporation of these heteroaromatic motifs in NHC-catalysed reactions is of widespread interest due to their importance in target bioactive compounds and natural products.^3^ As representative examples, interception of the BI (derived from heteroaromatic aldehydes and the NHC catalyst and acting as an acyl anion equivalent) with alternative electrophiles has been harnessed in NHC-catalysed transformations (Scheme 1). In a rare example of cross-benzoin reactions with heteroaromatic aldehydes, Gravel demonstrated the diastereoselective benzoin reaction of heteroaromatic aldehydes (predominantly using furfural) with enantiopure alkyl amino aldehydes (Scheme 1a).^4^ In related reports, the observed *anti*-diastereoselectivity was rationalised to be dependent upon the N–H substituent, with use of tertiary amines leading to *syn*-diastereoselectivity.^5^ In 2013 Gaunt reported the formation of ketones from a range of heteroaromatic aldehydes *via* arylation of the corresponding Breslow intermediate using iodonium salts to give a wide range of heteroaromatic ketones in good to excellent yields (Scheme 1b).^6^ Furthermore, in 2009, Rovis demonstrated the enantioselective intermolecular Stetter-type addition of heteroaromatic aldehydes (predominantly 2-pyridine carboxaldehyde) to nitroolefins giving the addition products in excellent yield and er (Scheme 1c).^7*a*^ Based upon invariance in reactivity and selectivity with electronic variation within the heteroarene, it was suggested that the pyridine nitrogen lone pair was not acting as a base in this transformation but may bias reactivity due to steric effects when compared with benzaldehydes.^7*b*^

**Scheme 2:**
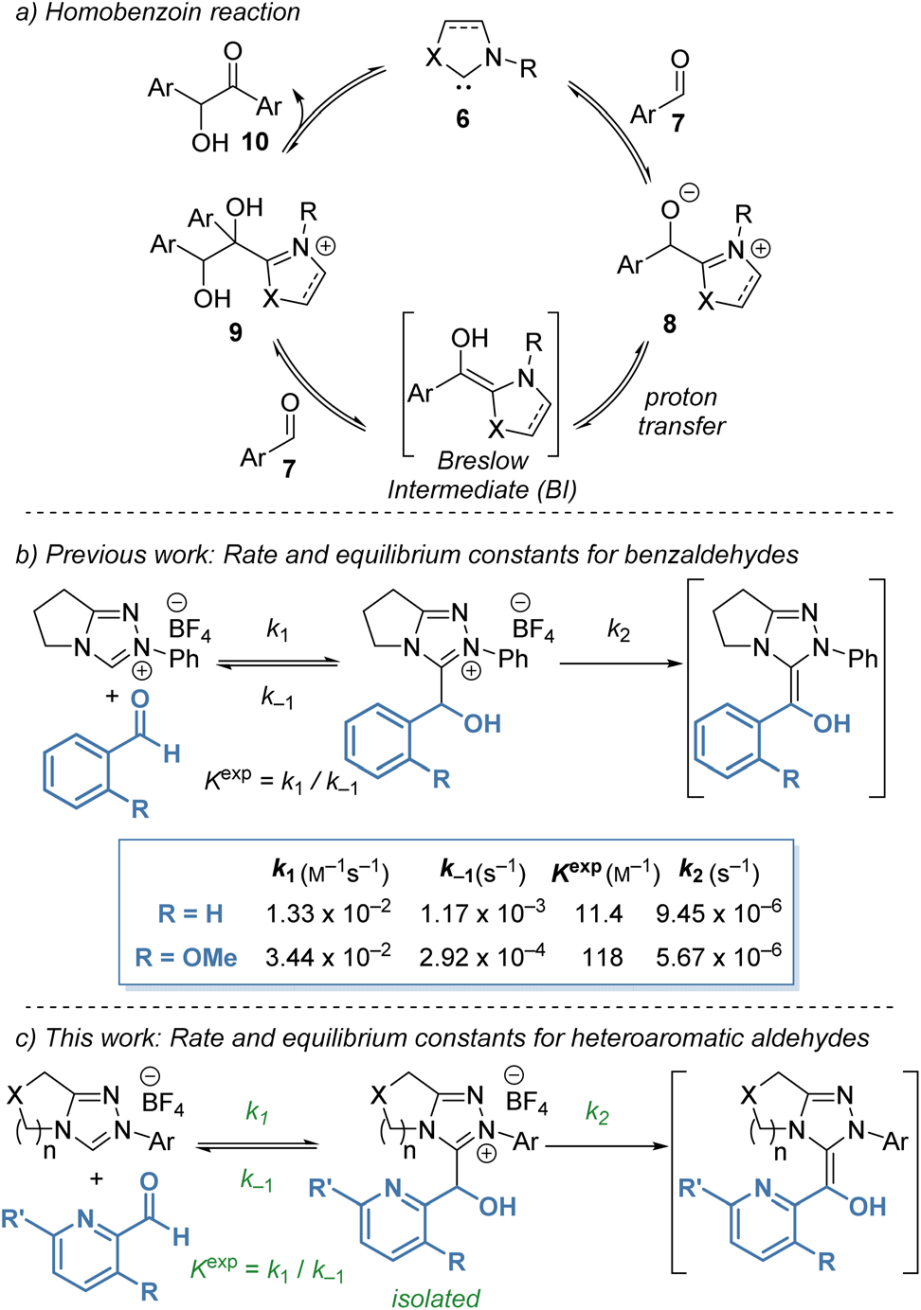
Mechanistic investigation of Breslow intermediate formation from *b*: substituted benzaldehydes and *c*: heteroaromatic aldehydes (this work). Starting concentrations for *b*: aldehyde (0.04 M), NHC precatalyst (0.04 M) in CD_3_OD and 0.18 M Et_3_N : Et_3_N·HCl (2 : 1) buffer at 25 °C. Starting concentrations for *c*: aldehyde (0.02 M), NHC precatalyst (0.02 M) in CD_3_OD and 0.09 M Et_3_N : Et_3_N·HCl (2 : 1) buffer at 25 °C.

Despite significant interest in the use of NHCs as organocatalysts, complete and rigorous mechanistic studies of processes proposed to involve the BI are still limited, with significant discussion of the role and character of the BI still ongoing.^8^ The original mechanism proposed by Breslow in 1958 (ref. 9) was based on Lapworth's mechanism for cyanide-catalysed benzoin formation^10^ and proceeds through a tetrahedral adduct of the NHC and aldehyde followed by proton transfer to form the reactive aminoenol (Scheme 2a). Reaction with aldehyde and elimination of the catalyst gives the homo-benzoin product. In recent studies, evidence of radical intermediates in the benzoin reaction has been shown computationally and experimentally, particularly in the presence of molecular oxidants, indicating that radical intermediates are feasible and should be considered.^11^ Under the polar protic conditions explored in this manuscript, an ionic proton-transfer mechanism is expected to dominate due to the rapidity of proton exchange under the methanolic reaction conditions. Furthermore, no radical derived side products have been observed under our reaction conditions.

In previous work, our groups investigated the rate and equilibrium constants for the reaction of NHCs derived from triazolium salts with benzaldehydes in triethylamine buffered methanol (CD_3_OD and 0.18 M Et_3_N : Et_3_N·HCl (2 : 1) buffer at 25 °C).^12^ Benzaldehydes bearing heteroatomic 2-substituents exhibited significantly higher *K*^exp^ than the parent benzaldehyde (Scheme 2b). For example, 2-methoxybenzaldehyde (R = OMe) gave a ten-fold increase in *K*^exp^ when compared with benzaldehyde. Further kinetic investigation of the reaction of various aldehydes with triazolium salts revealed that the increase in *K*^exp^ for 2-substituted aldehydes was a compound result of an increase in *k*_1_ and a reduction in *k*_−1_ for formation and decay of tetrahedral hydroxyaryl adduct, respectively. Despite the high *K*^exp^, 2-substituted aldehydes do not react selectively as nucleophilic partners in cross benzoin reactions. Instead, they are highly selective electrophilic partners, with this selectivity ascribed to onwards reactivity post BI formation. Also identified in this study, was an increased *K*^exp^ for 2-pyridyl carboxaldehyde in CH_2_Cl_2_ in comparison with benzaldehyde.

Intrigued by these observations, and the use of heteroaromatic aldehydes in NHC organocatalysis, we sought to investigate the kinetics of the reaction of heteroaromatic aldehydes with NHCs to determine if trends in reactivity could be identified. In this manuscript, the synthesis and isolation of a large range of tetrahedral adducts (20) formed from NHCs derived from triazolium salts and heteroaromatic aldehydes are reported (Scheme 2c).^13^ Kinetic analysis of tetrahedral adduct formation and decay was performed, and the effect of both catalyst structure (*N*-aryl substitution, variation of the fused ring-size (*n*) and incorporation of an oxygen atom within the fused ring) as well as heteroaromatic aldehyde structure on these rates is reported. Given the enormous interest in incorporating heteroaromatic groups into pharmaceutical and agrochemical compounds, understanding the reactivity of these heteroaromatic aldehydes in NHC-catalysed processes will be of significant interest.

## Results and discussion

### Synthesis and characterisation of tetrahedral adducts

**Scheme 3:**
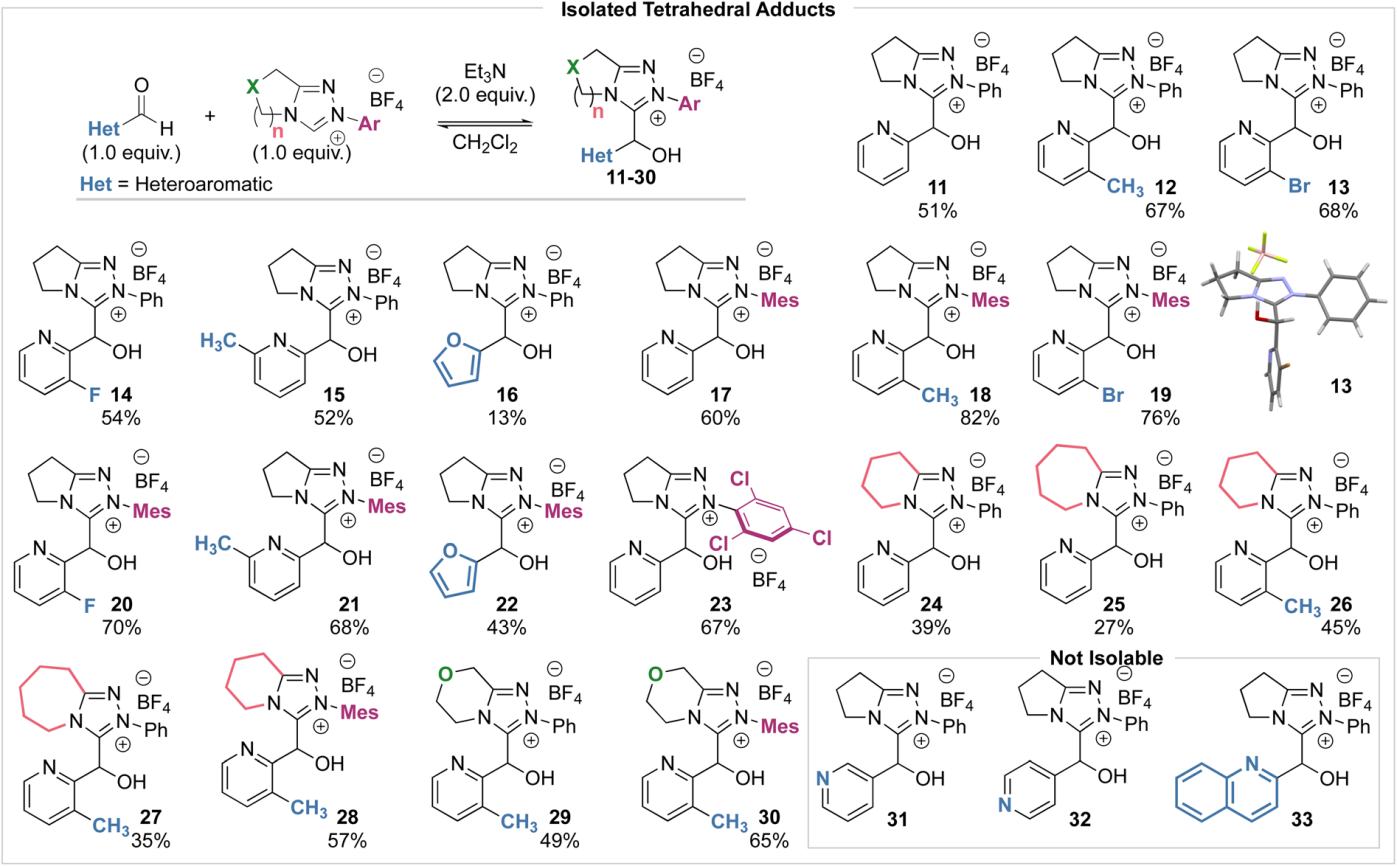
Preparation and characterization of tetrahedral intermediates.

To compare the rate and equilibrium constants for NHC addition to heteroaromatic aldehydes, characterisation of the corresponding tetrahedral adducts to allow unambiguous confirmation of their constitution was required (Scheme 3). Studies therefore began with the isolation of tetrahedral adducts through stoichiometric reaction of the NHCs derived from triazolium salts and heteroaromatic aldehydes in CH_2_Cl_2_ at room temperature. The reactions were allowed to reach equilibrium before direct purification by flash column chromatography to give the tetrahedral adducts in acceptable to good yields. A notable exception to this generalisation was observed when triazolium salts with 6- and 7-membered (*n* = 2, 3) auxiliary rings were used, with isolated yields generally lower, ranging from 27% to 45%. A selection of heteroaromatic aldehydes and triazolium salts were employed to facilitate the investigation of various steric and electronic aspects of each reaction component. For the heteroaromatic aldehydes, pyridine-2-carboxaldehyde derivatives with substituents in the 3-position (*ortho* to the aldehyde, R) or 6-position (*ortho* to the pyridine nitrogen, R′) as well as furfural were employed. For the triazolium salts, structural changes included variation of the *N*-aryl substituent on the triazolium nitrogen (Ar), variation of fused ring-size (*n*) and incorporation of an oxygen atom within the fused ring (X). Unfortunately, tetrahedral intermediates arising from the use of *N*-C_6_F_5_ substituted azolium salt 1 were not stable to purification and could not be isolated, although *N*-Ph, *N*-Mes and *N*-C_6_Cl_3_H_2_ substituted variants proved isolable.^14^ X-ray crystallographic analysis of 13 allowed unambiguous confirmation of product constitution.^15^ Due to their propensity to onward reactions under these conditions, 3- and 4-pyridyl, and quinolyl aldehydes led to complex mixtures from which the tetrahedral adducts 31, 32 and 33 could not be isolated but were identified spectroscopically in the crude reaction mixtures.^16^

### Kinetic analysis

Under reaction conditions first reported by Leeper and since adapted by us and others to give consistent and comparable experimental results, the reaction of the NHCs with heteroaromatic aldehydes were monitored by ^1^H NMR in buffered methanol (triazolium salt 20 mM, aldehyde 20 mM, Et_3_N : Et_3_N·HCl (2 : 1) 90 mM in CD_3_OD at 25 °C).^17^ The pseudo second order rate constant of tetrahedral adduct formation was measured (*k*_1_) as well as the equilibrium constant for adduct formation (*K*^exp^) (Table 1). The pseudo-first order rate constant for tetrahedral adduct decay (*k*_−1_) was calculated based on the measured values for *K*^exp^ and *k*_1_. The pseudo-first order reaction constant for forward reaction of the tetrahedral adduct (*k*_2_) was also estimated from the rate of decay of the tetrahedral intermediate once equilibrium for the intermediate formation had been established.^16^ It is assumed that this forward reaction proceeds *via* the corresponding BI. Although this intermediate cannot be observed directly under these polar protic conditions, the observation of H/D-exchange at C_α_ implicates a carbanionic intermediate. Furthermore, the increases in *k*_2_ observed in studies to date with electron withdrawing substituents on catalysts or aldehyde supports this proton transfer mechanism *via* a delocalised carbanion (*cf.* the diaminoenol or BI).

**Table 1:** Rate and equilibrium constants of NHC addition to pyridyl aldehydes 34–38 and Breslow intermediate formation^a^

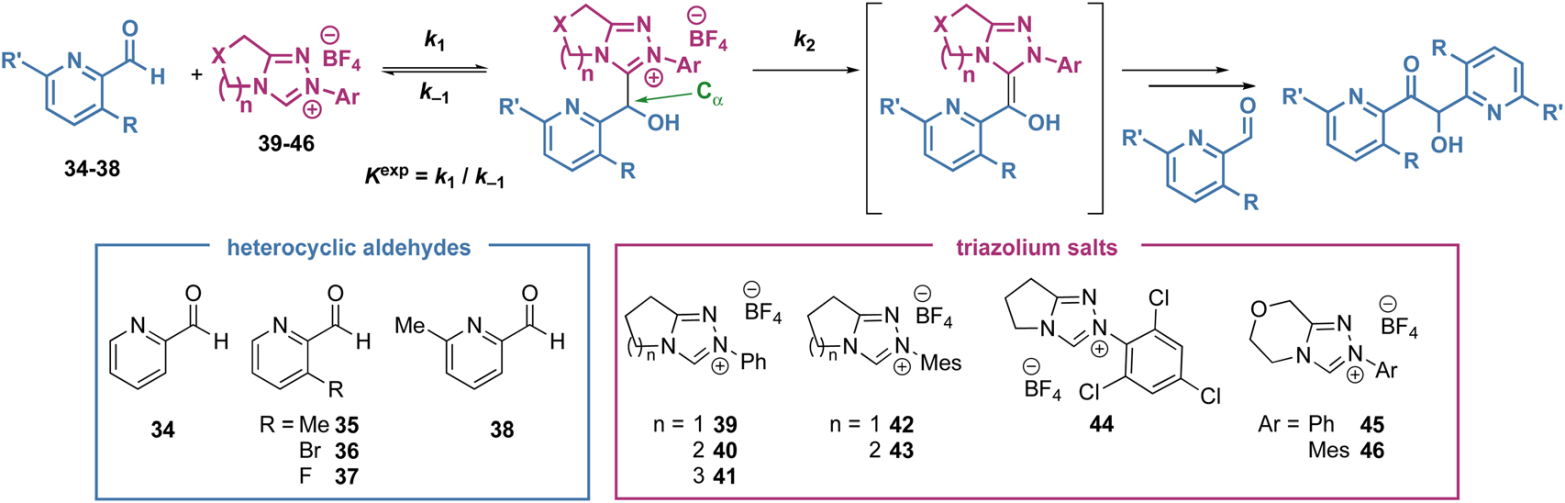										
Entry	Tetrahedral adduct	Aldehyde	Triazolium salt	*k*_1_ (×10^−2^ M^−1^ s^−1^)	*k*_1_^fit^ (×10^−2^ M^−1^ s^−1^)	*k*_−1_ (×10^−5^ s^−1^)	*k*_−1_^fit^ (×10^−5^ s^−1^)	*K* ^exp^ (×10^2^ M^−1^)	*K* ^fit^ (×10^2^ M^−1^)	*k*_2_ (×10^−6^ s^−1^)
1	11	34	39	—b	58.8	—b	29.4	—b	20.0	115
2	12	35	39	19.3	19.1	4.38	4.51	43.6	42.4	10.8
3	13	36	39	366	366	9.09	9.11	403	402	87.9
4	14	37	39	—b	253	—b	26.9	—b	94.1	344
5	15	38	39	—b	42.6	—b	21.6	—b	19.7	81.4
6	17	34	42	—b	77.7	—b	3.19	—b	244	105
7	18	35	42	35.0	33.9	0.725	0.710	482	477	5.65
8	19	36	42	491	424	1.30	1.11	3770	3810	9.00
9	20	37	42	—b	294	—b	3.28	—b	896	254
10	21	38	42	—b	79.1	—b	3.50	—b	226	64.3
11	23	34	44	—c	—c	—c	—c	481	—c	6190
12	47	35	44	—c	—c	—c	—c	1040	—c	1040
13	24	34	40	—b	5.06	—b	8.53	—b	5.93	3.94
14	25	34	41	—b	4.30	—b	15.4	—b	2.79	—d
15	26	35	40	1.86	1.92	4.11	4.12	4.53	4.66	2.28
16	27	35	41	1.27	1.21	7.99	7.37	1.59	1.65	—d
17	28	35	43	8.90	8.19	1.13	1.04	78.7	78.8	3.61
18	29	35	45	24.3	23.5	27.9	27.3	8.61	8.72	14.5
19	30	35	46	115	117	7.37	7.53	156	155	18.6

a Conditions: triazolium salt 20 mM, aldehyde 20 mM, Et_3_N : Et_3_N·HCl (2 : 1) 90 mM in CD_3_OD. *k*_1_, *k*_−1_, *K*^exp^ and *k*_2_ obtained from ^1^H NMR analysis, k^fit^_1_, k−1fit and *K*^fit^ obtained using Berkeley Madonna fitting software based on experimental data. See ESI for details.

b Competitive benzoin reaction prevented the experimental reaction from reaching equilibrium over the reaction course and so experimental data could not be calculated. The rate and equilibrium constants were extracted from experimental data through global fitting using Berkeley Madonna.

c The reaction proceeded rapidly and individual rate constants could not be calculated or extracted from global fitting using Berkeley Madonna.

d *k*_2_ could not be measured as no decrease in adduct concentration was observed.

Equilibrium constants for the decay of selected isolated adducts to their respective aldehydes and NHCs (*K*^diss^) under identical reaction conditions were also measured. While performing the experiments, competitive generation of benzoin product in some cases limited the data that could be measured experimentally. For example, with furfural no useable data could be extracted despite 16 and 22 being isolable and so kinetic analysis was not feasible. With pyridyl aldehyde 34, the limited data measured by experiments could be fitted using Berkeley Madonna global fitting software to access the rate constants. This fitting using Berkeley Madonna was also performed for reactions where the experimental data could be used directly, with the parameters obtained from fitting in good agreement with the experimental data in all cases.

An initial observation comparing pyridine-2-carboxaldehyde with benzaldehyde (under slightly modified conditions, Scheme 2), revealed a significant increase in *forward* rate constants for the pyridyl aldehyde (*k*_1_ = 5.88 × 10^−1^ M^−1^ s^−1^, *k*_2_ = 1.15 × 10^−4^ s^−1^) and decrease in *k*_−1_ (2.94 × 10^−4^ s^−1^) reflected in an almost 200-fold increase in *K*^exp^ (2.00 × 10^3^ M^−1^) (Table 1, entry 1 and Scheme 2b). Further trends in the reaction of pyridyl aldehydes with triazolium salts were observed and are discussed below.

### Tetrahedral adduct formation (*k*_1_, *k*_−1_, *K*^exp^)

#### Effect of 3-substituent on aldehyde (R) (Table 1, entries 1–4 and 6–9)

In the context of the previously observed enhanced reactivity of 2-substituted aldehydes, we proposed 3-substituted pyridine-2-carboxaldehyde derivatives to investigate the effect of additional substitution *ortho* to the aldehyde functional group in combination with the presence of a ring nitrogen *ortho* to the aldehyde. In reactions with triazolium 39, the rate and equilibrium constants can be compared when the substituent in the 3-position is varied (34 (R = H), 35 (R = Me), 36 (R = Br), 37 (R = F), Table 1, entries 1–4). Equilibrium constants *K*^exp^ are up to 20-fold larger for non-hydrogen *ortho* substituted aldehydes (R = H: 2.00 × 10^3^ M^−1^; Me: 4.36 × 10^3^ M^−1^; Br: 4.03 × 10^4^ M^−1^; F: 9.41 × 10^3^ M^−1^). This contrasts with the previously reported 2-substituent effects in benzaldehydes where a significant increase in *K*^exp^ was observed only for 2-heteroatom substituted aldehydes. Breaking each of these equilibrium values down into their constituent rate constants, *k*_1_ and *k*_−1_, the source of the increase in *K*^exp^ is not the same for each aldehyde. For 35 (R = Me, entry 2), a decrease in *k*_1_ (1.93 × 10^−1^ M^−1^ s^−1^ (35) *vs.* 5.88 × 10^−1^ M^−1^ s^−1^ (34)) was observed, but a corresponding 6-fold decrease in *k*_−1_ compared with 34 (4.38 × 10^−5^ s^−1^ (35) *vs.* 2.94 × 10^−4^ s^−1^ (34)) leads to an overall increase in *K*^exp^. A large *K*^exp^ (4.03 × 10^4^ M^−1^) is observed for 36 (R = Br, entry 3) and is a compound effect of large *k*_1_ (3.66 M^−1^ s^−1^) and a small *k*_−1_ (9.09 × 10^−5^ s^−1^), while for 37 (R = F, entry 4), the increase in *K*^exp^ is a result of an increase in *k*_1_ (2.53 M^−1^ s^−1^ (37) *vs.* 5.88 × 10^−1^ M^−1^ s^−1^ (34)) with approximate maintenance of *k*_−1_ (2.69 × 10^−4^ s^−1^ (37) *vs.* 2.94 × 10^−4^ s^−1^ (34)). Similar trends for the reaction constants involved in tetrahedral intermediate formation are observed for reactions of triazolium 42 (Ar = Mes) with 3-substituted aldehydes 35, 36 and 37 (entries 6–9).

The observed increase in *k*_1_ for 36 (R = Br) and 37 (R = F) can in part be attributed to an increase in electrophilicity of aldehyde when an electron-withdrawing group is *ortho* to the aldehyde. Given Br- and F- substituents have closely similar electron withdrawing substituent effects (*e.g.* Hammett *σ*_m_ = +0.39 (Br), +0.34 (F))^18^ the substantially enhanced *k*_1_ and *K*^exp^ for 36 (R = Br) points to an additional steric influence in this case. By twisting out of conjugation with the aldehyde and diminishing the donating effect of the heteroatom lone pair, sterically bulky 2-substituents (such as when R = Br) can additionally increase aldehyde reactivity towards nucleophiles.^19^ Steric effects are also revealed in a comparison of *k*_−1_ values. The decrease in *k*_−1_ for 35 and 36 (R = Me and R = Br) compared with 34 and 37 (R = H and R = F) parallels the observed trend of bulky substituents disfavouring reformation of starting materials from the tetrahedral intermediate.

Overall, these results clearly demonstrate that large 2-substituent effects are also observed for heteroaromatic aldehydes, which further enhance the effect of the 2-heteroatom in favouring formation of the tetrahedral intermediate. The largest combined effect is observed for the combination of the pyridyl nitrogen and *ortho*-bromo substituents with *K*^exp^ for 36 and triazolium 39 almost 4000-fold larger than for benzaldehyde (Table 1, entry 3 and Scheme 2).

#### Position of substituent on pyridine (R*vs.*R′) (Table 1, entries 1, 2 and 5; 6, 7 and 10)

Incorporation of a methyl substituent on the 6-position of the pyridine ring (*ortho* to the ring nitrogen and *meta*- to the aldehyde) (38, R = H, R′ = Me) was investigated in reaction with triazolium salts 39 (Ar = Ph) and 42 (Ar = Mes) (entries 5 and 10). In reaction with 39, 38 (entry 5) gave rate constants (*k*_1_, *k*_−1_) and *K*^exp^ closely similar to 34 (R = R′ = H entry 1), and therefore significantly different from 3-methyl-substituted aldehyde 35 (R = Me, R′ = H, entry 2). For reaction of 38 (R = Me, R′ = H) with 42 (Ar = Mes) (entry 10), the measured rate and equilibrium constants for intermediate formation (*k*_1_ and *K*^exp^) are again close to those measured for the reaction of 42 (Ar = Mes) with 34 (R = R′ = H) (entry 6) suggesting that substitution in this position has minimal effect on the equilibrium between starting materials and the tetrahedral intermediate. Overall, substitution *ortho* to the aldehyde has a substantial impact on rate and equilibrium constants for adduct formation whereas additional substitution *ortho* to the ring nitrogen has a negligible effect.

#### Effect of triazolium *N*-aryl substituent (Ar) (Table 1, entries 1, 6 and 11; 2, 7 and 12; 15 and 17)

As a general trend, our previous results with non-heteroaromatic aldehydes demonstrated that the equilibrium constant for adduct formation (*K*^exp^) increases with the introduction of 2,6-substitution on the *N*-aryl substituent of the triazolium salt.^12^ This effect is ascribed to the forced orthogonality of the substituted aryl group providing less steric hindrance in the plane of the reactive carbene and therefore a more favourable approach of the aldehyde. This trend is maintained for reaction with heteroaromatic aldehydes; for example, in reactions with pyridine aldehyde 34, *K*^exp^ increases from 39 (Ar = Ph, *K*^exp^ = 2.0 × 10^3^ M^−1^), to 42 (Ar = Mes, *K*^exp^ = 2.44 × 10^4^ M^−1^), to 44 (Ar = C_6_Cl_3_H_2_, *K*^exp^ = 4.81 × 10^4^ M^−1^) (Table 1, entries 1, 6, 11). While rate constants *k*_1_ and *k*_−1_ could not be extracted for 44,^20^ the observed increase in *K*^exp^ for 42 compared with 39 coincides with an increase in *k*_1_ and decrease in *k*_−1_. A similar trend is observed when comparing the reactions of 3-methyl substituted pyridine aldehyde 35 (R = Me) with two separate sets of triazolium salts (*n* = 1: 39, 42, 44 (entries 2, 7, 12), and *n* = 2: 40, 43 (entries 15 and 17)). With triazolium salts 39, 42 and 44, *K*^exp^ increases from 39 (Ar = Ph, *K*^exp^ = 4.36 × 10^3^ M^−1^), to 42 (Ar = Mes, *K*^exp^ = 4.82 × 10^4^ M^−1^) to 44 (Ar = C_6_Cl_3_H_2_, *K*^exp^ = 1.04 × 10^5^ M^−1^). For the reaction of 40 and 43 a larger *K*^exp^ is observed when Ar = Mes (43, *K*^exp^ = 7.87 × 10^3^ M^−1^) than when Ar = Ph (40, *K*^exp^ = 4.53 × 10^2^ M^−1^) resulting from both an increase in *k*_1_ and a decrease in *k*_−1_ for 43. For the other aldehydes tested (36 (R = Br), 37 (R = F) and 38 (R′ = Me)), catalyst 42 (*n* = 1, Ar = Mes, entries 8–10) consistently exhibited *K*^exp^ values approximately ten times larger than analogous reactions with 39 (*n* = 1, Ar = Ph, entries 3–5). For each aldehyde, *k*_1_ is approximately the same order of magnitude for 39 and 42, with the difference in *K*^exp^ primarily a consequence of smaller *k*_−1_ values for 42 compared with 39. This indicates that the backward reaction of the intermediate is generally slower for bulky *N*-aryl substituents. Presumably, steric congestion reduces access of the adduct OH to base for initiation of the decomposition reaction.^21^

#### Effect of fused ring size (*n*) (Table 1, entries 1, 13 and 14; 2, 15 and 16)

A distinct effect can be observed moving from a 5- to a 6-membered fused ring (*n* = 1 (39) and *n* = 2 (40)) in their reactions with 34 (entries 1 and 13). With an increase in ring size, significant 10-fold decreases in rate constant *k*_1_ (5.88 × 10^−1^ M^−1^ s^−1^ (39) *vs.* 5.06 × 10^−2^ M^−1^ s^−1^ (40)) and a 3-fold decrease in equilibrium constant *K*^exp^ (2.00 × 10^3^ M^−1^ (39) *vs.* 5.93 × 10^2^ M^−1^ (40)) are observed. Moving from 40 to 41 (*n* = 2 to *n* = 3, entries 13 and 14), the rate constant (*k*_1_) and equilibrium constant (*K*^exp^) for adduct formation again decrease (41: *k*_1_ = 4.30 × 10^−2^ M^−1^ s^−1^, *K*^exp^ = 2.79 × 10^2^ M^−1^) though the difference between 40 and 41 is smaller than between 39 and 40.^22^ Similar trends are observed for the reaction of 39, 40 and 41 with 3-methyl aldehyde 35 (Table 1, entries 2, 15 and 16) and in the reaction of *N*-Mes catalysts 42 (*n* = 1) and 43 (*n* = 2) with 35 (Table 1, entries 9 and 10).

In our recent study of the C_α_-H/D exchange reactions of a large series of twenty bicyclic triazolium salts,^22^ a similar trend was observed with higher key rate constants for the 5-fused series (*n* = 1) and similar, smaller values for the 6- and 7-fused salts (*n* = 2 and *n* = 3). Based on X-ray structural data obtained for the twenty triazolium salts and DFT computational evaluation, these changes were attributed to several structural effects including changes in internal triazolyl NCN angle and positioning of the most proximal CH_2_ with variation in fused ring size. In the case of C_α_-H/D exchange reactions, subtle changes were observed with *k*_ex_ up to 2.2 times larger for *n* = 1 compared with *n* = 2. In this study, the effect is magnified with *k*_1_ and *K*^exp^ in the range of four to ten times larger for *n* = 1 *vs. n* = 2 (39*vs.*40 and 42*vs.*43), and smaller but significant differences observed comparing *n* = 2 with *n* = 3 (40*vs.*41). This enhanced effect highlighted by our present results is likely a result of the greater steric demand for the addition to an aldehyde compared to relatively undemanding H/D exchange process.

#### Effect of heteroatom on fused ring (X) (Table 1, entries 15 and 17–19)

Triazolium salts 45 (Ar = Ph) and 46 (Ar = Mes) based on morpholine were also tested under the model reaction conditions and their reactivity can be compared directly with analogous piperidine-based catalysts 40 (Ar = Ph) and 43 (Ar = Mes). Comparison of 40 with 45 reveals a small but significant 2-fold increase in the equilibrium constant for tetrahedral adduct formation *K*^exp^ (4.53 × 10^2^ M^−1^ (40) *vs.* 8.61 × 10^2^ M^−1^ (45)). A similar 2-fold increase in *K*^exp^ was observed when a methylene group is exchanged for an oxygen atom in *N*-Mes catalysts 43 and 46 (7.87 × 10^3^ M^−1^ (43) *vs.* 1.56 × 10^4^ M^−1^ (46)). In both cases, an increase in *k*_−1_ was observed, but a greater increase in *k*_1_ led to an overall increase in *K*^exp^.

### Forward reaction of tetrahedral adduct (*k*_2_)

For a given aldehyde, the rate constant of the onward reaction of the tetrahedral adduct, *k*_2_, is generally in the same order of magnitude for 39 (*n* = 1, Ar = Ph) and 42 (*n* = 1, Ar = Mes) and, in the cases where it could be measured, was significantly higher for 44 (*n* = 1, Ar = C_6_Cl_3_H_2_) (entries 1, 6 and 11; 2, 7 and 12). Taking pyridine-2-carboxaldehyde 34 to demonstrate (entries 1, 6 and 11), the measured *k*_2_ values for 39 and 42 were roughly similar (*k*_2_ = 1.05 × 10^−4^ s^−1^ (39) and 1.15 × 10^−4^ s^−1^ (42)) but significantly larger for 44 (*k*_2_ = 6.19 × 10^−3^ s^−1^), consistent with electron withdrawing *N*-aryl substituents facilitating deprotonation. Although the trend is maintained for most aldehydes (35 (R = Me), 37 (R = F) and 38 (R′= Me)) in reactions with 39 and 42 (entries 2 and 7; 4 and 9; 5 and 10), using aldehyde 36 (R = Br) a significantly smaller *k*_2_ is observed for 42 (Ar = Mes, *k*_2_ = 9.00 × 10^−6^ s^−1^, entry 13) compared with 39 (Ar = Ph, *k*_2_ = 87.9 × 10^−6^ s^−1^, entry 12). The electron withdrawing electronic effect of an Br substituent *ortho* to the aldehyde would be expected to favour this deprotonation step, thus the observed ∼10-fold decrease in *k*_2_ is attributed to steric factors when 42 is used in combination with aldehydes bearing bulky 2-substituents. This provides another example of additional steric effects present in heteroaromatic aldehydes with *ortho*-bromo substituents, with the magnitude also dependent on the *N*-Ar substituent.

For the size and constitution of the fused ring, decrease in ring size leads to a substantial decrease in *k*_2_ (*e.g.* 1.15 × 10^−4^ s^−1^ (39, *n* = 1) *vs.* 3.94 × 10^−6^ s^−1^ (40, *n* = 2) and negligible (41, *n* = 3) in reaction with 34, entries 1, 13 and 14). Inclusion of oxygen in the fused ring leads to a slight increase in *k*_2_ with either *N*-Ph (2.28 × 10^−6^ s^−1^ (40) *vs.* 1.45 × 10^−5^ s^−1^ (45), entries 15 and 18) or *N*-Mes catalysts (*k*_2_ = 3.61 × 10^−6^ s^−1^ (43) *vs.* 1.86 × 10^−5^ s^−1^ (46, entries 17 and 19).

The effect of aldehyde structure on *k*_2_ was also assessed. In reactions with both 39 (Ar = Ph) and 42 (Ar = Mes), significant decreases in *k*_2_ were observed for 35 (R = Me) when compared with 34 (R = H) (entries 1 and 2; 6 and 7), while increases in *k*_2_ were observed for 37 (R = F) when compared with 34 (R = H) (entries 1 and 4; 5 and 9). Using 39, a slight decrease in *k*_2_ is observed for 36 (R = Br) compared with 34 (R = H) (1.15 × 10^−4^ s^−1^ (34) *vs.* 8.79 × 10^−5^ s^−1^ (36), entries 1 and 3) but in reaction with 42, a more significant 10-fold decease is observed (1.05 × 10^−4^ s^−1^ (34) *vs.* 9.00 × 10^−6^ s^−1^ (36), entries 6 and 8) further highlighting the importance of steric effects in reaction of the tetrahedral adducts. Again, only a slight decrease in *k*_2_ is observed for 38 (R′= Me) *vs.*34 irrespective of triazolium (39 (entries 1 and 5) or 42 (entries 6 and 10)) indicating the minimal effect of 6-substitution on the pyridyl aldehyde.

### Tetrahedral adduct dissociation (*K*^diss^)

To further validate these rate and equilibrium constants, the decay of selected tetrahedral adducts towards equilibrium was studied (Table 2). Analysis of the ^1^H NMR reaction profiles for dissociation of selected adducts of aldehyde 35 allowed rate and equilibrium constants of dissociation to be measured (*k*_d_, s^−1^ and *K*^diss^, M^−1^) and rate constants for association (*k*_a_, M^−1^ s^−1^) to be calculated. In this analysis, although *k*_a_ = *k*_1_ and *k*_d_ = *k*_−1_ a distinction has been made to differentiate between the two methods of measurement for either the forward (addition of NHC to aldehyde) or reverse (dissociation of tetrahedral adduct) processes. The observation of closely similar values for *k*_a_ and *k*_1_, or *k*_d_ and *k*_−1_, through starting from different ends of the equilibrium gives confidence in the absolute and relative magnitudes of these values. For example, the dissociation rate constant *k*_d_ (4.54 × 10^−5^ s^−1^) of tetrahedral adduct 12 (Table 2, entry 1) is close to the reverse rate constant *k*_−1_ (4.38 × 10^−5^ s^−1^) of tetrahedral adduct 12 formation (Table 1, entry 2).

**Table 2:** Equilibrium and rate constants for the dissociation of 3-(hydroxybenzyl)triazolium adducts^a^

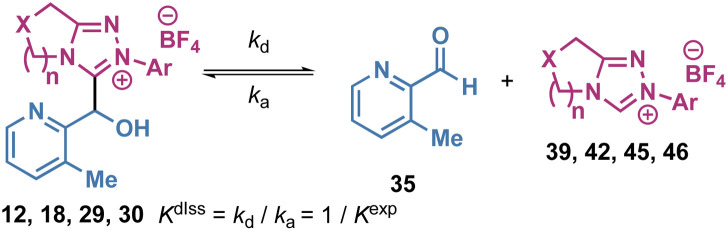									
Entry	Triazolium salt	Tetrahedral adduct	*k*_d_ (×10^−5^ s^−1^)	*k*_d_^fit^ (×10^−5^ s^−1^)	*k*_a_ (×10^−2^ M^−1^ s^−1^)	*k*_a_^fit^ (×10^−2^ M^−1^ s^−1^)	*K* ^diss^ (×10^−5^ M^−1^)	*K* ^fit^ (×10^−5^ M^−1^)	1/*K*^diss^ (×10^2^ M^−1^)
1	39	12	4.54	4.06	20.7	16.6	21.9	24.5	45.7
2	42	18	0.882	0.879	41.8	43.5	2.11	2.02	474
3	45	29	25.6	26.1	23.3	24.8	11.0	10.5	9.09
4	46	30	8.46	8.57	135	136	6.27	6.30	160

a Conditions: tetrahedral adduct 20 mM, Et_3_N : Et_3_N·HCl (2 : 1) 90 mM in CD_3_OD at 25 °C.

The rate constant *k*_a_ (2.07 × 10^−1^ M^−1^ s^−1^) and the reciprocal (4.57 × 10^3^ M^−1^) of equilibrium constant *K*^diss^ of tetrahedral adduct 12 dissociation were also respectively similar to rate constant *k*_1_ (1.93 × 10^−1^ M^−1^ s^−1^) and equilibrium constant *K*^exp^ (4.36 × 10^3^ M^−1^) of adduct 12 formation (*k*_d_ ≈ *k*_−1_, *k*_a_ ≈ *k*_1_, 1/*K*^diss^ ≈ *K*^exp^). The same trend was also observed comparing the corresponding rate and equilibrium constants of adduct formation and dissociation for other triazolium salts (entries 2–4 in Table 2*vs.* entries 7, 18, 19 in Table 1).^23^

## Conclusions

In conclusion, measurements of equilibrium and rate constants for the reaction of triazolium NHC precatalysts with heteroaromatic aldehydes to give tetrahedral 3-(hydroxybenzyl)azolium adducts under stoichiometric conditions have been made. Twenty tetrahedral adducts were isolated and fully characterised including X-ray crystallographic analysis for 13. The results obtained from kinetic analysis and fitting data show that nucleophilic addition into pyridyl aldehydes is fast compared with existing data for addition to benzaldehydes. Notably, the combined effect of the heteroatom of the heterocyclic aldehyde and an additional substituent both *ortho* to the aldehydic position result in exceptionally large enhancements in equilibrium constants for tetrahedral adduct formation. In particular, large effects are observed with both 3-halogen (Br, F) and 3-methyl substituted pyridine-2-carboxaldehydes, which together substantially favour formation of the tetrahedral intermediate relative to benzaldehyde derivatives. Given this initial adduct-forming step is common to all NHC-catalysed transformations of aldehydes involving the BI, the accelerated formation of adduct clearly must underpin the preferential reactivity of heteroaromatic aldehydes. Catalyst and aldehyde structure affect the observed kinetics of adduct formation, decay to starting materials and onward reaction to BI. Key observations indicate that increased steric hindrance lead to a reduction in both *k*_2_ and *k*_−1_ for large (2,6-disubstituted)-*N*-Ar groups and sterically demanding aldehyde substituents in the 3-position, but not in the 6-position of the pyridine-2-carboxaldehyde derivatives. A decrease in the forward reaction constants (*k*_1_, *k*_2_) for larger fused rings (*n* = 2, 3) on the NHC is observed, but an increase in the forward reaction constants is observed upon incorporation of heteroatom in the fused ring. Further work from our laboratories will extend this type of analysis to alternative aldehyde and NHC-catalyst families.

## Data availability

The research data supporting this publication can be accessed at https://doi.org/10.17630/4e21c331-2fa6-440f-8636-f9ed563bda3c.

## Author contributions

ADS and AOD designed the project. ZD, CMY and JZ carried out the experimental work and analysed the data. AMZS carried out X-ray crystallographic analysis. CMY, ADS and AOD wrote the paper with contributions from all authors.

## Conflicts of interest

There are no conflicts to declare.

## Supplementary Material

SC-014-D2SC05704B-s001

SC-014-D2SC05704B-s002
