# Supplementary material for: Rate and equilibrium constants for the addition of triazolium salt derived N-heterocyclic carbenes to heteroaromatic aldehydes
Source: Chem Sci. 2022 Nov 14;14(1):162–70. doi: 10.1039/d2sc05704b (PMC9769090; doi:10.1039/d2sc05704b)
Supplement: SC-014-D2SC05704B-s001 [file SC-014-D2SC05704B-s001.pdf]

Electronic Supplementary Material (ESI) for Chemical Science.
This journal is © The Royal Society of Chemistry 2022

Supporting Information

Rate and Equilibrium Constants for the Addition of Triazolium Salt derived N-Heterocyclic Carbenes to Heteroaromatic Aldehydes

Zhuan Duan,[a] Claire M. Young,[a] Jiayun Zhu,[b] Alexandra M. Z. Slawin,[a] AnnMarie C. O'Donoghue,[b]* and Andrew D. Smith[a]*

[a] School of Chemistry, University of St Andrews, North Haugh, St Andrews KY16 9ST, UK
E-mail: ads10@st-andrews.ac.uk
[b] Department of Chemistry, University Science Laboratories, South Road, Durham, DH1 3LE, UK
E-mail: annmarie.odonoghue@durham.ac.uk

Table of Contents

General Instrumentation 2

1. Synthesis of N-Heterocyclic Carbene Precatalysts 3

2. Isolation of 3-(Hydroxybenzyl)triazolium Adducts 7

3. Determination of Rate and Equilibrium Constants for 3-(Hydroxybenzyl)triazolium Adduct Formation in CD_3OD 24

4. Determination of Rate and Equilibrium Constants: Decay of 3-(Hydroxybenzyl)triazolium Adducts to Equilibrium 89

X-ray Crystal Structure 100

References 101

^1H, $^{13}C\{^1H\}$ and $^{19}F\{^1H\}$ NMR Spectra 102

General Instrumentation

All reactions to synthesize triazolium precatalysts were performed in flame-dried glassware under an N_2 atmosphere. Anhydrous CH_2Cl_2 was obtained from an MBraun SPS-800 system. All other solvents were used without further purification unless otherwise stated. All heterocyclic aldehydes were purified before being used. Room temperature (rt) refers to 20–25°C. All kinetic experiments were conducted under N_2.

Analytical thin layer chromatography was performed on pre-coated aluminium plates (Kieselgel 60 F_{254} silica). Aluminium plates were visualized under UV light (254 nm). Flash column chromatography was performed on Kieselgel 60 silica in the solvent system stated under a positive pressure of compressed air.

Melting points were measured using an Electrothermal 9100 melting point apparatus.

Infrared spectra (ν_{max}) were recorded on a Shimadzu IRAffinity-1 Fourier transform IR spectrophotometer fitted with a Specac Quest ATR accessory (diamond puck) using either thin film or solid, and only characteristic absorption wavenumbers (ν_{max}) were reported.

NMR spectra were recorded on either a Bruker AV400 with a BBFO probe (^{1}H 400 MHz; ^{13}C{^{1}H} 101 MHz; ^{19}F{^{1}H} 377 MHz), a Bruker AVII 400 with a BBFO probe (^{1}H 400 MHz; ^{13}C{^{1}H} 101 MHz; ^{19}F{^{1}H} 376 MHz), a Bruker AVIII-HD 500 with a SmartProbe BBFO + probe (^{1}H 500 MHz, ^{13}C{^{1}H} 126 MHz, ^{19}F{^{1}H} 470 MHz), or a Bruker AVIII 500 with a CryoProbe Prodigy BBO probe (^{1}H 500 MHz, ^{13}C{^{1}H} 126 MHz, ^{19}F 470 MHz) in the deuterated solvent stated. All kinetic data was measured using a Bruker AVIII-HD 500 with a SmartProbe BBFO + probe (^{1}H 500 MHz, ^{13}C{^{1}H} 126 MHz, ^{19}F{^{1}H} 470 MHz). All chemical shifts are quoted in parts per million (ppm) relative to the residual solvent peak. All coupling constants (*J*) are quoted in Hz. Multiplicities are indicated as s (singlet), d (doublet), t (triplet), q (quartet), m (multiplet), and multiples thereof. The abbreviation Ar denotes aromatic and br denotes broad singlet. NMR peak assignments were confirmed using 2D ^{1}H correlated spectroscopy (COSY), 2D ^{1}H-^{13}C heteronuclear single quantum coherence (HSQC), 2D ^{1}H-^{13}C heteronuclear multiple-bond correlation spectroscopy (HMBC).

Mass spectrometry (m/z) data were acquired by electrospray ionisation (ES), and electron impact (EI) at University of St Andrews.

The calculation method used is based on literature (DOI: 10.1002/anie.201501840)[1] and Jiayun Zhu's thesis.[2]

1. Synthesis of N-Heterocyclic Carbene Precatalysts

General procedure A

Based upon a reported procedure,[3, 4] trimethyloxonium tetrafluoroborate (1 equiv.) was added to a flame-dried flask containing a solution of lactam (1 equiv.) in anhydrous CH_2Cl_2 and stirred at room temperature overnight under a N_2 atmosphere. The hydrazine (1 equiv.) was then added and stirred for 2 days before concentrating in vacuo (Some hydrazines were prepared from hydrazine hydrochloride immediately before use through treatment with NaOH solution, extraction with CH_2Cl_2, drying over Na_2SO_4 and concentration in vacuo). The residue was dissolved in methanol and triethyl orthoformate or pure trimethyl orthoformate, and then the solution was refluxed. The heterogeneous mixture was filtered, and the filtrate was concentrated in vacuo to give a solid or oil, then recrystallization from Et_2O and CH_2Cl_2 to give the title compound.

2-Phenyl-6,7-dihydro-5H-pyrrolo[2,1-c][1,2,4]triazol-2-ium tetrafluoroborate 39

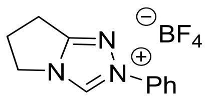

Following general procedure A, trimethyloxonium tetrafluoroborate (414 mg, 2.8 mmol) was added to a flame-dried flask containing a solution of pyrrolidin-2-one (210 μL, 2.72 mmol) in anhydrous CH_2Cl_2 (15 mL) and the reaction was stirred at room temperature overnight under N_2 atmosphere. Phenyl hydrazine (278 μL, 2.8 mmol) was added and the reaction was stirred for 2 days before concentration in vacuo. The residue was dissolved in MeOH (10 mL), and triethyl orthoformate (3 mL) was added then the solution was refluxed for 10 h at 110 °C. The heterogeneous mixture was filtered and the solid was recrystallized from MeOH to give the title compound (494 mg, 67 %) as a beige solid. **mp** 153-155 ºC (154-156 ºC[5]); **IR** ν_{max} (film): 3140, 1589, 1435, 1387, 1229, 1028, 974, 774; **^{1}H NMR** (400 MHz, d_6-DMSO) δ_H: 2.76 (2H, tt, *J* 7.4, C*H*$_2$), 3.22 (2H, t, *J* 7.4, C*H*$_2$), 4.42 (2H, t, *J* 7.4, C*H*$_2$), 7.61-7.66 (H, m, Ar*H*), 7.68-7.73 (2H, m, Ar*H*), 7.87-7.91 (2H, m, Ar*H*), 10.70 (1H, s, C*H*). Data in accordance with literature.[5]

2-Phenyl-5,6,7,8-tetrahydro-[1,2,4]triazolo[4,3-a]pyridin-2-ium tetrafluoroborate 40

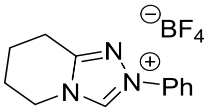

Following general procedure A, trimethyloxonium tetrafluoroborate (888 mg, 6.0 mmol) was added to a flame-dried flask containing a solution of piperidin-2-one (594 mg, 6.0 mmol) in anhydrous CH_2Cl_2 (25 mL) and the reaction was stirred at room temperature overnight under N_2 atmosphere. Phenyl hydrazine (648 mg, 6.0 mmol) was added and the reaction was stirred for 2 days before concentration in vacuo. The residue was dissolved in trimethyl orthoformate (10 mL) and the solution was refluxed overnight at 110 ˚C. The heterogeneous mixture was filtered and the solid was recrystallized from dichloromethane and diethyl ether to give triazolium salt (560 mg, 33%) as a white solid. **mp** 154-157 ºC (155-159 ºC[6]); **IR** ν_{max} (film): 3142, 1585, 1402, 1227, 1032, 976, 862, 762; **^{1}H NMR** (400 MHz, d_6-DMSO) δ_H: 1.94-2.08 (4H, m, 2C*H*$_2$), 3.10 (2H, t, *J* 8.0, C*H*$_2$), 4.31 (2H, t, *J* 8.0, C*H*$_2$), 7.61-7.66 (H, m, Ar*H*), 7.68-7.73 (2H, m, Ar*H*), 7.87-7.90 (2H, m, Ar*H*), 10.74 (1H, s, C*H*). Data in accordance with literature.[6]

2-Phenyl-6,7,8,9-tetrahydro-5H-[1,2,4]triazolo[4,3-a]azepin-2-ium tetrafluoroborate 41

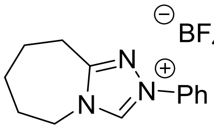

Following general procedure A, trimethyloxonium tetrafluoroborate (888 mg, 6.0 mmol) was added to a flame-dried flask containing a solution of caprolactam (678 mg, 6.0 mmol) in anhydrous CH_2Cl_2 (25 mL) and the reaction was stirred at room temperature overnight under N_2 atmosphere. Phenyl hydrazine (648 mg, 6.0 mmol) was added and the reaction was stirred for 2 days before concentration in vacuo. The residue was dissolved in trimethyl orthoformate (10 mL) and the solution was refluxed overnight at 110 ˚C. The heterogeneous mixture was filtered and the solid was recrystallized from dichloromethane and diethyl ether to give triazolium salt (1.37 g, 76%) as a white solid. **mp** 146-148 ºC; **IR** ν_{max} (film): 3113, 1591, 1441, 1221, 1022, 968, 916, 810, 760; **^{1}H NMR** (400 MHz, d_6-DMSO) δ_H: 1.73-1.80 (2H, m, C*H*$_2$), 1.90-1.92 (4H, m, 2C*H*$_2$), 3.17 (2H, t, *J* 4.0, C*H*$_2$), 4.39 (2H, t, *J* 4.0, C*H*$_2$), 7.60-7.65 (H, m, Ar*H*), 7.68-7.73 (2H, m, Ar*H*), 7.86-7.89 (2H, m, Ar*H*), 10.69 (1H, s, C*H*). Data in accordance

with literature.[6]

2-Mesityl-6,7-dihydro-5H-pyrrolo[2,1-c][1,2,4]triazol-2-ium tetrafluoroborate 42

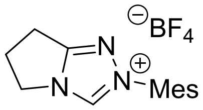

Following general procedure A, trimethyloxonium tetrafluoroborate (1.48 g, 10.0 mmol) was added to a flame-dried flask containing a solution of pyrrolidin-2-one (850 mg, 10.0 mmol) in anhydrous CH_2Cl_2 (25 mL) and the reaction was stirred at room temperature overnight under N_2 atmosphere. Freshly prepared mesitylhydrazine (1.50 g, 10.0 mmol) was added and the reaction was stirred for 2 days before concentration in vacuo. The residue was dissolved in trimethyl orthoformate (15 mL) with 4 drops of $HBF_4·Et_2O$ and the solution was refluxed for two days at 110 ˚C. The heterogeneous mixture was filtered and the solid was recrystallized from dichloromethane and diethyl ether to give triazolium salt (1.59 g, 50%) as a beige solid. **mp** 176-180 ºC (174-176 ºC[7]); **IR** ν_{max} (film): 3148, 2978, 1589, 1387, 1198, 1030, 854; **^{1}H NMR** (400 MHz, d$_6$-DMSO) δ_H: 2.07 (6H, s, 2 × C*H₃*), 2.35 (3H, s, C*H₃*), 2.73-2.80 (2H, m, C*H₂*), 3.20 (2H, t, *J* 7.7, C*H₂*), 4.45 (2H, t, *J* 7.4, C*H₂*), 7.16 (2H, s, 2 × Ar*H*), 10.70 (1H, s, C*H*). Data in accordance with literature.[7]

2-Mesityl-5,6,7,8-tetrahydro-[1,2,4]triazolo[4,3-a]pyridin-2-ium tetrafluoroborate 43

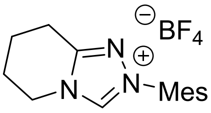

Following general procedure A, trimethyloxonium tetrafluoroborate (0.74 g, 5.0 mmol) was added to a flame-dried flask containing a solution of piperidin-2-one (0.50 g, 5.0 mmol) in anhydrous CH_2Cl_2 (15 mL) and the reaction was stirred at room temperature overnight under N_2 atmosphere. Freshly prepared mesitylhydrazine (0.75 g, 5.0 mmol) was added and the reaction was stirred for 2 days before concentration in vacuo. The residue was dissolved in trimethyl orthoformate (8 mL) with 4 drops of $HBF_4·Et_2O$, 4Å molecular sieves were added and reaction mixture was refluxed for 12 h at 110 ˚C. The heterogeneous mixture was filtered and the solid was recrystallized from dichloromethane and diethyl ether to give triazolium salt (510 mg, 16%) as a white solid. **mp** 160-162 ºC; **IR** ν_{max} (film): 3142, 2960, 1581, 1447, 1219, 1051, 843; **^{1}H NMR** (400 MHz, d$_6$-DMSO) δ_H: 1.99-2.06 (10H, m, 2 × CH_3 + 2 × CH_2), 2.34 (3H, s,

CH_3), 3.05-3.08 (2H, m, CH_2), 4.34-4.37 (2H, m, CH_2), 7.15 (2H, s, 2 × Ar*H*), 10.24 (1H, s, C*H*). **$^{13}C^3$ NMR** (101 MHz, d_6-DMSO) δ_C: 17.4, 18.7, 21.0, 21.1, 21.2, 46.0, 129.8, 131.8, 135.3, 141.6, 144.3, 153.9.

2-(2,4,6-Trichlorophenyl)-6,7-dihydro-5H-pyrrolo[2,1-c][1,2,4]triazol-2-ium tetrafluoroborate 44

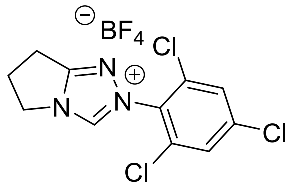

Following general procedure A, trimethyloxonium tetrafluoroborate (1.48 g, 10.0 mmol) was added to a flame-dried flask containing a solution of pyrrolidin-2-one (850 mg, 10.0 mmol) in anhydrous CH_2Cl_2 (25 mL) and the reaction was stirred at room temperature overnight under N_2 atmosphere. 2,4,6-Trichlorophenyl hydrazine (2.10 g, 10.0 mmol) was added and the reaction was stirred for 2 days before concentration in vacuo. The residue was dissolved in trimethyl orthoformate (15 mL), 4Å molecular sieves were added and reaction mixture was refluxed for 5-6h at 110 ˚C. The heterogeneous mixture was filtered and the solid was recrystallized from dichloromethane and diethyl ether to give triazolium salt (1.35 g, 36%) as a beige solid. **mp** 185-188 ºC; **IR** ν_{max} (film): 3144, 1597, 1560, 1418, 1204, 1030, 824; **^{1}H NMR** (400 MHz, d_6-DMSO) δ_H: 2.73-2.80 (2H, m, CH_2), 3.27 (2H, t, *J* 7.7, CH_2), 4.53 (2H, t, *J* 7.4, CH_2), 8.18 (H, s, 2 × Ar*H*), 10.47 (1H, s, C*H*). Data in accordance with literature.[8]

2-Phenyl-5,6-dihydro-8H-[1,2,4]triazolo[3,4-c][1,4]oxazin-2-ium tetrafluoroborate 45

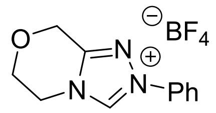

Following general procedure A, trimethyloxonium tetrafluoroborate (1.48 g, 10.0 mmol) was added to a flame-dried flask containing a solution of morpholin-3-one (1.01 g, 10.0 mmol) in anhydrous CH_2Cl_2 (25 mL) and the reaction was stirred at room temperature overnight under N_2 atmosphere. Phenyl hydrazine (1.08 g, 10.0 mmol) was added and the reaction was stirred for 1 days before concentration in vacuo. The residue was dissolved in trimethyl orthoformate (10 mL), 4Å molecular sieves were added and reaction mixture was refluxed for 5 h at 110 ˚C.

The heterogeneous mixture was filtered and the solid was recrystallized from dichloromethane and diethyl ether to give triazolium salt (1.43 g, 49%) as a yellow solid. **mp** 145-146 ºC; **IR** ν_{max} (film): 3140, 1591, 1410, 1219, 1034, 858; **^{1}H NMR** (400 MHz, d_6-DMSO) δ_H: 4.18 (2H, t, *J* 5.2, C*H*$_2$), 4.43 (2H, t, *J* 5.2, C*H*$_2$), 5.19 (2H, s, C*H*$_2$), 7.64-7.75 (3H, m, 3 × Ar*H*), 7.88-7.91 (2H, m, 2 × Ar*H*), 10.91 (1H, s, C*H*). **^{13}C{^{1}H} NMR** (101 MHz, d_6-DMSO) δ_C: 45.1, 61.7, 62.9, 121.1, 130.9, 131.1, 135.4, 141.8, 150.3.

2-Mesityl-5,6-dihydro-8H-[1,2,4]triazolo[3,4-c][1,4]oxazin-2-ium tetrafluoroborate 46

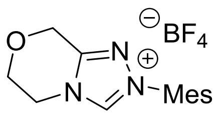

Following general procedure A, trimethyloxonium tetrafluoroborate (1.48 g, 10.0 mmol) was added to a flame-dried flask containing a solution of morpholin-3-one (1.01 g, 10.0 mmol) in anhydrous CH_2Cl_2 (25 mL) and the reaction was stirred at room temperature overnight under N_2 atmosphere. Freshly prepared mesitylhydrazine (1.50 g, 10.0 mmol) was added and the reaction was stirred for 1 days before concentration in vacuo. The residue was dissolved in trimethyl orthoformate (10 mL), 4Å molecular sieves were added and reaction mixture was refluxed for 5 h at 110 ˚C. The heterogeneous mixture was filtered and the solid was recrystallized from dichloromethane and diethyl ether to give triazolium salt (1.13 g, 34%) as a yellow solid. **mp** 190-191 ºC; **IR** ν_{max} (film): 3169, 2978, 1582, 1331, 1194, 1032, 856; **^{1}H NMR** (400 MHz, d_6-DMSO) δ_H: 2.08 (6H, s, 2 × C*H*$_3$), 2.35 (3H, s, C*H*$_3$), 4.21 (2H, t, *J* 5.2, C*H*$_2$), 4.47 (2H, t, *J* 5.2, C*H*$_2$), 5.15 (2H, s, C*H*$_2$), 7.17 (2H, s, 2 × Ar*H*), 10.43 (1H, s, C*H*). **^{13}C{^{1}H} NMR** (101 MHz, d_6-DMSO) δ_C: 17.4, 21.1, 45.2, 61.8, 62.8, 129.8, 131.7, 135.4, 141.8, 144.9, 150.6.

2. Isolation of 3-(Hydroxybenzyl)triazolium Adducts

General procedure B

Based on reported literature procedures,[9, 10, 11] the requisite aldehydes (1.0 equiv.) was added to a solution of NHC precatalyst (1.0 equiv.) and Et_3N (2.0 equiv.) in CH_2Cl_2, and after 0.5-3 hours, a small amount of hydrochloric acid (2 M) was added to stop the reaction. The solvent was removed under reduced pressure, and the crude material was purified by column

chromatography to give the titled compound.

3-(Hydroxy(pyridin-2-yl)methyl)-2-phenyl-6,7-dihydro-5H-pyrrolo[2,1-c][1,2,4]triazol-2-ium tetrafluoroborate 11

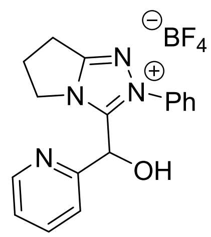

Following general procedure B, picolinaldehyde (57 μL, 0.6 mmol) was added to a solution of NHC precatalyst (164 mg, 0.6 mmol) and Et_3N (168 μL, 1.2 mmol) in CH_2Cl_2 (10 mL). After stirring at rt for 30 mins, 4 drops aqueous 2 M HCl was added to quench the reaction and then the mixture was concentrated under reduced pressure. The crude product was purified by column chromatography (50:1 chloroform:methanol) to yield the title compound as a pale yellow oil (116 mg, 51%). **IR** ν_{max} (film): 3445, 3065, 2556, 1589, 1285, 1047, 867, 764; **^{1}H NMR** (400 MHz, CD_3OD) δ_H: 2.77-2.94 (2H, m, $NCH_2C\mathit{H_2}$), 3.22-3.27 (2H, m, $NCH_2CH_2C\mathit{H_2}$), 4.26 (1H, ddd, *J* 12.2, 8.5, 6.3, $NC\mathit{H_A}H_B$), 4.58 (1H, ddd, *J* 12.2, 8.5, 6.3, $NCH_A\mathit{H_B}$), 6.32 (1H, s, C*H*), 7.38-7.42 (1H, m, 5-Ar*H*), 7.55-7.65 (6H, m, NAr*H* + 6-Ar*H*), 7.86-7.90 (1H, m, 3-Ar*H*), 8.51-8.52 (1H, m, 4-Ar*H*); **^{13}C{^{1}H} NMR** (101 MHz, CD_3OD) δ_C: 21.0 ($NCH_2CH_2\mathit{C}H_2$), 26.6 ($NCH_2\mathit{C}H_2$), 47.8 ($N\mathit{C}H_2$), 68.0 (*C*H), 122.1 (3-Ar*C*H), 124.2 (4-Ar*C*H), 125.5 (2 × NAr*C*H), 129.6 (2 × NAr*C*H), 131.3 (NAr*C*H), 135.4 (NAr*C*), 137.9 (5-Ar*C*H), 149.3 (6-Ar*C*H), 151.5 (N*C*(3)N), 155.9 (Ar*C*), 162.7 (N*C*(7a)N); **HRMS** (ESI^+) $C_{17}H_{17}N_4O$ $[M-BF_4]^+$ found 293.1395, requires 293.1397 (–0.6 ppm).

3-(Hydroxy(3-methylpyridin-2-yl)methyl)-2-phenyl-6,7-dihydro-5H-pyrrolo[2,1-c][1,2,4]triazol-2-ium tetrafluoroborate 12

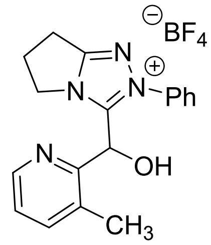

Following general procedure B, 3-methylpicolinaldehyde (67 μL, 0.6 mmol) was added to a

solution of NHC precatalyst (164 mg, 0.6 mmol) and Et_3N (168 µL, 1.2 mmol) in CH_2Cl_2 (10 mL). After stirring at rt for 30 mins, 4 drops aqueous 2 M HCl was added to quench the reaction and then the mixture was concentrated under reduced pressure. The crude product was purified by column chromatography (50:1 chloroform:methanol) to yield the title compound as a colourless oil (158 mg, 67%). **IR** ν_{max} (film): 3389, 1603, 1398, 1281, 1045, 872; **^{1}H NMR** (400 MHz, CD_3OD) δ_H: 2.26 (3H, s, C*H₃*), 2.90-2.97 (2H, m, NCH_2CH_2), 3.27-3.34 (3H, m, =CCH_2 + CH_3OH), 4.61-4.73 (2H, m, NCH_2), 6.46 (1H, s, C*H*), 7.28-7.33 (3H, m, 2,6-NAr*H* + 4-Ar*H*), 7.47-7.52 (2H, m, 3,5-NAr*H*), 7.56-7.60 (1H, m, 6-Ar*H*), 7.64 (1H, ddd, *J* 7.1, 1.6, 0.8, 4-Ar*H*), 8.34 (1H, ddd, *J* 4.8, 1.7, 0.7, 5-Ar*H*); **^{13}C{^{1}H} NMR** (101 MHz, CD_3OD) δ_C: 16.1 (CH_3), 21.0 (=CCH_2), 26.7 (NCH_2CH_2), 48.6 (NCH_2), 67.6 (*C*H), 124.7 (4-NAr*C*H), 125.0 (2,6-NAr*C*H), 129.7 (3,5-NAr*C*H), 131.3 (6-Ar*C*H), 132.8 (2-Ar*C*), 135.2 (1-NAr*C*), 139.5 (4-Ar*C*H), 146.8 (5-Ar*C*H), 152.7 (N*C*(3)N), 153.9 (3-Ar*C*), 162.6 (N*C*(7a)N); **HRMS** (ESI$^+$) $C_{18}H_{19}N_4O$ [M-BF_4]$^+$ found 307.1547, requires 307.1553 (–2.2 ppm).

3-((3-Bromopyridin-2-yl)(hydroxy)methyl)-2-phenyl-6,7-dihydro-5H-pyrrolo[2,1-c][1,2,4]triazol-2-ium tetrafluoroborate 13

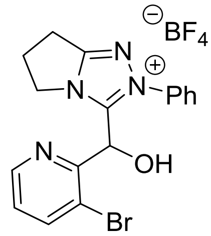

Following general procedure B, 3-bromopicolinaldehyde (56 mg, 0.3 mmol) was added to a solution of NHC precatalyst (82 mg, 0.3 mmol) and Et_3N (84 µL, 0.6 mmol) in CH_2Cl_2 (10 mL). After stirring at rt for 30 mins, 4 drops aqueous 2 M HCl was added to quench the reaction and then the mixture was concentrated under reduced pressure. The crude product was purified by column chromatography (50:1 chloroform:methanol) to yield the title compound as a colourless oil (93 mg, 68%). **IR** ν_{max} (film): 3447, 3067, 1595, 1429, 1283, 1020, 804; **^{1}H NMR** (400 MHz, CD_3OD) δ_H: 2.90-2.98 (2H, m, NCH_2CH_2), 3.25-3.32 (2H, m, =CCH_2), 4.71-4.74 (2H, m, NCH_2), 6.67 (1H, s, C*H*), 7.31-7.34 (3H, m, 3,5-NAr*H* + 4-Ar*H*), 7.49-7.54 (2H, m, 2,6-NAr*H*), 7.56-7.61 (1H, m, 4-NAr*H*), 8.01 (1H, dd, *J* 8.1, 1.4, 4-Ar*H*), 8.50 (1H, dd, *J* 4.7, 1.4, 5-Ar*H*); **^{13}C{^{1}H} NMR** (101 MHz, CD_3OD) δ_C: 21.0 (=CCH_2), 26.7 (NCH_2CH_2), 48.9

(NCH_2), 68.8 (*C*H), 120.3 (3-Ar*C*), 125.0 (3,5-NAr*C*H), 126.1 (6-Ar*C*H), 129.8 (2,6-NAr*C*H), 131.3 (4-NAr*C*H), 135.0 (1-NAr*C*), 141.8 (4-Ar*C*H), 148.4 (5-Ar*C*H), 151.9 (N*C*(3)N), 153.8 (2-Ar*C*), 162.8 (N*C*(7a)N); **HRMS** (ESI^+) $C_{17}H_{16}BrN_4O$ $[M-BF_4]^+$ found 371.0502, requires 371.0502 (0 ppm).

3-((3-Fluoropyridin-2-yl)(hydroxy)methyl)-2-phenyl-6,7-dihydro-5H-pyrrolo[2,1-c][1,2,4]triazol-2-ium tetrafluoroborate 14

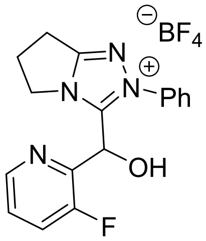

Following general procedure B, 3-fluoropicolinaldehyde (38 mg, 0.3 mmol) was added to a solution of NHC precatalyst (82 mg, 0.3 mmol) and Et_3N (84 µL, 0.6 mmol) in CH_2Cl_2 (10 mL). After stirring at rt for 30 mins, 4 drops aqueous 2 M HCl was added to quench the reaction and then the mixture was concentrated under reduced pressure. The crude product was purified by column chromatography (50:1 chloroform:methanol) to yield the title compound as a yellow oil (64 mg, 54%). **IR** ν_{max} (film): 3429, 3076, 1597, 1452, 1045, 810; **^{1}H NMR** (400 MHz, $CDCl_3$) δ_H: 2.84-3.04 (2H, m, NCH_2CH_2), 3.19-3.36 (2H, m, $=CCH_2$), 4.62 (1H, ddd, *J* 12.3, 8.7, 5.4, NCH_AH_B), 4.84 (1H, ddd, *J* 12.3, 8.6, 6.8, NCH_AH_B), 5.70 (1H, s, O*H*), 6.52 (1H, s, C*H*), 7.30-7.34 (2H, m, 3,5-NAr*H*), 7.40-7.50 (4H, m, 2,6-NAr*H* + 4,6-Ar*H*), 7.52-7.56 (1H, m, 4-NAr*H*), 8.33 (1H, dd, *J* 4.7, 1.4, 5-Ar*H*); **^{13}C{^{1}H} NMR** (101 MHz, $CDCl_3$) δ_C: 21.7 ($=CCH_2$), 27.0 (NCH_2CH_2), 48.6 (NCH_2), 64.9 (*C*H), 124.8 (d, 4-Ar*C*H), 125.6 (3,5-NAr*C*H), 126.8 (d, 6-Ar*C*H), 129.9 (2,6-NAr*C*H), 131.7 (4-NAr*C*H), 134.8 (1-NAr*C*), 143.0 (d, 3-Ar*C*), 145.4 (d, 5-Ar*C*H), 151.3 (N*C*(3)N), 155.9 (2-Ar*C*), 158.5 (2-Ar*C*), 162.1 (N*C*(7a)N); **^{19}F NMR** (377 MHz, $CDCl_3$) δ_F: -123.5 (s, ArF), -153.7 (s, BF_4); **HRMS** (ESI^+) $C_{17}H_{16}FN_4O$ $[M-BF_4]^+$ found 311.1294, requires 311.1303 (-2.7 ppm).

3-(Hydroxy(6-methylpyridin-2-yl)methyl)-2-phenyl-6,7-dihydro-5H-pyrrolo[2,1-c][1,2,4]triazol-2-ium tetrafluoroborate 15

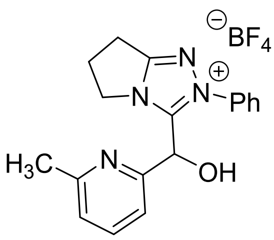

Following general procedure B, 6-methylpicolinaldehyde (72 mg, 0.6 mmol) was added to a solution of NHC precatalyst (164 mg, 0.6 mmol) and Et_3N (168 μL, 1.2 mmol) in CH_2Cl_2 (10 mL). After stirring at rt for 30 mins, 4 drops aqueous 2 M HCl was added to quench the reaction and then the mixture was concentrated under reduced pressure. The crude product was purified by column chromatography (50:1 chloroform:methanol) to yield the title compound as a colourless oil (123 mg, 52%). **IR** ν_{max} (film): 3453, 1595, 1458, 1283, 1047, 870; **^{1}H NMR** (400 MHz, CD_3OD) δ_H: 2.47 (3H, s, C*H₃*), 2.78-2.94 (2H, m, NCH_2CH_2), 3.26 (2H, t, *J* 7.8, =CCH_2), 4.28 (1H, ddd, *J* 12.2, 8.5, 6.4, NCH_AH_B), 4.58 (1H, ddd, *J* 12.2, 8.4, 6.4, NCH_AH_B), 6.26 (1H, s, C*H*), 7.24 (1H, d, *J* 7.8, 5-Ar*H*), 7.43 (1H, dq, *J* 7.7, 0.8, 3-Ar*H*), 7.57-7.68 (5H, m, 2,6-NAr*H* + 3,5-NAr*H* + 4-NAr*H*), 7.75 (1H, t, *J* 7.7, 4-Ar*H*); **^{13}C{^{1}H} NMR** (101 MHz, CD_3OD) δ_C: 21.0 (=CCH_2), 22.7 (CH_3), 26.6 (NCH_2CH_2), 47.9 (NCH_2), 67.9 (*C*H), 118.8 (3-Ar*C*H), 123.6 (5-Ar*C*H), 125.6 (2,6-NAr*C*H), 129.5 (3,5-NAr*C*H), 131.2 (4-NAr*C*H), 135.5 (1-NAr*C*), 138.0 (4-Ar*C*H), 151.5 (N*C*(3)N), 155.1 (2-Ar*C*), 158.7 (6-Ar*C*H), 162.7 (N*C*(7a)N); **HRMS** (ESI^+) $C_{18}H_{19}N_4O$ $[M-BF_4]^+$ found 307.1543, requires 307.1553 (–3.3 ppm).

3-(Furan-2-yl(hydroxy)methyl)-2-phenyl-6,7-dihydro-5H-pyrrolo[2,1-c][1,2,4]triazol-2-ium tetrafluoroborate 16

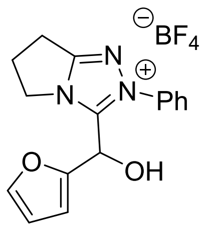

Following general procedure B, furan-2-carbaldehyde (50 μL, 0.6 mmol) was added to a solution of NHC precatalyst (164 mg, 0.6 mmol) and Et_3N (168 μL, 1.2 mmol) in CH_2Cl_2 (10 mL). After stirring at rt for 30 mins, 4 drops aqueous 2 M HCl was added to quench the reaction and then the mixture was concentrated under reduced pressure. The crude product was purified by column chromatography (50:1 chloroform:methanol) to yield the title compound as a pale

yellow oil (28 mg, 13%). **IR** ν_{max} (film): 3462, 3129, 1591, 1499, 1393, 1285, 1038, 930, 764; **^{1}H NMR** (400 MHz, CD_3OD) δ_H: 2.86-2.94 (2H, m, NCH$_2$C*H$_2$*), 3.23-3.27 (2H, m, =CC*H$_2$*), 4.56-4.69 (2H, m, NC*H$_2$*), 6.23 (1H, s, C*H*), 6.41 (1H, dd, *J* 3.4, 1.9, OCHC*H*), 6.45-6.46 (1H, m, OCC*H*), 7.49-7.52 (2H, m, Ar*H*), 7.56 (1H, dd, *J* 1.9, 0.8, OC*H*), 7.58-7.66(3H, m, Ar*H*); **^{13}C{^{1}H} NMR** (101 MHz, CD_3OD) δ_C: 21.0 (=C*C*H$_2$), 26.6 (NCH$_2$*C*H$_2$), 48.6 (N*C*H$_2$), 61.6 (*C*H), 110.5 (OCH*C*H), 110.7 (OC*C*H), 125.4 (2 × NAr*C*H), 129.6 (2 × NAr*C*H), 131.3 (NAr*C*H), 135.2 (NAr*C*), 144.3 (O*C*HCH), 148.4 (O*C*CH), 150.0 (N*C*(3)N), 162.7 (N*C*(7a)N); **HRMS** (ESI$^+$) $C_{16}H_{16}N_3O_2$ [M-BF$_4$]$^+$ found 282.1231, requires 282.1237 (–2.1 ppm).

3-(Hydroxy(pyridin-2-yl)methyl)-2-mesityl-6,7-dihydro-5H-pyrrolo[2,1-c][1,2,4]triazol-2-ium tetrafluoroborate 17

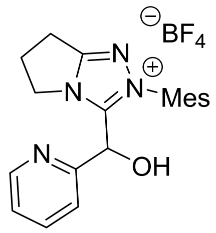

Following general procedure B, picolinaldehyde (57 µL, 0.6 mmol) was added to a solution of NHC precatalyst (189 mg, 0.6 mmol) and Et_3N (168 µL, 1.2 mmol) in CH_2Cl_2 (10 mL). After stirring at rt for 30 mins, 4 drops aqueous 2 M HCl was added to quench the reaction and then the mixture was concentrated under reduced pressure. The crude product was purified by column chromatography (50:1 chloroform:methanol) to yield the title compound as a pale yellow oil (152 mg, 60%). **IR** ν_{max} (film): 3441, 1589, 1439, 1387, 1283, 1049, 854, 758; **^{1}H NMR** (400 MHz, CD_3OD) δ_H: 1.79 (3H, s, 2,6-C*H$_3$*NAr), 2.11 (3H, s, 2,6-C*H$_3$*NAr), 2.37 (3H, s, 4-C*H$_3$*NAr), 2.84-2.97 (2H, m, NCH$_2$C*H$_2$*), 3.29 (2H, t, *J* 7.8, =CC*H$_2$*), 4.39-4.46 (1H, m, NC*H$_A$*H$_B$), 4.59-4.66 (1H, m, NCH$_A$*H$_B$*), 5.93 (1H, s, C*H*), 7.01 (1H, s, NAr*H*), 7.14 (1H, s, NAr*H*) 7.40-7.45 (2H, m, 3, 4-Ar*H*), 7.85 (1H, td, *J* 7.8, 1.8, 5-Ar*H*), 8.52 (1H, ddd, J 4.9, 1.8, 0.9, 6-Ar*H*); **^{13}C{^{1}H} NMR** (101 MHz, CD_3OD) δ_C: 15.8 (2,6-*C*H$_3$NAr), 15.9 (2,6-*C*H$_3$NAr), 19.8 (4-*C*H$_3$NAr), 21.2 (=C*C*H$_2$), 26.5 (NCH$_2$*C*H$_2$), 48.1 (N*C*H$_2$), 68.1 (*C*H), 121.9 (3-Ar*C*H), 124.3 (4-Ar*C*H), 129.2 (3,5-NAr*C*H), 129.4 (3,5-NAr*C*H), 130.7 (1-NAr*C*), 135.0 (2,6-NAr*C*), 136.2 (2,6-NAr*C*), 137.8 (5-Ar*C*H), 142.4 (4-NAr*C*), 149.5 (6-Ar*C*H), 152.3 (N*C*(3)N), 155.7 (Ar*C*), 163.5 (N*C*(7a)N); **HRMS** (ESI$^+$) $C_{20}H_{23}N_4O$ [M-BF$_4$]$^+$ found 335.1862, requires

335.1866 (–1.3 ppm).

3-(Hydroxy(3-methylpyridin-2-yl)methyl)-2-mesityl-6,7-dihydro-5H-pyrrolo[2,1-c][1,2,4]triazol-2-ium tetrafluoroborate 18

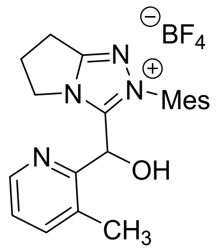

Following general procedure B, 3-methylpicolinaldehyde (67 µL, 0.6 mmol) was added to a solution of NHC precatalyst (189 mg, 0.6 mmol) and Et_3N (168 µL, 1.2 mmol) in CH_2Cl_2 (10 mL). After stirring at rt for 30 mins, 4 drops aqueous 2 M HCl was added to quench the reaction and then the mixture was concentrated under reduced pressure. The crude product was purified by column chromatography (50:1 chloroform:methanol) to yield the title compound as a colourless oil (215 mg, 82%). **IR** ν_{max} (film): 3445, 2926, 1593, 1456, 1283, 1045, 856; **^{1}H NMR** (400 MHz, CD_3OD) δ_H: 1.36 (3H, s, 2,6-C*H*$_3$NAr), 1.99 (3H, s, 3-C*H*$_3$Ar), 2.23 (3H, s, 2,6-C*H*$_3$NAr), 2.36 (3H, s, 4-C*H*$_3$NAr), 2.93-3.01 (2H, m, NCH$_2$C*H*$_2$), 3.31-3.35 (2.55H, m, =CC*H*$_2$ + C*H*$_3$OH), 4.71-4.85 (2H, m, NC*H*$_2$), 6.05 (1H, s, C*H*), 6.92 (1H, s, 3,5-NAr*H*), 7.20 (1H, s, 3,5-NAr*H*), 7.33 (1H, ddd, *J* 8.0, 4.8, 1.4, 6-Ar*H*), 7.62 (1H, d, *J* 7.7, 4-Ar*H*), 8.39 (1H, d, *J* 4.4, 5-Ar*H*); **^{13}C{^{1}H} NMR** (101 MHz, CD_3OD) δ_C: 15.3 (2,6-*C*H$_3$NAr), 15.8 (2,6-*C*H$_3$NAr), 15.9 (3-*C*H$_3$Ar), 19.8 (4-*C*H$_3$NAr), 21.2 (=C*C*H$_2$), 26.6 (NCH$_2$*C*H$_2$), 48.6 (N*C*H$_2$), 66.3 (*C*H), 124.7 (6-Ar*C*H), 129.4 (3,5-NAr*C*H), 129.5 (3,5-NAr*C*H), 130.3 (1-NAr*C*), 132.6 (2-Ar*C*), 134.9 (2,6-NAr*C*), 136.2 (2,6-NAr*C*), 139.3 (4-Ar*C*H), 142.6 (4-NAr*C*), 147.3 (5-Ar*C*H), 153.4 (N*C*(3)N), 153.4 (3-Ar*C*), 163.6 (N*C*(7a)N); **HRMS** (ESI$^+$) $C_{21}H_{25}N_4O$ [M-BF$_4$]$^+$ found 349.2015, requires 349.2023 (–2.1 ppm).

3-((3-Bromopyridin-2-yl)(hydroxy)methyl)-2-mesityl-6,7-dihydro-5H-pyrrolo[2,1-c][1,2,4]triazol-2-ium tetrafluoroborate 19

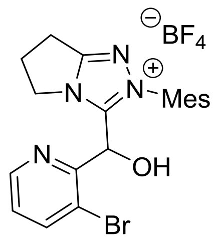

Following general procedure B, 3-bromopicolinaldehyde (56 mg, 0.3 mmol) was added to a solution of NHC precatalyst (95 mg, 0.3 mmol) and Et_3N (84 µL, 0.6 mmol) in CH_2Cl_2 (10 mL). After stirring at rt for 30 mins, 4 drops aqueous 2 M HCl was added to quench the reaction and then the mixture was concentrated under reduced pressure. The crude product was purified by column chromatography (50:1 chloroform:methanol) to yield the title compound as a colourless oil (114 mg, 76%). **IR** ν_{max} (film): 3588, 2942, 1591, 1431, 1283, 1020, 804; **^{1}H NMR** (400 MHz, CD_3OD) δ_H: 1.39 (3H, s, 2,6-C*H$_3$*NAr), 2.23 (3H, s, 2,6-C*H$_3$*NAr), 2.36 (3H, s, 4-C*H$_3$*NAr), 2.94-3.02 (2H, m, NCH$_2$C*H$_2$*), 3.31-3.35 (3.4H, m, =CC*H$_2$* + C*H$_3$*OH), 4.75-4.94 (5.4H, m, NC*H$_2$* + *H$_2$*O), 6.33 (1H, s, C*H*), 6.92-6.93 (1H, m, 3,5-NAr*H*), 7.19-7.20 (1H, m, 3,5-NAr*H*), 7.36 (1H, dd, *J* 8.2, 4.6, 6-Ar*H*), 8.03 (1H, dd, *J* 8.2, 1.4, 4-Ar*H*), 8.56 (1H, dd, *J* 4.7, 1.4, 5-Ar*H*); **^{13}C{^{1}H} NMR** (101 MHz, CD_3OD) δ_C: 15.4 (2,6-*C*H$_3$NAr), 15.8 (2,6-*C*H$_3$NAr), 19.8 (4-*C*H$_3$NAr), 21.2 (=*C*CH$_2$), 26.7 (NCH$_2$*C*H$_2$), 48.9 (N*C*H$_2$), 67.7 (*C*H), 120.3 (3-Ar*C*), 126.2 (6-Ar*C*H), 129.5 (3,5-NAr*C*H), 129.6 (3,5-NAr*C*H), 130.2 (1-NAr*C*), 134.7 (2,6-NAr*C*), 135.9 (2,6-NAr*C*), 141.7 (4-Ar*C*H), 142.7 (4-NAr*C*), 148.8 (5-Ar*C*H), 152.5 (N*C*(3)N), 153.4 (2-Ar*C*), 163.8 (N*C*(7a)N); **HRMS** (ESI$^+$) $C_{20}H_{22}BrN_4O$ $[M-BF_4]^+$ found 413.0963, requires 413.0972 (–2.0 ppm).

3-((3-fluoropyridin-2-yl)(hydroxy)methyl)-2-mesityl-6,7-dihydro-5H-pyrrolo[2,1-c][1,2,4]triazol-2-ium tetrafluoroborate 20

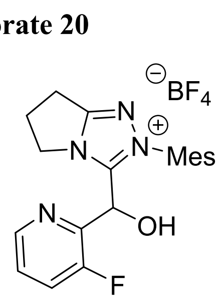

Following general procedure B, 3-fluoropicolinaldehyde (38 mg, 0.3 mmol) was added to a solution of NHC precatalyst (95 mg, 0.3 mmol) and Et_3N (84 µL, 0.6 mmol) in CH_2Cl_2 (10 mL). After stirring at rt for 30 mins, 4 drops aqueous 2 M HCl was added to quench the reaction

and then the mixture was concentrated under reduced pressure. The crude product was purified by column chromatography (50:1 chloroform:methanol) to yield the title compound as a yellow oil (92 mg, 70%). **IR** ν_{max} (film): 3435, 2926, 1591, 1452, 1047, 810; **^{1}H NMR** (500 MHz, CD_3OD) δ_H: 1.49 (3H, s, 2,6-C*H₃*NAr), 2.21 (3H, s, 2,6-CH_3NAr), 2.34 (3H, s, 4-CH_3NAr), 2.93-3.00 (2H, m, NCH_2CH_2), 3.31-3.34 (2.30H, m, =CCH_2 + CH_3OH), 4.74-4.83 (3.98H, m, NCH_2 + H_2O), 6.18 (1H, s, C*H*), 6.90 (1H, d, *J* 2.0, 3,5-NAr*H*), 7.16 (1H, d, *J* 2.0, 3,5-NAr*H*), 7.52 (1H, dt, *J* 8.6, 4.4, 6-Ar*H*), 7.61 (1H, ddd, *J* 9.8, 8.5, 1.3, 4-Ar*H*), 8.41 (1H, dt, *J* 4.6, 1.4, 5-Ar*H*); **^{13}C{^{1}H} NMR** (126 MHz, CD_3OD) δ_C: 15.5 (2,6-*C*H_3NAr), 15.9 (2,6-*C*H_3NAr), 19.8 (4-*C*H_3NAr), 21.2 (=C*C*H_2), 26.5 (NCH_2*C*H_2), 48.9 (N*C*H_2), 63.4 (*C*H), 124.4 (d, 4-Ar*C*H), 127.1 (6-Ar*C*H), 129.4 (3,5-NAr*C*H), 129.5 (3,5-NAr*C*H), 130.2 (1-NAr*C*), 134.9 (2,6-NAr*C*), 135.8 (2,6-NAr*C*), 142.6 (4-NAr*C*), 142.9 (d, 3-Ar*C*), 145.9 (d, 5-Ar*C*H), 151.8 (N*C*(3)N), 158.0 (2-Ar*C*), 156.0 (2-Ar*C*), 163.8 (N*C*(7a)N); **^{19}F NMR** (470 MHz, CD_3OD) δ_F: -127.3(s, ArF), -154.4 (s, BF_4); **HRMS** (ESI$^+$) $C_{20}H_{22}FN_4O$ [M-BF_4]$^+$ found 353.1760, requires 353.1772 (–3.4 ppm).

3-(Hydroxy(3-methylpyridin-2-yl)methyl)-2-mesityl-6,7-dihydro-5H-pyrrolo[2,1-c][1,2,4]triazol-2-ium tetrafluoroborate 21

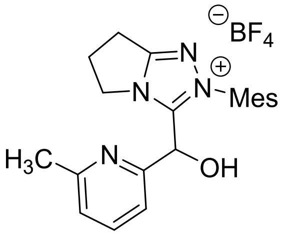

Following general procedure B, 6-methylpicolinaldehyde (36 mg, 0.3 mmol) was added to a solution of NHC precatalyst (95 mg, 0.3 mmol) and Et_3N (84 µL, 0.6 mmol) in CH_2Cl_2 (10 mL). After stirring at rt for 30 mins, 4 drops aqueous 2 M HCl was added to quench the reaction and then the mixture was concentrated under reduced pressure. The crude product was purified by column chromatography (50:1 chloroform:methanol) to yield the title compound as a colourless oil (89 mg, 68%). **IR** ν_{max} (film): 3447, 2926, 1591, 1460, 1283, 1049, 854; **^{1}H NMR** (400 MHz, CD_3OD) δ_H: 1.81 (3H, s, 2,6-CH_3NAr), 2.11 (3H, s, 2,6-CH_3NAr), 2.37 (3H, s, 4-CH_3NAr), 2.46 (3H, s, 6-CH_3Ar), 2.83-2.98 (2H, m, NCH_2CH_2), 3.26-3.30 (2H, m, =CCH_2), 4.41-4.48 (1H, m, NC$H_A$$H_B$), 4.60-4.67 (1H, m, NC$H_A$$H_B$), 5.87 (1H, s, C*H*), 7.00 (1H, s, 3,5-

NAr*H*), 7.13 (1H, s, 3,5-NAr*H*), 7.24-7.26 (2H, m, 3-Ar*H* + 5-Ar*H*), 7.70 (1H, d, *J* 7.8, 4-Ar*H*); **^{13}C{^{1}H} NMR** (101 MHz, CD_3OD) δ_C: 15.8 (2,6-*C*H_3NAr), 16.1 (2,6-*C*H_3NAr), 19.8 (4-*C*H_3NAr), 21.2 (=C*C*H_2), 22.7 (6-*C*H_3Ar), 26.5 (NCH_2*C*H_2), 48.1 (N*C*H_2), 68.1 (*C*H), 118.8 (5-Ar*C*H), 123.8 (3-Ar*C*H), 129.2 (3,5-NAr*C*H), 129.3 (3,5-NAr*C*H), 130.8 (1-NAr*C*), 135.0 (2,6-NAr*C*), 136.2 (2,6-NAr*C*), 137.9 (4-Ar*C*H), 142.3 (4-NAr*C*), 152.4 (N*C*(3)N), 154.8 (2-Ar*C*), 158.9 (6-Ar*C*), 163.4 (N*C*(7a)N); **HRMS** (ESI$^+$) $C_{21}H_{25}N_4O$ [M-BF_4]$^+$ found 349.2011, requires 349.2023 (–3.4 ppm).

3-(Furan-2-yl(hydroxy)methyl)-2-mesityl-6,7-dihydro-5H-pyrrolo[2,1-c][1,2,4]triazol-2-ium tetrafluoroborate 22

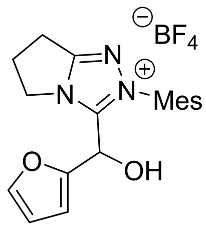

Following general procedure B, furan-2-carbaldehyde (50 μL, 0.6mmol) was added to a solution of NHC precatalyst (189 mg, 0.6 mmol) and Et_3N (168 μL, 1.2 mmol) in CH_2Cl_2 (10 mL). After stirring at rt for 30 mins, 4 drops aqueous 2 M HCl was added to quench the reaction and then the mixture was concentrated under reduced pressure. The crude product was purified by column chromatography (50:1 chloroform:methanol) to yield the title compound as a pale yellow oil (105 mg, 43%). **IR** ν_{max} (film): 3441, 1740, 1591, 1499, 1385, 1283, 1047, 930, 754; **^{1}H NMR** (400 MHz, CD_3OD) δ_H: 1.66 (3H, s, 2,6-C*H_3*NAr), 2.15 (3H, s, 2,6-C*H_3*NAr), 2.39 (3H, s, 4-C*H_3*NAr), 2.91-2.99 (2H, m, NCH_2C*H_2*), 3.27-3.31 (2H, m, =CC*H_2*), 4.65-4.76 (2H, m, NC*H_2*), 5.88 (1H, s, C*H*), 6.36 (1H, dd, *J* 3.3, 0.9, OCHC*H*), 6.43 (1H, dd, *J* 3.4, 1.9, OCC*H*), 7.03-7.04 (H, m, Ar*H*), 7.17-7.18 (H, m, Ar*H*), 7.58 (1H, dd, *J* 1.9, 0.8, OC*H*); **^{13}C{^{1}H} NMR** (101 MHz, CD_3OD) δ_C: 15.4 (2,6-*C*H_3NAr), 15.7 (2,6-*C*H_3NAr), 19.8 (4-*C*H_3NAr), 21.2 (=C*C*H_2), 26.4 (NCH_2*C*H_2), 48.7 (N*C*H_2), 61.1 (*C*H), 110.5 (OCH*C*H), 110.7 (OC*C*H), 129.3 (NAr*C*H), 129.4 (NAr*C*H), 130.5 (1-NAr*C*), 134.9 (2,6-NAr*C*), 136.0 (2,6-NAr*C*), 142.4 (4-NAr*C*), 144.6 (O*C*HCH), 148.0 (O*C*CH) 150.6 (N*C*(3)N), 163.8 (N*C*(7a)N); **HRMS** (ESI$^+$) $C_{19}H_{22}N_3O_2$ [M-BF_4]$^+$ found 324.1696, requires 324.1707 (–3.3 ppm).

3-(Hydroxy(pyridin-2-yl)methyl)-2-(2,4,6-trichlorophenyl)-6,7-dihydro-5H-pyrrolo[2,1-

c][1,2,4]triazol-2-ium tetrafluoroborate 23

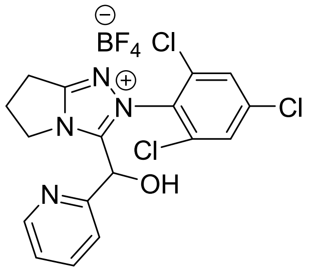

Following general procedure B, picolinaldehyde (57 µL, 0.6mmol) was added to a solution of NHC precatalyst (225 mg, 0.6 mmol) and Et_3N (168 µL, 1.2 mmol) in CH_2Cl_2 (10 mL). After stirring at rt for 30 mins, 4 drops aqueous 2 M HCl was added to quench the reaction and then the mixture was concentrated under reduced pressure. The crude product was purified by column chromatography (50:1 chloroform:methanol) to yield the title compound as a pale yellow oil (194 mg, 67%). **IR** ν_{max} (film): 3076, 2359, 1732, 1574, 1383, 1047, 824; **^{1}H NMR** (400 MHz, CD_3OD) δ_H: 2.91-2.99 (2H, m, NCH_2CH_2), 3.31-3.35 (2H, =CCH_2), 4.67-4.79 (2H,m, NCH_2), 6.27 (1H, s, C*H*), 7.40 (1H, ddd, *J* 7.6, 4.8, 1.1, 4-Ar*H*), 7.53 (1H, dt, *J* 7.9, 1.1, 3-Ar*H*), 7.76 (1H, d, *J* 2.2, NAr*H*), 7.84-7.89 (2H, m, 6-Ar*H* + NAr*H*), 8.50 (1H, ddd, *J* 4.8, 1.7, 0.9, 5-Ar*H*); **^{13}C{^{1}H} NMR** (101 MHz, CD_3OD) δ_C: 21.3 (=CCH_2), 26.5 (NCH_2CH_2), 49.0 (NCH_2), 69.0 (*C*H), 121.7 (3-Ar*C*H), 124.4 (4-Ar*C*H), 129.1 (NAr*C*H), 129.3 (NAr*C*), 129.3 (NAr*C*H), 133.9 (NAr*C*), 135.2 (NAr*C*), 137.9 (6-Ar*C*H), 139.1 (NAr*C*), 149.5 (5-Ar*C*H), 154.1 (N*C*(3)N), 155.3 (Ar*C*), 164.0 (N*C*(7a)N); **HRMS** (ESI$^+$) $C_{17}H_{14}Cl_3N_4O$ [M-BF_4]$^+$ found 395.0221, requires 395.0228 (–2.8 ppm).

3-(Hydroxy(pyridin-2-yl)methyl)-2-phenyl-5,6,7,8-tetrahydro-[1,2,4]triazolo[4,3-a] pyridine-2-ium tetrafluoroborate 24

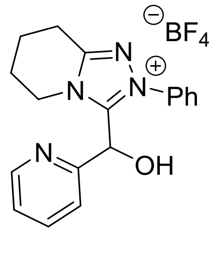

Following general procedure B, picolinaldehyde (57 µL, 0.6 mmol) was added to a solution of NHC precatalyst (172 mg, 0.6 mmol) and Et_3N (168 µL, 1.2 mmol) in CH_2Cl_2 (10 mL). After stirring at rt for 30 mins, 4 drops aqueous 2 M HCl was added to quench the reaction and then

the mixture was concentrated under reduced pressure. The crude product was purified by column chromatography (50:1 chloroform:methanol) to yield the title compound as a pale yellow oil (91 mg, 39%). **IR** ν_{max} (film): 3441, 1572, 1441, 1317, 1057, 874, 766; **^{1}H NMR** (400 MHz, CD_3OD) δ_H: 1.99-2.18 (4H, m, 2 × C*H₂*), 3.15 (2H, ddd, *J* 7.3, 5.9, 1.6, =CC*H₂*), 3.83 (1H, ddd, *J* 13.1, 7.1, 4.2, NC$H_A$$H_B$), 4.52 (1H, dtd, *J* 13.2, 5.0, 2.0, NC$H_A$$H_B$), 6.39 (1H, s, C*H*), 7.41-7.45 (1H, m, 5-Ar*H*), 7.59-7.71 (5H, m, NAr*H*), 7.77-7.79 (1H, m, 6-Ar*H*), 7.95(1H, td, *J* 7.8, 1.8, 3-Ar*H*), 8.53-8.55 (1H, m, 4-Ar*H*); **^{13}C{^{1}H} NMR** (101 MHz, CD_3OD) δ_C: 17.9 (*C*H₂), 20.6 (*C*H₂), 21.0 (=C*C*H₂), 45.7 (N*C*H₂), 66.9 (*C*H), 121.3 (3-Ar*C*H), 124.0 (4-Ar*C*H), 125.7 (2 × NAr*C*H), 129.6 (2 × NAr*C*H), 131.3 (NAr*C*H), 135.0 (NAr*C*), 137.9 (5-Ar*C*H), 149.1 (6-Ar*C*H), 152.0 (N*C*(3)N), 153.4 (N*C*(7a)N), 156.0 (Ar*C*); **HRMS** (ESI$^+$) $C_{18}H_{19}N_4O$ $[M-BF_4]^+$ found 307.1547, requires 307.1553 (–2.2 ppm).

3-(Hydroxy(pyridin-2-yl)methyl)-2-phenyl-6,7,8,9-tetrahydro-5H-[1,2,4]triazolo[4,3-a]azepin-2-ium tetrafluoroborate 25

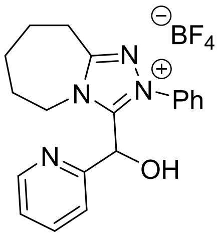

Following general procedure B, picolinaldehyde (29 µL, 0.3 mmol) was added to a solution of NHC precatalyst (91 mg, 0.3 mmol) and Et_3N (84 µL, 0.6 mmol) in CH_2Cl_2 (10 mL). After stirring at rt for 30 mins, 4 drops aqueous 2 M HCl was added to quench the reaction and then the mixture was concentrated under reduced pressure. The crude product was purified by column chromatography (50:1 chloroform:methanol) to yield the title compound as a pale yellow oil (33 mg, 27%). **IR** ν_{max} (film): 3447, 2934, 1578, 1441, 1209, 1056, 889, 737; **^{1}H NMR** (400 MHz, CD_3OD) δ_H: 0.74-0.77 (1H, m, C$H_A$$H_B$), 0.96-1.14 (5H, m, C$H_A$$H_B$ + 2 × C*H₂*), 2.40-2.42 (2H, m, =CC*H₂*), 3.41-3.61 (2H, m, NC*H₂*), 5.56 (1H, s, C*H*), 6.63-6.66 (1H, m, 5-Ar*H*), 6.82-6.96 (5H, m, NAr*H*), 7.02-7.04 (1H, m, 6-Ar*H*), 7.16-7.20 (1H, m, 3-Ar*H*), 7.70-7.76 (1H, m, 4-Ar*H*); **^{13}C{^{1}H} NMR** (101 MHz, CD_3OD) δ_C: 23.3 (*C*H₂), 24.4 (=C*C*H₂), 25.4 (*C*H₂), 28.4 (NCH₂*C*H₂), 46.8 (N*C*H₂), 66.1 (*C*H), 120.2 (6-Ar*C*H), 123.2 (5-Ar*C*H), 124.9 (2 × 3,5-NAr*C*H), 128.9 (2 × 2,6-NAr*C*H), 130.7 (4-NAr*C*H), 134.0 (NAr*C*), 137.3 (3-Ar*C*H),

148.3 (4-Ar*C*H), 152.4 (N*C*(3)N), 155.8 (Ar*C*), 158.3 (N*C*(7a)N); **HRMS** (ESI^+) $C_{19}H_{21}N_4O$ $[M-BF_4]^+$ found 321.1700, requires 327.1710 (–3.1 ppm).

3-(Hydroxy(3-methylpyridin-2-yl)methyl)-2-phenyl-5,6,7,8-tetrahydro-[1,2,4]triazolo[4,3-a] pyridine-2-ium tetrafluoroborate 26

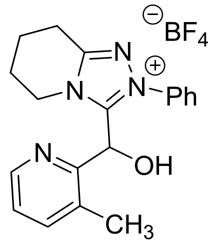

Following general procedure B, 3-methylpicolinaldehyde (67 μL, 0.6 mmol) was added to a solution of NHC precatalyst (172 mg, 0.6 mmol) and Et_3N (168 μL, 1.2 mmol) in CH_2Cl_2 (10 mL). After stirring at rt for 30 mins, 4 drops aqueous 2 M HCl was added to quench the reaction and then the mixture was concentrated under reduced pressure. The crude product was purified by column chromatography (50:1 chloroform:methanol) to yield the title compound as a pale yellow oil (110 mg, 45%). **IR** ν_{max} (film): 3447, 2959, 1574, 1454, 1315, 1047, 770; **^{1}H NMR** (400 MHz, CD_3OD) δ_H: 2.08-2.25 (4H, m, 2 × C*H₂*), 2.32 (3H, s, C*H₃*), 3.14-3.25 (2H, m, =CC*H₂*), 4.43-4.58 (2H, m, NC*H₂*), 6.44 (1H, s, C*H*), 7.31.-7.34 (2H, m, 3,5-NAr*H*), 7.40 (1H, dd, *J* 7.7, 4.8, 6-Ar*H*), 7.54-7.59 (2H, m, 2, 6-NAr*H*), 7.63-7.68 (1H, m, 4-NAr*H*), 7.75 (1H, ddd, *J* 7.7, 1.7, 0.8, 4-Ar*H*), 8.38 (1H, ddd, *J* 4.9, 1.7, 0.7, 5-Ar*H*); **^{13}C{^{1}H} NMR** (101 MHz, CD_3OD) δ_C: 16.2 (*C*H₃), 18.0 (=CCH₂*C*H₂), 20.9 (NCH₂*C*H₂), 21.2 (=C*C*H₂), 46.8 (N*C*H₂), 68.5 (*C*H), 124.7 (6-Ar*C*H), 125.2 (3,5-NAr*C*H), 129.7 (2,6- NAr*C*H), 131.4 (4-NAr*C*H), 133.2 (2-Ar*C*), 134.9 (1-NAr*C*), 139.8 (4-Ar*C*H), 146.5 (5-Ar*C*H), 152.7 (N*C*(3)N), 153.4 (N*C*(7a)N), 153.6 (3-Ar*C*); **HRMS** (ESI^+) $C_{18}H_{19}N_4O$ $[M-BF_4]^+$ found 321.1698, requires 321.1770 (–3.7 ppm).

3-(Hydroxy(3-methylpyridin-2-yl)methyl)-2-phenyl-6,7,8,9-tetrahydro-5H-[1,2,4]triazolo[4,3-a]azepin-2-ium tetrafluoroborate 27

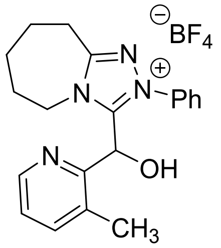

Following general procedure B, 3-methylpicolinaldehyde (57 μL, 0.6 mmol) was added to a solution of NHC precatalyst (181 mg, 0.6 mmol) and Et_3N (168 μL, 1.2 mmol) in CH_2Cl_2 (10 mL). After stirring at rt for 30 mins, 4 drops aqueous 2 M HCl was added to quench the reaction and then the mixture was concentrated under reduced pressure. The crude product was purified by column chromatography (50:1 chloroform:methanol) to yield the title compound as a pale yellow oil (89 mg, 35%). **IR** ν_{max} (film): 3431, 2934, 1578, 1456, 1285, 1055, 889, 768; **^{1}H NMR** (400 MHz, CD_3OD) δ_H: 1.88-2.11 (6H, m, 3 × C*H₂*), 2.34 (3H, S, C*H₃*), 3.21-3.29 (2H, m, =CC*H₂*), 4.55-4.74 (2H, m, NC*H₂*), 6.42 (1H, s, C*H*), 7.33-7.36 (2H, m, 3,5-NAr*H*), 7.41 (1H, dd, *J* 7.7, 4.8, 6-Ar*H*), 7.56-7.61 (2H, m, 2,6-NAr*H*), 7.65-7.70 (1H, m, 4-NAr*H*), 7.77 (1H, ddd, *J* 7.7, 1.6, 0.8, 4-Ar*H*), 8.40 (1H, ddd, *J* 4.8, 1.7, 0.7, 5-Ar*H*); **^{13}C{^{1}H} NMR** (101 MHz, CD_3OD) δ_C: 16.3 (*C*H₃), 24.3 (=CCH₂*C*H₂), 25.4 (=C*C*H₂), 26.5 (NCH₂CH₂*C*H₂), 29.4 (NCH₂*C*H₂), 48.7 (N*C*H₂), 68.6 (*C*H), 124.8 (6-Ar*C*H), 125.2 (3,5-NAr*C*H), 129.8 (2,6-NAr*C*H), 131.5 (4-NAr*C*H), 133.4 (2-Ar*C*), 134.7 (1-NAr*C*), 139.9 (4-Ar*C*H), 146.3 (5-Ar*C*H), 153.5 (N*C*(3)N), 153.6 (3-Ar*C*), 159.3 (N*C*(7a)N); **HRMS** (ESI^+) $C_{19}H_{21}N_4O$ $[M-BF_4]^+$ found 335.1857, requires 335.1866 (–2.7 ppm).

3-(Hydroxy(3-methylpyridin-2-yl)methyl)-2-mesityl-5,6,7,8-tetrahydro-[1,2,4]triazolo[4,3-a]pyridin-2-ium tetrafluoroborate 28

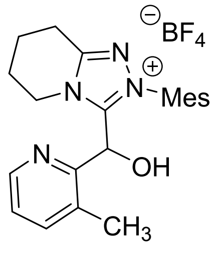

Following general procedure B, 3-methylpicolinaldehyde (34 μL, 0.3 mmol) was added to a solution of NHC precatalyst (99 mg, 0.3 mmol) and Et_3N (84 μL, 0.6 mmol) in CH_2Cl_2 (10 mL). After stirring at rt for 30 mins, 4 drops aqueous 2 M HCl was added to quench the reaction

and then the mixture was concentrated under reduced pressure. The crude product was purified by column chromatography (50:1 chloroform:methanol) to yield the title compound as a colourless oil (77 mg, 57%). **IR** ν_{max} (film): 3428, 2961, 1570, 1456, 1287, 1053, 797; **^{1}H NMR** (400 MHz, CD_3OD) δ_H: 1.40 (3H, s, 2,6-C*H*$_3$NAr), 2.07 (3H, s, 3-C*H*$_3$Ar), 2.12-2.30 (7H, m, 2,6-C*H*$_3$NAr + NCH$_2$C*H*$_2$ +N CH$_2$CH$_2$C*H*$_2$), 2.37 (3H, s, 4-C*H*$_3$NAr), 3.16-3.26 (2H, m, =CC*H*$_2$), 4.60-4.66 (1H, m, NC*H*$_A$H$_B$), 4.78-4.84 (1H, m, NCH$_A$*H*$_B$), 6.10 (1H, s, C*H*), 6.96 (1H, s, 3,5-NAr*H*), 7.21 (1H, s, 3,5-NAr*H*), 7.35 (1H, dd, *J* 7.7, 4.8, 6-Ar*H*), 7.67 (1H, ddd, *J* 7.7, 1.7, 0.8, 4-Ar*H*), 8.38 (1H, dd, *J* 4.8, 1.7, 5-Ar*H*); **^{13}C{^{1}H} NMR** (101 MHz, CD_3OD) δ_C: 15.3 (2,6-*C*H$_3$NAr), 15.9 (2,6-*C*H$_3$NAr + 3-*C*H$_3$Ar), 18.0 (NCH$_2$CH$_2$*C*H$_2$), 19.8 (4-*C*H$_3$NAr), 20.9 (NCH$_2$*C*H$_2$), 21.2 (=C*C*H$_2$), 47.2 (N*C*H$_2$), 67.4 (*C*H), 124.8 (6-Ar*C*H), 129.5 (3,5-NAr*C*H), 129.5 (3,5-NAr*C*H), 130.0 (1-NAr*C*), 132.9 (2-Ar*C*), 134.8 (2,6-NAr*C*), 136.1 (2,6-NAr*C*), 139.5 (4-Ar*C*H), 142.5 (4-NAr*C*), 146.9 (5-Ar*C*H), 153.3 (3-Ar*C*), 153.8 (N*C*(3)N), 154.4 (N*C*(7a)N); **HRMS** (ESI$^+$) $C_{22}H_{27}N_4O$ [M-BF$_4$]$^+$ found 363.2165, requires 363.2179 (–3.9 ppm).

3-(Hydroxy(3-methylpyridin-2-yl)methyl)-2-phenyl-5,6-dihydro-8H-[1,2,4]triazolo[3,4-c][1,4]oxazin-2-ium tetrafluoroborate 29

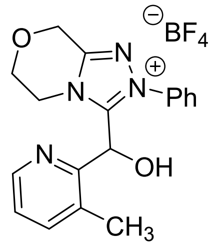

Following general procedure B, 3-methylpicolinaldehyde (34 μL, 0.3 mmol) was added to a solution of NHC precatalyst (87 mg, 0.3 mmol) and Et_3N (84 μL, 0.6 mmol) in CH_2Cl_2 (10 mL). After stirring at rt for 30 mins, 4 drops aqueous 2 M HCl was added to quench the reaction and then the mixture was concentrated under reduced pressure. The crude product was purified by column chromatography (50:1 chloroform:methanol) to yield the title compound as a pale yellow oil (60 mg, 49%). After crystallization from methanol, crystal triazolium adduct was collected, **mp** 160-162 °C. **IR** ν_{max} (film): 3399, 1578, 1447, 1327, 1065, 887; **^{1}H NMR** (400 MHz, CD_3OD) δ_H: 2.31 (3H, s, C*H*$_3$), 4.23-4.33 (2H, m, NCH$_2$C*H*$_2$), 4.63 (1H, ddd, *J* 13.7, 5.7, 4.4, NC*H*$_A$H$_B$), 4.72 (1H, ddd, *J* 13.8, 6.5, 4.6, NCH$_A$*H*$_B$), 5.21 (2H, s, =CC*H*$_2$), 6.48 (1H, s,

C*H*), 7.30.-7.33 (2H, m, 3,5-NAr*H*), 7.40 (1H, dd, *J* 7.8, 4.8, 6-Ar*H*), 7.56-7.60 (2H, m, 2, 6-NAr*H*), 7.66-7.70 (1H, m, 4-NAr*H*), 7.74 (1H, ddd, *J* 7.8, 1.7, 0.8, 4-Ar*H*), 8.39 (1H, ddd, *J* 4.8, 1.7, 0.7, 5-Ar*H*); **^{13}C{^{1}H} NMR** (101 MHz, CD_3OD) δ_C: 16.1 (*C*H_3), 46.2 (N*C*H_2), 61.7 (=C*C*H_2), 62.6 (NCH_2*C*H_2), 68.6 (*C*H), 124.8 (6-Ar*C*H), 125.1 (3,5-NAr*C*H), 129.9 (2,6-NAr*C*H), 131.7 (4-NAr*C*H), 133.0 (2-Ar*C*), 134.6 (1-NAr*C*), 139.8 (4-Ar*C*H), 146.7 (5-Ar*C*H), 150.1 (N*C*(7a)N), 153.6 (N*C*(3)N), 153.6 (3-Ar*C*); **HRMS** (ESI^+) $C_{18}H_{19}N_4O_2$ [M-BF_4]$^+$ found 323.1491, requires 323.1503 (–3.6 ppm).

3-(Hydroxy(3-methylpyridin-2-yl)methyl)-2-mesityl-5,6-dihydro-8H-[1,2,4]triazolo[3,4-c][1,4]oxazin-2-ium tetrafluoroborate 30

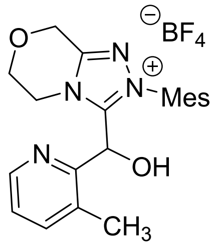

Following general procedure B, 3-methylpicolinaldehyde (34 µL, 0.3 mmol) was added to a solution of NHC precatalyst (99 mg, 0.3 mmol) and Et_3N (84 µL, 0.6 mmol) in CH_2Cl_2 (10 mL). After stirring at rt for 30 mins, 4 drops aqueous 2 M HCl was added to quench the reaction and then the mixture was concentrated under reduced pressure. The crude product was purified by column chromatography (50:1 chloroform:methanol) to yield the title compound as a colourless oil (88 mg, 65%). After crystallization from methanol, crystal triazolium adduct was collected, **mp** 171-173 °C. **IR** ν_{max} (film): 3422, 2945, 1580, 1456, 1300, 1057, 864, 795; **^{1}H NMR** (400 MHz, CD_3OD) δ_H: 1.37 (3H, s, 2,6-C*H_3*NAr), 2.05 (3H, s, 3-C*H_3*Ar), 2.23 (3H, s, 2,6-C*H_3*NAr), 2.38 (3H, s, 4-C*H_3*NAr), 4.25-4.42 (2H, m, NCH_2C*H_2*), 4.76-4.82 (1H, m, NC*H_A*H_B), 4.86-4.97 (7.85H, m, NCH_A*H_B* + H_2O), 5.22 (2H, m, =CC*H_2*), 6.14 (1H, s, C*H*), 6.98 (1H, s, 3,5-NAr*H*), 7.23 (1H, s, 3,5-NAr*H*), 7.36 (1H, dd, *J* 7.8, 4.7, 6-Ar*H*), 7.67 (1H, d, *J* 7.7, 4-Ar*H*), 8.39 (1H, d, *J* 5.1, 5-Ar*H*); **^{13}C{^{1}H} NMR** (101 MHz, CD_3OD) δ_C: 15.2 (2,6-*C*H_3NAr), 15.8 (2,6-*C*H_3NAr), 15.9 (3-*C*H_3Ar), 19.8 (4-*C*H_3NAr), 46.4 (N*C*H_2), 61.7 (=C*C*H_2), 62.6 (NCH_2*C*H_2), 67.4 (*C*H), 124.9 (6-Ar*C*H), 129.6 (3,5-NAr*C*H), 129.7 (1-NAr*C*), 132.8 (2-Ar*C*), 134.8 (2,6-NAr*C*), 136.1 (2,6-NAr*C*), 139.5 (4-Ar*C*H), 142.8 (4-NAr*C*), 147.1 (5-Ar*C*H), 151.0 (N*C*(7a)N), 153.2 (3-Ar*C*), 154.5 (N*C*(3)N); **HRMS** (ESI^+) $C_{21}H_{25}N_4O_2$ [M-

$BF_4]^+$ found 365.1960, requires 365.1972 (–3.3 ppm).

Unsuccessful Isolation (Adduct 31, 32 and 33)

3-(Hydroxybenzyl)triazolium Adduct **31**, **32** and **33** were observed from ^{1}H NMR spectra of the crude reaction mixtures (Shown in Figure a, b and c), but the isolation was unsuccessful as result of unstable ability.

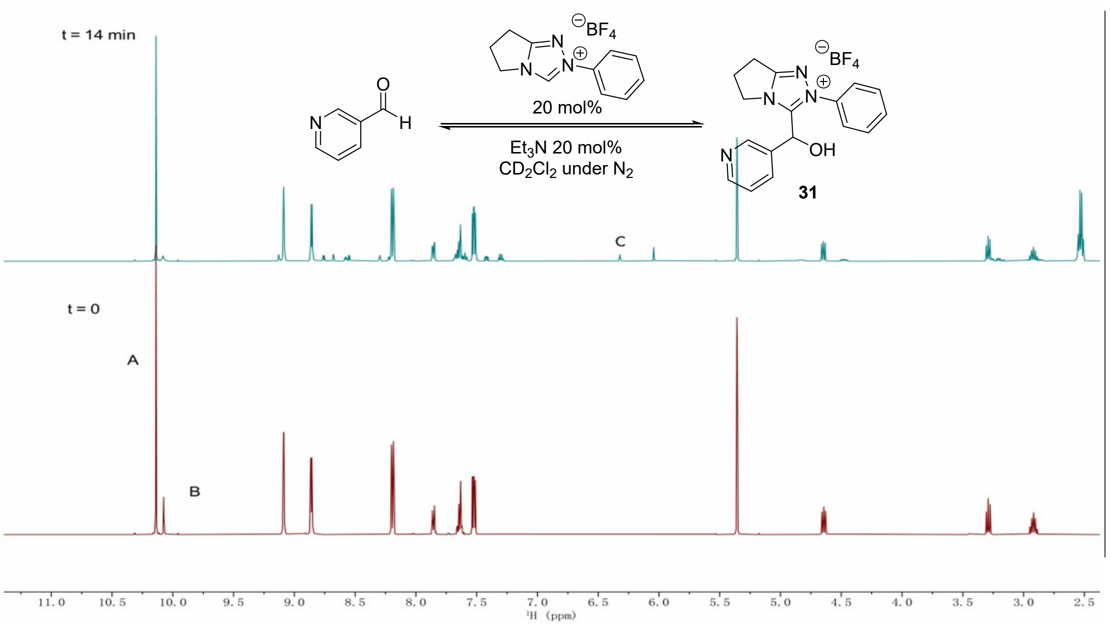

Figure a. ^{1}H NMR spectra (400 MHz) for reaction of nicotinaldehyde (0.1 M) with N-Ph NHC precursor **39** (0.02 M) and Et_3N (0.02 M) in CD_2Cl_2 at 25 °C. A = ArC*H*O, B = NHC precursor NC*H*N, C = Adduct C(α)*H*.

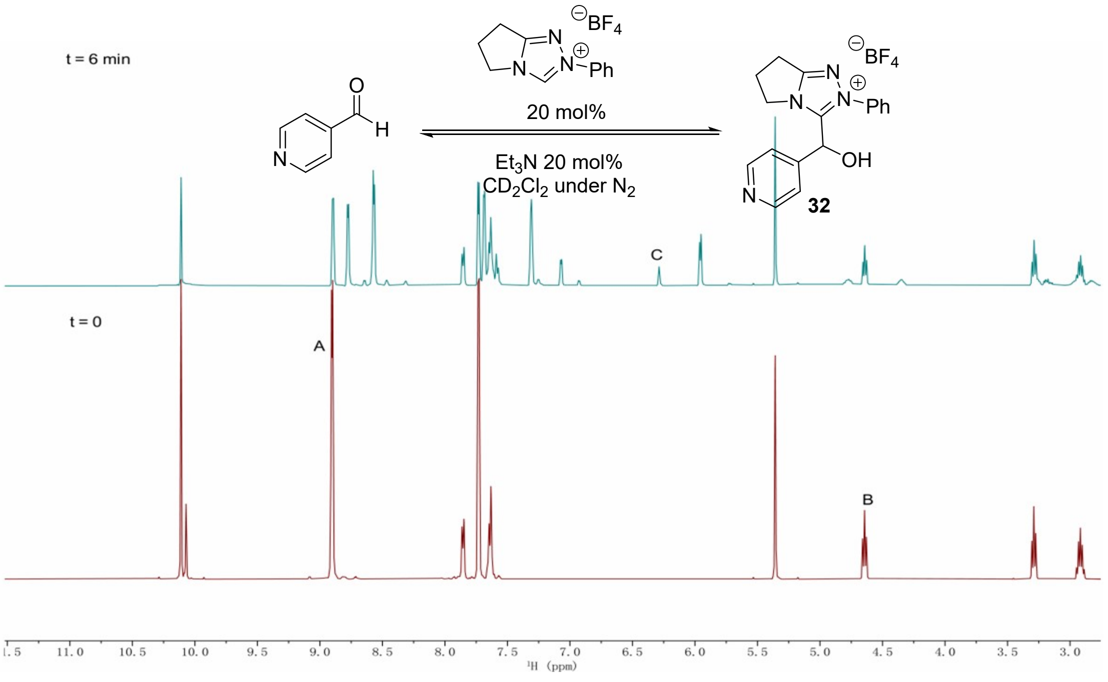

Figure b. ^{1}H NMR spectra (400 MHz) for reaction of isonicotinaldehyde (0.1 M) with N-Ph NHC precursor **39** (0.02 M) and Et_3N (0.02 M) in CD_2Cl_2 at 25 °C. A =Ar*H*CHO, B = NHC precursor C*H*$_2$, C = Adduct C(α)*H*.

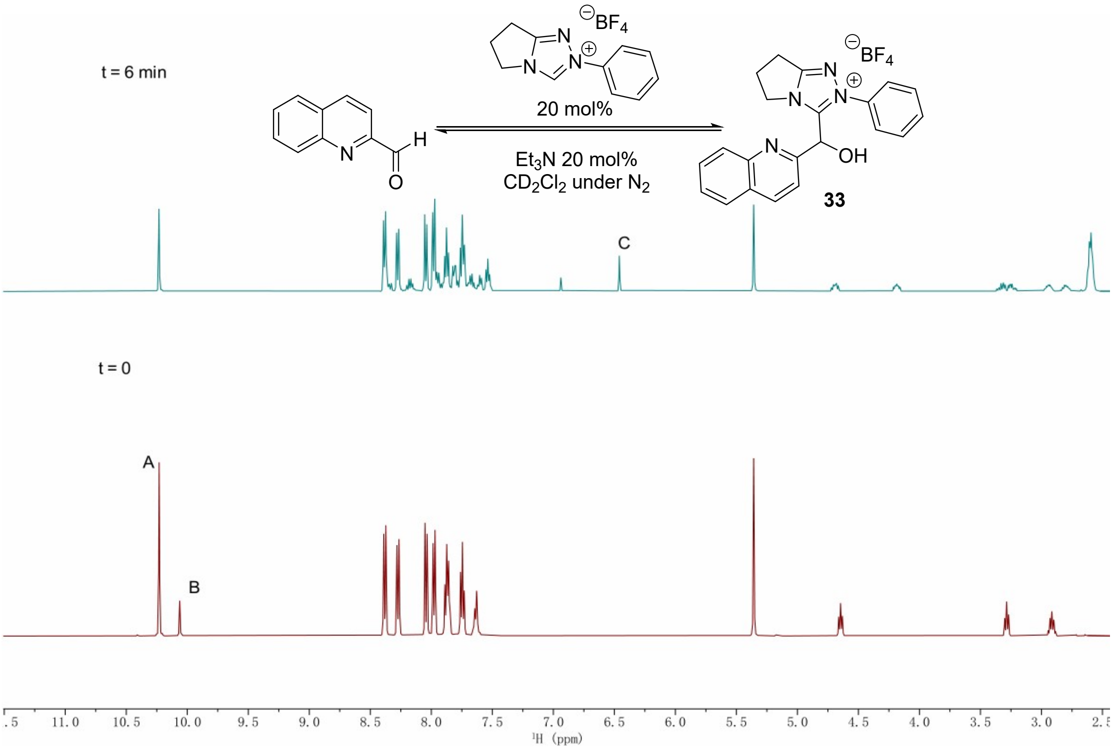

Figure c.^{1}H NMR spectra (400 MHz) for reaction of quinoline-2-carbaldehyde (0.1 M) with N-Ph NHC precursor **39** (0.02 M) and Et_3N (0.02 M) in CD_2Cl_2 at 25 °C. A =ArCHO, B = NHC precursor NC*H*N, C = Adduct C(α)*H*.

3. Determination of Rate and Equilibrium Constants for 3-(Hydroxybenzyl)triazolium Adduct Formation in CD_3OD

Based on Leeper and White's kinetic experiments of thiazolium ion-catalyzed benzoin condensation in 2001,[12] ^{1}H NMR spectroscopy was used to monitor the benzoin reaction. Stoichiometric quantities of aldehyde and triazolium precatalyst were added to a buffer solution of NEt_3 and Et_3N·HCl in CD_3OD and the reaction process was monitored by ^{1}H NMR spectroscopy at 25 °C. The concentrations of aldehyde, triazolium precatalyst and triazolium adduct at different times were obtained from ^{1}H NMR spectra, and then was used to calculate kinetic parameters of the reversible process. Due to methanol-d_4's strong deuteration ability, the concurrent H/D-exchange of the benzylic hydrogens of triazolium adduct also occurred, thus the calculation of the formation rate of Breslow intermediate was possible by using the decreasing concentration of H-adduct (III, Scheme 1). Additionally, the equilibrium of hemiacetal formation from the addition of solvent to the

aldehyde was also taken into consideration when the kinetic parameters were calculated.

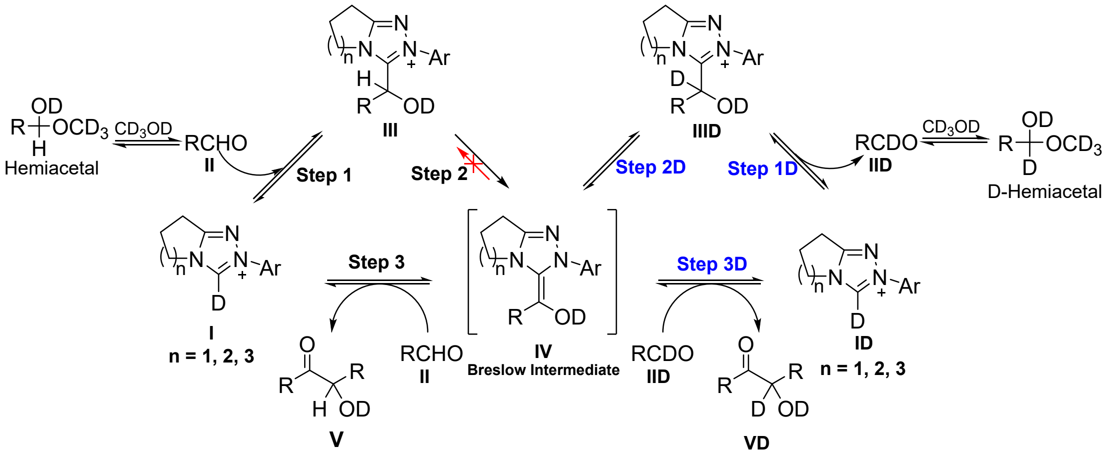

Scheme 1. Mechanistic model for the self-condensation of aldehyde catalyzed by a triazolium-derived NHC in buffered methanol-d$_4$ solution.

3.1 General Experimental Procedure

In a nitrogen protected NMR tube, the aldehyde (15 μmol) was added to a CD_3OD solution of the appropriate NHC precatalyst (650 μL, 0.023 M). The reaction was initiated by adding 100 μL CD_3OD solution of NEt_3 (0.450 M) and $Et_3N{\cdot}HCl$ (0.225 M). This gave an overall aldehyde and NHC concentration of 0.02 M and a total buffer concentration of 0.09 M. The reaction was monitored by temperature controlled (25°C) ^{1}H NMR spectroscopy on a Bruker Avance 500 MHz NMR spectrometer. Spectra were taken at 1 or 2 minutes intervals over 1.5 - 8 hours. Over the course of this reaction, it was possible to observe concentration changing of NHC, aldehyde, the corresponding 3-(hydroxybenzyl)triazolium adduct. It was also possible to observe formation of deuterated 3-(hydroxybenzyl)triazolium salts (D-adducts), via deuteration of the Breslow intermediate.

3.2 Aldehyde-Methanol Adduct Equilibrium

In methanol-d$_4$, the aldehydes were in equilibrium with hemiacetals (Scheme 2) under our reaction conditions, which can be observed.

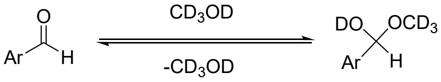

Scheme 2. Aldehyde-hemiacetal equilibrium

The equilibrium constant for this process, K_{hem}, is described by Equation 1. A value for K_{hem} was

determined for the reaction conditions using the integrals of the hemiacetal and the aldehyde. The fraction of aldehyde presented at equilibrium, f_{ald}, is described by Equation 2. Additionally, the rapidly reversible reaction remains across the whole experiment, and therefore, we can consider f_{ald} unchanged across the whole experiment. For example, the profile of f_{ald} at different times for the reaction between *N*-phenyl triazolium precatalyst **39** (0.02 M) and 3-methyl-2-pyridinecarboxaldehyde **35** (0.02 M) under a triethylamine buffer (0.06 M NEt_3 and 0.03 M $NEt_3 \cdot HCl$) in methanol-d_4 at 25 °C (shown in Figure S1). The value of f_{ald} was unchanged at about 0.21 across the whole process, and the average f_{ald} value obtained within this reaction was 0.211 (SD = 0.003).

$$K_{hem} = \frac{[Hemiacetal]}{[Aldehyde]} \quad \textbf{Equation 1}$$

$$f_{ald} = \frac{[Aldehyde]}{([Aldehyde] + [Hemiacetal])} \quad \textbf{Equation 2}$$

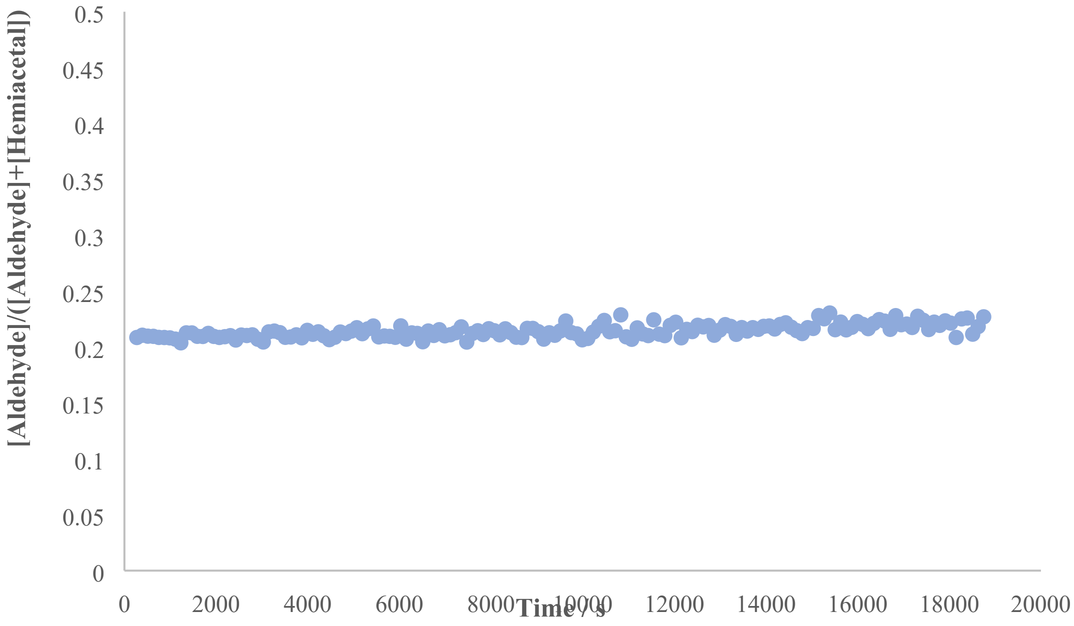

Figure S1. A f_{ald} profile for the reaction between *N*-phenyl triazolium precatalyst **39** (0.02 M) and 3-methyl-2-pyridinecarboxaldehyde **35** (0.02 M) under a triethylamine buffer (0.06 M NEt_3 and 0.03 M $NEt_3 \cdot HCl$) in methanol-d_4 at 25 °C.

3.3 Determination of Concentration

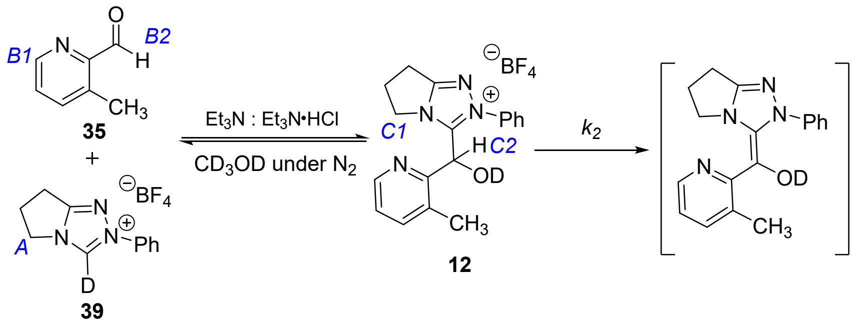

As an example, the reaction of aldehyde **35** and triazolium precatalyst **39** was monitored using ^{1}H NMR spectra, with representative NMR spectra over the course of the experiment given in Figure S2.

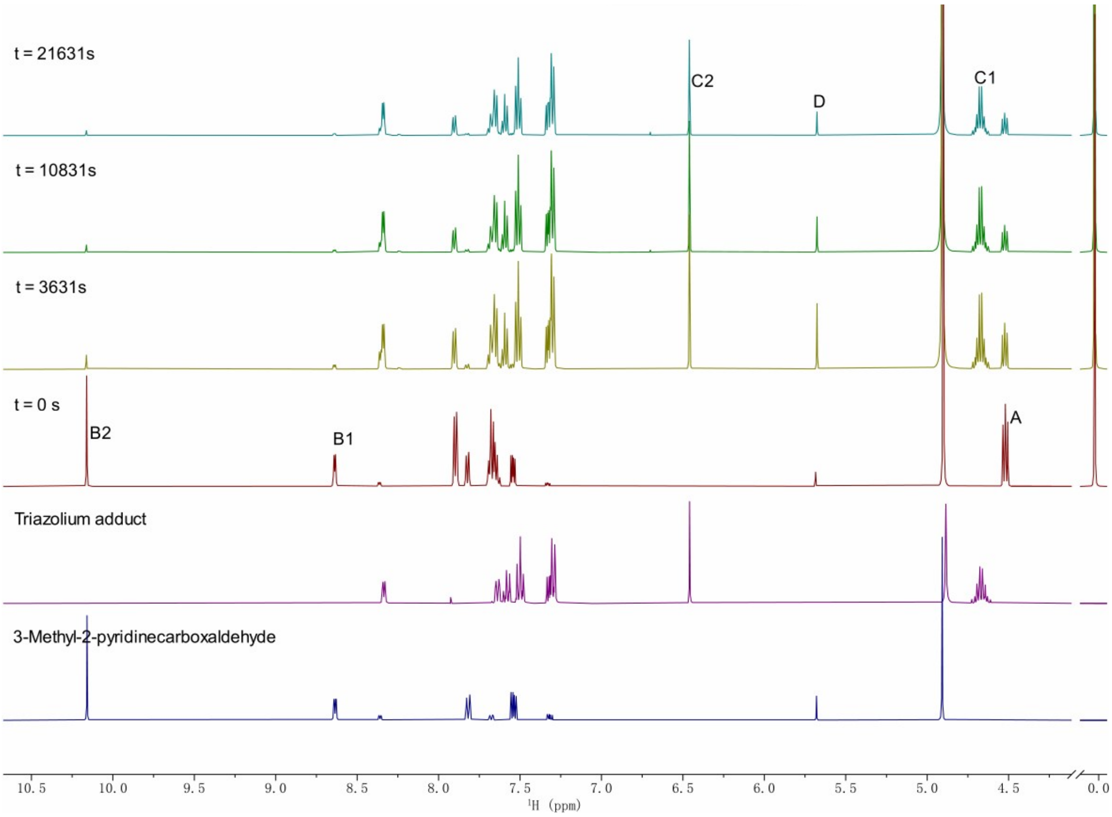

Figure S2. Representative ^{1}H NMR spectra (400 MHz) for reaction of 3-methyl-2-pyridinecarboxaldehyde **35** (0.02 M) with *N*-Ph NHC precursor **39** (0.02 M) under a triethylamine buffered (NEt$_3$:NEt$_3$·HCl, 2:1, 0.09 M) CD$_3$OD at 25 °C. A = NHC precursor NCH_2, B1 =ArHCHO, B2 =ArHCHO, C1 = Adduct NCH_2, C2 =Tetrahedral Adduct C(α)H, D = Hemiacetal CH.

Upon initiation of the reaction, the multiplet signal at 4.67 ppm (**C1**) appeared in the ^{1}H NMR spectrum. The ^{1}H NMR spectrum of the 3-(hydroxybenzyl)triazolium adduct **12** allows us to assign the signal to the CH_2 protons adjacent to the N(4) position. Additionally, according to spectra of other adducts we prepared, the catalyst CH_2 signal is sometimes split into two diastereotopic signals.

As the triazolium species were present in solution only as the free triazolium precatalyst **39** and the 3-(hydroxybenzyl)triazolium adduct **12** (or deuterated adduct **12D**), the sum of these integrals at any time during the reaction should equal to the concentration of precatalyst present initially (0.02 M). Thus, the integral for **C1** was used to calculate the concentration of total 3-(hydroxybenzyl)triazolium adduct **12** in solution, relative to the sum of **C1** and **A**, which corresponded to the total concentration of triazolium species in adduct and precatalyst form (Equation 3).

$$[Adduct\ (tot)] = \frac{A_{C1}/2}{(A_{C1} + A_A)/2} \times 0.02$$

Equation 3

The multiplet signal at 4.52 ppm (**A**), corresponding to the pair of CH_2 protons on the five-membered ring (adjacent to the *N*(4) atom), was used to calculate the concentration of precatalyst **39** (Equation 4). Signals corresponding to the other CH_2 peaks on the five-membered ring could not be used as these overlapped with signals corresponding to the triethylamine buffer.

$$[Catalyst] = \frac{A_A/2}{(A_{C1} + A_A)/2} \times 0.02$$

Equation 4

The singlet at 6.46 ppm (**C2**) corresponds to the exchangeable C(α)-*H* on the adduct **12**. Using this signal, the concentration of protonated adduct **12** was determined relative to the sum of the integrals of signals **C1** and **A** (Equation 5). Furthermore, the concentration of deuterated adduct **12D** was calculated from the difference in concentration between the total and protonated species (Equation 6).

$$[Adduct\ (H)] = \frac{A_{C2}}{(A_{C1} + A_A)/2} \times 0.02$$

Equation 5

$$[Adduct\ (D)] = [adduct\ (tot)] - [adduct(H)]$$

Equation 6

To create an accurate depiction of speciation over time, a correction must be made for f_{ald} in each aldehyde concentration. As most of aldehyde **35** was converted to hemiacetal in solution, the concentration of aldehyde was calculated from the doublet signals at 8.64 (**B1**) corresponding to the aryl C*H* proton, relative to the sum of the **C1** and **A** signals (Equation 7), and the total concentration of aldehyde containing aldehyde and hemiacetal was calculated using the equation 8 based on equation 2. Concentrations of protonated aldehyde **35** and hemiacetal were determined from the

singlet signals at 10.16 (**B2**) and 5.68 ppm (**D**) corresponding to the aldehydic hydrogen and the C*H* on hemiacetal. Equations 7 – 11 summarize how these concentrations were calculated.

$$[Aldehyde] = \frac{A_{B1}}{(A_{C1} + A_A)/2} \times 0.02$$ **Equation 7**

$$[Aldehyde\ (tot)] = \frac{1}{f_{ald}} \times \frac{A_{B1}}{(A_{C1} + A_A)/2} \times 0.02$$ **Equation 8**

$$[Aldehyde\ (H)] = \frac{A_{B2}}{(A_{C1} + A_A)/2} \times 0.02$$ **Equation 9**

$$[Aldehyde\ (D)] = [aldehyde] - [aldehyde(H)]$$ **Equation 10**

$$[Hemiacetal] = \frac{A_D}{(A_{C1} + A_A)/2} \times 0.02$$ **Equation 11**

Therefore, a concentration profile for the self-condensation of 3-methyl-2-pyridine-carboxaldehyde **35** (0.02 M) with *N*-Ph NHC precursor (0.02 M) under a triethylamine buffer was made, such as Figure S3. The equilibrium concentrations of precatalyst, aldehyde, adduct and hemiacetal were obtained from this concentration profile.

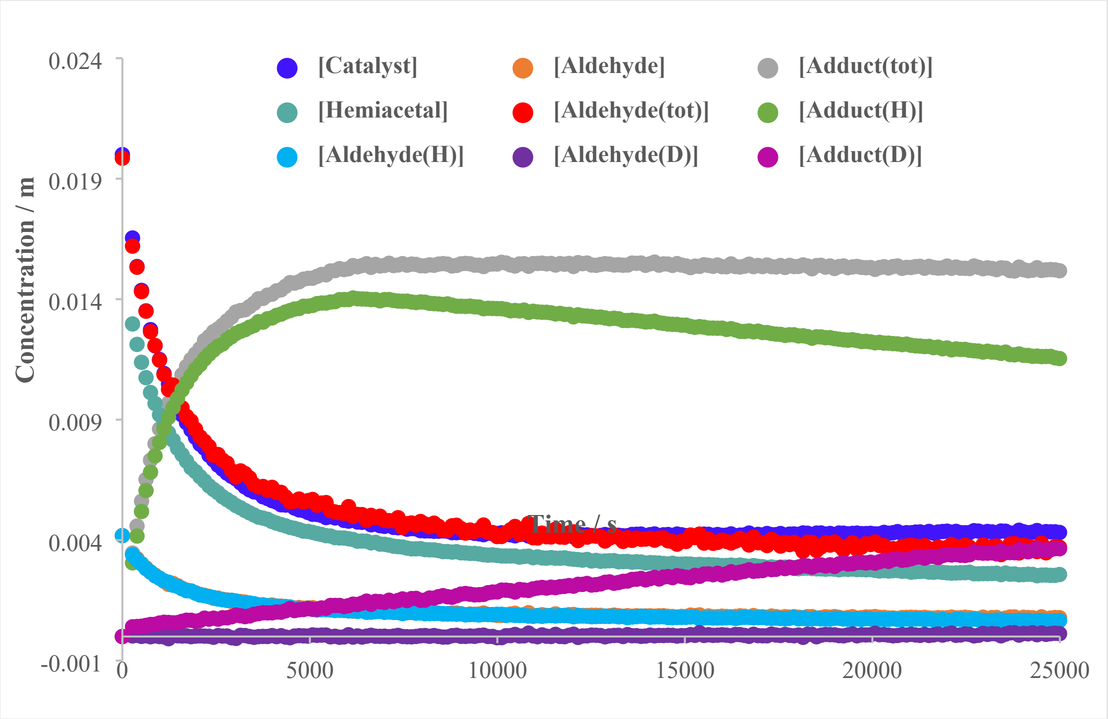

Figure S3. Concentration profile for the self-condensation of 3-methyl-2-pyridinecarboxaldehyde **35** (0.02 M) with *N*-Ph NHC precursor **39** (0.02 M) under a triethylamine buffered (NEt_3:NEt_3·HCl, 2:1, 0.09 M) CD_3OD at 25 °C.

3.4 Determination of k_1, k_{-1} and K

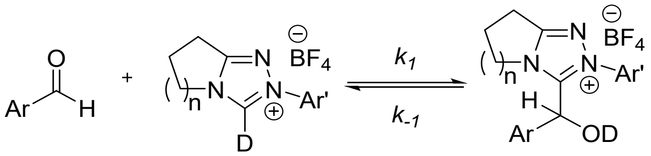

The equilibrium constant of adduct formation, K (M^{-1}), was obtained using Equation 12 (where $[ald]_e$ is the equilibrium concentration of aldehyde, the equilibrium is relatively fast compared to the onward reaction, thus the position of the equilibrium can be accurately determined). The aldehyde concentration at a given timepoint can be expressed by Equation 13. A second-order rate constant for adduct formation from precatalyst and aldehyde, k_1 (M^{-1} s^{-1}), may be obtained based on the consumption of catalyst in the period leading to equilibrium. The expression for the consumption of catalyst is given in Equation 14, which may be rewritten as equation 15 (where $[ald] = f_{ald} \times [ald(tot)]$, $[add] = [cat]_0 - [cat]$, $k_{-1} = k_1/K$). If we set $K' = \frac{[add]_e}{[cat]_e \times [ald(tot)]_e}$, the equation 15 may be rewritten as equation 16.

$$K = \frac{[add]_e}{[cat]_e \times [ald]_e} = \frac{[add]_e}{[cat]_e \times [ald(tot)]_e \times f_{ald}}$$ **Equation 12**

$$[ald] = f_{ald} \times [ald(tot)] = f_{ald} \times ([ald]_0 - ([cat]_0 - [cat]))$$ **Equation 13**

$$\frac{d[cat]}{dt} = -k_1[cat][ald] + k_{-1}[add]$$ **Equation 14**

$$\frac{1}{f_{ald}} \times \frac{d[cat]}{dt} = -k_1[cat][ald(tot)] + k_1\frac{[cat]_e \times [ald(tot)]_e}{[add]_e}([cat]_0 - [cat])$$ **Equation 15**

$$\frac{1}{f_{ald}} \times \frac{d[cat]}{dt} = -k_1[cat][ald(tot)] + \frac{k_1}{K'}([cat]_0 - [cat])$$ **Equation 16**

To simplify the equations, the initial concentration of catalyst, $[cat]_0$, was set to x_0, the initial concentration of aldehyde, $[ald]_0$, to y_0, and concentration of catalyst, $[cat]$, to x. Equation 16 can then be rewritten into equation 17, and the k_1 can be obtained as the slope of the function of x against time (Equation 18).

$$\frac{1}{f_{ald}} \times \frac{dx}{dt} = -k_1x(y_0 - x_0 + x) + \frac{k_1}{K'}(x_0 - x)$$ **Equation 17**

$$k_1 t = \frac{1}{f_{ald}} \times \frac{1}{\sqrt{(y_0 - x_0 + \frac{1}{K'})^2 + \frac{4x_0}{K'}}} \times \left| ln(\frac{x - x_1}{x - x_2}) \right|$$

, where

$$x_1 = \frac{-\left(y_0 - x_0 + \frac{1}{K'}\right) + \sqrt{(y_0 - x_0 + \frac{1}{K'})^2 + \frac{4x_0}{K'}}}{2}$$

$$x_2 = \frac{-\left(y_0 - x_0 + \frac{1}{K'}\right) - \sqrt{(y_0 - x_0 + \frac{1}{K'})^2 + \frac{4x_0}{K'}}}{2}$$

Equation 18

As a result, semilogarithmic plots of $(x-x_1)/(x-x_2)$ against time (shown in Figure S4) can be genenrated. For example, this treatment of the data acquired for the reaction of 3-methyl-2-pyridinecarboxaldehyde **35** (0.02 M) with *N*-Ph NHC precursor **39** (0.02 M) under a triethylamine buffered (NEt_3:NEt_3·HCl, 2:1, 0.09 M) CD_3OD at 25 °C gives the plot shown in Figure S4. k_1 and k$_{-1}$ ($k_{-1} = k_1/K$) can then be determined from the slope of the graph.

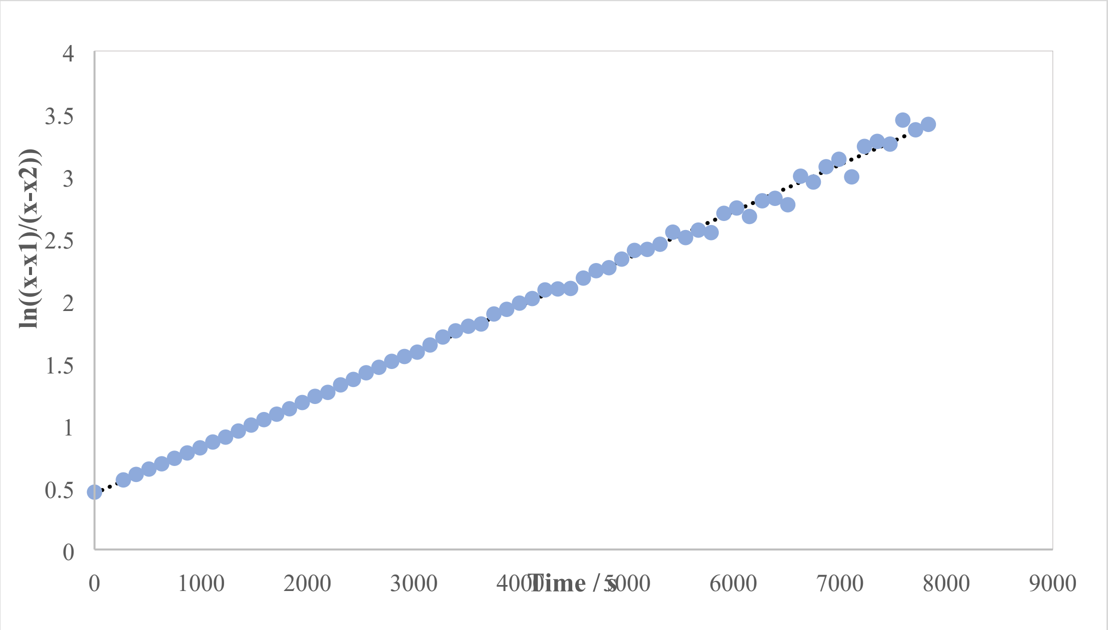

Figure S4. Semilogarithmic plots of $(x-x_1)/(x-x_2)$ against time, obtained from the reaction of 3-methyl-2-pyridinecarboxaldehyde **35** (0.02 M) with *N*-Ph NHC precursor **39** (0.02 M) under a triethylamine buffer (NEt_3:NEt_3·HCl, 2:1, 0.09 M) in CD_3OD at 25 °C.

3.5 Global Fitting Software: Berkeley Madonna

Based on the concentration profile (such as Figure S3) obtained via ^{1}H NMR spectroscopy, Global fitting software Berkeley Madonna was used to fit the data and calculation reaction parameters as a parallel method. The reaction of 3-methyl-2-pyridinecarboxaldehyde **35** (0.02 M) with *N*-Ph NHC precursor **39** (0.02 M) under a triethylamine buffer was applied for data fitting (Figure S5). The model only accounts for the total concentrations of triazolium precatalyst **39** and adduct **12**, which include both deuterated and protonated components, and the concentrations of aldehyde **35**, and hemiacetal. This means that aldehyde-methanol adduct equilibrium and aldehyde-triazolium adduct equilibrium were fitted simultaneously in this software. Thus, the kinetic parameters (such as k_1, k_{-1}, K) of the aldehyde-triazolium adduct equilibrium were obtained from the global fitting software.

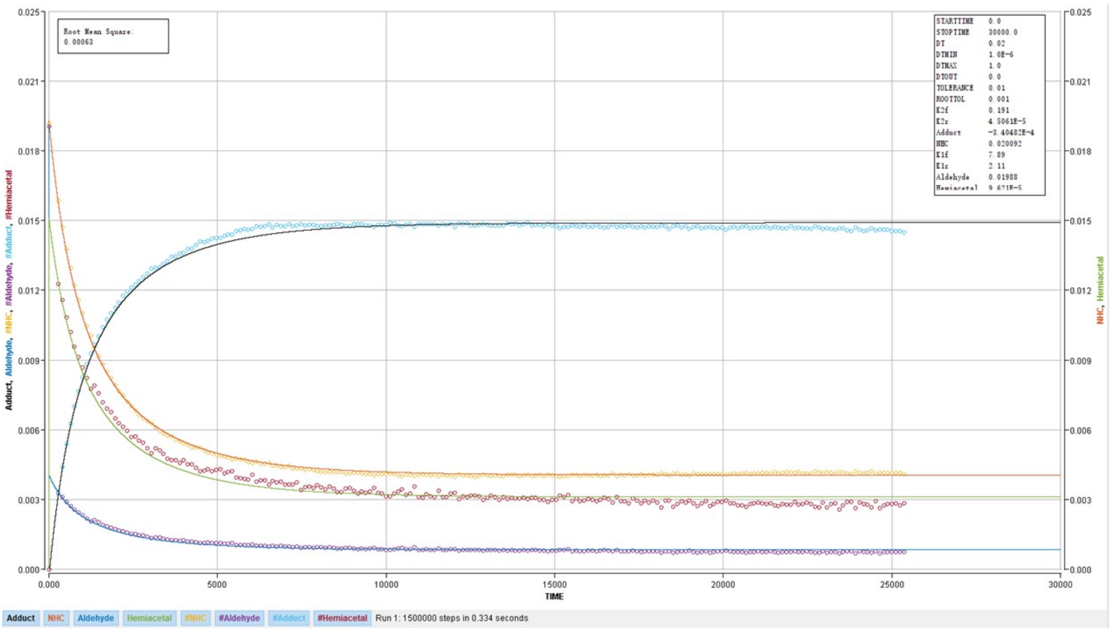

Figure S5. Global fitting profile for the reaction of 3-methyl-2-pyridinecarboxaldehyde **35** (0.02 M) with *N*-Ph NHC precursor **39** (0.02 M) under a triethylamine buffer (NEt_3:NEt_3·HCl, 2:1, 0.09 M) in CD_3OD at 25 °C. Open circles show the experimental data, with the solid line representing the fit to the kinetic model. Fitting data from t = 0 to t = 30000 s from Figure S3.

3.6 Determination of k_2

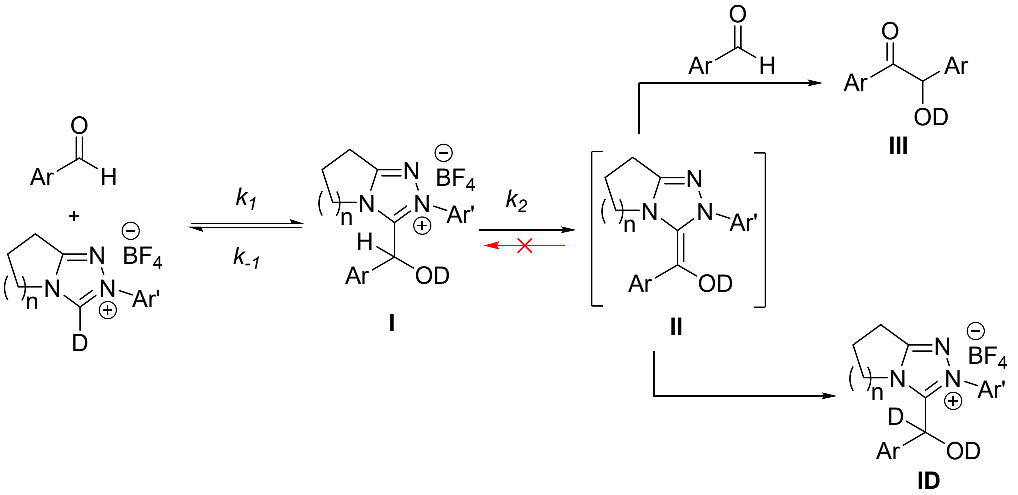

Scheme 3. Mechanism of Breslow intermediate formation and consumption by onward reactions.

According to Scheme 3, a pseudo-first-order rate constant for adduct deprotonation to give the transient Breslow intermediate **II**, k_2 (s^{-1}), may be estimated from the consumption of protonated adduct once the equilibrium concentration of the species has been reached. In methanol-d_4, formation of the Breslow intermediate **II** from **I** is essentially irreversible and subsequent reactions lead to deuterated adduct **ID** or product **III**. The expression for the rate of adduct consumption is given in Equation 19 and the integrated rate equation is shown in Equation 20.

$$-\frac{d[add\ (H)]}{dt} = k_2[add(H)] \qquad \textbf{Equation 19}$$

$$[add\ (H)] = [add\ (H)]_0 e^{-k_2 t} \qquad \textbf{Equation 20}$$

Therefore, semilogarithmic plots of [Adduct (H)] against time (shown in Figure S6), obtained using the data acquired for the reaction of 3-methyl-2-pyridinecarboxaldehyde **35** (0.02 M) with *N*-Ph NHC precursor **39** (0.02 M) in triethylamine buffer (NEt_3:NEt_3·HCl, 2:1, 0.09 M) in CD_3OD at 25 °C were prepared and k_2 was determined from this slope of the graph.

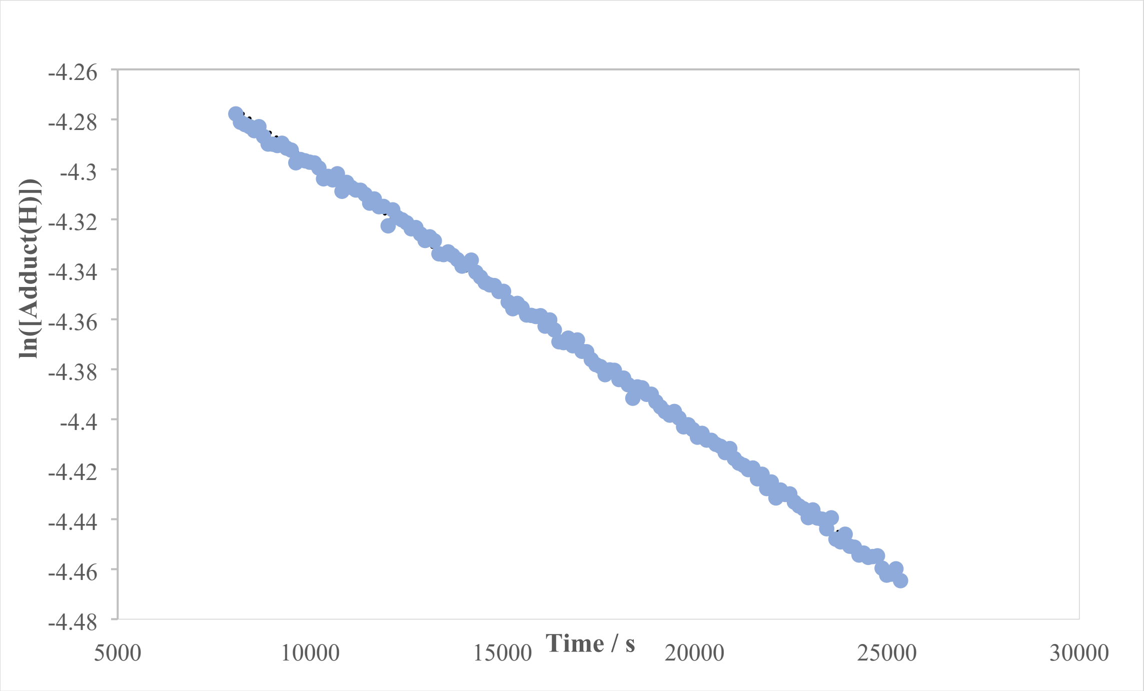

Figure S6. Semilogarithmic plots of [Adduct (H)] against time for the reactions of 3-methyl-2-pyridinecarboxaldehyde **35** (0.02 M) with *N*-Ph NHC precursor **39** (0.02 M) under a triethylamine buffer (NEt_3:NEt_3·HCl, 2:1, 0.09 M) in CD_3OD at 25 °C.

Table 1 Entry 1

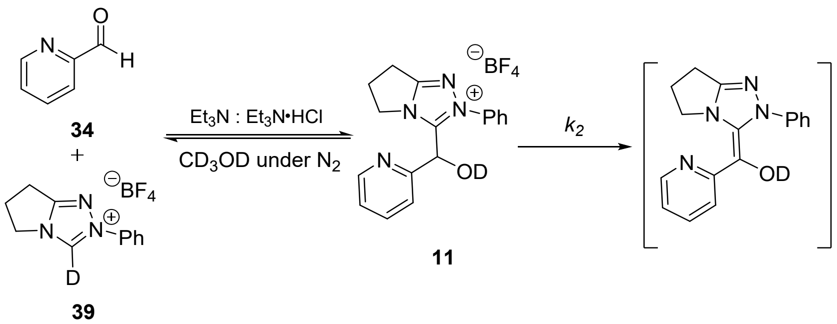

The reaction of aldehyde **34** and triazolium precatalyst **39** was monitored using ^{1}H NMR spectra, with representative NMR spectra over the course of the experiment given in Figure S7.

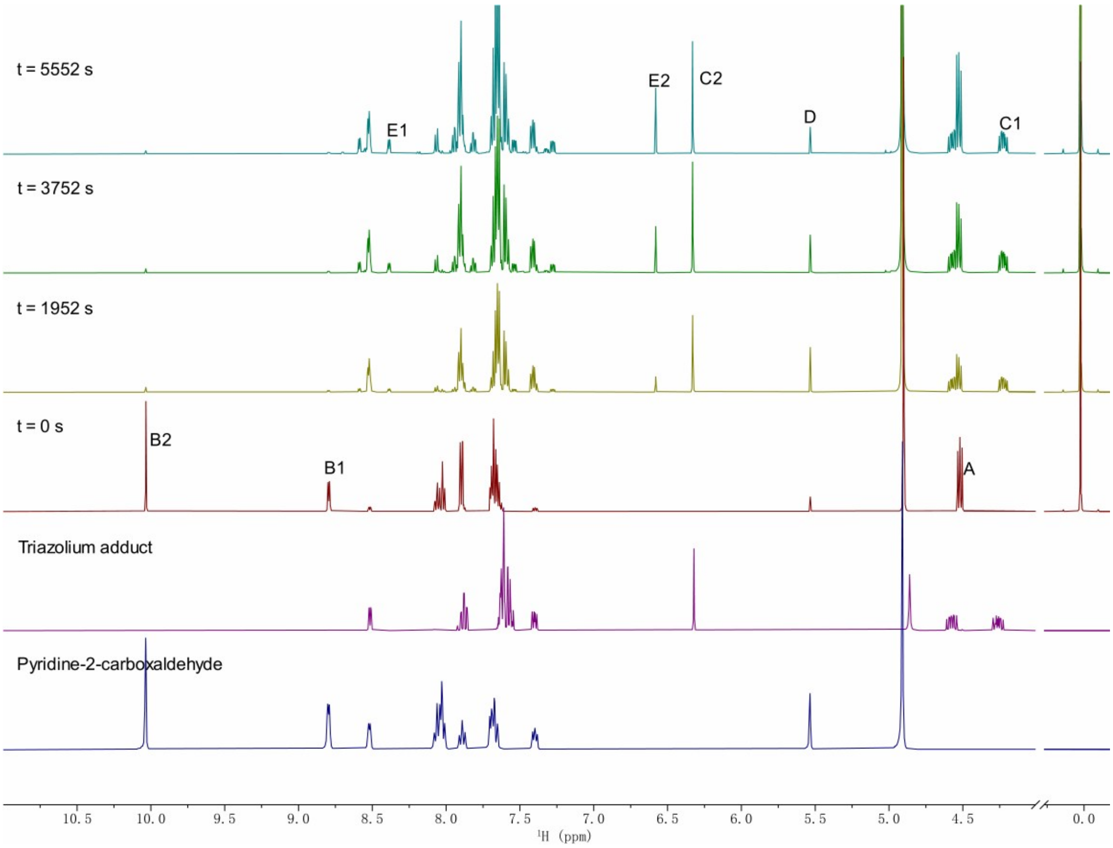

Figure S7. Representative ^{1}H NMR spectra (400 MHz) for reaction of pyridine-2-carboxaldehyde **34** (0.02 M) with *N*-Ph NHC precursor **39** (0.02 M) under a triethylamine buffer (NEt_3:NEt_3·HCl, 2:1, 0.09 M) in CD_3OD at 25 °C A = NHC precursor NCH_2, B1 =Ar*H*CHO, B2 =ArHC*H*O, C1 = Adduct NC$H_A$$H_B$, C2 =Adduct C(α)*H*, D = Hemiacetal C*H*, E1 = Benzoin Ar*H*, E2 = Benzoin C*H*.

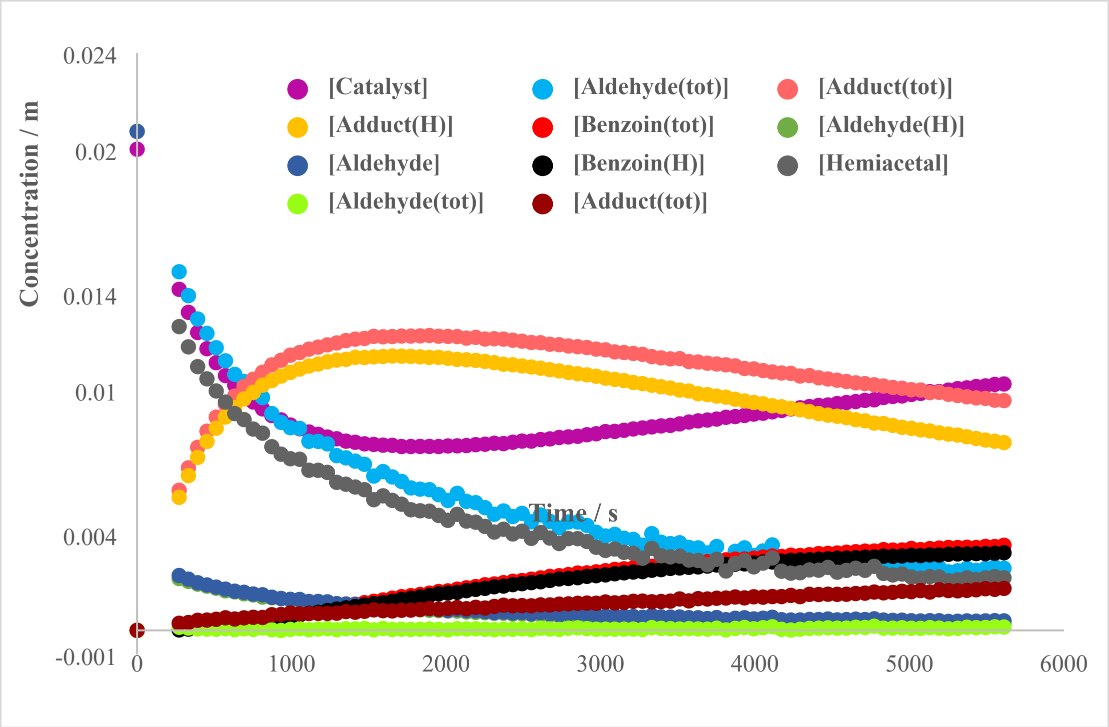

Figure S8. Concentration profile for the self-condensation of pyridine-2-carboxaldehyde **34** (0.02

M) with *N*-Ph NHC precursor **39** (0.02 M) under a triethylamine buffer (NEt_3:NEt_3·HCl, 2:1, 0.09 M) in CD_3OD at 25 °C.

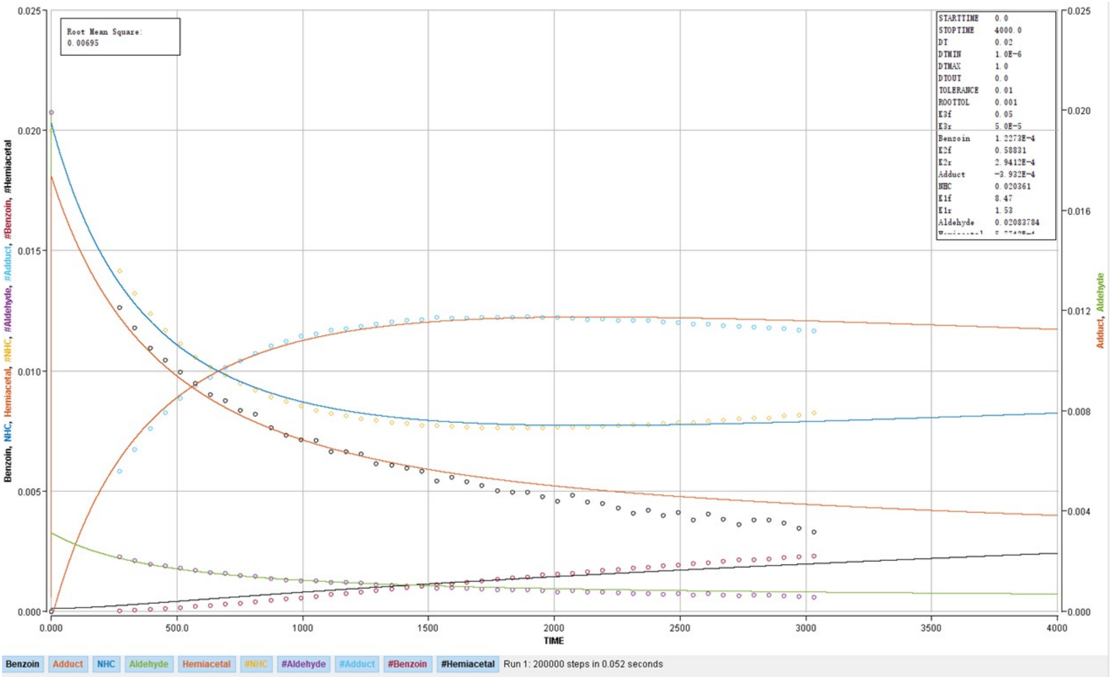

Figure S9. Global fitting profile for the reaction of pyridine-2-carboxaldehyde **34** (0.02 M) with *N*-Ph NHC precursor **39** (0.02 M) under a triethylamine buffer (NEt_3:NEt_3·HCl, 2:1, 0.09 M) in CD_3OD at 25 °C. Open circles show the experimental data, with the solid line representing the fit to the kinetic model. Fitting data from t = 0 to t = 4000 s from Figure S8.

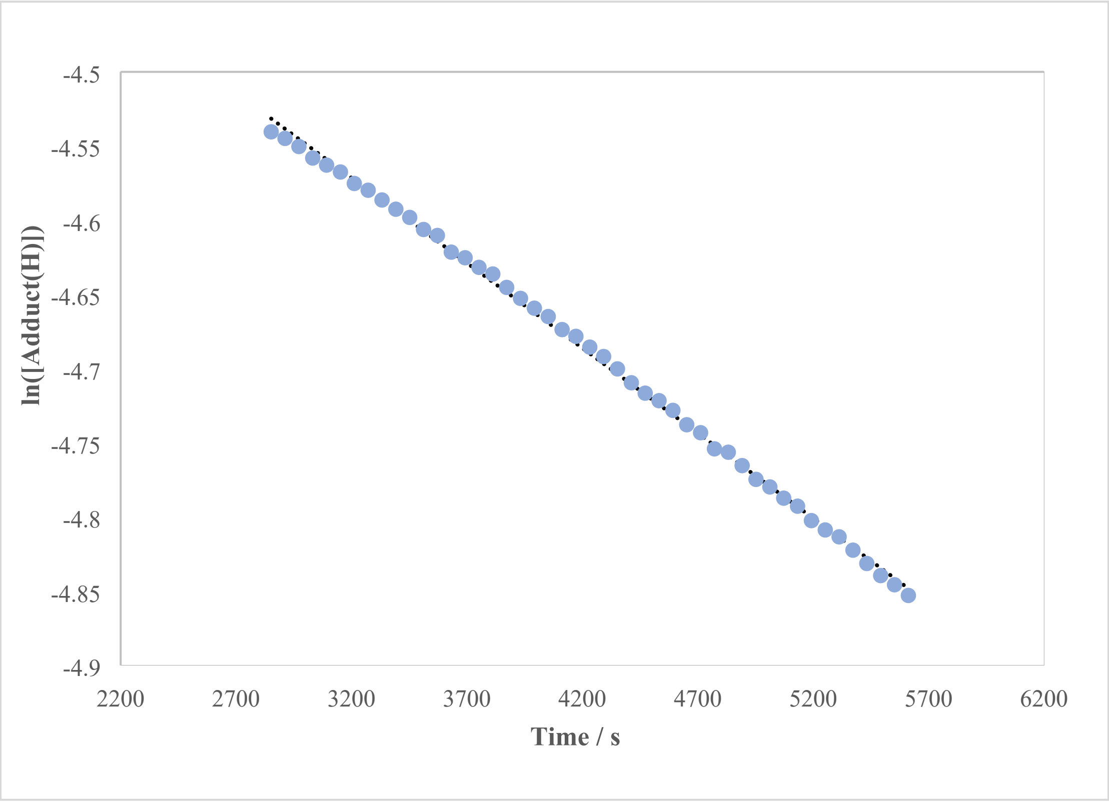

Figure S10. Semilogarithmic plots of [Adduct (H)] against time for the reactions of pyridine-2-carboxaldehyde **34** (0.02 M) with *N*-Ph NHC precursor **39** (0.02 M) under a triethylamine buffer (NEt_3:NEt_3·HCl, 2:1, 0.09 M) in CD_3OD at 25 °C.

Table 1 Entry 2

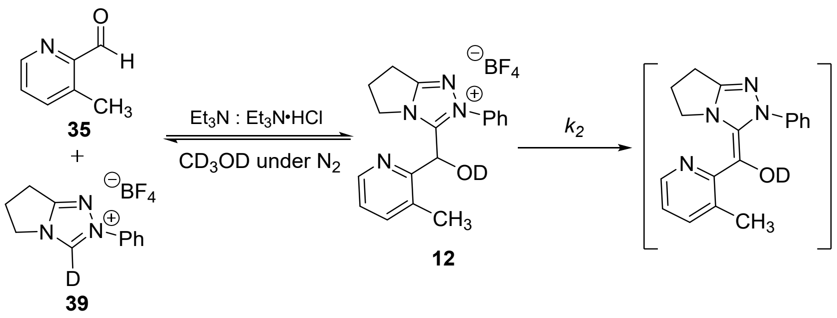

The reaction of aldehyde **35** and triazolium precatalyst **39** was monitored using ^{1}H NMR spectra, with representative NMR spectra over the course of the experiment given in Figure S2.

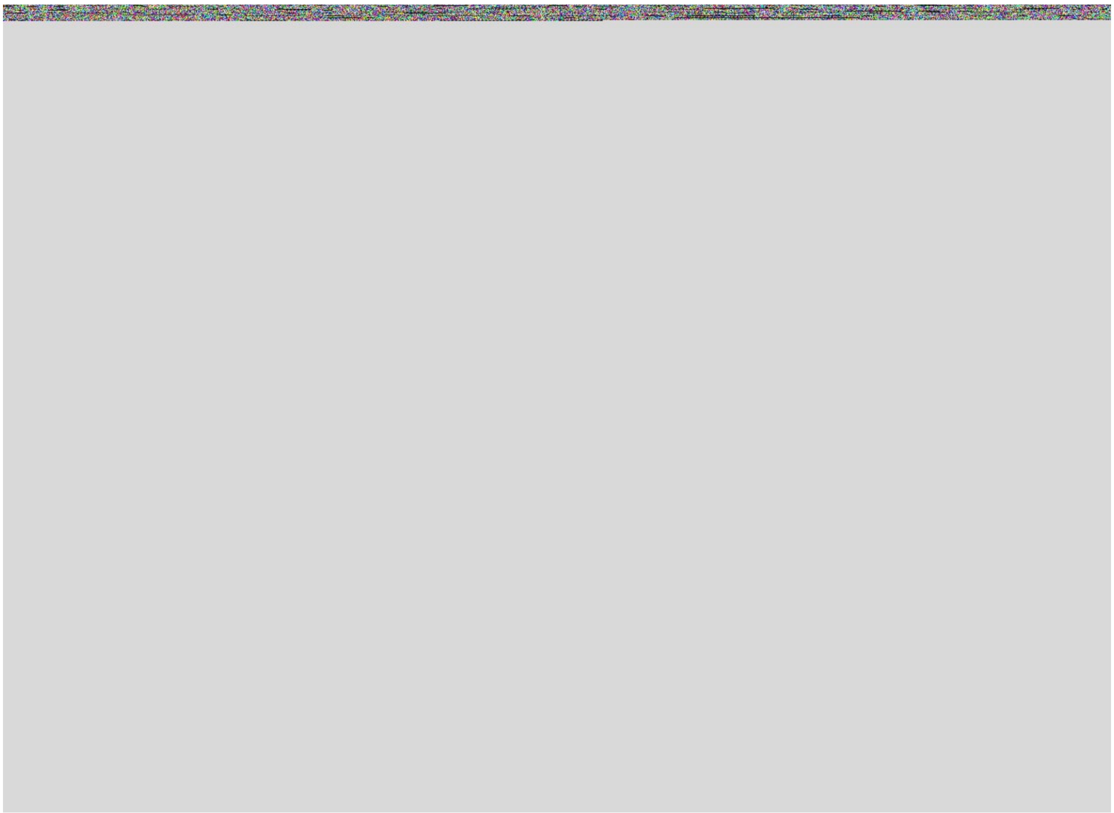

Figure S2. Representative ^{1}H NMR spectra (400 MHz) for reaction of 3-methyl-2-pyridinecarboxaldehyde **35** (0.02 M) with *N*-Ph NHC precursor **39** (0.02 M) under a triethylamine buffered (NEt_3:NEt_3·HCl, 2:1, 0.09 M) CD_3OD at 25 °C. A = NHC precursor NC*H*$_2$, B1 =Ar*H*CHO, B2 =ArHC*H*O, C1 = Adduct NC*H*$_2$, C2 =Tetrahedral Adduct C(α)*H,* D = Hemiacetal C*H.*

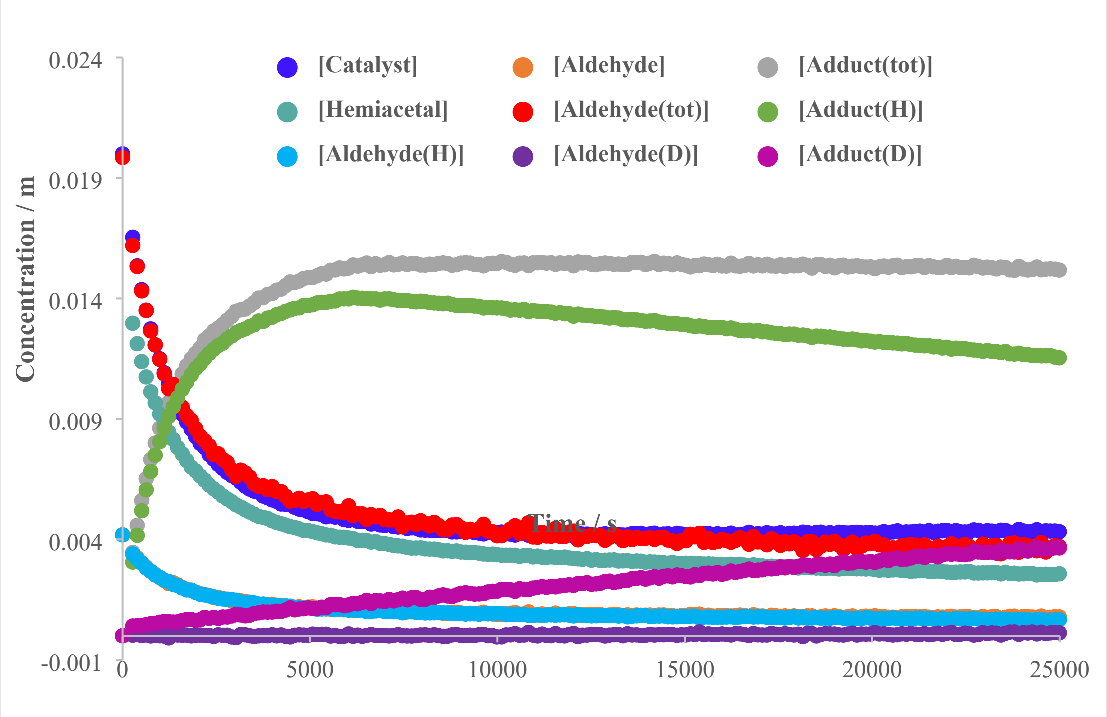

Figure S3. Concentration profile for the self-condensation of 3-methyl-2-pyridinecarboxaldehyde **35** (0.02 M) with *N*-Ph NHC precursor **39** (0.02 M) under a triethylamine buffer (NEt_3:NEt_3·HCl, 2:1, 0.09 M) in CD_3OD at 25 °C.

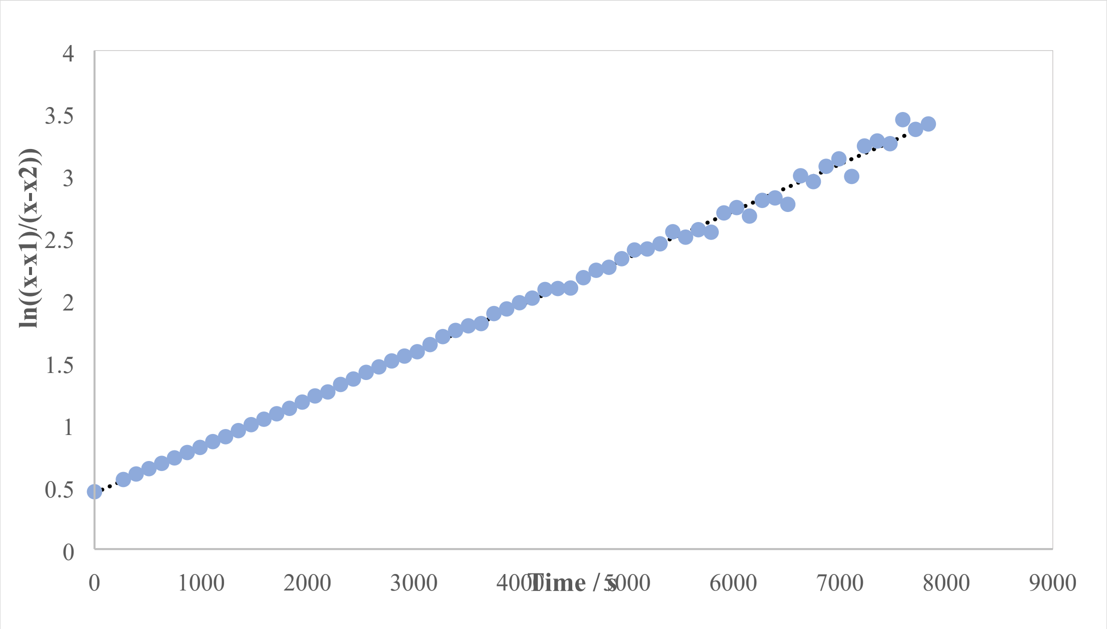

Figure S4. Semilogarithmic plots of $(x\text{-}x_1)/(x\text{-}x_2)$ against time, obtained from the reaction of 3-methyl-2-pyridinecarboxaldehyde **35** (0.02 M) with *N*-Ph NHC precursor **39** (0.02 M) under a triethylamine buffer (NEt_3:NEt_3·HCl, 2:1, 0.09 M) in CD_3OD at 25 °C.

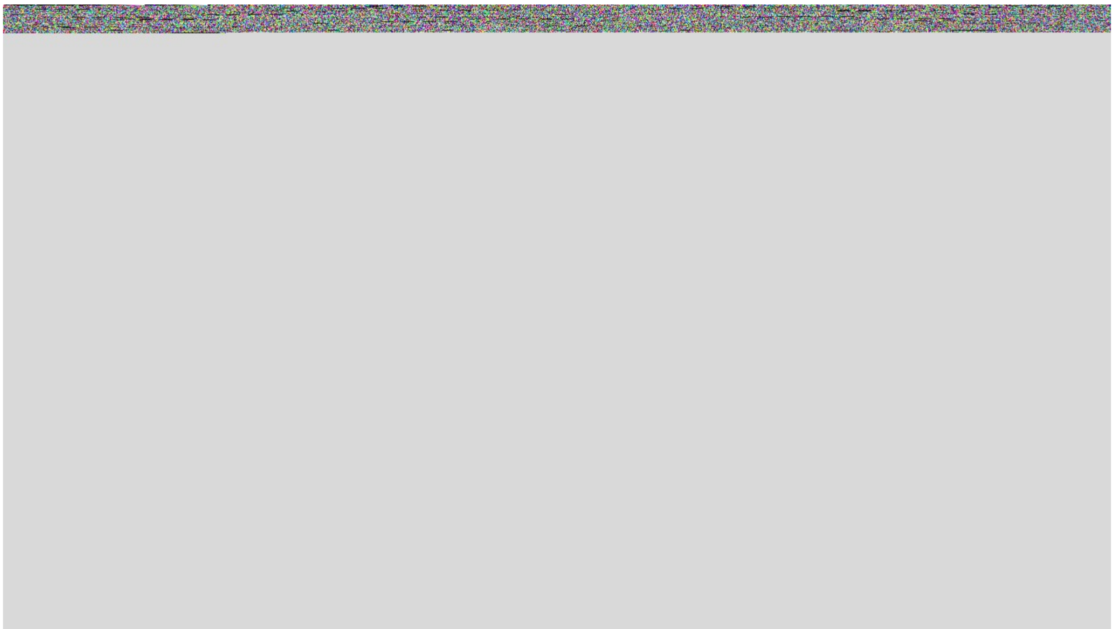

Figure S5. Global fitting profile for the reaction of 3-methyl-2-pyridinecarboxaldehyde **35** (0.02 M) with *N*-Ph NHC precursor **39** (0.02 M) under a triethylamine buffer (NEt_3:NEt_3·HCl, 2:1, 0.09 M) in CD_3OD at 25 °C. Open circles show the experimental data, with the solid line representing the fit to the kinetic model. Fitting data from t = 0 to t = 30000 s from Figure S3.

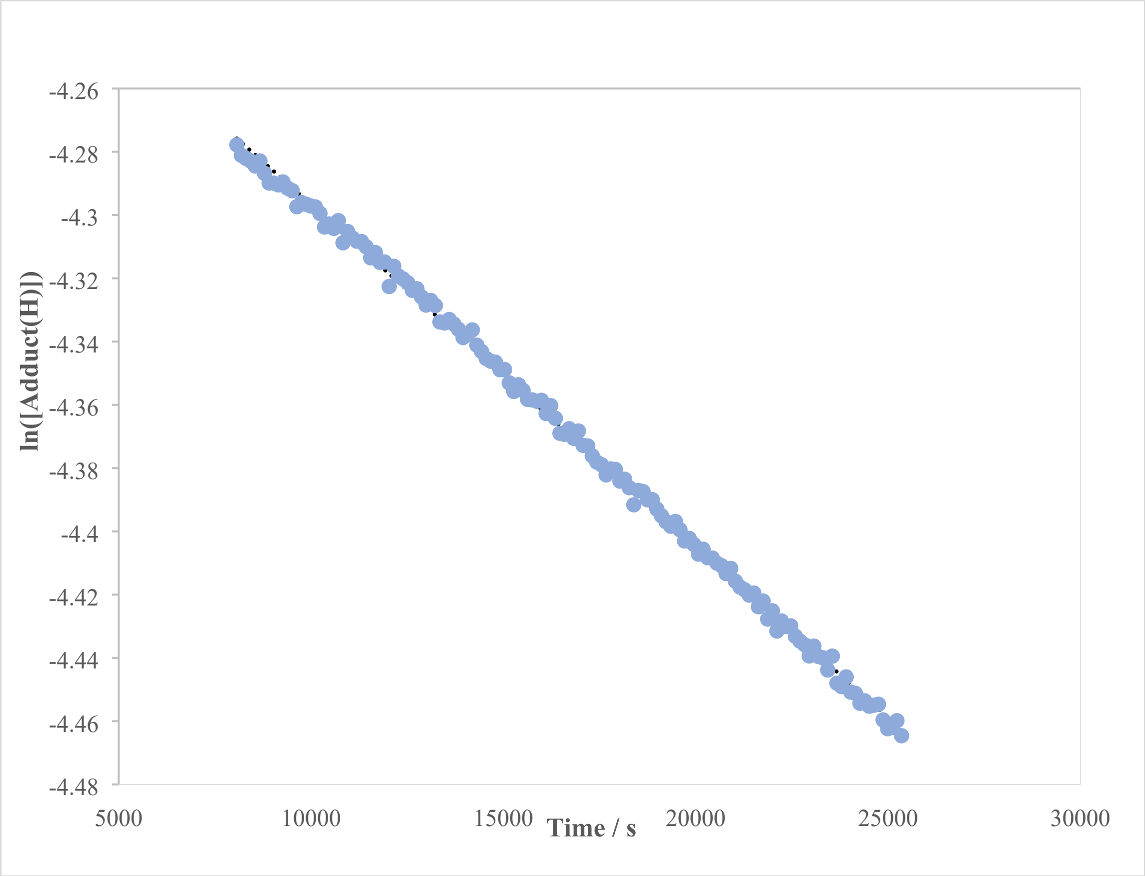

Figure S6. Semilogarithmic plots of [Adduct (H)] against time for the reactions of 3-methyl-2-pyridinecarboxaldehyde **35** (0.02 M) with *N*-Ph NHC precursor **39** (0.02 M) under a triethylamine buffer (NEt_3:NEt_3·HCl, 2:1, 0.09 M) in CD_3OD at 25 °C.

Table 1 Entry 3

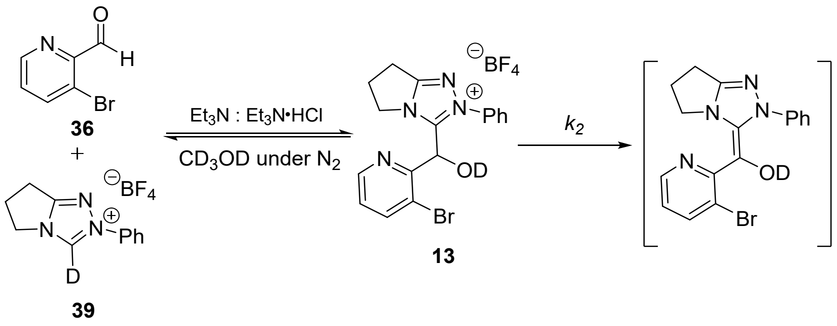

The reaction of aldehyde **36** and triazolium precatalyst **39** was monitored using ^{1}H NMR spectra, with representative NMR spectra over the course of the experiment given in Figure S11.

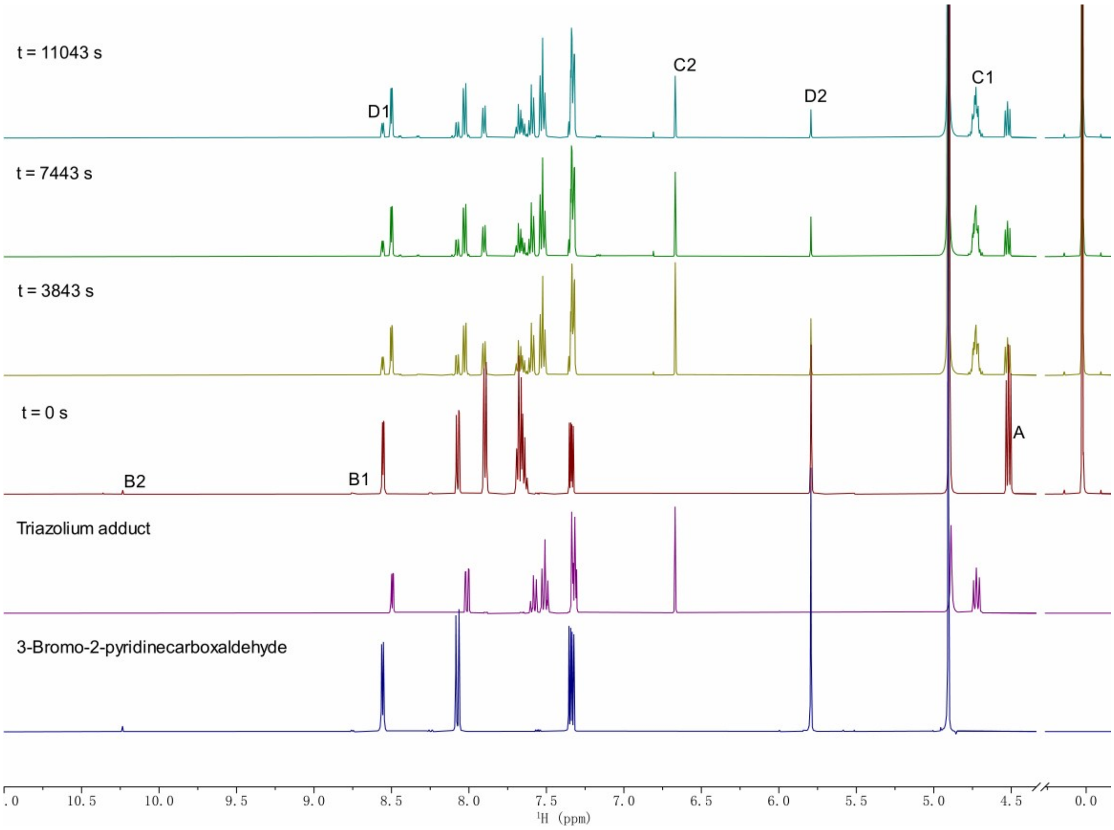

Figure S11. Representative ^{1}H NMR spectra (400 MHz) for reaction of 3-bromo-2-pyridinecarboxaldehyde **36** (0.02 M) with *N*-Ph NHC precursor **39** (0.02 M) under a triethylamine buffer (NEt_3:NEt_3·HCl, 2:1, 0.09 M) in CD_3OD at 25 °C. A = NHC precursor NCH_2, B1 =Ar*H*CHO, B2 =ArHC*H*O, C1 = Adduct NCH_2, C2 =Adduct C(α)*H,* D1 = Hemiacetal Ar*H*, D2 = Hemiacetal C*H*.

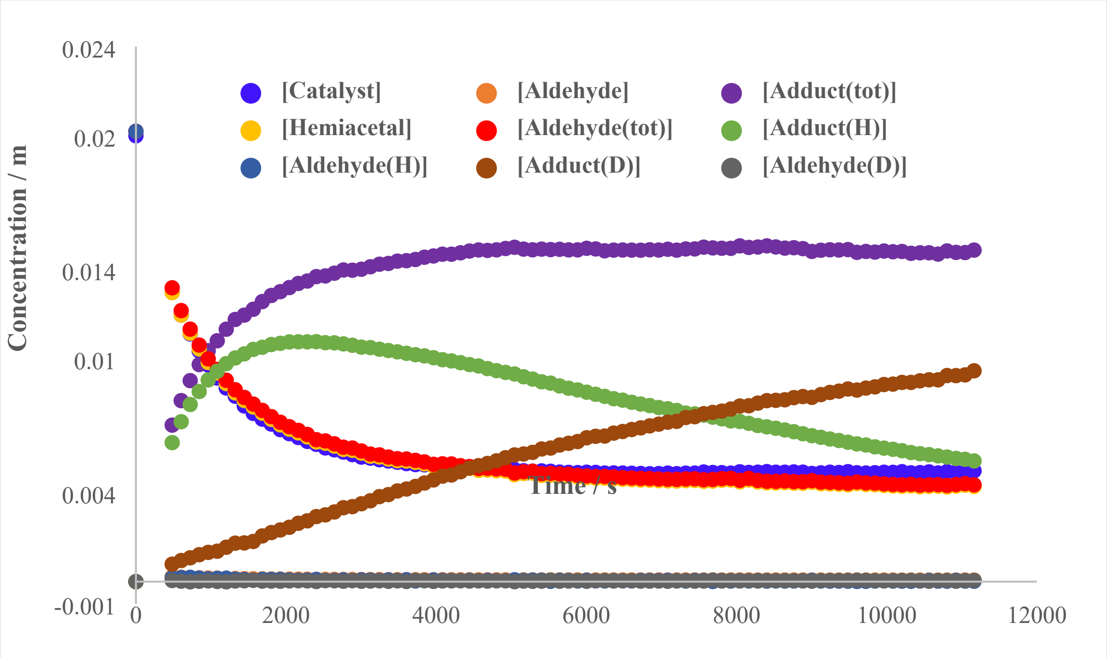

Figure S12. Concentration profile for the self-condensation of 3-bromo-2-pyridinecarboxaldehyde **36** (0.02 M) with *N*-Ph NHC precursor **39** (0.02 M) under a triethylamine buffer (NEt_3:NEt_3·HCl, 2:1, 0.09 M) in CD_3OD at 25 °C.

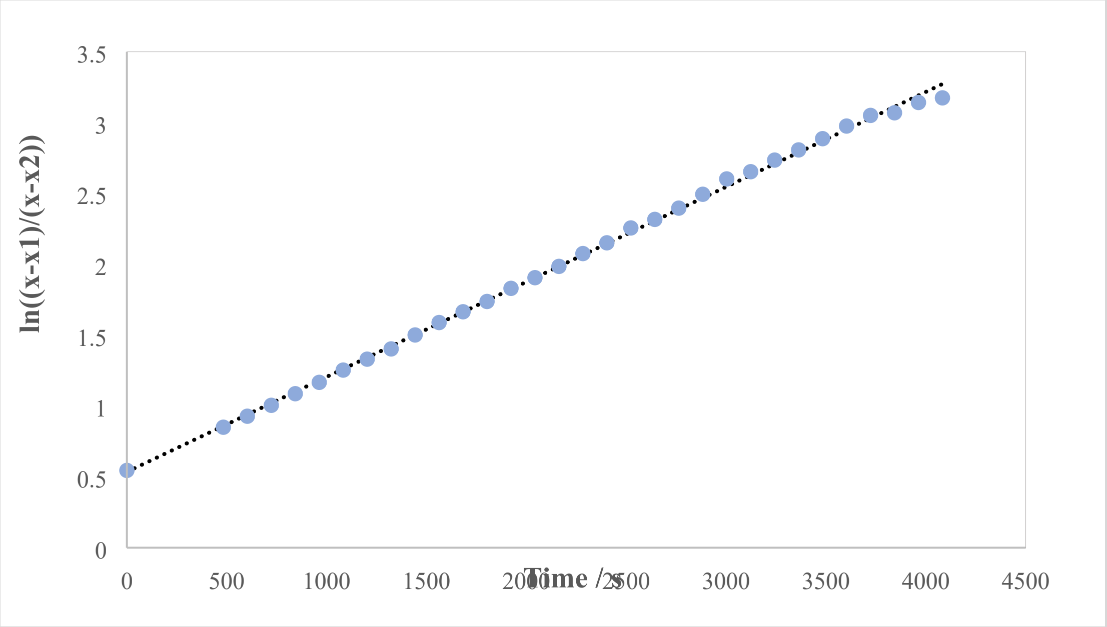

Figure S13. Semilogarithmic plots of (x-x_1)/(x-x_2) against time, obtained from the reaction of 3-bromo-2-pyridinecarboxaldehyde **36** (0.02 M) with *N*-Ph NHC precursor **39** (0.02 M) under a triethylamine buffer (NEt_3:NEt_3·HCl, 2:1, 0.09 M) in CD_3OD at 25 °C.

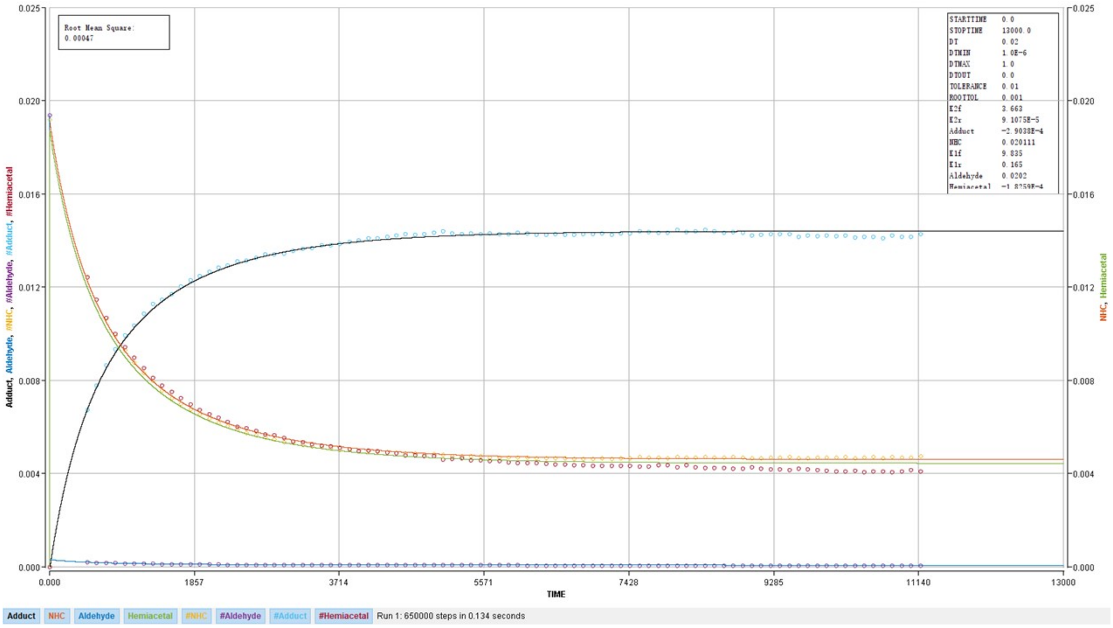

Figure S14. Global fitting profile for the reaction of 3-bromo-2-pyridinecarboxaldehyde **36** (0.02 M) with *N*-Ph NHC precursor **39** (0.02 M) under a triethylamine buffer (NEt_3:NEt_3·HCl, 2:1, 0.09 M) in CD_3OD at 25 °C. Open circles show the experimental data, with the solid line representing the fit to the kinetic model. Fitting data from t = 0 to t = 13000 s from Figure S12.

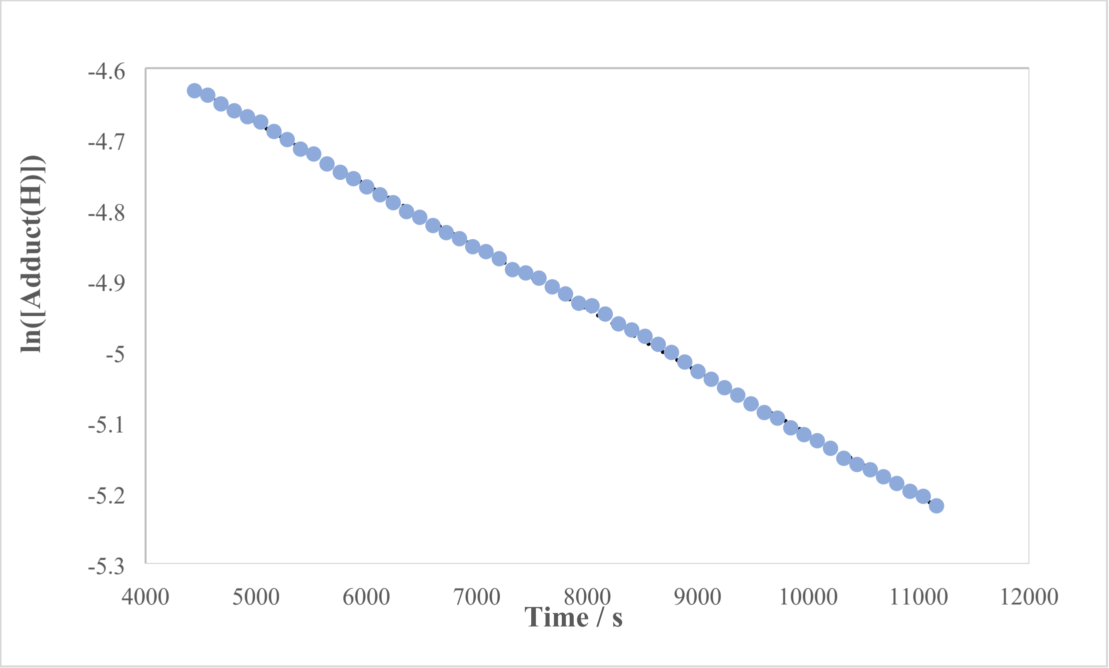

Figure S15. Semilogarithmic plots of [Adduct (H)] against time for the reactions of 3-bromo-2-pyridinecarboxaldehyde **36** (0.02 M) with *N*-Ph NHC precursor **39** (0.02 M) under a triethylamine buffer (NEt_3:NEt_3·HCl, 2:1, 0.09 M) in CD_3OD at 25 °C.

Table 1 Entry 4

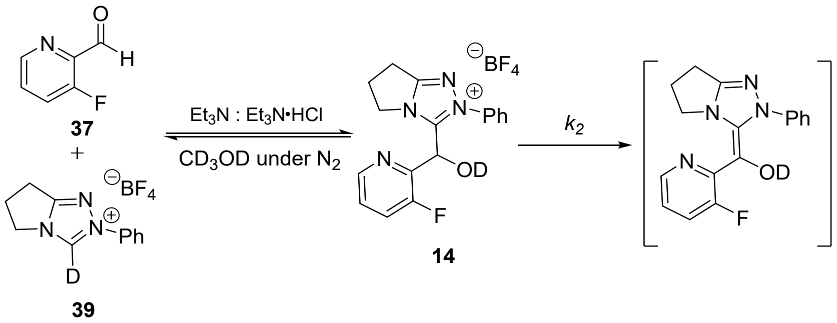

The reaction of aldehyde **37** and triazolium precatalyst **39** was monitored using ^{1}H NMR spectra, with representative NMR spectra over the course of the experiment given in Figure S16.

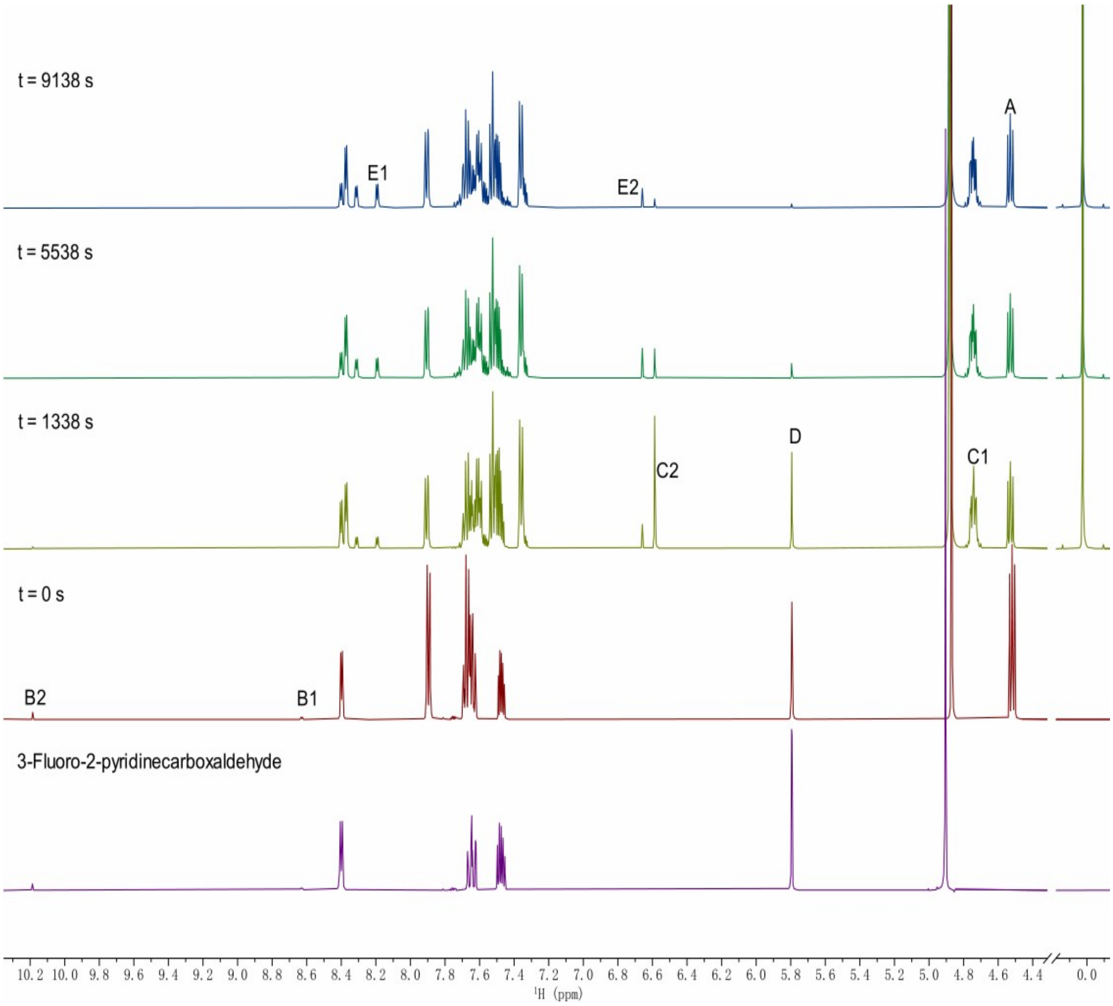

Figure S16. Representative ^{1}H NMR spectra (400 MHz) for reaction of 3-fluoro-2-pyridinecarboxaldehyde **37** (0.02 M) with *N*-Ph NHC precursor **39** (0.02 M) under a triethylamine buffer (NEt_3:NEt_3·HCl, 2:1, 0.09 M) in CD_3OD at 25 °C. A = NHC precursor NCH_2, B1 =Ar*H*CHO, B2 =ArHC*H*O, C1 = Adduct NCH_2, C2 =Adduct C(α)*H*, D = Hemiacetal C*H*, E1 = Benzoin Ar*H*, E2 = Benzoin C*H*.

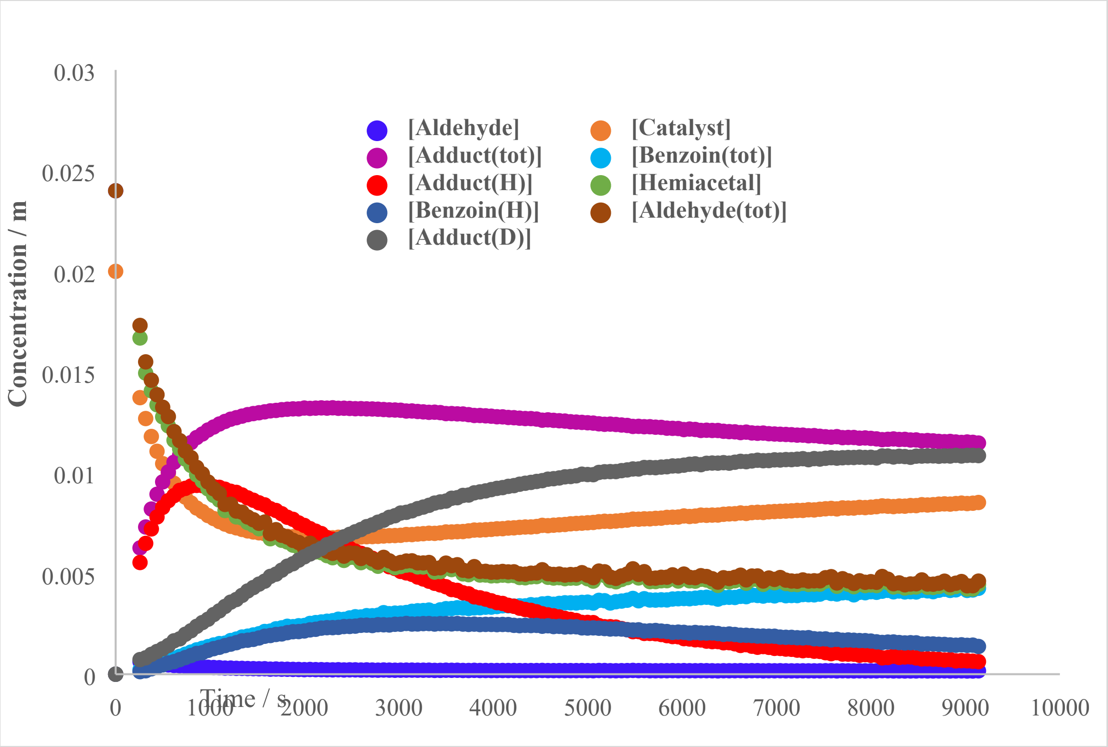

Figure S17. Concentration profile for the self-condensation of 3-fluoro-2-pyridinecarboxaldehyde **37** (0.02 M) with *N*-Ph NHC precursor **39** (0.02 M) under a triethylamine buffer (NEt_3:NEt_3·HCl, 2:1, 0.09 M) in CD_3OD at 25 °C.

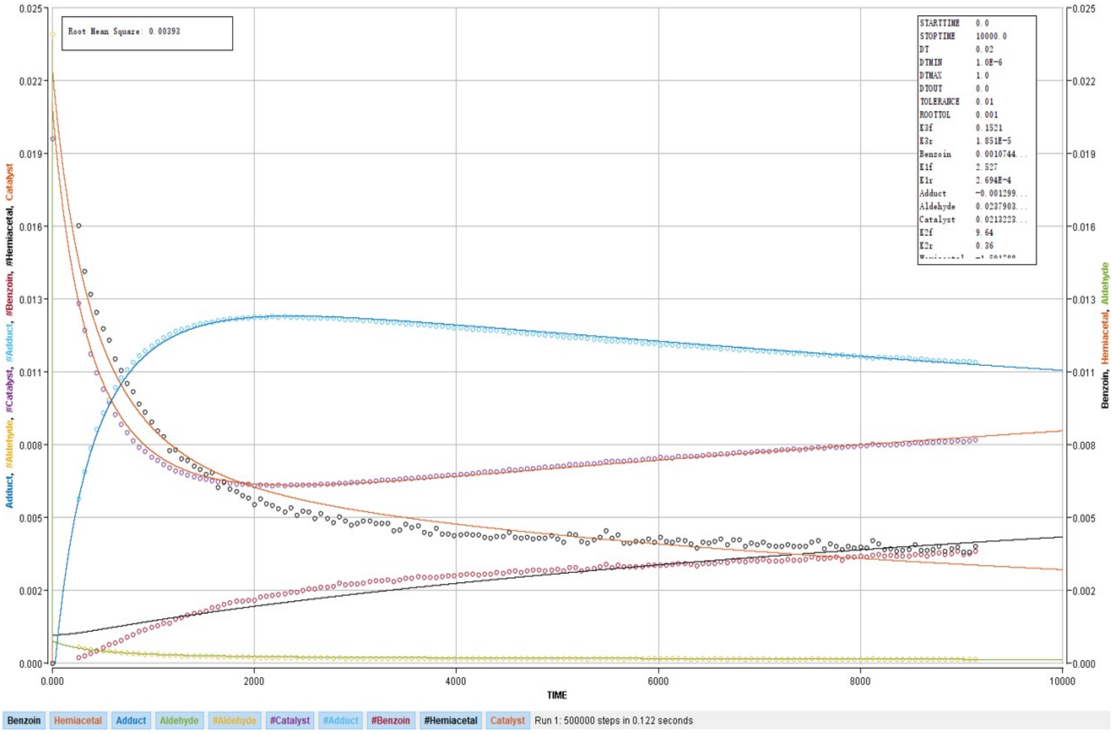

Figure S18. Global fitting profile for the reaction of 3-fluoro-2-pyridinecarboxaldehyde **37** (0.02 M) with *N*-Ph NHC precursor **39** (0.02 M) under a triethylamine buffer (NEt_3:NEt_3·HCl, 2:1, 0.09 M) in CD_3OD at 25 °C. Open circles show the experimental data, with the solid line representing the fit to the kinetic model. Fitting data from t = 0 to t = 10000 s from Figure S17.

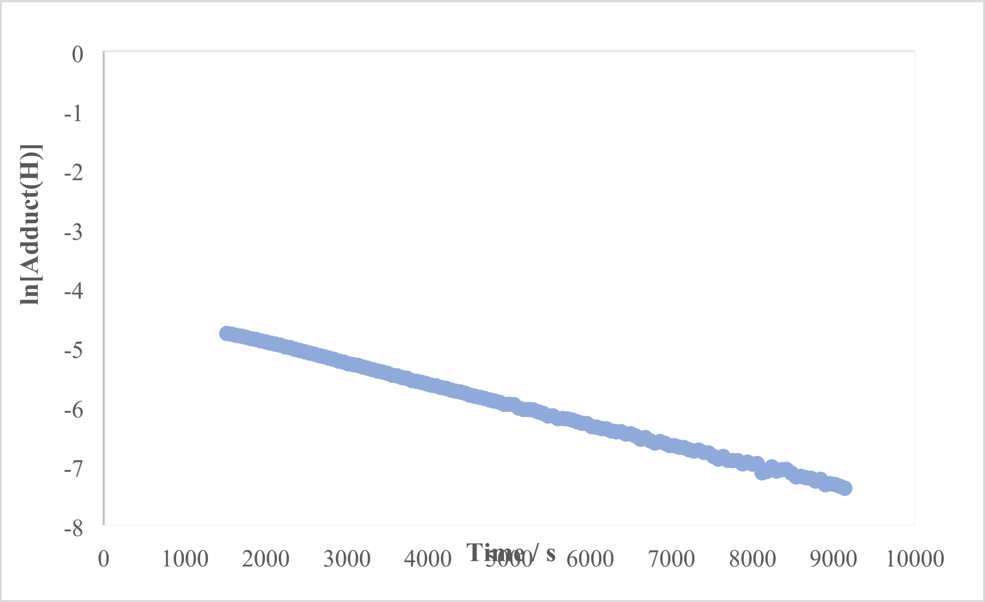

Figure S19. Semilogarithmic plots of [Adduct (H)] against time for the reactions of 3-fluoro-2-pyridinecarboxaldehyde **37** (0.02 M) with *N*-Ph NHC precursor **39** (0.02 M) under a triethylamine buffer (NEt_3:NEt_3·HCl, 2:1, 0.09 M) in CD_3OD at 25 °C.

Table 1 Entry 5

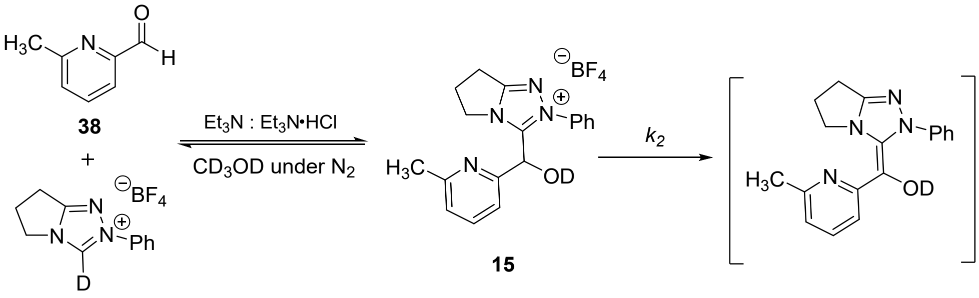

The reaction of aldehyde **38** and triazolium precatalyst **39** was monitored using ^{1}H NMR spectra, with representative NMR spectra over the course of the experiment given in Figure S20.

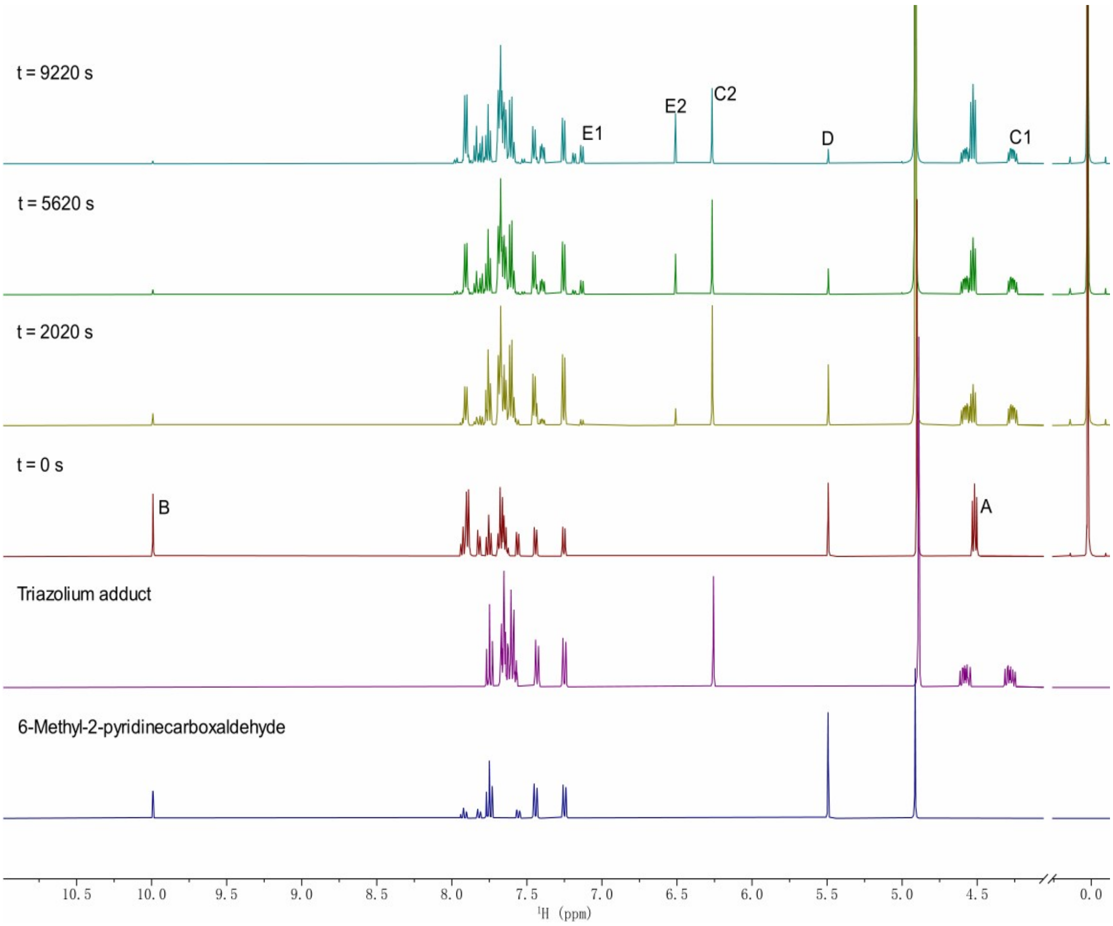

Figure S20. Representative ^{1}H NMR spectra (400 MHz) for reaction of 6-methyl-2-pyridinecarboxaldehyde **38** (0.02 M) with *N*-Ph NHC precursor **39** (0.02 M) under a triethylamine buffer (NEt_3:NEt_3·HCl, 2:1, 0.09 M) in CD_3OD at 25 °C. A = NHC precursor NCH_2, B =ArHC*H*O (The integral of aldehydic proton B is used to calculate the approximate [Aldehyde] as all the other protons of the aldehyde are overlapped with other components in the experiment), C1 = Adduct NC$H_A$$H_B$, C2 =Adduct C(α)*H,* D = Hemiacetal C*H*, E1 = Benzoin Ar*H*, E2 = Benzoin C*H*.

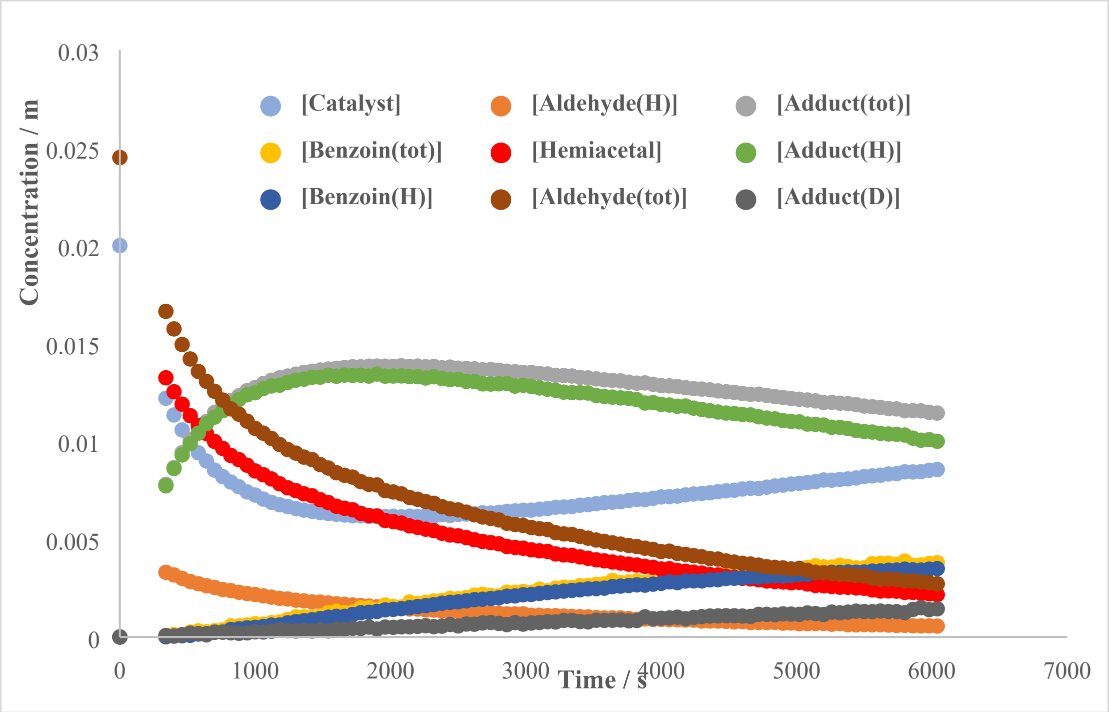

Figure S21. Concentration profile for the self-condensation of 6-methyl-2-pyridinecarboxaldehyde **38** (0.02 M) with *N*-Ph NHC precursor **39** (0.02 M) under a triethylamine buffer (NEt_3:NEt_3·HCl, 2:1, 0.09 M) in CD_3OD at 25 °C.

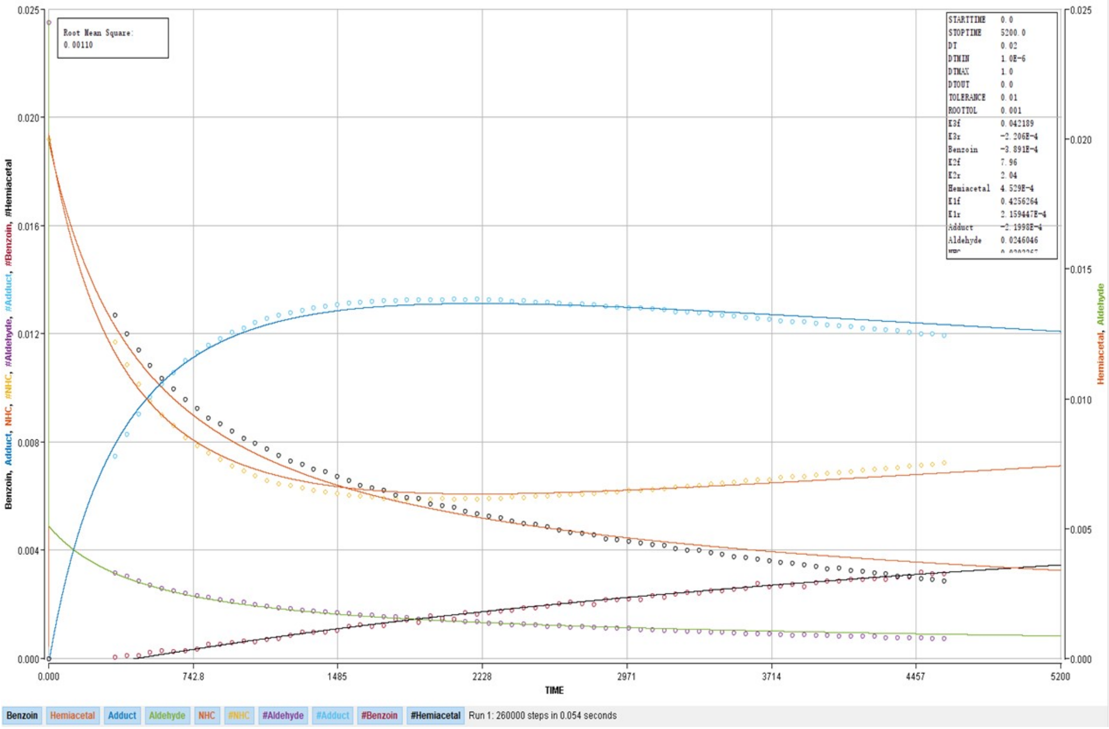

Figure S22. Global fitting profile for the reaction of 6-methyl-2-pyridinecarboxaldehyde **38** (0.02 M) with *N*-Ph NHC precursor **39** (0.02 M) under a triethylamine buffer (NEt_3:NEt_3·HCl, 2:1, 0.09 M) in CD_3OD at 25 °C. Open circles show the experimental data, with the solid line representing the fit to the kinetic model. Fitting data from t = 0 to t = 5200 s from Figure S21.

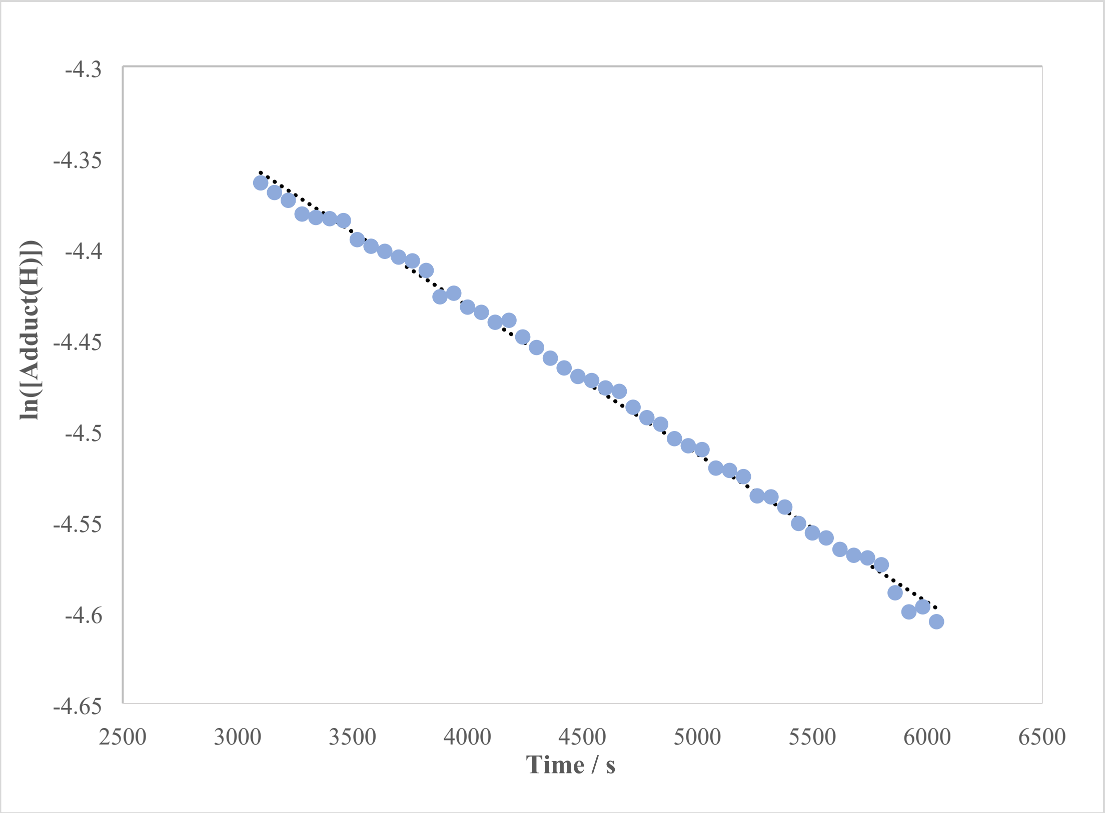

Figure S23. Semilogarithmic plots of [Adduct (H)] against time for the reactions of 6-methyl-2-pyridinecarboxaldehyde **38** (0.02 M) with *N*-Ph NHC precursor **39** (0.02 M) under a triethylamine buffer (NEt_3:NEt_3·HCl, 2:1, 0.09 M) in CD_3OD at 25 °C.

Table 1 Entry 6

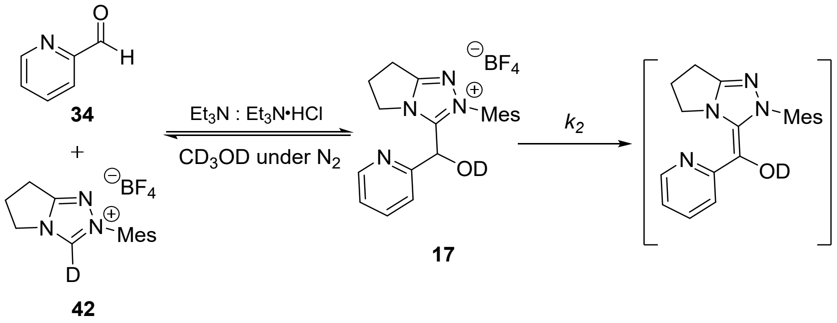

The reaction of aldehyde **34** and triazolium precatalyst **42** was monitored using ^1H NMR spectra, with representative NMR spectra over the course of the experiment given in Figure S24.

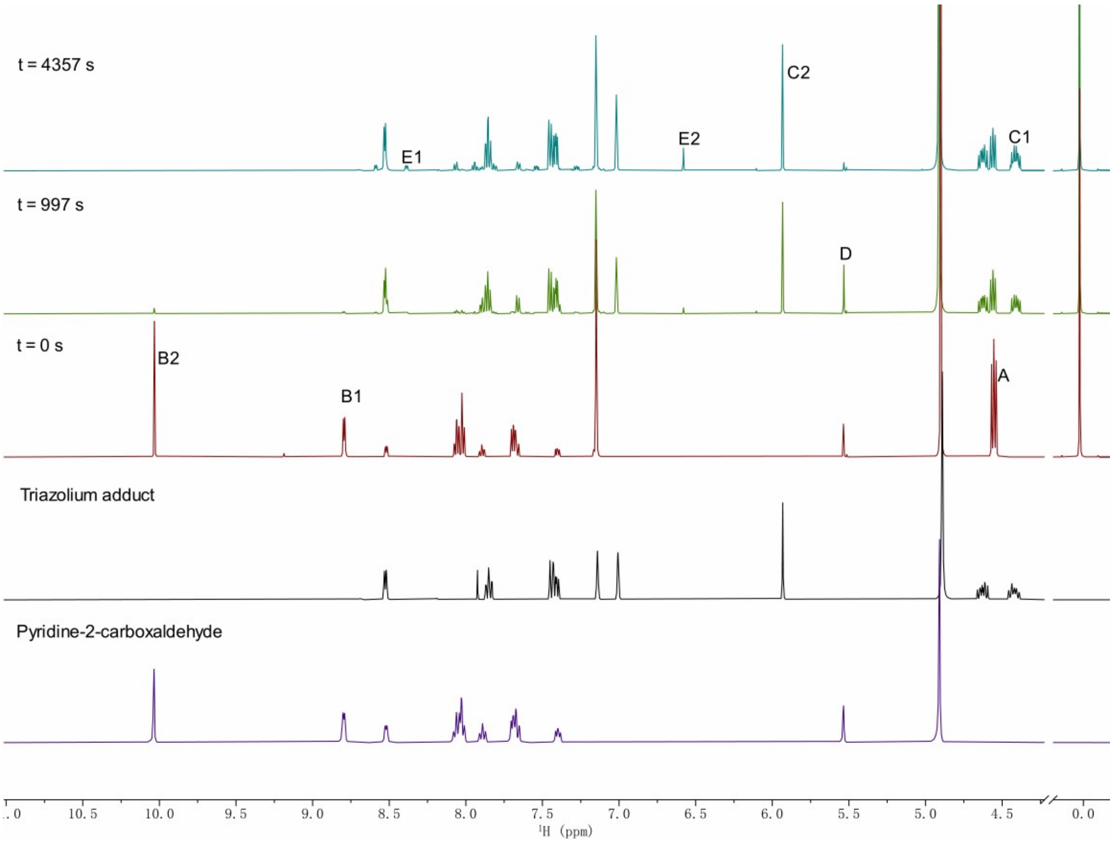

Figure S24. Representative ^{1}H NMR spectra (400 MHz) for reaction of pyridine-2-carboxaldehyde **34** (0.02 M) with *N*-Mes NHC precursor **42** (0.02 M) under a triethylamine buffer (NEt_3:NEt_3·HCl, 2:1, 0.09 M) in CD_3OD at 25 °C. A = NHC precursor NCH_2, B1 =Ar*H*CHO, B2 =ArHC*H*O, C1 = Adduct NC$H_A$$H_B$, C2 =Adduct C(α)*H*, D = Hemiacetal C*H*, E1 = Benzoin Ar*H*, E2 = Benzoin C*H*.

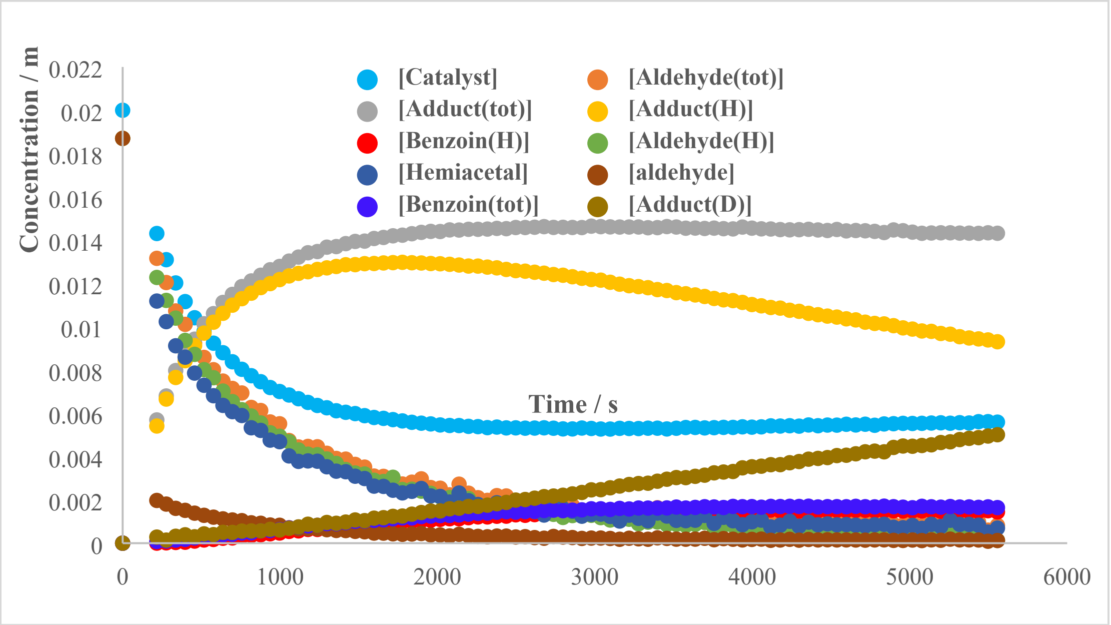

Figure S25. Concentration profile for the self-condensation of pyridine-2-carboxaldehyde **34** (0.02 M) with *N*-Mes NHC precursor **42** (0.02 M) under a triethylamine buffer (NEt_3:NEt_3·HCl, 2:1, 0.09 M) in CD_3OD at 25 °C.

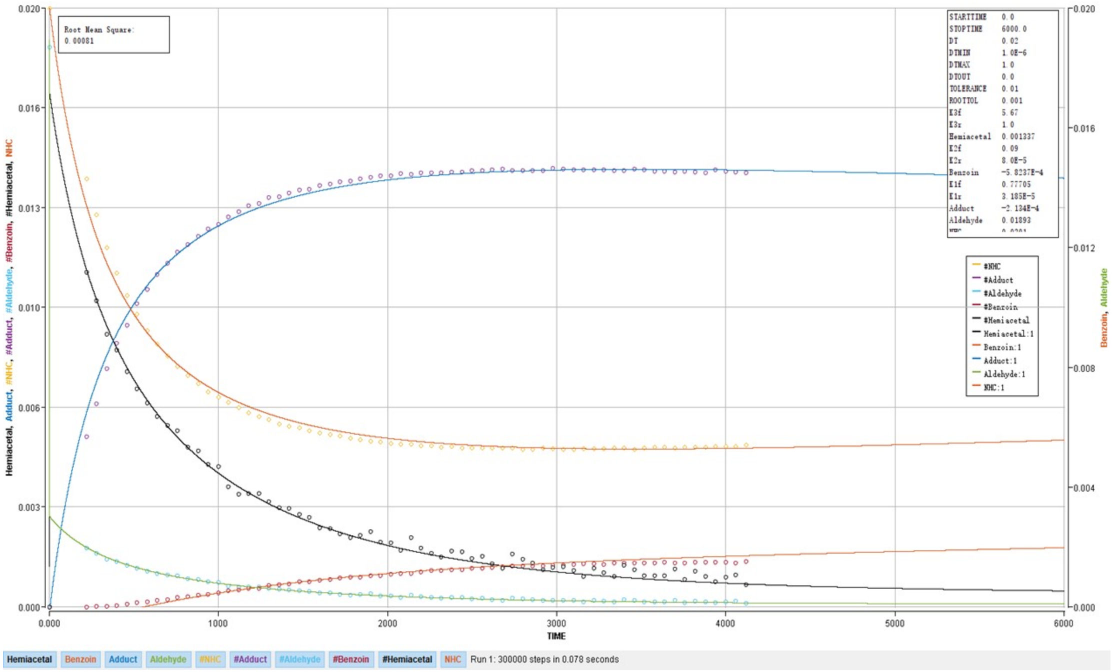

Figure S26. Global fitting profile for the reaction of pyridine-2-carboxaldehyde **34** (0.02 M) with *N*-Mes NHC precursor **42** (0.02 M) under a triethylamine buffer (NEt_3:NEt_3·HCl, 2:1, 0.09 M) in CD_3OD at 25 °C. Open circles show the experimental data, with the solid line representing the fit to the kinetic model. Fitting data from t = 0 to t = 6000 s from Figure S25.

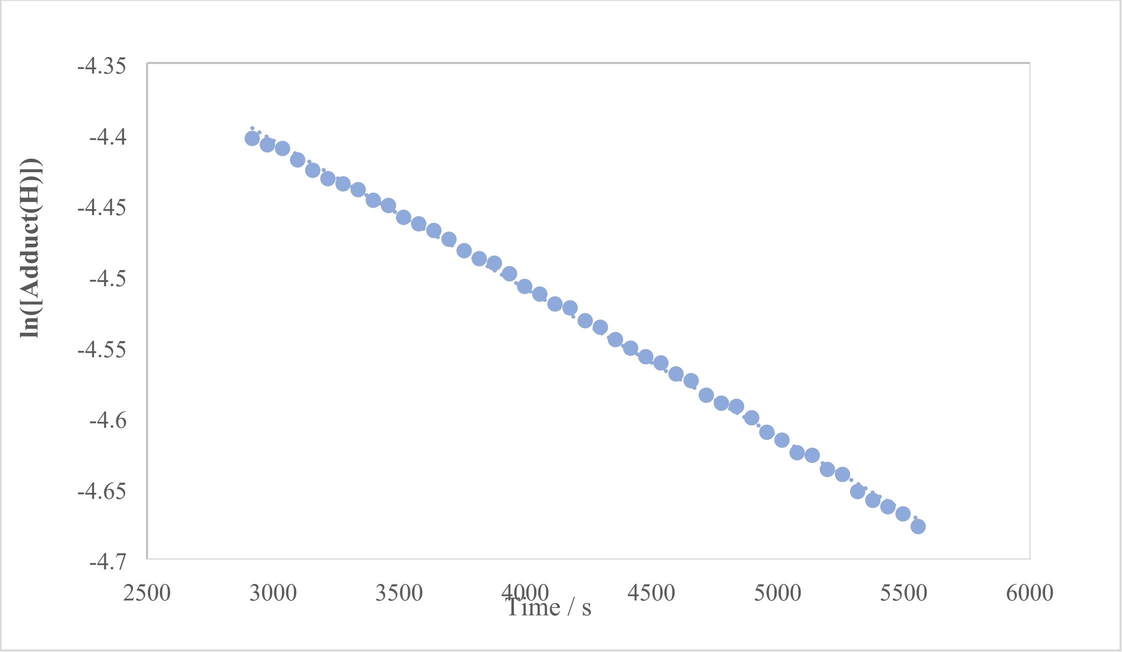

Figure S27. Semilogarithmic plots of [Adduct (H)] against time for the reactions of pyridine-2-carboxaldehyde **34** (0.02 M) with *N*-Mes NHC precursor **42** (0.02 M) under a triethylamine buffer (NEt_3:NEt_3·HCl, 2:1, 0.09 M) in CD_3OD at 25 °C.

Table 1 Entry 7

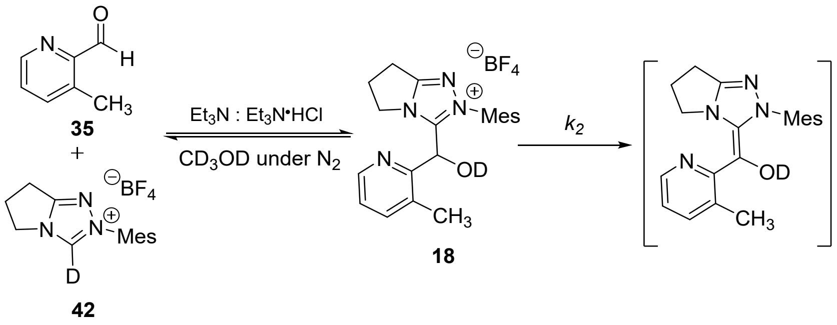

The reaction of aldehyde **35** and triazolium precatalyst **42** was monitored using ^{1}H NMR spectra, with representative NMR spectra over the course of the experiment given in Figure S28.

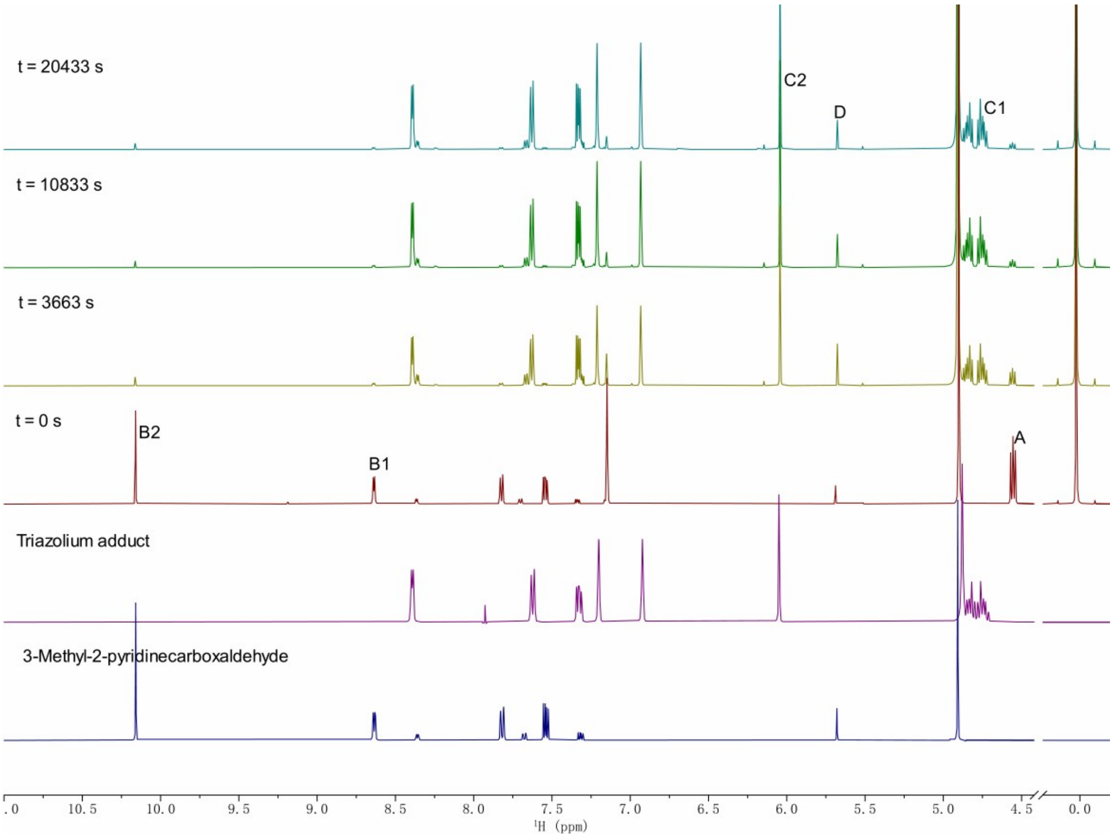

Figure S28. Representative ^{1}H NMR spectra (400 MHz) for reaction of 3-methyl-2-pyridinecarboxaldehyde **35** (0.02 M) with *N*-Mes NHC precursor **42** (0.02 M) under a triethylamine buffer (NEt_3:NEt_3·HCl, 2:1, 0.09 M) in CD_3OD at 25 °C. A = NHC precursor NCH_2, B1 =Ar*H*CHO, B2 =ArHC*H*O, C1 = Adduct NCH_AH_B, C2 =Adduct C(α)*H*, D = Hemiacetal C*H*.

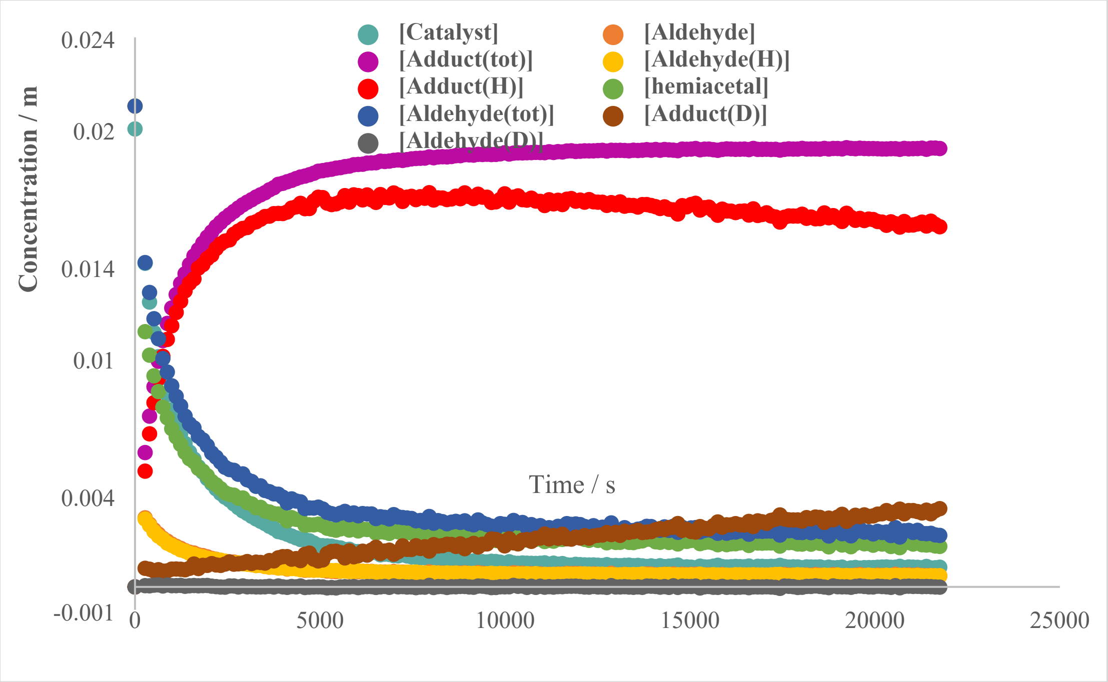

Figure S29. Concentration profile for the self-condensation of 3-methyl-2-pyridinecarboxaldehyde **35** (0.02 M) with *N*-Mes NHC precursor **42** (0.02 M) under a triethylamine buffer (NEt_3:NEt_3·HCl, 2:1, 0.09 M) in CD_3OD at 25 °C.

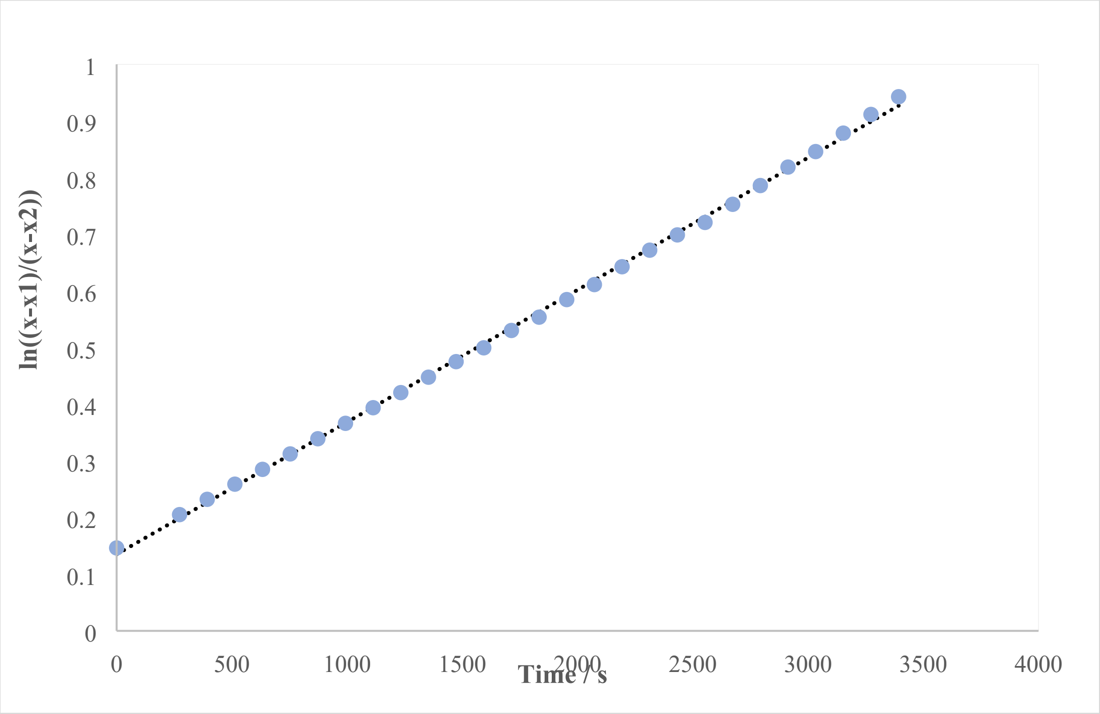

Figure S30. Semilogarithmic plots of $(x-x_1)/(x-x_2)$ against time, obtained from the reaction of 3-methyl-2-pyridinecarboxaldehyde **35** (0.02 M) with *N*-Mes NHC precursor **42** (0.02 M) under a triethylamine buffer (NEt_3:NEt_3·HCl, 2:1, 0.09 M) in CD_3OD at 25 °C.

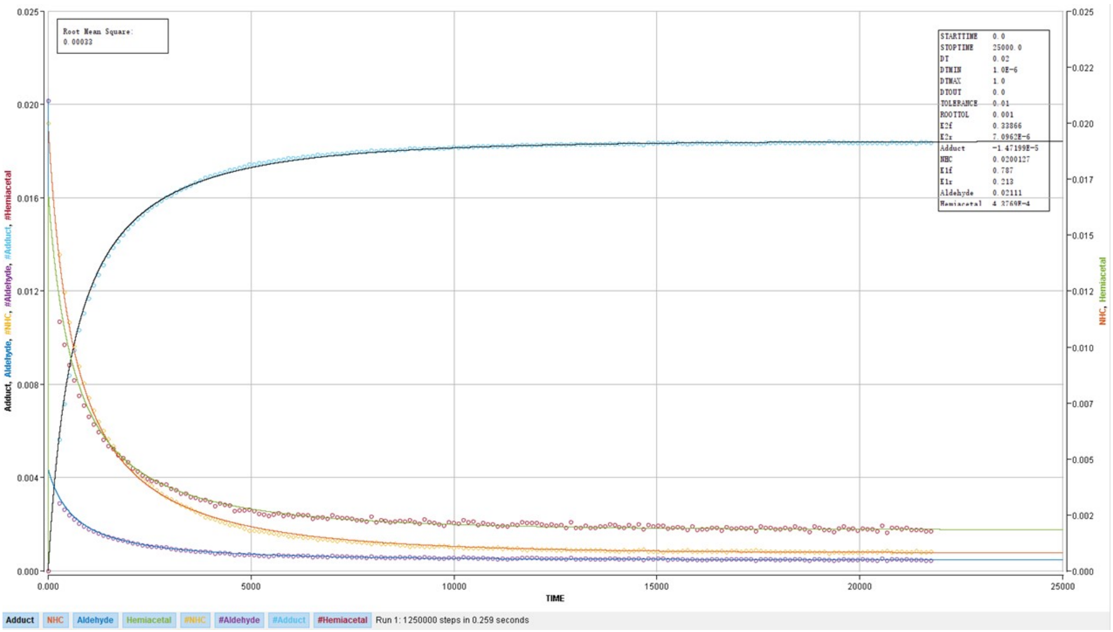

Figure S31. Global fitting profile for the reaction of 3-methyl-2-pyridinecarboxaldehyde **35** (0.02 M) with *N*-Mes NHC precursor **42** (0.02 M) under a triethylamine buffer (NEt_3:NEt_3·HCl, 2:1, 0.09 M) in CD_3OD at 25 °C. Open circles show the experimental data, with the solid line representing the fit to the kinetic model. Fitting data from t = 0 to t = 25000 s from Figure S29.

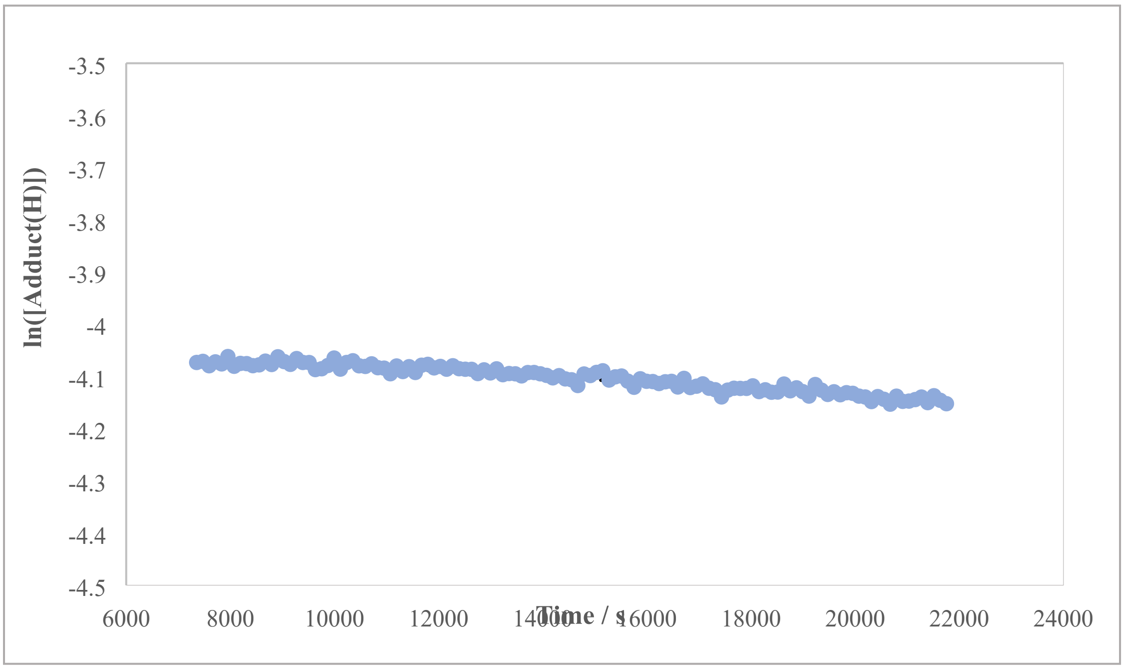

Figure S32. Semilogarithmic plots of [Adduct (H)] against time for the reactions of 3-methyl-2-pyridinecarboxaldehyde **35** (0.02 M) with *N*-Mes NHC precursor **42** (0.02 M) under a triethylamine buffer (NEt_3:NEt_3·HCl, 2:1, 0.09 M) in CD_3OD at 25 °C.

Table 1 Entry 8

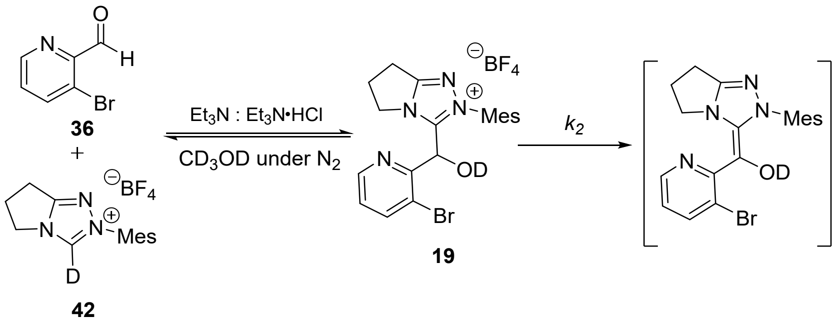

The reaction of aldehyde **36** and triazolium precatalyst **42** was monitored using ^{1}H NMR spectra, with representative NMR spectra over the course of the experiment given in Figure S33.

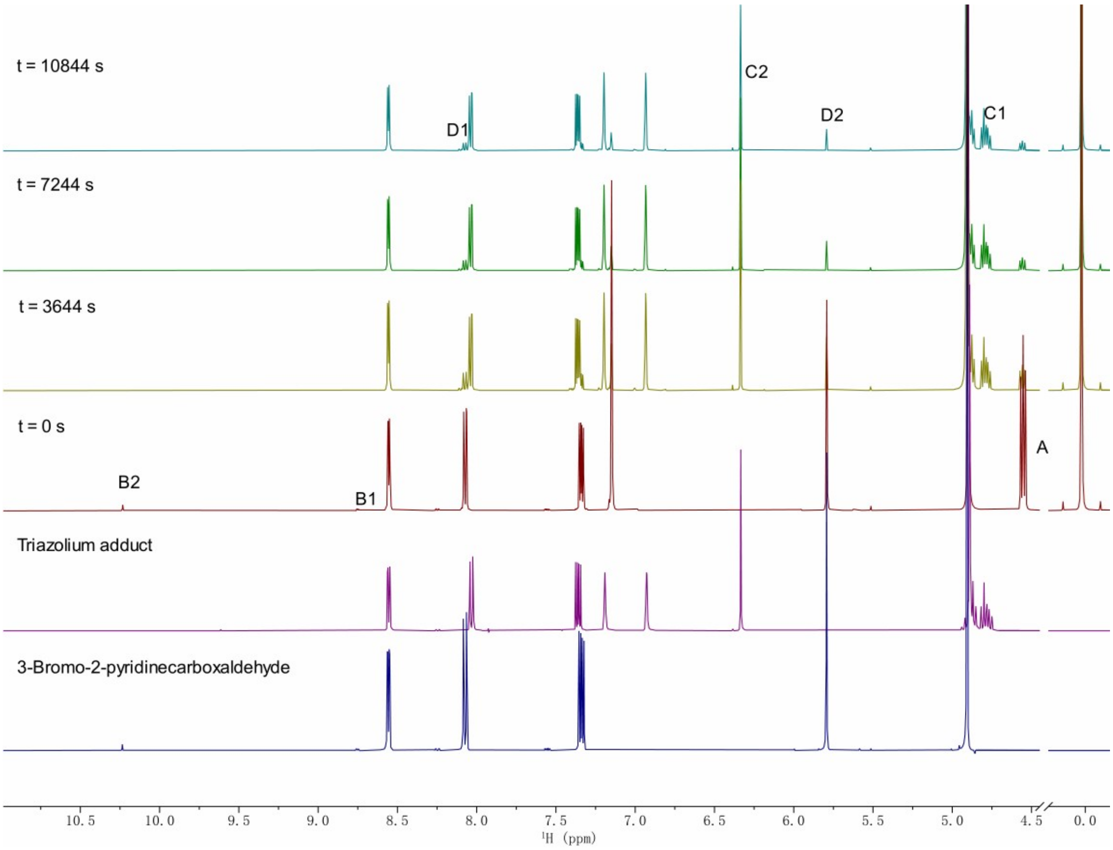

Figure S33. Representative ^{1}H NMR spectra (400 MHz) for reaction of 3-bromo-2-pyridinecarboxaldehyde **36** (0.02 M) with *N*-Mes NHC precursor **42** (0.02 M) under a triethylamine buffer (NEt_3:NEt_3·HCl, 2:1, 0.09 M) in CD_3OD at 25 °C. A = NHC precursor NCH_2, B1 =Ar*H*CHO, B2 =ArHC*H*O, C1 = Adduct NCH_AH$_B$, C2 =Adduct C(α)*H,* D1 = Hemiacetal Ar*H*, D2 = Hemiacetal C*H*.

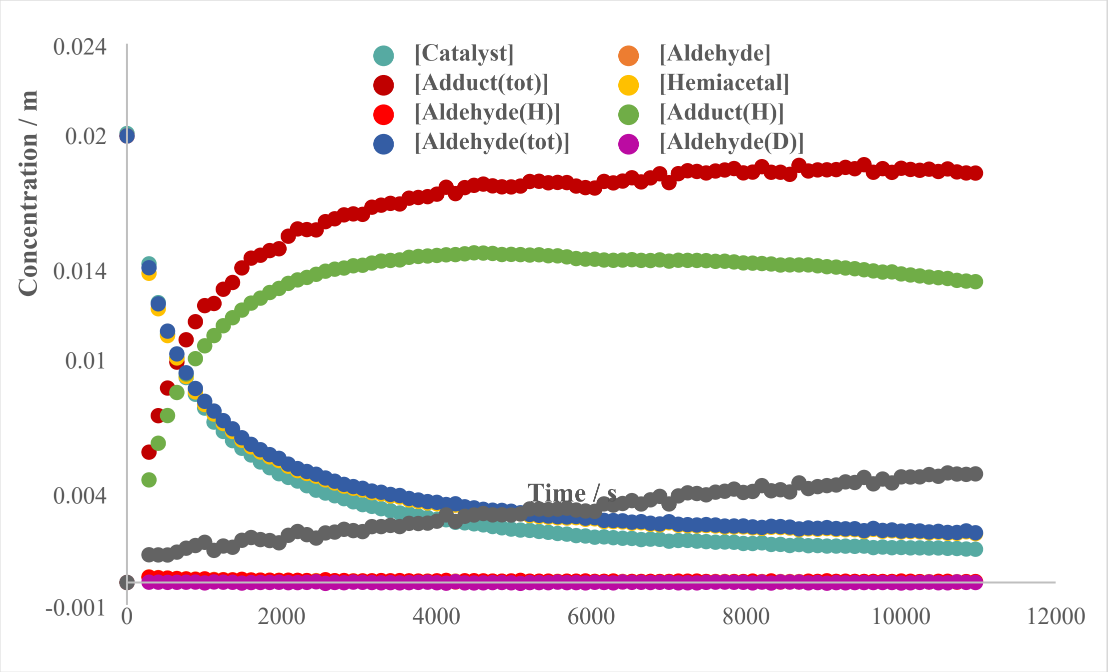

Figure S34. Concentration profile for the self-condensation of 3-bromo-2-pyridinecarboxaldehyde **36** (0.02 M) with *N*-Mes NHC precursor **42** (0.02 M) under a triethylamine buffer (NEt_3:NEt_3·HCl, 2:1, 0.09 M) in CD_3OD at 25 °C.

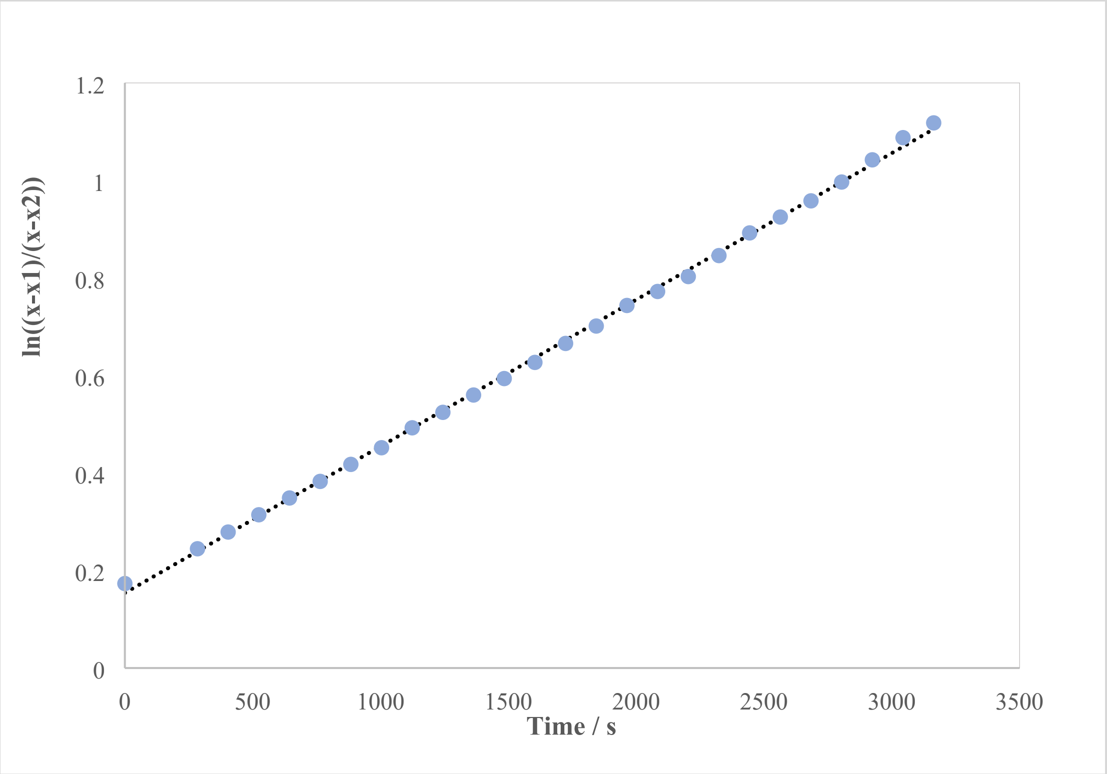

Figure S35. Semilogarithmic plots of $(x-x_1)/(x-x_2)$ against time, obtained from the reaction of 3-bromo-2-pyridinecarboxaldehyde **36** (0.02 M) with *N*-Mes NHC precursor **42** (0.02 M) under a triethylamine buffer (NEt_3:NEt_3·HCl, 2:1, 0.09 M) in CD_3OD at 25 °C.

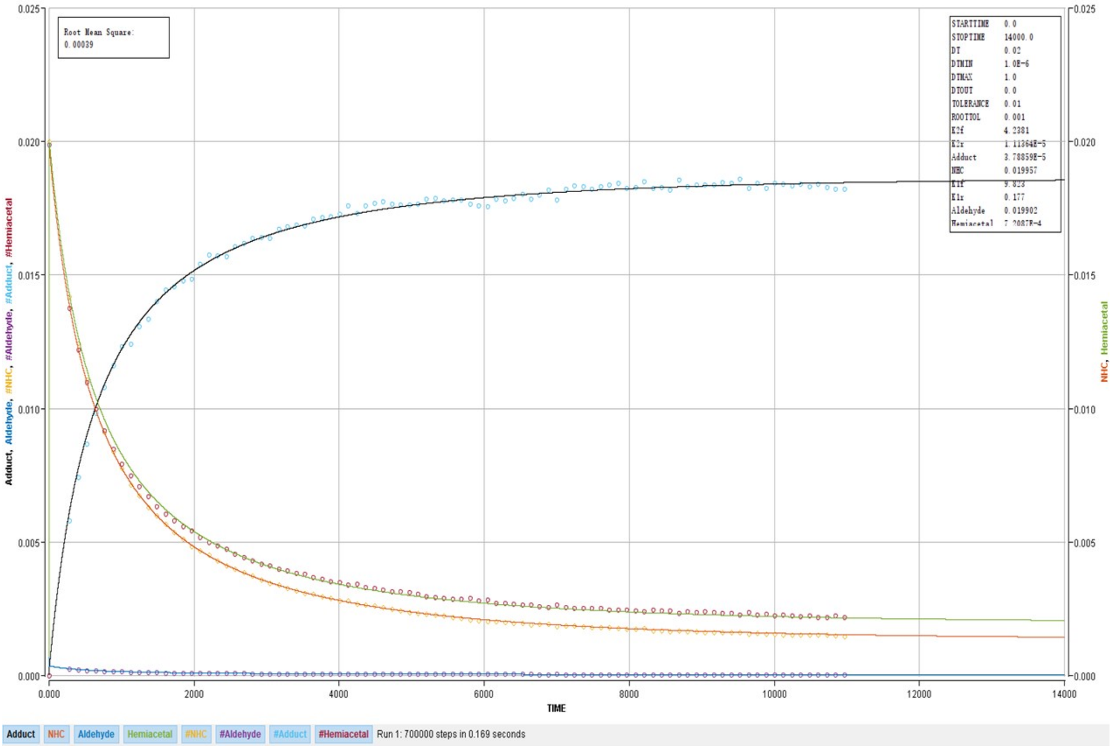

Figure S36. Global fitting profile for the reaction of 3-bromo-2-pyridinecarboxaldehyde **36** (0.02 M) with *N*-Mes NHC precursor **42** (0.02 M) under a triethylamine buffer (NEt_3:NEt_3·HCl, 2:1, 0.09 M) in CD_3OD at 25 °C. Open circles show the experimental data, with the solid line representing the fit to the kinetic model. Fitting data from t = 0 to t = 14000 s from Figure S34.

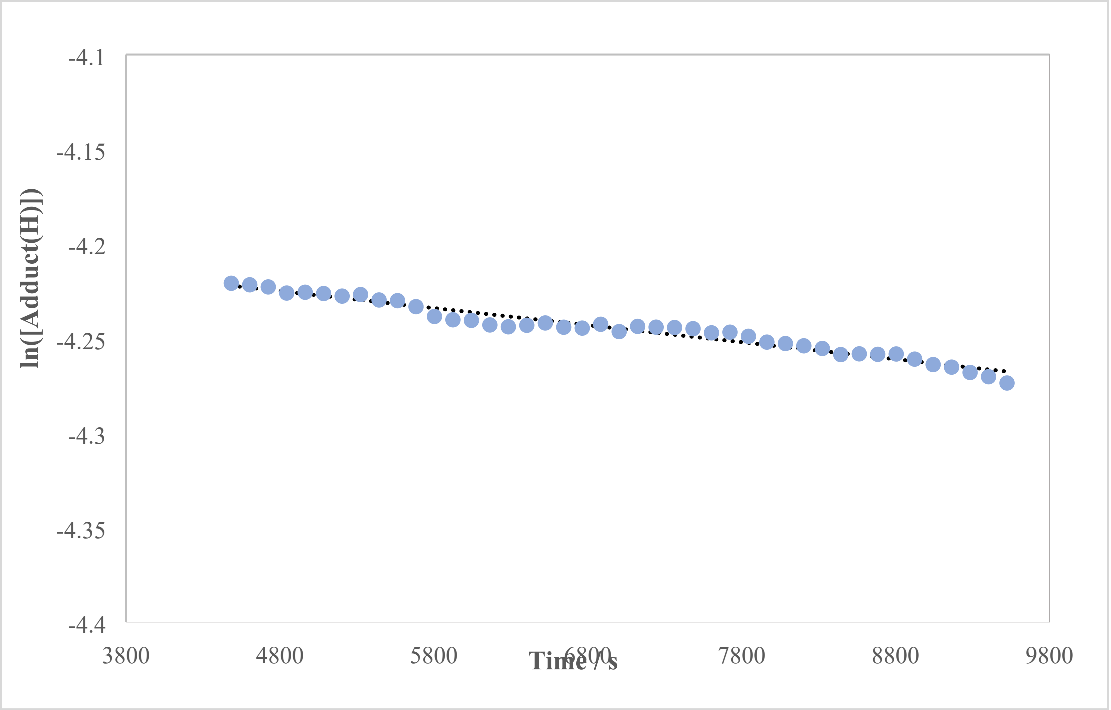

Figure S37. Semilogarithmic plots of [Adduct (H)] against time for the reactions of 3-bromo-2-pyridinecarboxaldehyde **36** (0.02 M) with *N*-Mes NHC precursor **42** (0.02 M) under a triethylamine buffer (NEt_3:NEt_3·HCl, 2:1, 0.09 M) in CD_3OD at 25 °C.

Table 1 Entry 9

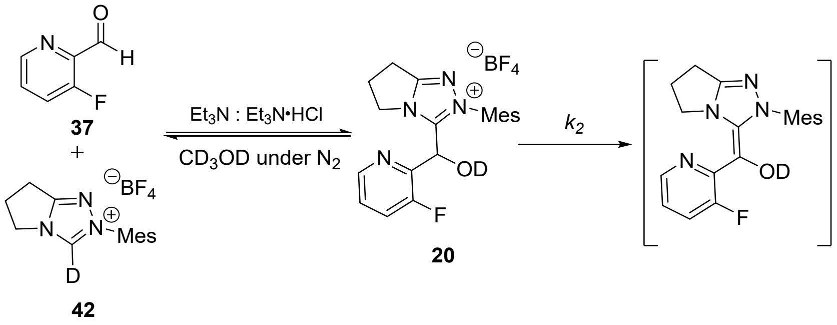

The reaction of aldehyde **37** and triazolium precatalyst **42** was monitored using ^{1}H NMR spectra, with representative NMR spectra over the course of the experiment given in Figure S38.

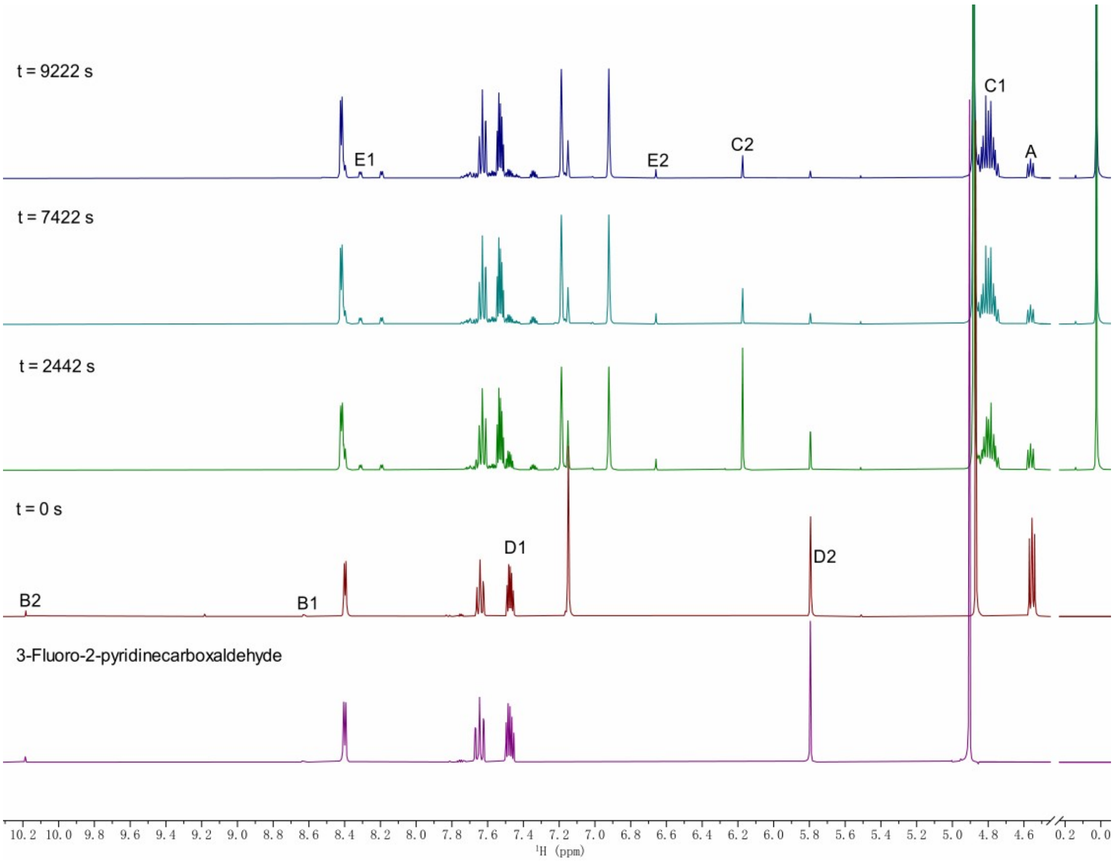

Figure S38. Representative ^{1}H NMR spectra (400 MHz) for reaction of 3-fluoro-2-pyridinecarboxaldehyde **37** (0.02 M) with *N*-Mes NHC precursor **42** (0.02 M) under a triethylamine buffer (NEt_3:NEt_3·HCl, 2:1, 0.09 M) in CD_3OD at 25 °C. A = NHC precursor NCH_2, B1 =Ar*H*CHO, B2 =ArHC*H*O, C1 = Adduct NCH_2, C2 =Adduct C(α)*H*, D1 = Hemiacetal Ar*H*, D2 = Hemiacetal C*H*, E1 = Benzoin Ar*H*, E2 = Benzoin C*H*.

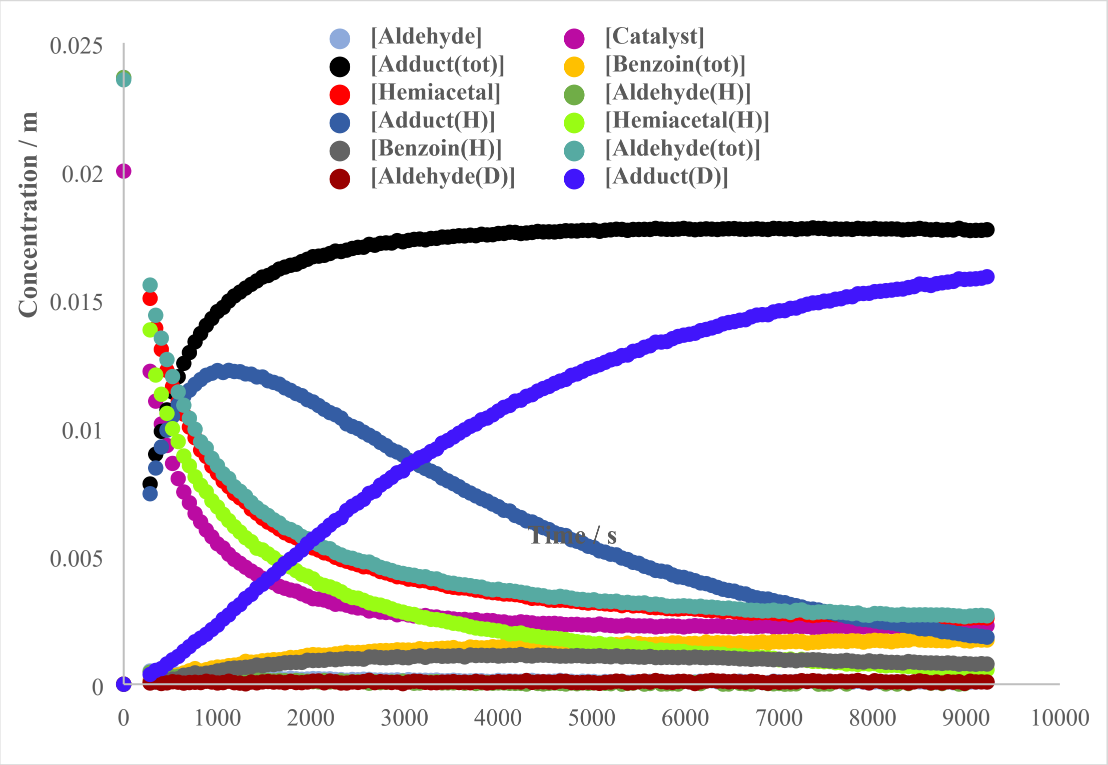

Figure S39. Concentration profile for the self-condensation of 3-fluoro-2-pyridinecarboxaldehyde **37** (0.02 M) with *N*-Mes NHC precursor **42** (0.02 M) under a triethylamine buffer (NEt_3:NEt_3·HCl, 2:1, 0.09 M) in CD_3OD at 25 °C.

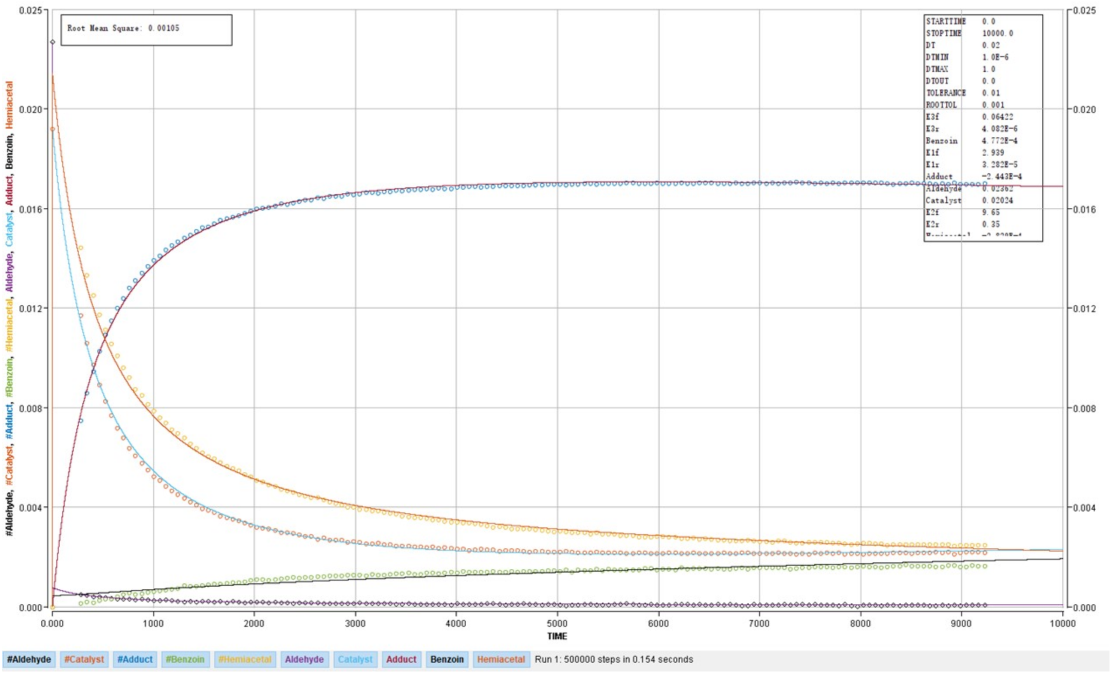

Figure S40. Global fitting profile for the reaction of 3-fluoro-2-pyridinecarboxaldehyde **37** (0.02 M) with *N*-Mes NHC precursor **42** (0.02 M) under a triethylamine buffer (NEt_3:NEt_3·HCl, 2:1, 0.09 M) in CD_3OD at 25 °C. Open circles show the experimental data, with the solid line representing the fit to the kinetic model. Fitting data from t = 0 to t = 10000 s from Figure S39.

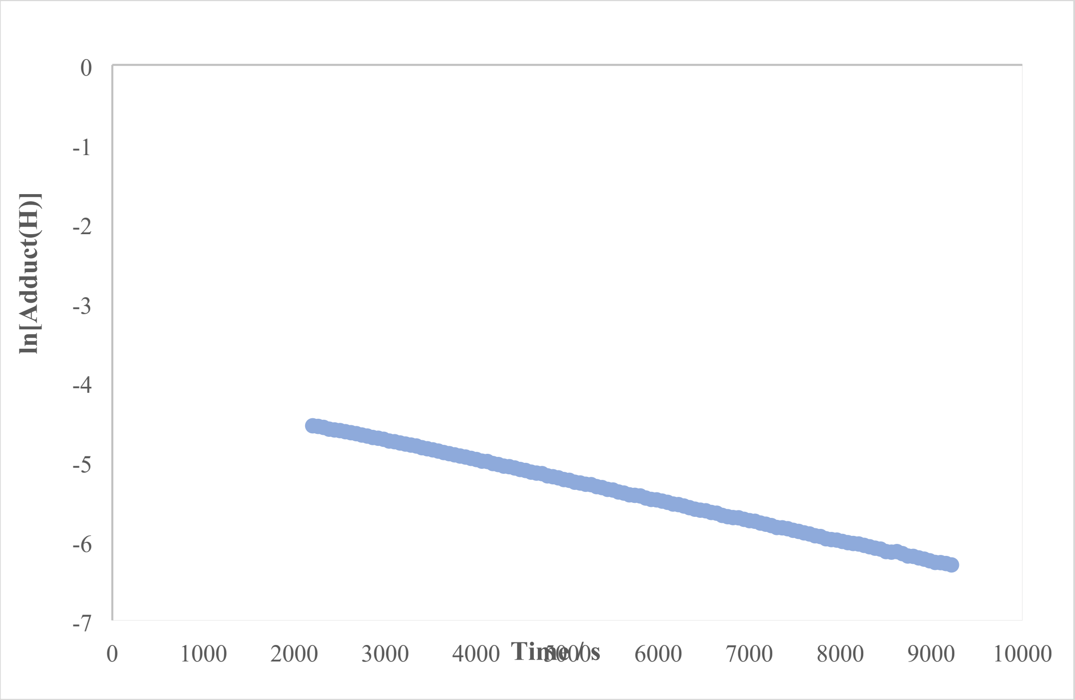

Figure S41. Semilogarithmic plots of [Adduct (H)] against time for the reactions of 3-fluoro-2-pyridinecarboxaldehyde **37** (0.02 M) with *N*-Mes NHC precursor **42** (0.02 M) under a triethylamine buffer (NEt_3:NEt_3·HCl, 2:1, 0.09 M) in CD_3OD at 25 °C.

Table 1 Entry 10

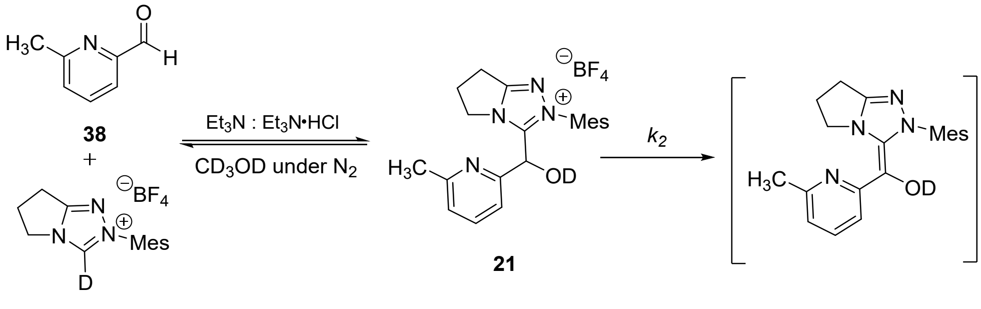

The reaction of aldehyde **38** and triazolium precatalyst **42** was monitored using ^{1}H NMR spectra, with representative NMR spectra over the course of the experiment given in Figure S42.

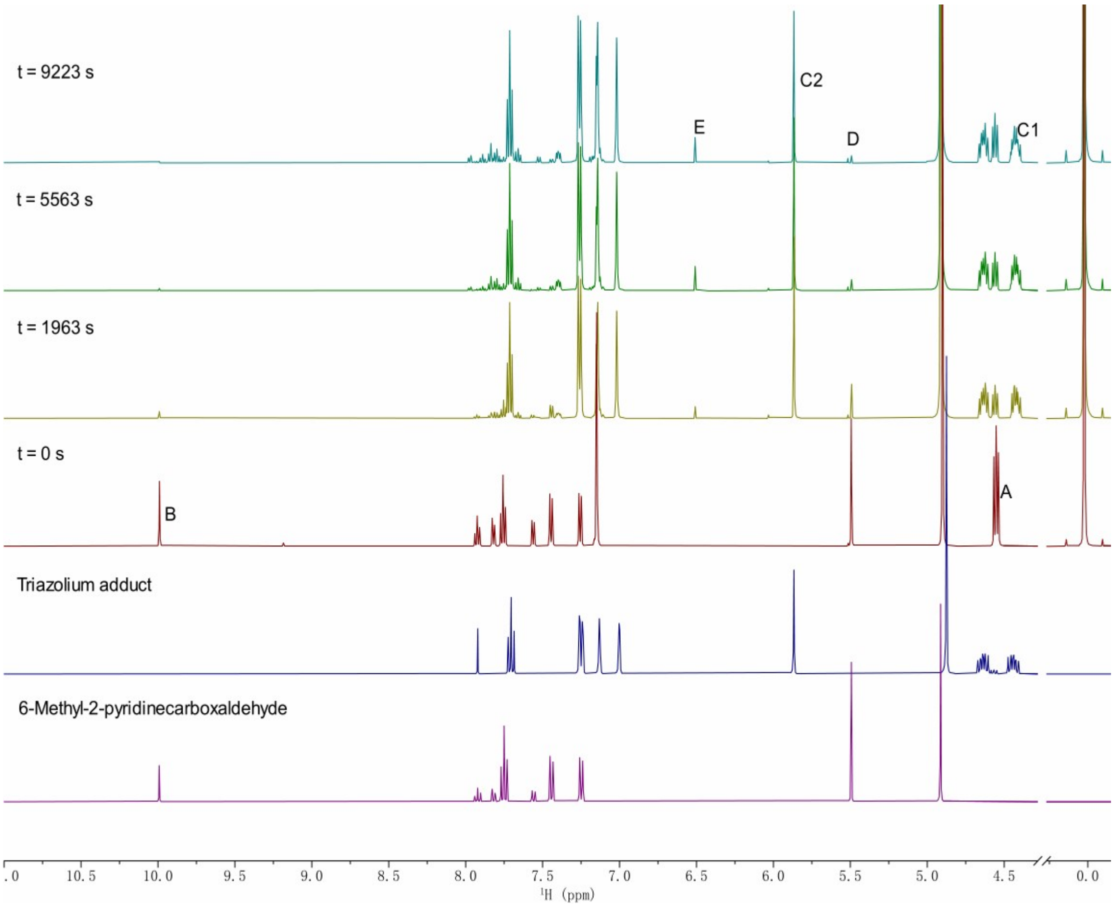

Figure S42. Representative ^{1}H NMR spectra (400 MHz) for reaction of 6-methyl-2-pyridinecarboxaldehyde **38** (0.02 M) with *N*-Mes NHC precursor **42** (0.02 M) under a triethylamine buffer (NEt_3:NEt_3·HCl, 2:1, 0.09 M) in CD_3OD at 25 °C. A = NHC precursor NCH_2, B =ArHC*H*O (The integral of aldehydic proton B is used to calculate the approximate [Aldehyde] as all the other protons of the aldehyde are overlapped with other components in the experiment), C1 = Adduct NC$H_A$$H_B$, C2 =Adduct C(α)*H,* D = Hemiacetal C*H*, E1 = Benzoin C*H*.

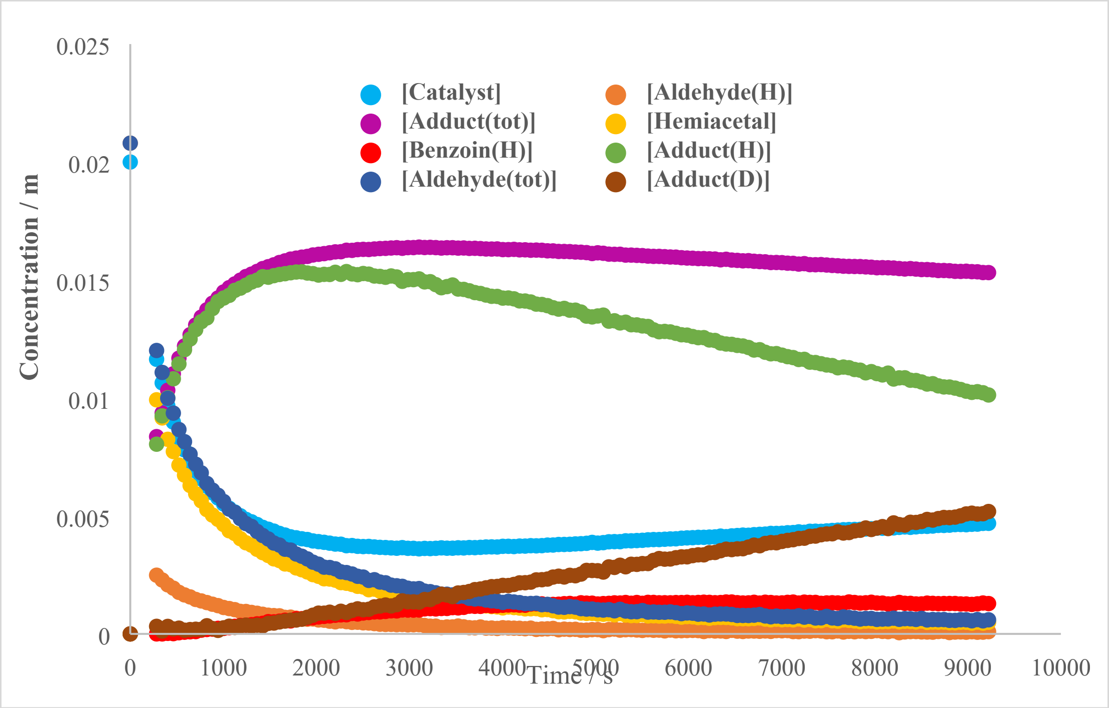

Figure S43. Concentration profile for the self-condensation of 6-methyl-2-pyridinecarboxaldehyde **38** (0.02 M) with *N*-Mes NHC precursor **42** (0.02 M) under a triethylamine buffer (NEt_3:NEt_3·HCl, 2:1, 0.09 M) in CD_3OD at 25 °C.

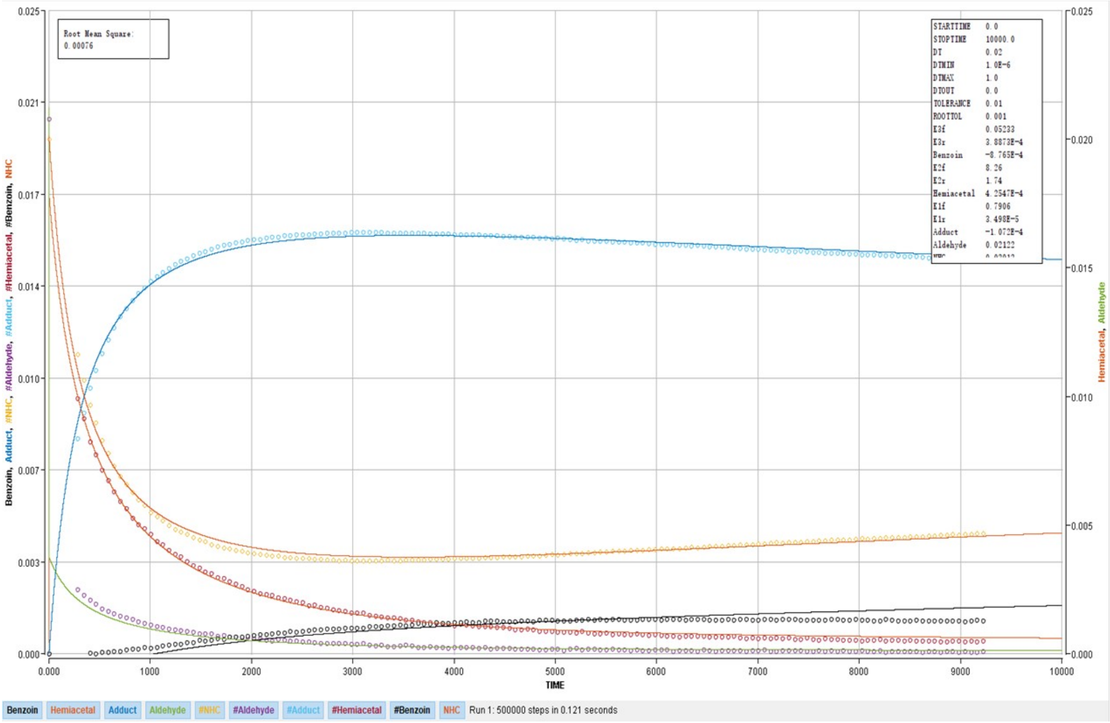

Figure S44. Global fitting profile for the reaction of 6-methyl-2-pyridinecarboxaldehyde **38** (0.02 M) with *N*-Mes NHC precursor **42** (0.02 M) under a triethylamine buffer (NEt_3:NEt_3·HCl, 2:1, 0.09 M) in CD_3OD at 25 °C. Open circles show the experimental data, with the solid line representing the fit to the kinetic model. Fitting data from t = 0 to t = 10000 s from Figure S43.

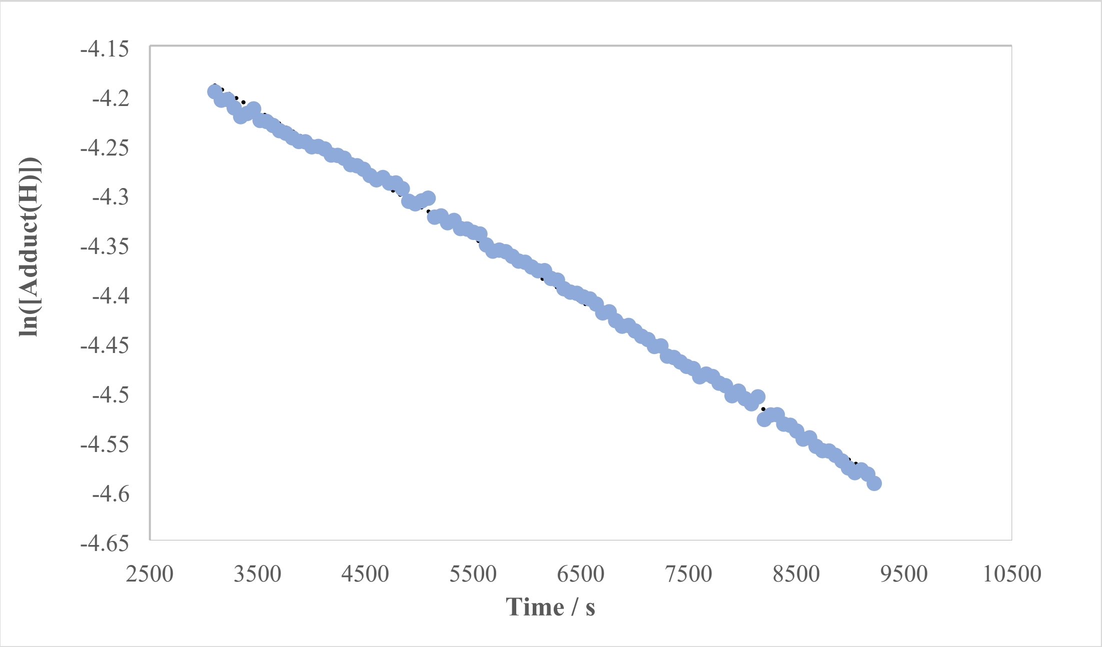

Figure S45. Semilogarithmic plots of [Adduct (H)] against time for the reactions of 6-methyl-2-pyridinecarboxaldehyde **38** (0.02 M) with *N*-Mes NHC precursor **42** (0.02 M) under a triethylamine buffer (NEt_3:NEt_3·HCl, 2:1, 0.09 M) in CD_3OD at 25 °C.

Table 1 Entry 11

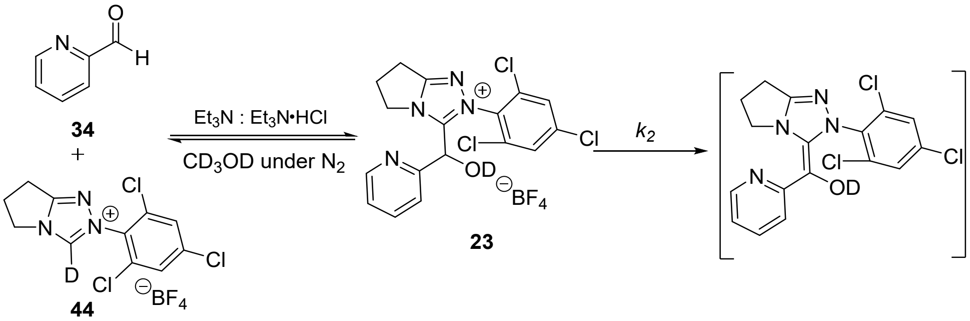

The reaction of aldehyde **34** and triazolium precatalyst **44** was monitored using ^{1}H NMR spectra, with representative NMR spectra over the course of the experiment given in Figure S42.

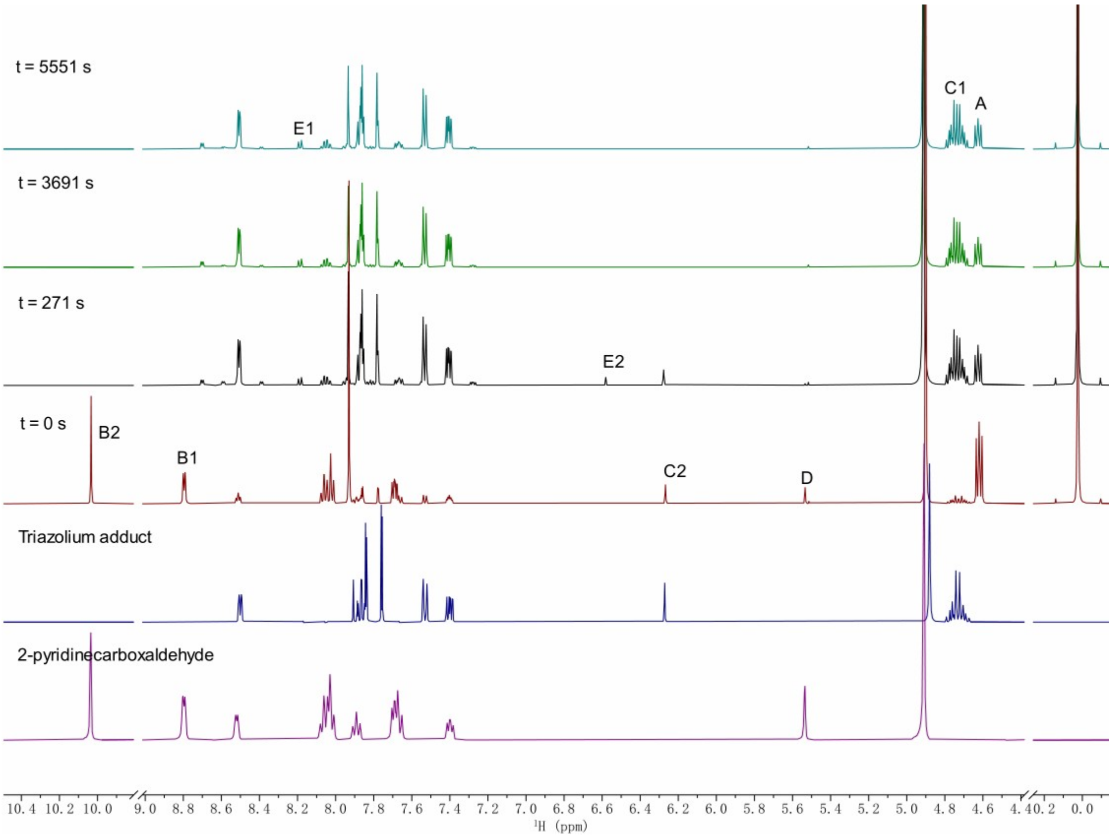

Figure S46. Representative ^{1}H NMR spectra (400 MHz) for reaction of 2-pyridinecarboxaldehyde **34** (0.02 M) with *N*-$C_6H_2Cl_3$ NHC precursor **44** (0.02 M) under a triethylamine buffer (NEt_3:NEt_3·HCl, 2:1, 0.09 M) in CD_3OD at 25 °C. A = NHC precursor NC*H_2*, B1 =Ar*H*CHO, B2 =ArHC*H*O, C1 = Adduct NC*H_2*, C2 =Adduct C(α)*H,* D = Hemiacetal C*H*, E1 = Benzoin Ar*H*, E2 = Benzoin C*H*.

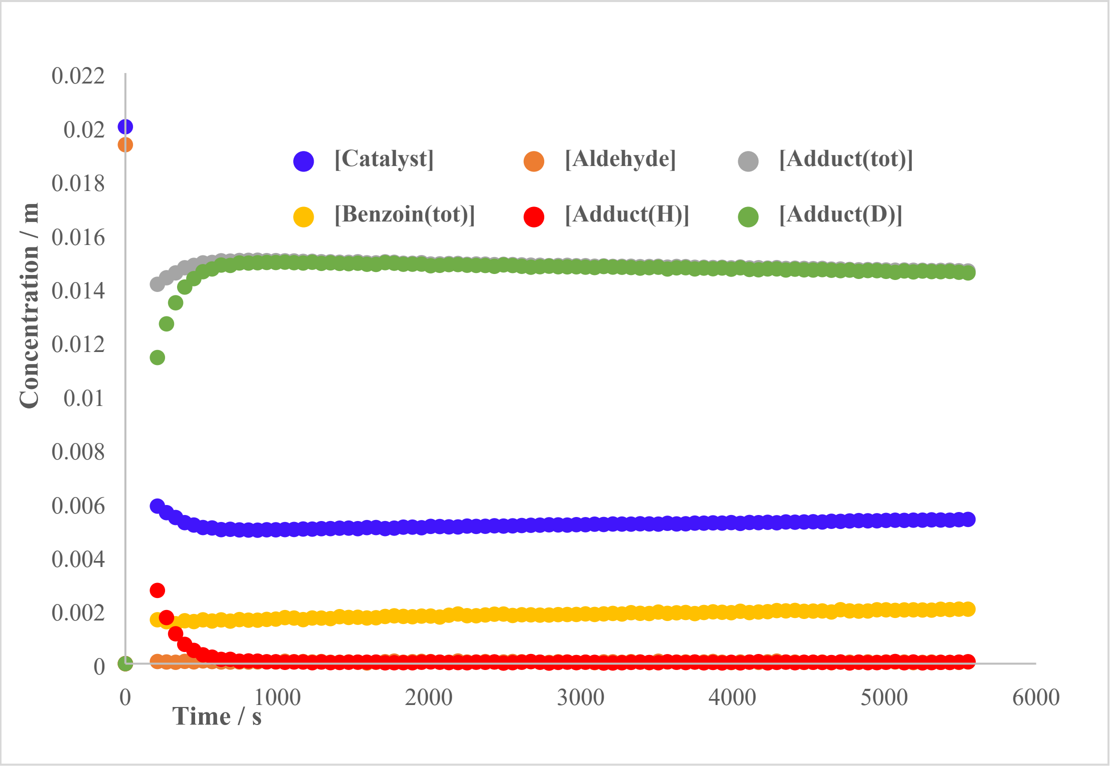

Figure S47. Concentration profile for the self-condensation of 2-pyridinecarboxaldehyde **34** (0.02 M) with *N*-$C_6H_2Cl_3$ NHC precursor **44** (0.02 M) under a triethylamine buffer (NEt_3:NEt_3·HCl, 2:1, 0.09 M) in CD_3OD at 25 °C.

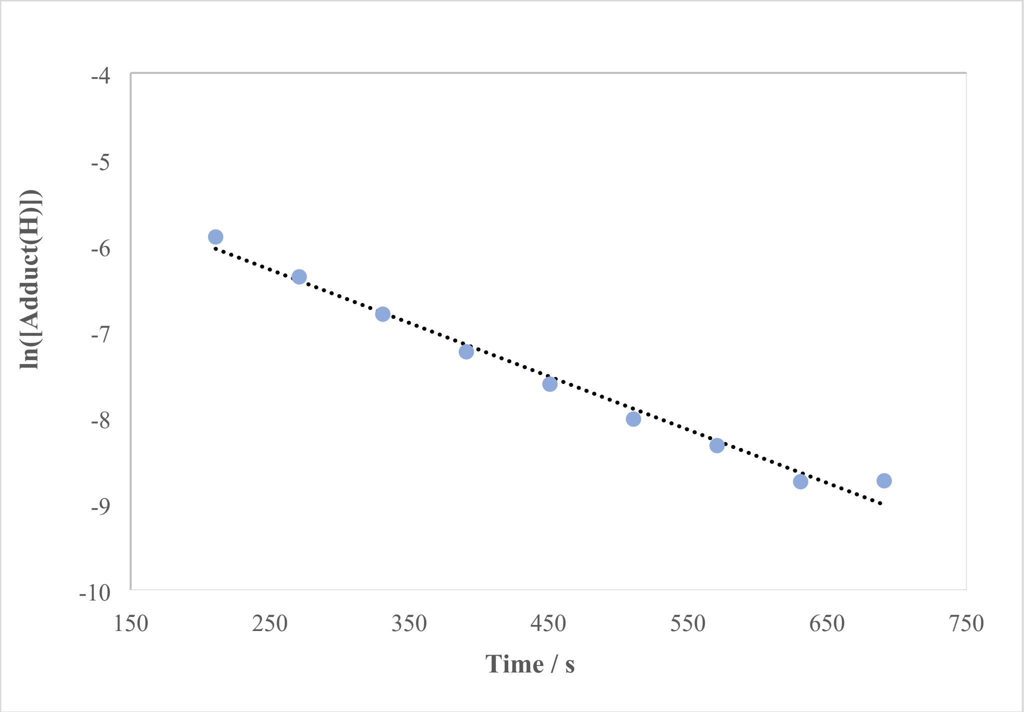

Figure S48. Semilogarithmic plots of [Adduct (H)] against time for the reactions of 2-pyridinecarboxaldehyde **34** (0.02 M) with *N*-$C_6H_2Cl_3$ NHC precursor **44** (0.02 M) under a triethylamine buffer (NEt_3:NEt_3·HCl, 2:1, 0.09 M) in CD_3OD at 25 °C.

Table 1 Entry 12

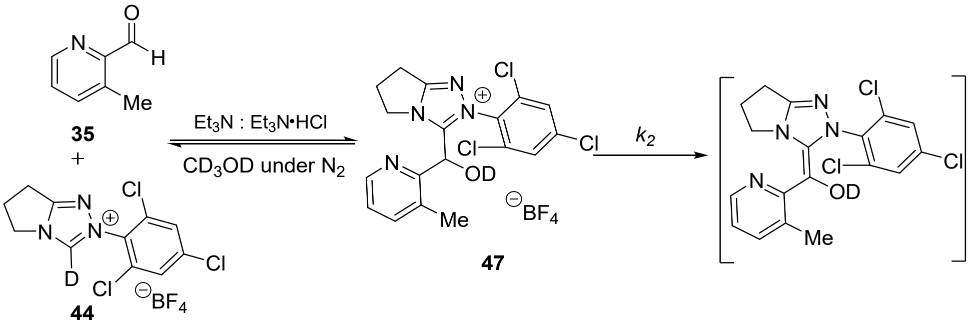

The reaction of aldehyde **35** and triazolium precatalyst **44** was monitored using ^{1}H NMR spectra, with representative NMR spectra over the course of the experiment given in Figure S42.

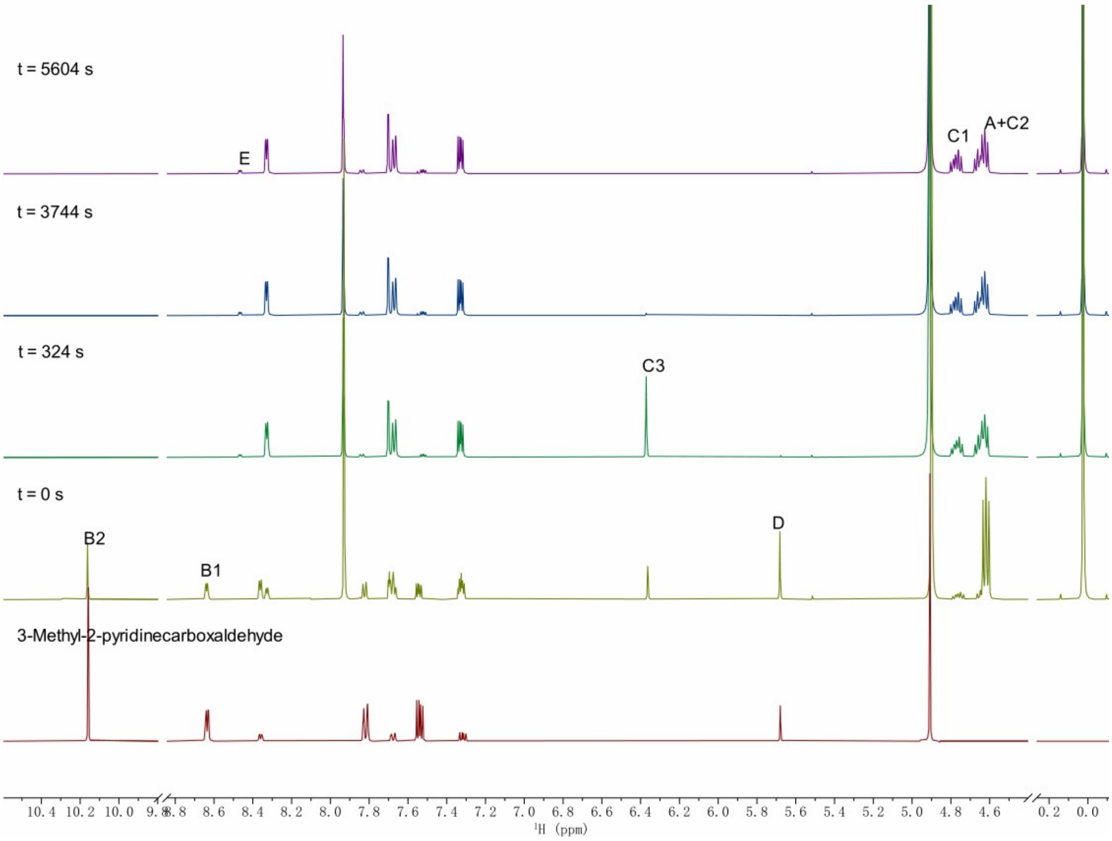

Figure S49. Representative ^{1}H NMR spectra (400 MHz) for reaction of 3-methyl-2-pyridinecarboxaldehyde **35** (0.02 M) with *N*-$C_6H_2Cl_3$ NHC precursor **44** (0.02 M) under a triethylamine buffer (NEt_3:NEt_3·HCl, 2:1, 0.09 M) in CD_3OD at 25 °C. A = NHC precursor NCH_2, B1 =Ar*H*CHO, B2 =ArHC*H*O, C1 = Adduct NC$H_A$$H_B$, C2 = Adduct NC$H_A$$H_B$, C3 =Adduct C(α)*H*, D = Hemiacetal C*H*, E1 = Benzoin Ar*H*.

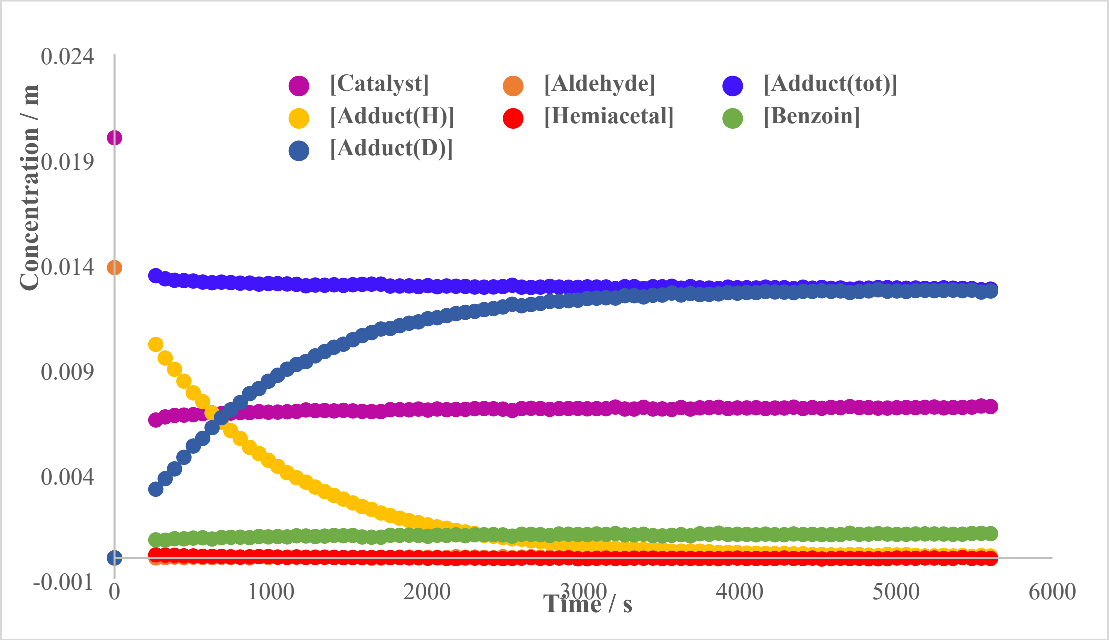

Figure S50. Concentration profile for the self-condensation of 3-methyl-2-pyridinecarboxaldehyde

35 (0.02 M) with *N*-$C_6H_2Cl_3$ NHC precursor **44** (0.02 M) under a triethylamine buffer (NEt_3:NEt_3·HCl, 2:1, 0.09 M) in CD_3OD at 25 °C.

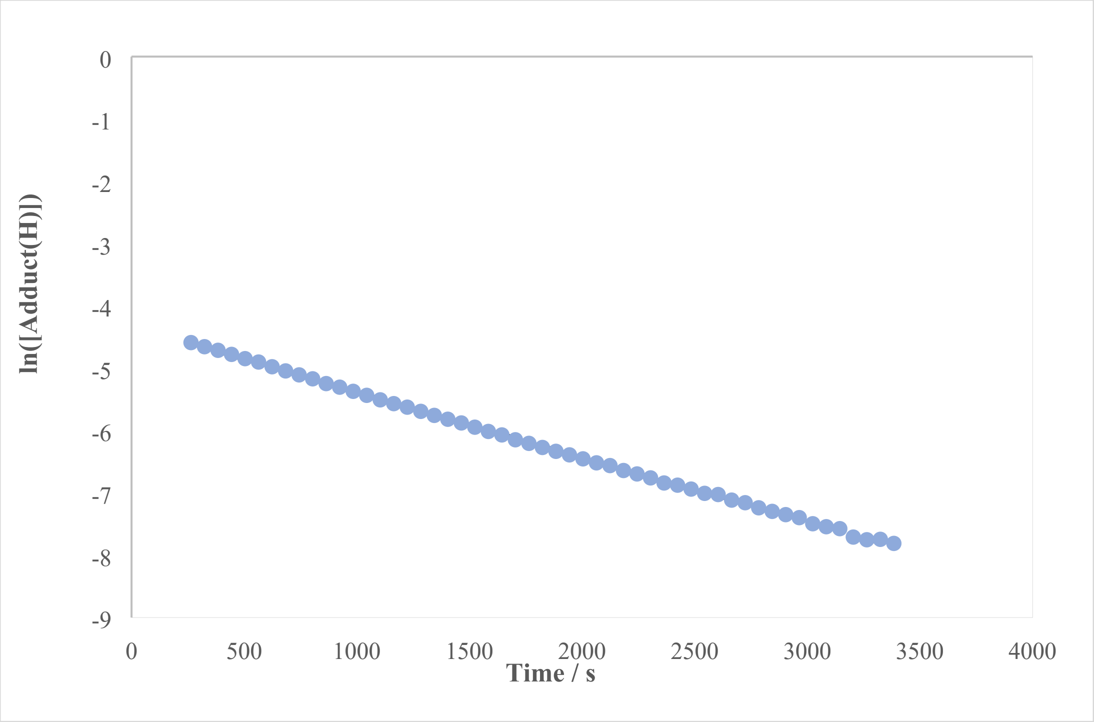

Figure S51. Semilogarithmic plots of [Adduct (H)] against time for the reactions of 3-methyl-2-pyridinecarboxaldehyde **35** (0.02 M) with *N*-$C_6H_2Cl_3$ NHC precursor **44** (0.02 M) under a triethylamine buffer (NEt_3:NEt_3·HCl, 2:1, 0.09 M) in CD_3OD at 25 °C.

Table 1 Entry 13

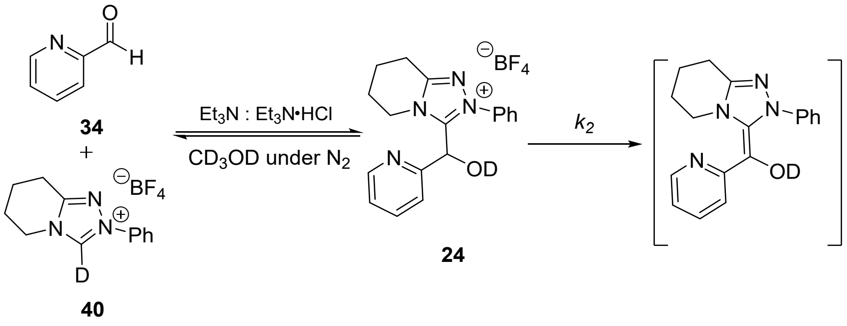

The reaction of aldehyde **34** and triazolium precatalyst **40** was monitored using ^{1}H NMR spectra, with representative NMR spectra over the course of the experiment given in Figure S52.

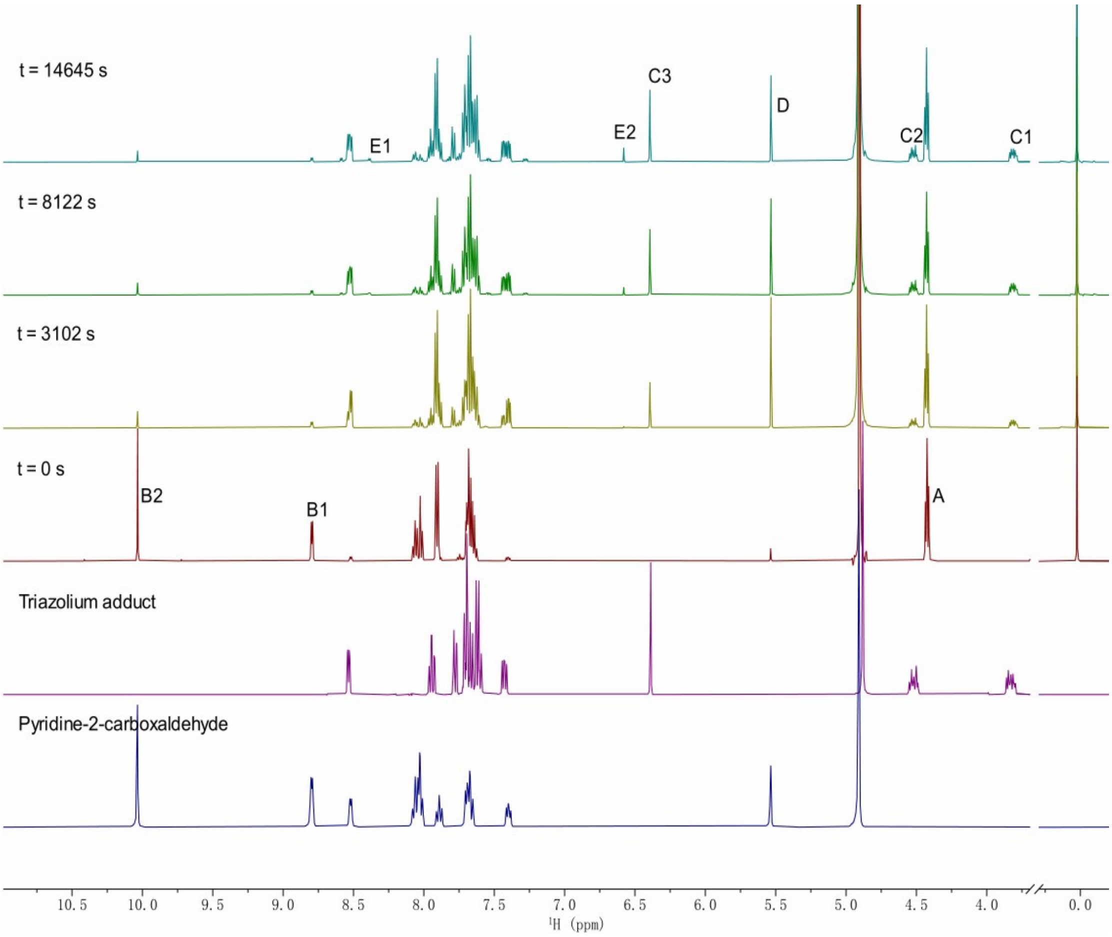

Figure S52. Representative ^{1}H NMR spectra (400 MHz) for reaction of pyridine-2-carboxaldehyde **34** (0.02 M) with *N*-Ph NHC precursor **40** (0.02 M) under a triethylamine buffer (NEt_3:NEt_3·HCl, 2:1, 0.09 M) in CD_3OD at 25 °C. A = NHC precursor NCH_2, B1 =Ar*H*CHO, B2 =ArHC*H*O, C1 = Adduct NC$H_A$$H_B$, C2 = Adduct NC$H_A$$H_B$, C3 =Adduct C(α)*H*, D = Hemiacetal C*H*, E1 = Benzoin Ar*H*, E2 = Benzoin C*H*.

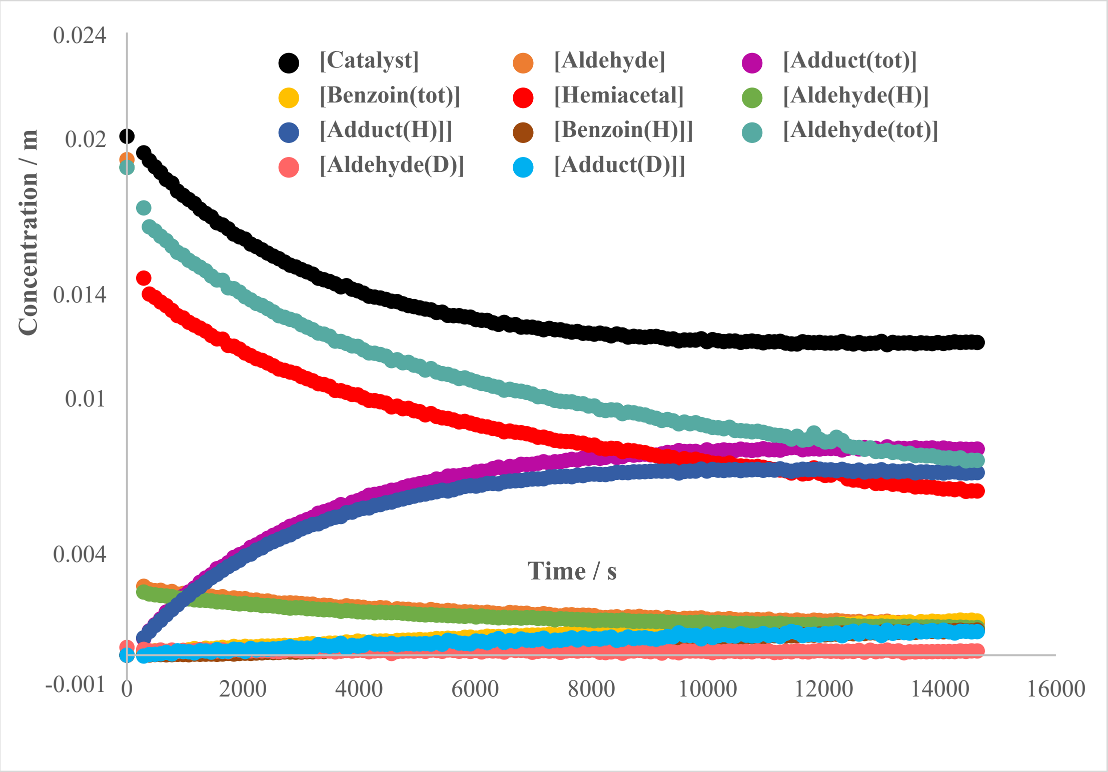

Figure S53. Concentration profile for the self-condensation of pyridine-2-carboxaldehyde **34** (0.02 M) with *N*-Ph NHC precursor **40** (0.02 M) under a triethylamine buffer (NEt_3:NEt_3·HCl, 2:1, 0.09 M) in CD_3OD at 25 °C.

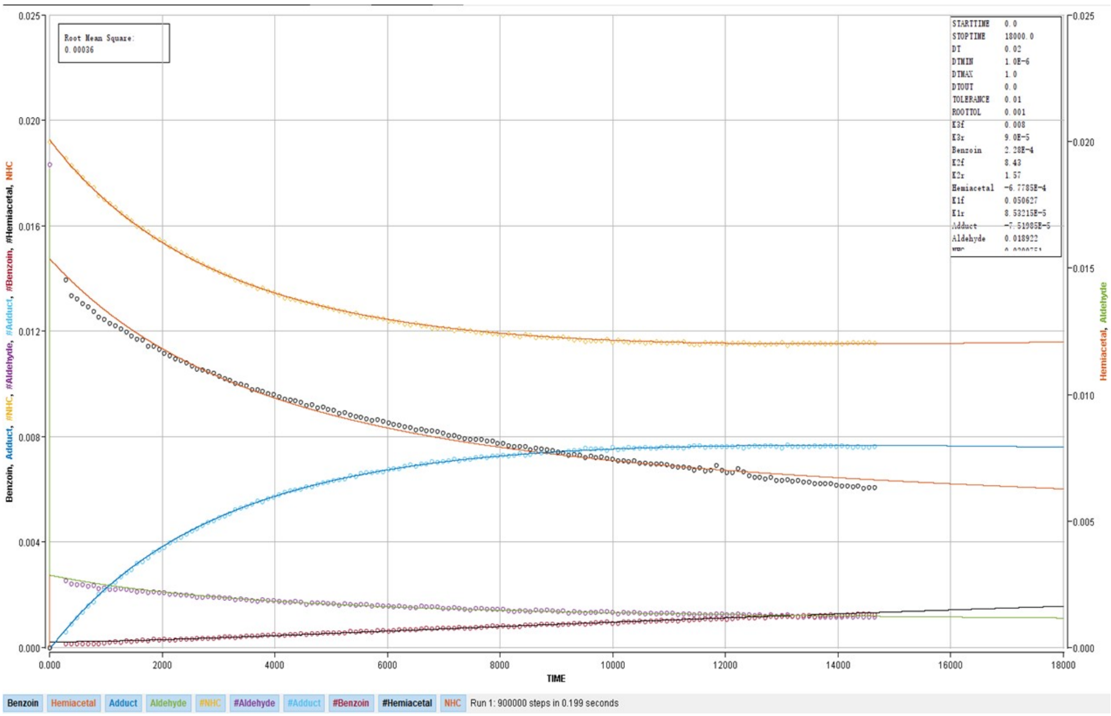

Figure S54. Global fitting profile for the reaction of pyridine-2-carboxaldehyde **34** (0.02 M) with *N*-Ph NHC precursor **40** (0.02 M) under a triethylamine buffer (NEt_3:NEt_3·HCl, 2:1, 0.09 M) in CD_3OD at 25 °C. Open circles show the experimental data, with the solid line representing the fit to the

kinetic model. Fitting data from t = 0 to t = 18000 s from Figure S53.

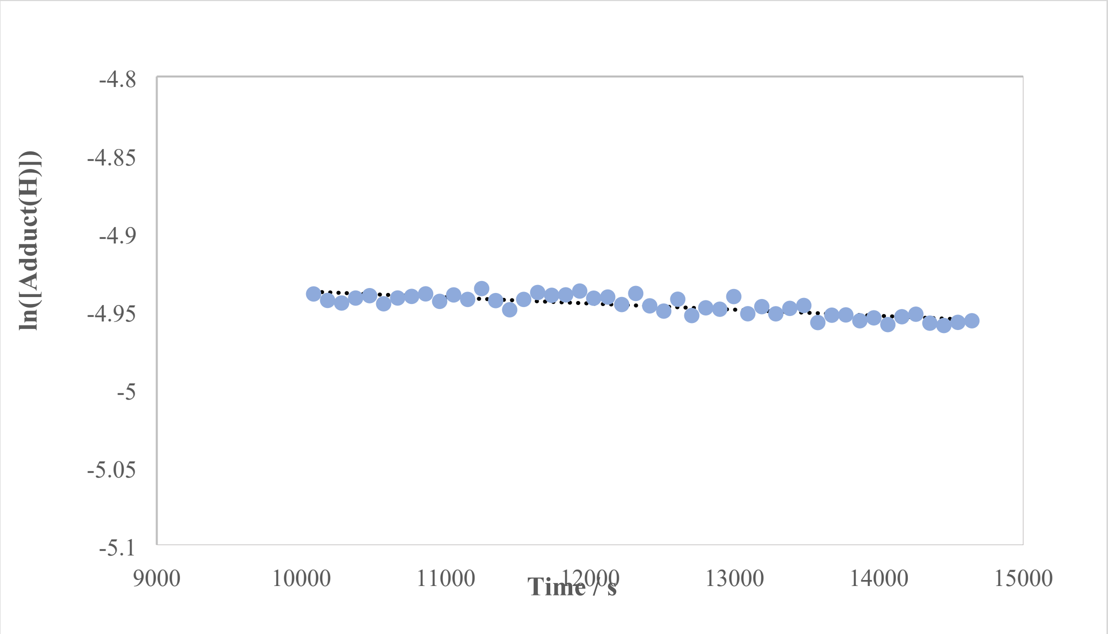

Figure S55. Semilogarithmic plots of [Adduct (H)] against time for the reactions of pyridine-2-carboxaldehyde **34** (0.02 M) with *N*-Ph NHC precursor **40** (0.02 M) under a triethylamine buffer (NEt_3:NEt_3·HCl, 2:1, 0.09 M) in CD_3OD at 25 °C.

Table 1 Entry 14

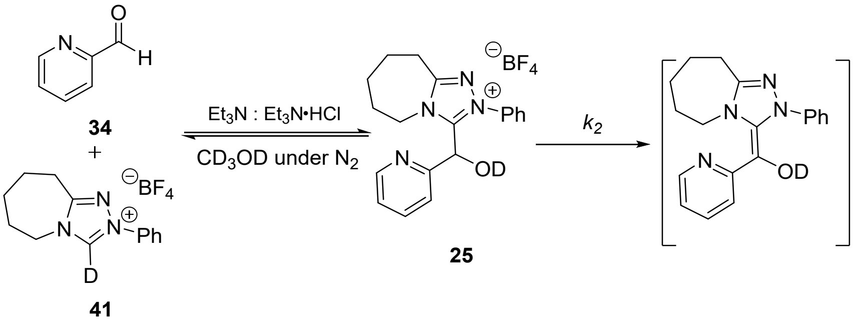

The reaction of aldehyde **34** and triazolium precatalyst **41** was monitored using ^{1}H NMR spectra, with representative NMR spectra over the course of the experiment given in Figure S56.

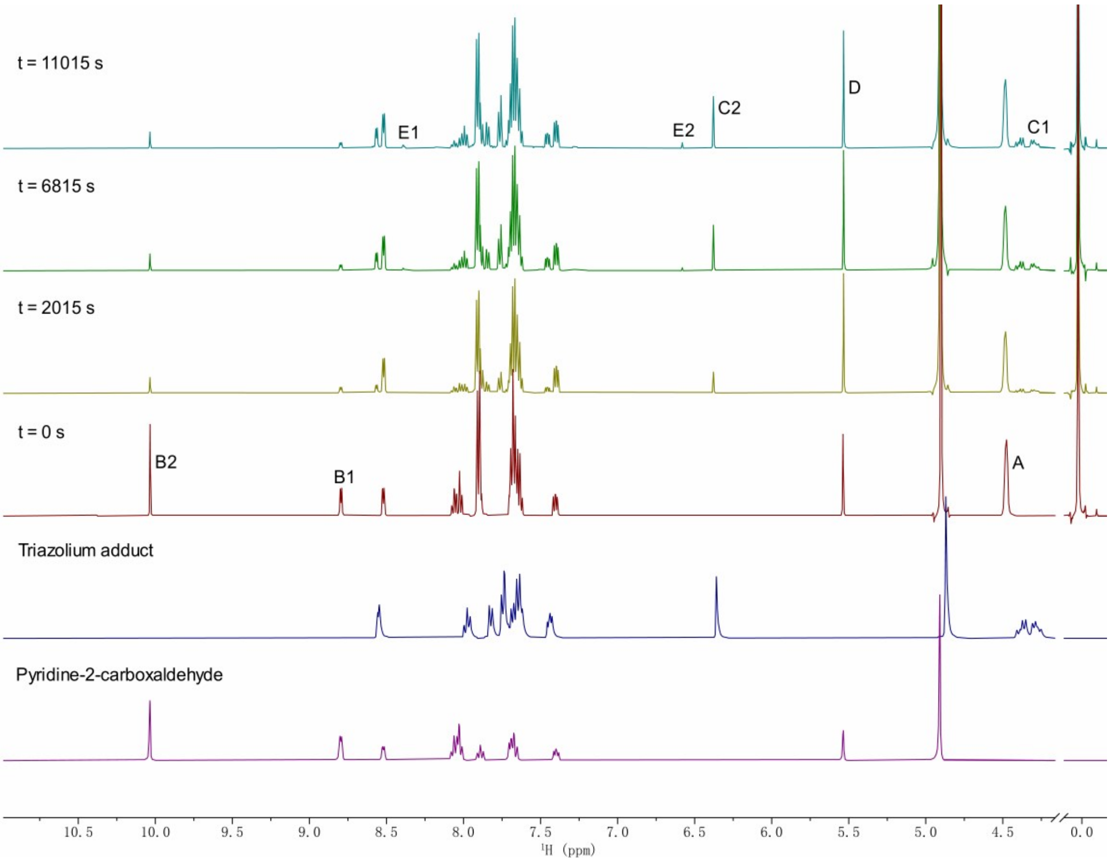

Figure S56. Representative ^{1}H NMR spectra (400 MHz) for reaction of pyridine-2-carboxaldehyde **34** (0.02 M) with *N*-Ph NHC precursor **41** (0.02 M) under a triethylamine buffer (NEt_3:NEt_3·HCl, 2:1, 0.09 M) in CD_3OD at 25 °C. A = NHC precursor NCH_2, B1 =Ar*H*CHO, B2 =ArHC*H*O, C1 = Adduct NCH_AH_B, C2 =Adduct C(α)*H*, D = Hemiacetal C*H*, E1 = Benzoin Ar*H*, E2 = Benzoin C*H*.

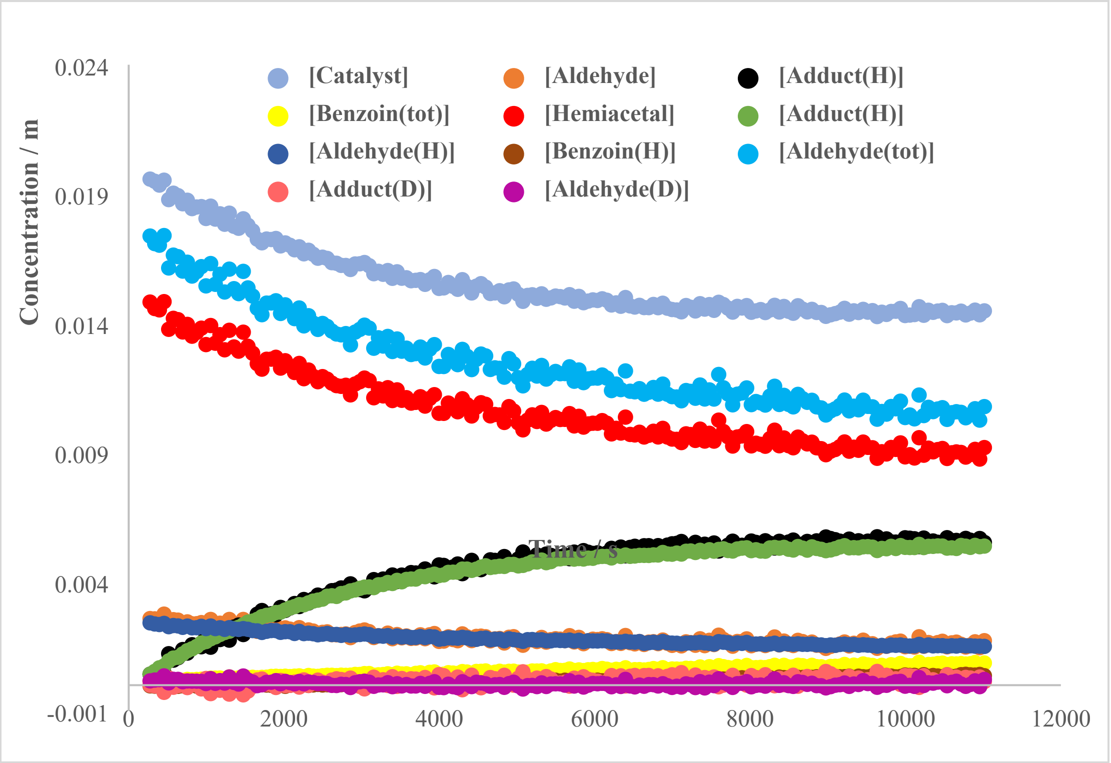

Figure S57. Concentration profile for the self-condensation of pyridine-2-carboxaldehyde **34** (0.02 M) with *N*-Ph NHC precursor **41** (0.02 M) under a triethylamine buffer (NEt_3:NEt_3·HCl, 2:1, 0.09 M) in CD_3OD at 25 °C.

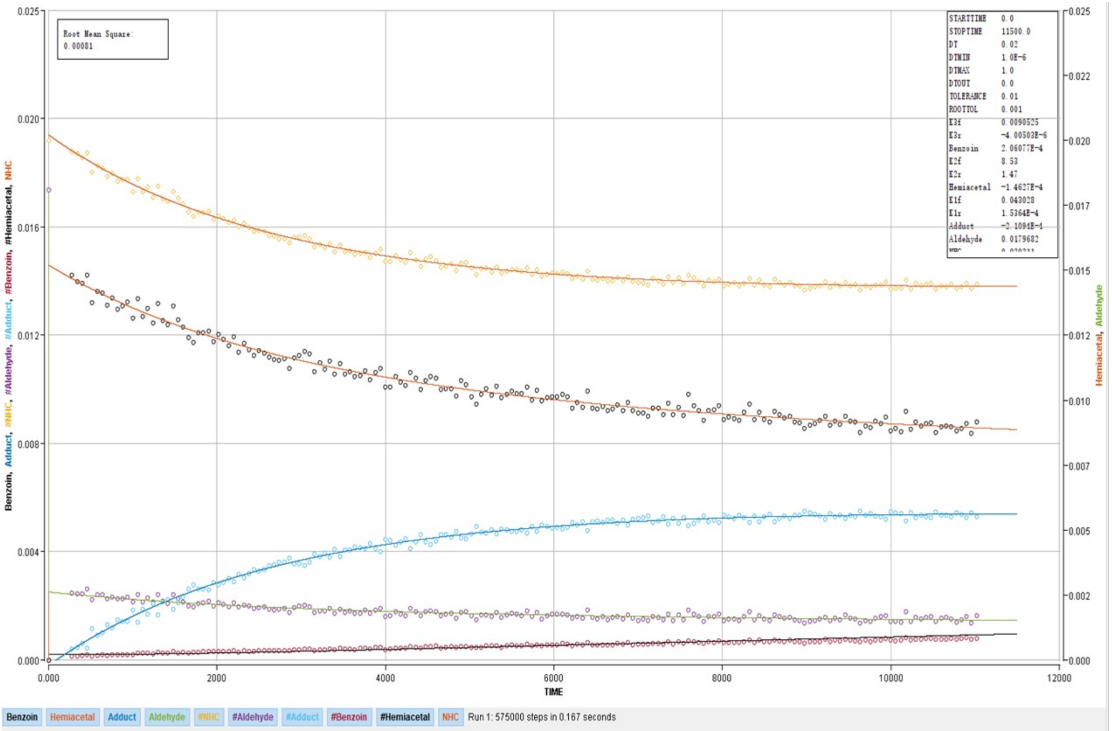

Figure S58. Global fitting profile for the reaction of pyridine-2-carboxaldehyde **34** (0.02 M) with *N*-Ph NHC precursor **41** (0.02 M) under a triethylamine buffer (NEt_3:NEt_3·HCl, 2:1, 0.09 M) in CD_3OD at 25 °C. Open circles show the experimental data, with the solid line representing the fit to the kinetic model. Fitting data from t = 0 to t = 12000 s from Figure S57.

Table 1 Entry 15

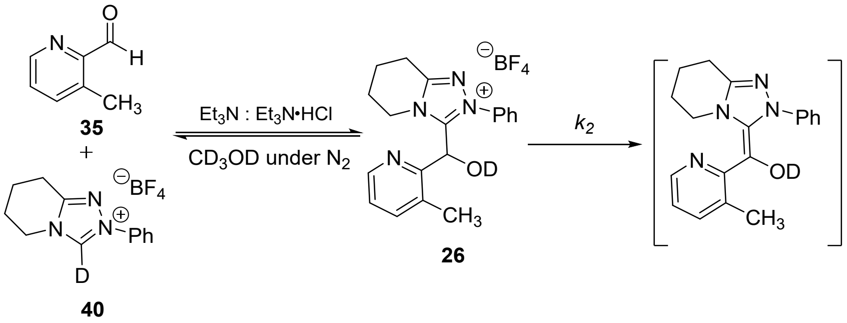

The reaction of aldehyde **35** and triazolium precatalyst **40** was monitored using ^1H NMR spectra, with representative NMR spectra over the course of the experiment given in Figure S59.

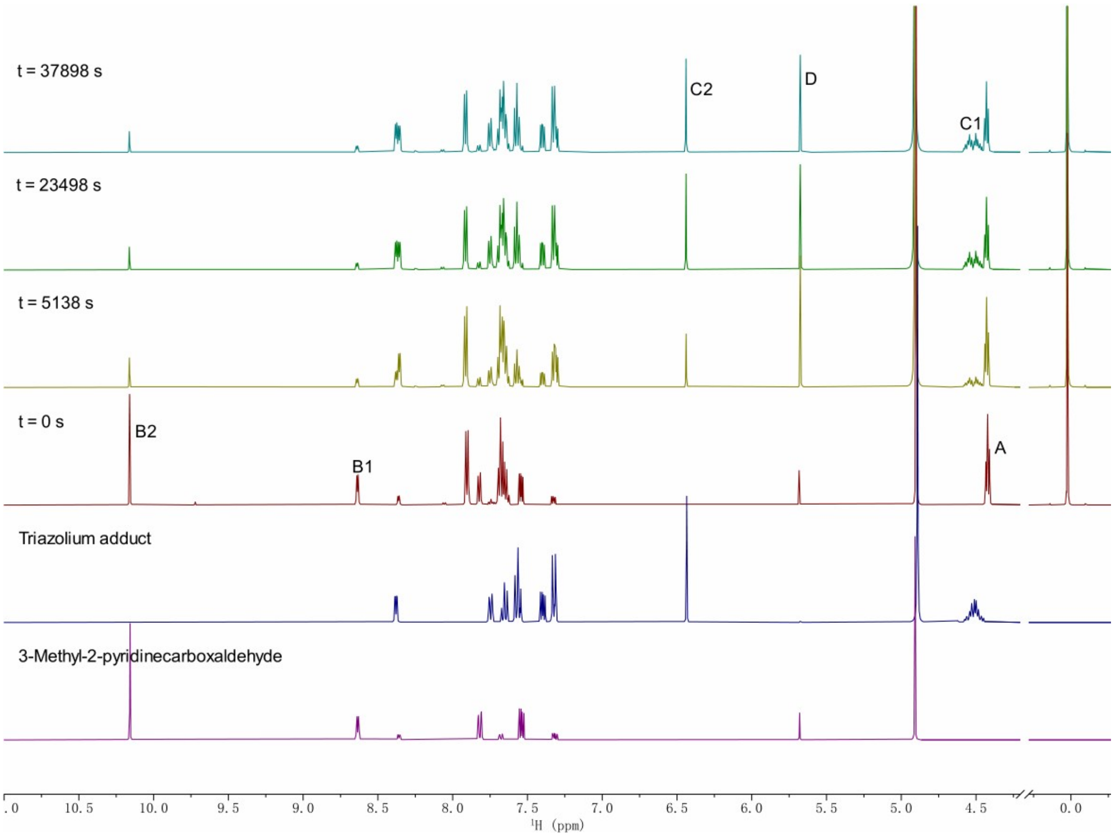

Figure S59. Representative ^{1}H NMR spectra (400 MHz) for reaction of 3-methyl-2-pyridinecarboxaldehyde **35** (0.02 M) with *N*-Ph NHC precursor **40** (0.02 M) under a triethylamine buffer (NEt_3:NEt_3·HCl, 2:1, 0.09 M) in CD_3OD at 25 °C. A = NHC precursor NCH_2, B1 =Ar*H*CHO, B2 =ArHC*H*O, C1 = Adduct NCH_2, C2 =Adduct C(α)*H,* D = Hemiacetal C*H*.

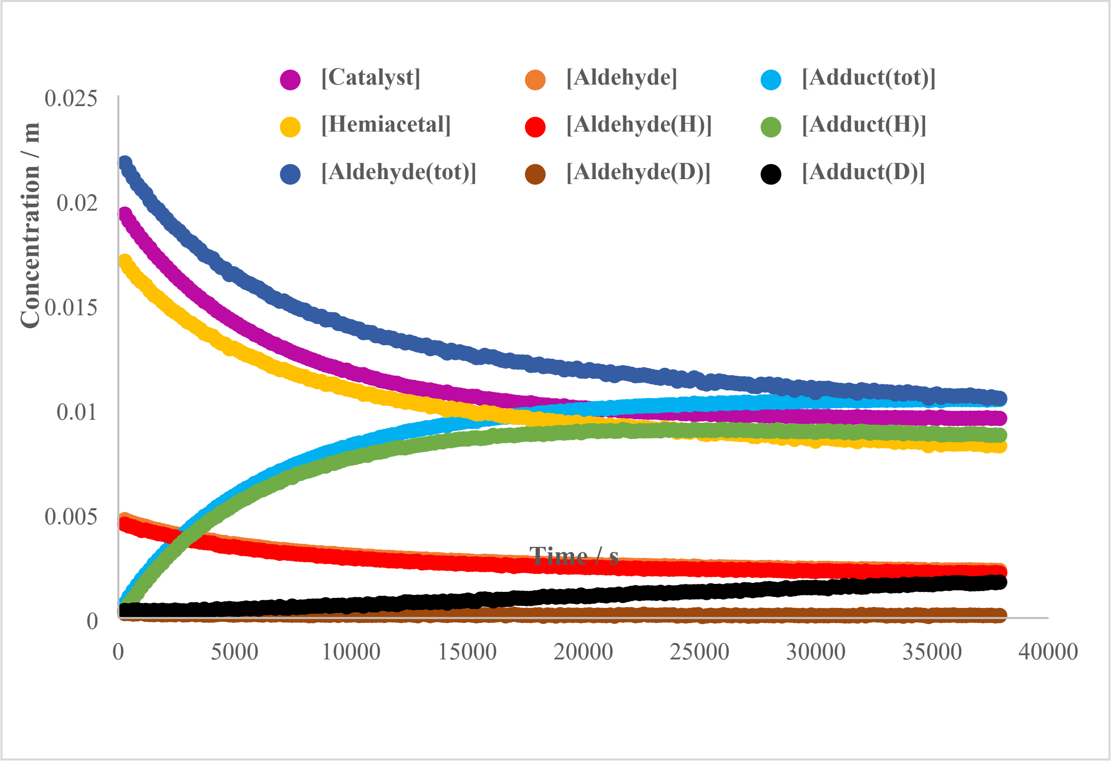

Figure S60. Concentration profile for the self-condensation of 3-methyl-2-pyridinecarboxaldehyde **35** (0.02 M) with *N*-Ph NHC precursor **40** (0.02 M) under a triethylamine buffer (NEt_3:NEt_3·HCl, 2:1, 0.09 M) in CD_3OD at 25 °C.

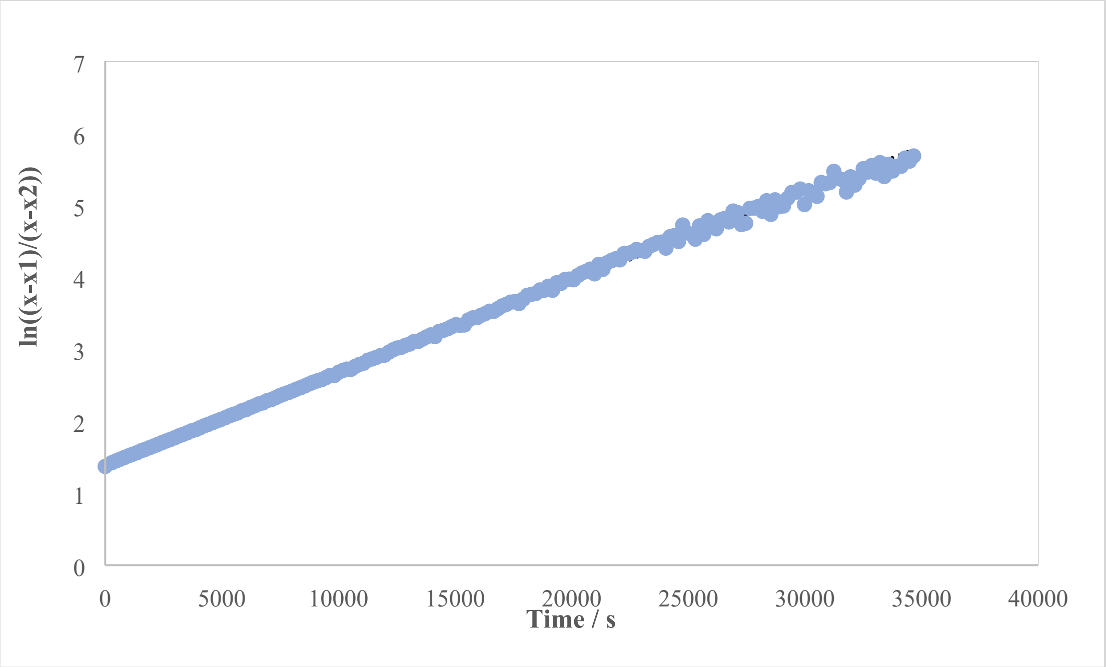

Figure S61. Semilogarithmic plots of $(x\text{-}x_1)/(x\text{-}x_2)$ against time, obtained from the reaction of 3-methyl-2-pyridinecarboxaldehyde **35** (0.02 M) with *N*-Ph NHC precursor **40** (0.02 M) under a triethylamine buffer (NEt_3:NEt_3·HCl, 2:1, 0.09 M) in CD_3OD at 25 °C.

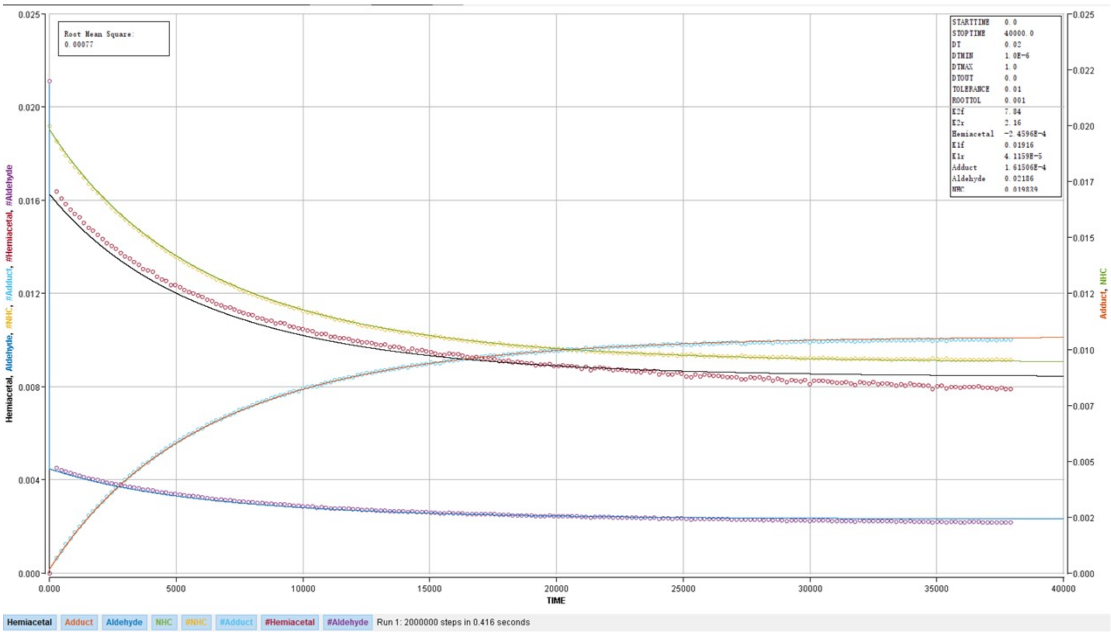

Figure S62. Global fitting profile for the reaction of 3-methyl-2-pyridinecarboxaldehyde **35** (0.02 M) with *N*-Ph NHC precursor **40** (0.02 M) under a triethylamine buffer (NEt_3:NEt_3·HCl, 2:1, 0.09 M) in CD_3OD at 25 °C. Open circles show the experimental data, with the solid line representing

the fit to the kinetic model. Fitting data from t = 0 to t = 40000 s from Figure S60.

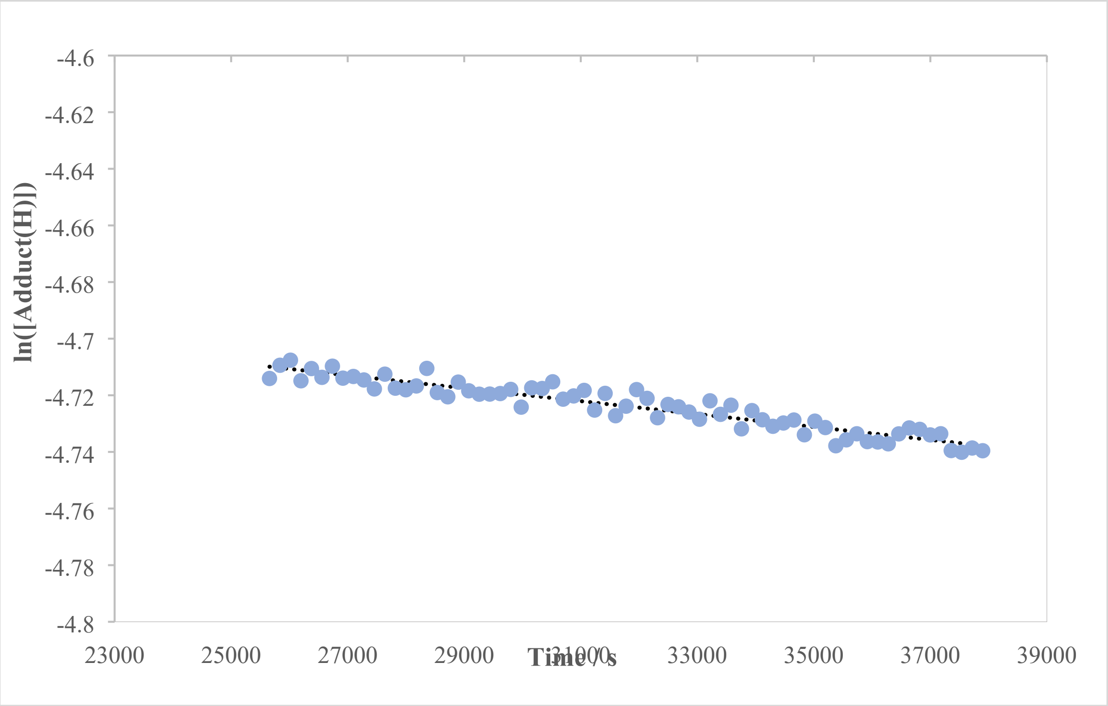

Figure S63. Semilogarithmic plots of [Adduct (H)] against time for the reactions of 3-methyl-2-pyridinecarboxaldehyde **35** (0.02 M) with *N*-Ph NHC precursor **40** (0.02 M) under a triethylamine buffer (NEt_3:NEt_3·HCl, 2:1, 0.09 M) in CD_3OD at 25 °C.

Table 1 Entry 16

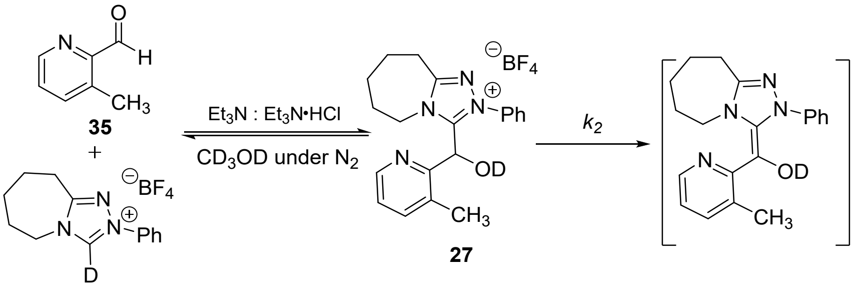

The reaction of aldehyde **1** and triazolium precatalyst **2** was monitored using ^{1}H NMR spectra, with representative NMR spectra over the course of the experiment given in Figure S64.

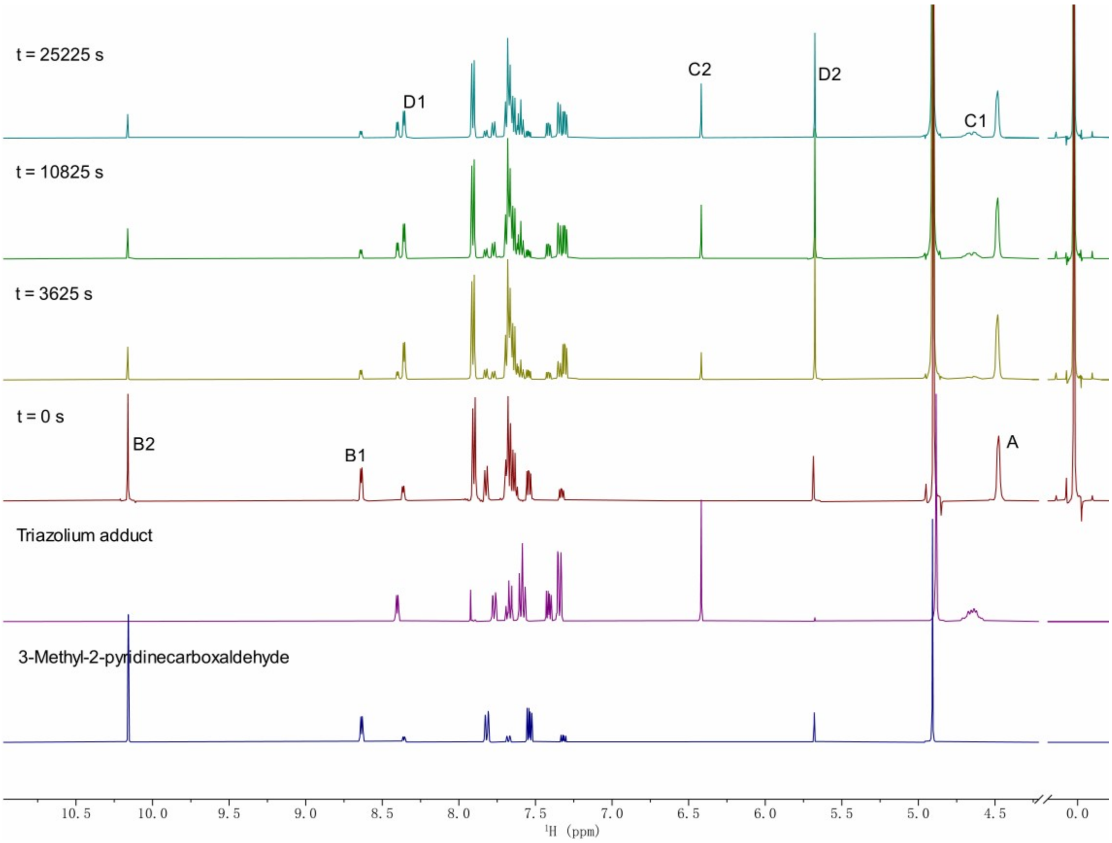

Figure S64. Representative ^{1}H NMR spectra (400 MHz) for reaction of 3-methyl-2-pyridinecarboxaldehyde **35** (0.02 M) with *N*-Ph NHC precursor **41** (0.02 M) under a triethylamine buffer (NEt_3:NEt_3·HCl, 2:1, 0.09 M) in CD_3OD at 25 °C. A = NHC precursor NCH_2, B1 =Ar*H*CHO, B2 =ArHC*H*O, C1 = Adduct NCH_2, C2 =Adduct C(α)*H,* D1 = Hemiacetal Ar*H*, D2 = Hemiacetal C*H*.

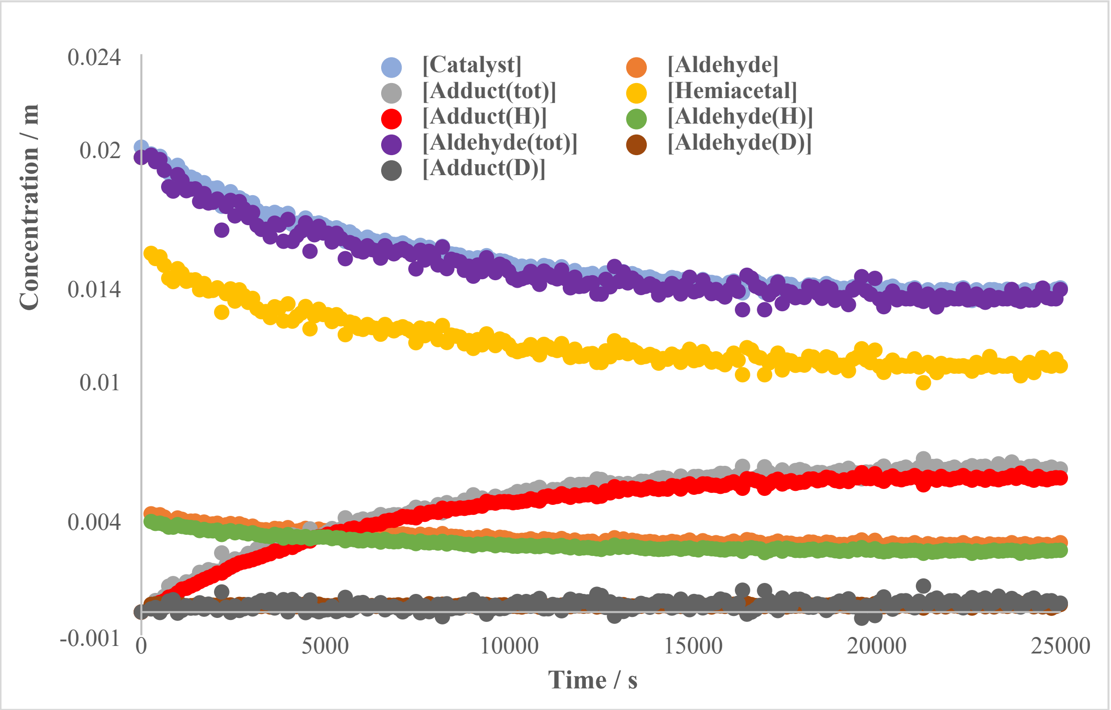

Figure S65. Concentration profile for the self-condensation of 3-methyl-2-pyridinecarboxaldehyde **35** (0.02 M) with *N*-Ph NHC precursor **41** (0.02 M) under a triethylamine buffer (NEt_3:NEt_3·HCl, 2:1, 0.09 M) in CD_3OD at 25 °C.

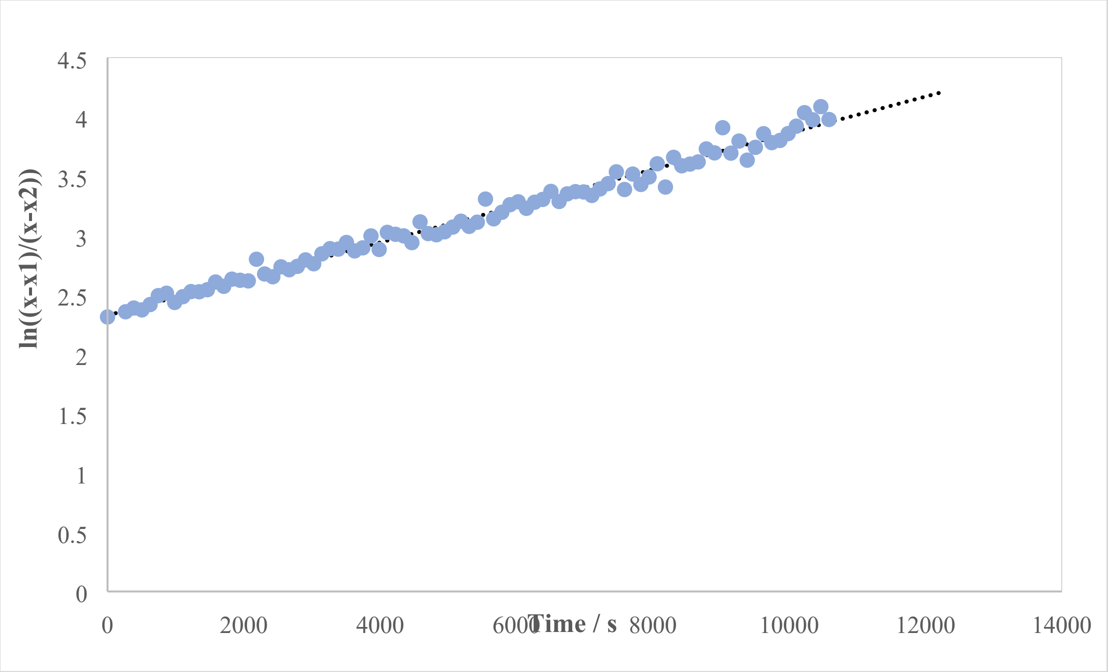

Figure S66. Semilogarithmic plots of $(x-x_1)/(x-x_2)$ against time, obtained from the reaction of 3-methyl-2-pyridinecarboxaldehyde **35** (0.02 M) with *N*-Ph NHC precursor **41** (0.02 M) under a triethylamine buffer (NEt_3:NEt_3·HCl, 2:1, 0.09 M) in CD_3OD at 25 °C.

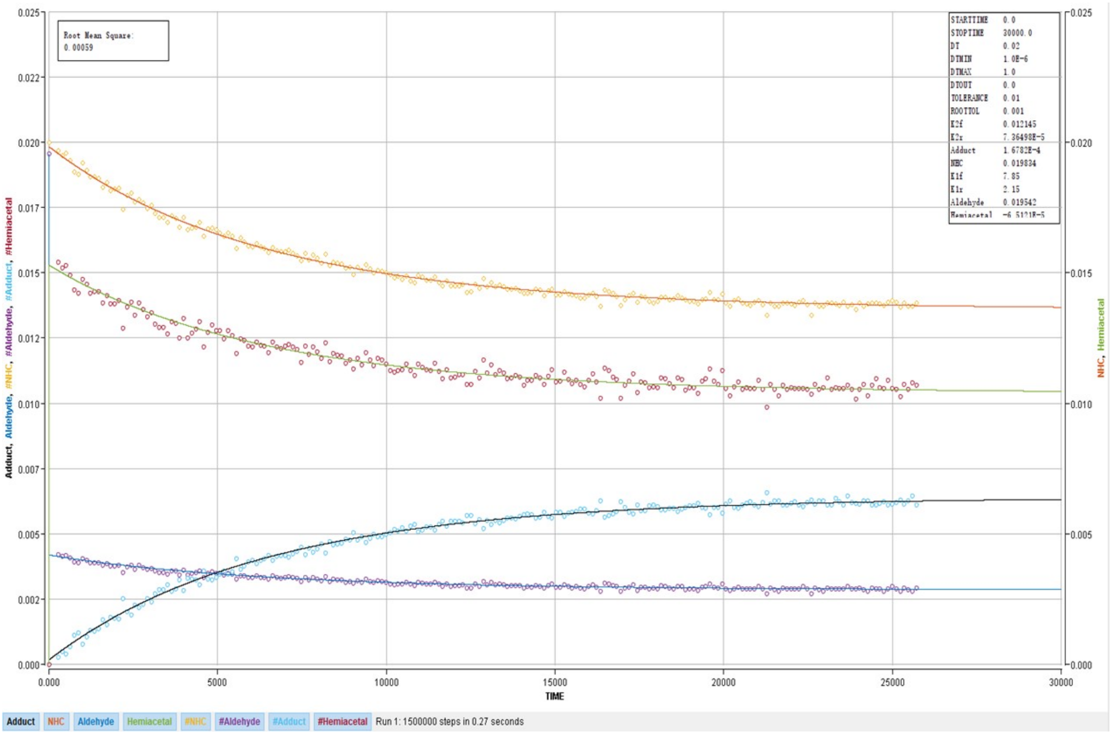

Figure S67. Global fitting profile for the reaction of 3-methyl-2-pyridinecarboxaldehyde **35** (0.02

M) with *N*-Ph NHC precursor **41** (0.02 M) under a triethylamine buffer (NEt_3:NEt_3·HCl, 2:1, 0.09 M) in CD_3OD at 25 °C. Open circles show the experimental data, with the solid line representing the fit to the kinetic model. Fitting data from t = 0 to t = 30000 s from Figure S65.

Table 1 Entry 17

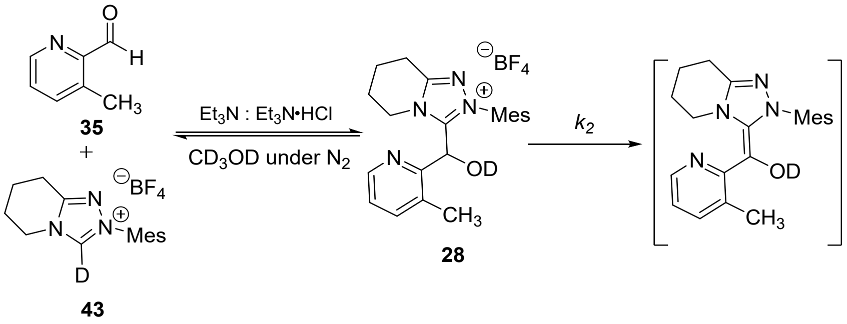

The reaction of aldehyde 35 and triazolium precatalyst **43** was monitored using ^{1}H NMR spectra, with representative NMR spectra over the course of the experiment given in Figure S68.

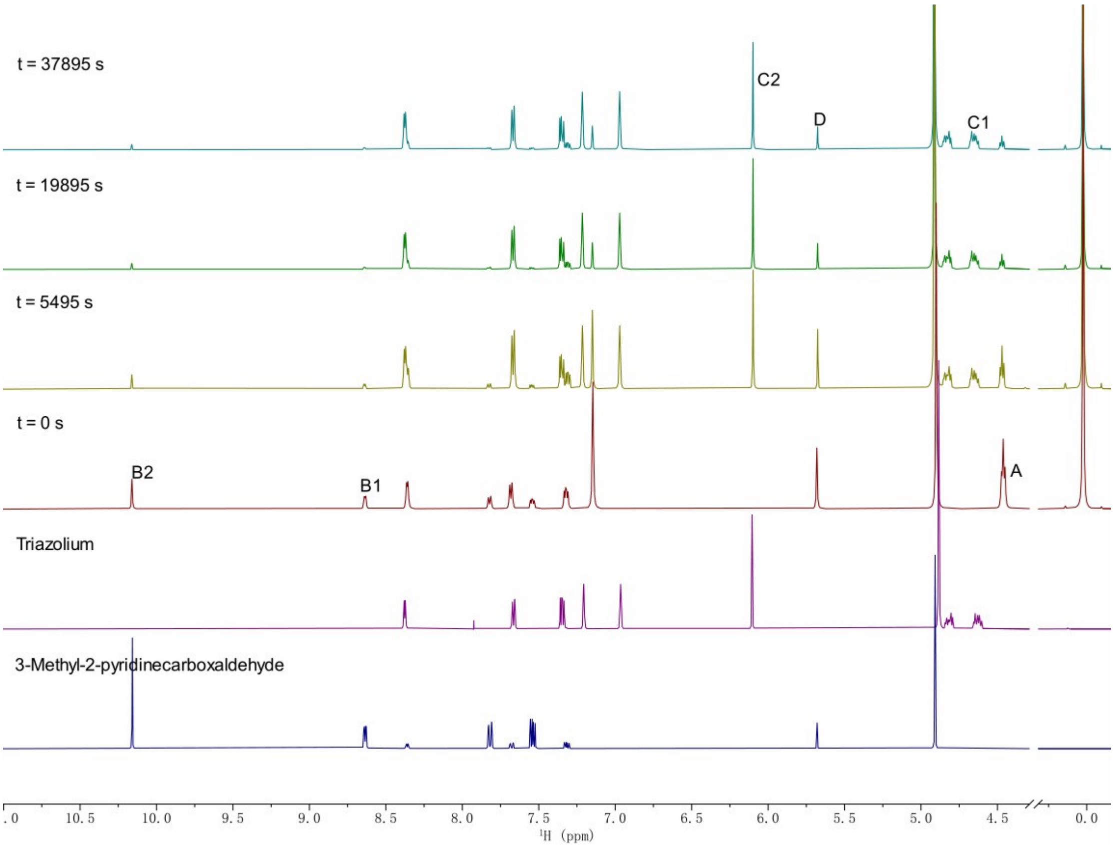

Figure S68. Representative ^{1}H NMR spectra (400 MHz) for reaction of 3-methyl-2-pyridinecarboxaldehyde **35** (0.02 M) with *N*-Mes NHC precursor **43** (0.02 M) under a triethylamine buffer (NEt_3:NEt_3·HCl, 2:1, 0.09 M) in CD_3OD at 25 °C. A = NHC precursor NCH_2, B1 =Ar*H*CHO, B2 =ArHC*H*O, C1 = Adduct NCH_2, C2 =Adduct C(α)*H,* D1 = Hemiacetal C*H*.

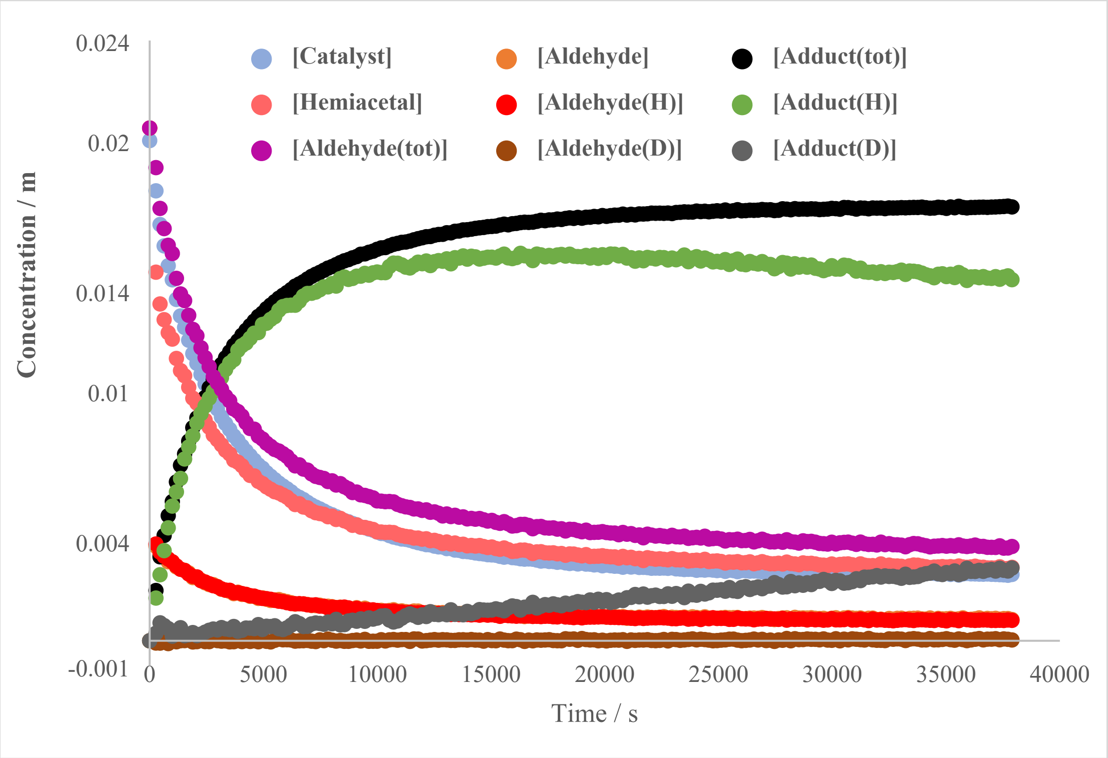

Figure S69. Concentration profile for the self-condensation of 3-methyl-2-pyridinecarboxaldehyde **35** (0.02 M) with *N*-Mes NHC precursor **43** (0.02 M) under a triethylamine buffer (NEt_3:NEt_3·HCl, 2:1, 0.09 M) in CD_3OD at 25 °C.

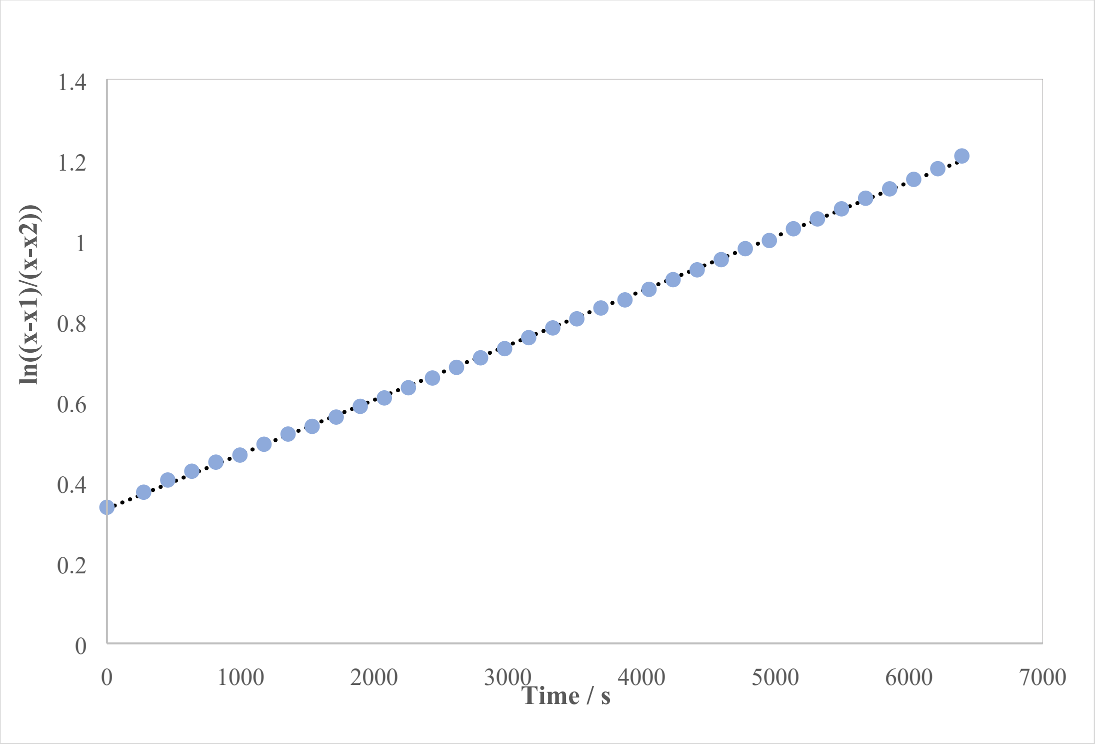

Figure S70. Semilogarithmic plots of $(x\text{-}x_1)/(x\text{-}x_2)$ against time, obtained from the reaction of 3-methyl-2-pyridinecarboxaldehyde **35** (0.02 M) with *N*-Mes NHC precursor **43** (0.02 M) under a triethylamine buffer (NEt_3:NEt_3·HCl, 2:1, 0.09 M) in CD_3OD at 25 °C.

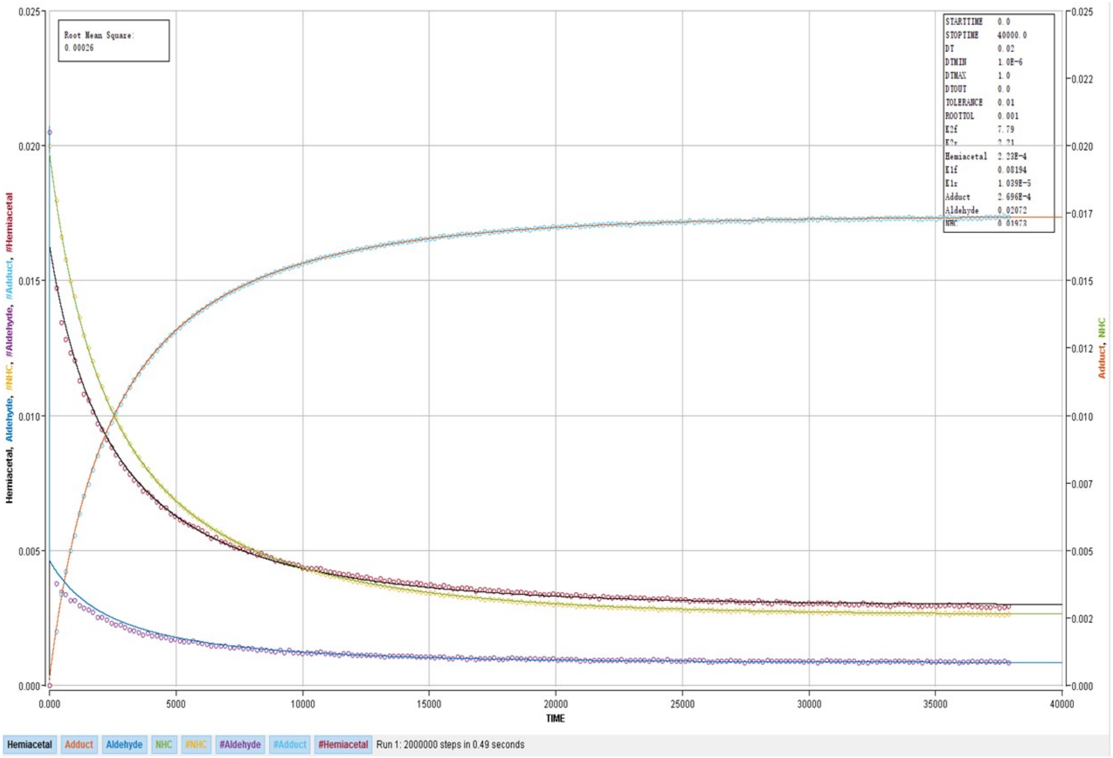

Figure S71. Global fitting profile for the reaction of 3-methyl-2-pyridinecarboxaldehyde **35** (0.02 M) with *N*-Mes NHC precursor **43** (0.02 M) under a triethylamine buffer (NEt_3:NEt_3·HCl, 2:1, 0.09 M) in CD_3OD at 25 °C. Open circles show the experimental data, with the solid line representing the fit to the kinetic model. Fitting data from t = 0 to t = 40000 s from Figure S69.

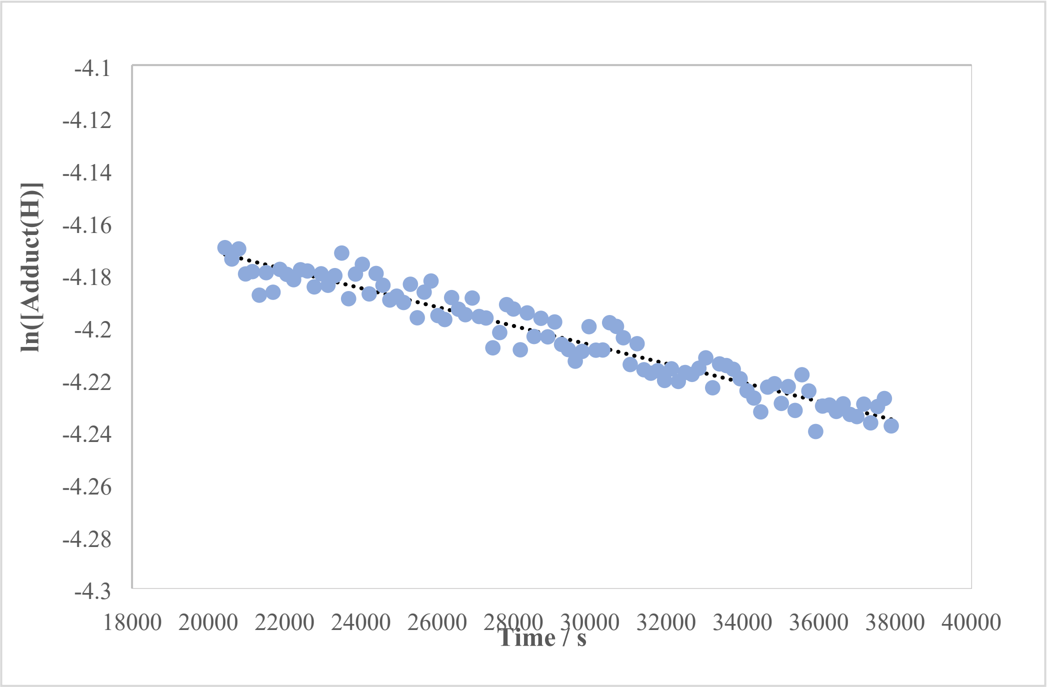

Figure S72. Semilogarithmic plots of [Adduct (H)] against time for the reactions of 3-methyl-2-pyridinecarboxaldehyde **35** (0.02 M) with *N*-Mes NHC precursor **43** (0.02 M) under a triethylamine buffer (NEt_3:NEt_3·HCl, 2:1, 0.09 M) in CD_3OD at 25 °C.

Table 1 Entry 18

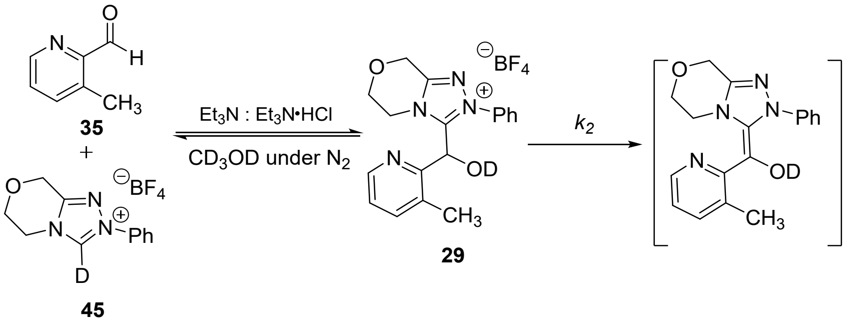

The reaction of aldehyde **35** and triazolium precatalyst **45** was monitored using ^{1}H NMR spectra, with representative NMR spectra over the course of the experiment given in Figure S73.

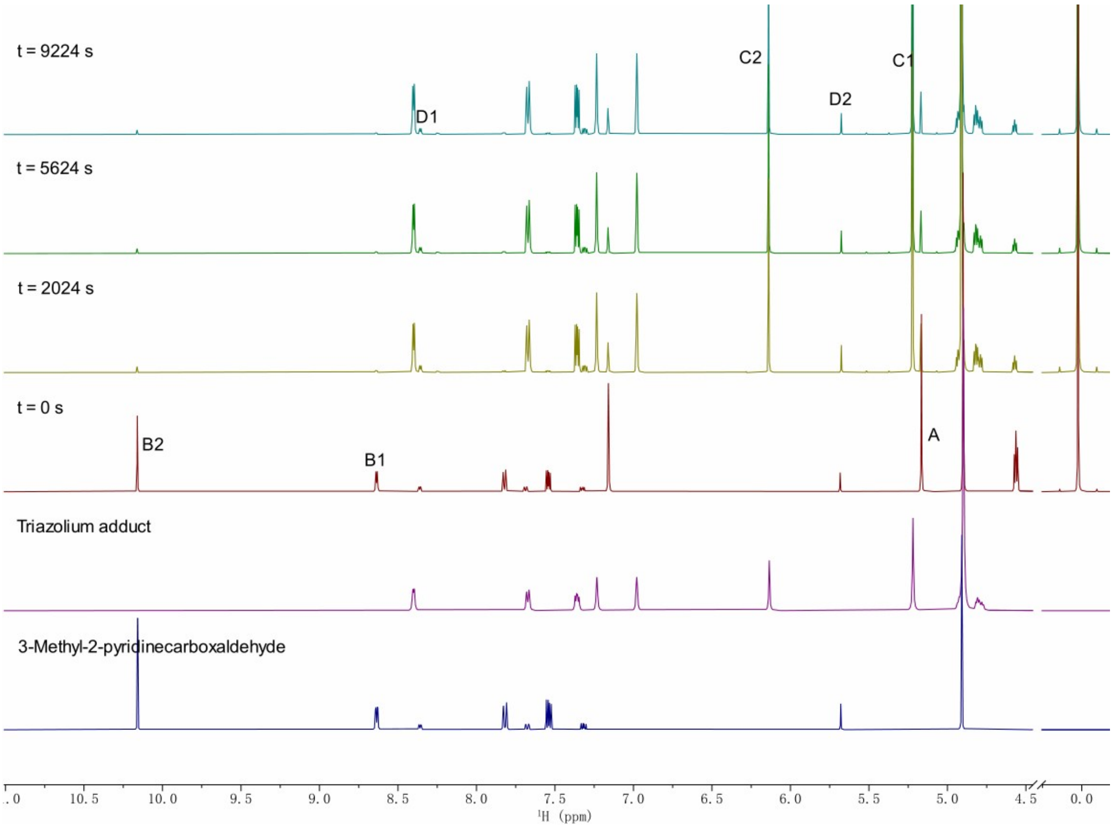

Figure S73. Representative ^{1}H NMR spectra (400 MHz) for reaction of 3-methyl-2-pyridinecarboxaldehyde **35** (0.02 M) with *N*-Ph NHC precursor **45** (0.02 M) under a triethylamine buffer (NEt_3:NEt_3·HCl, 2:1, 0.09 M) in CD_3OD at 25 °C. A = NHC precursor OC*H*$_2$C=, B1 =Ar*H*CHO, B2 =ArHC*H*O, C1 = Adduct OC*H*$_2$C=, C2 =Adduct C(α)*H,* D1 = Hemiacetal Ar*H*, D2 = Hemiacetal C*H*.

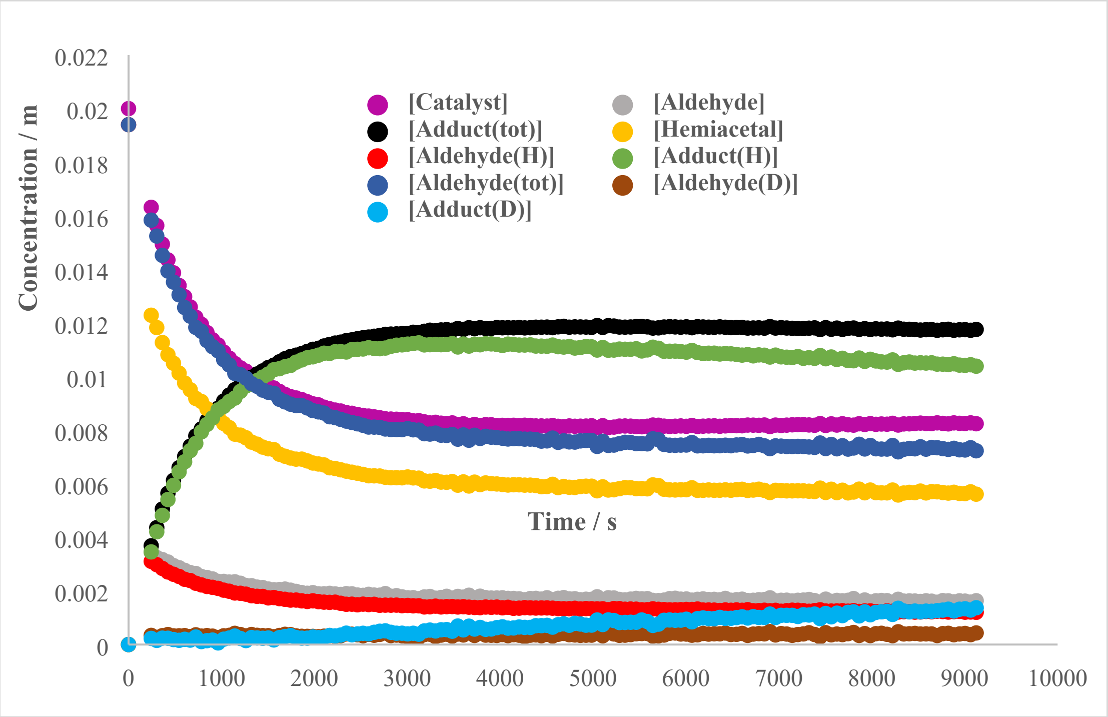

Figure S74. Concentration profile for the self-condensation of 3-methyl-2-pyridinecarboxaldehyde **35** (0.02 M) with *N*-Ph NHC precursor **45** (0.02 M) under a triethylamine buffer (NEt_3:NEt_3·HCl, 2:1, 0.09 M) in CD_3OD at 25 °C.

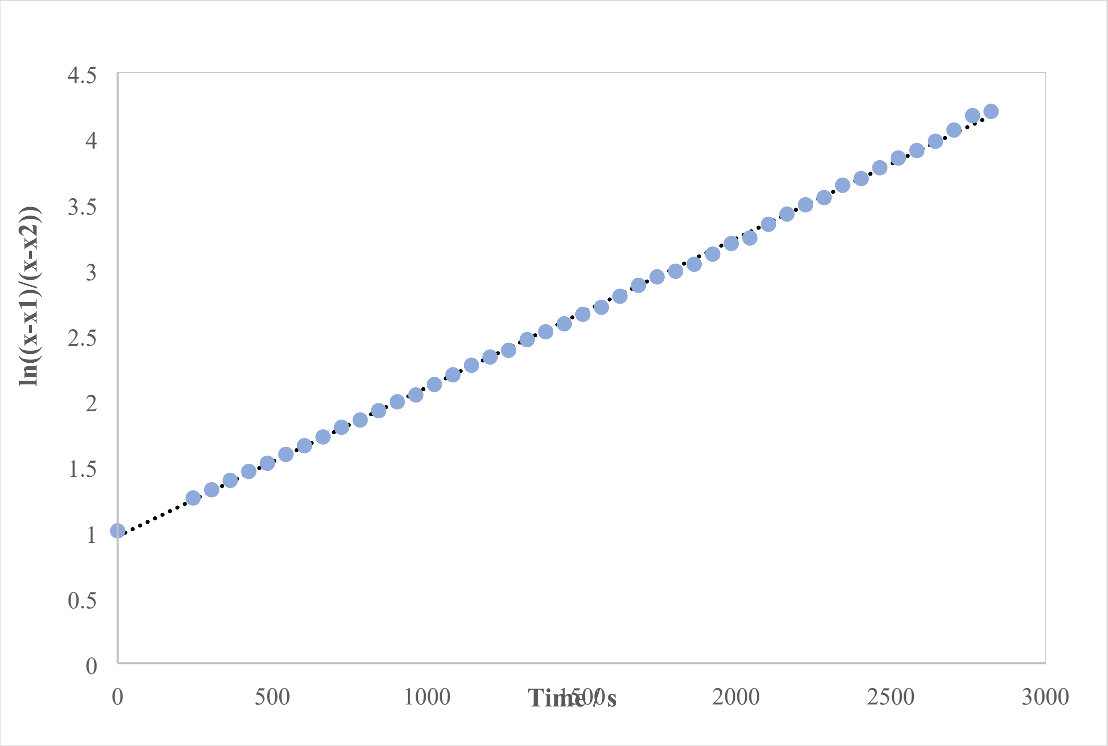

Figure S75. Semilogarithmic plots of $(x-x_1)/(x-x_2)$ against time, obtained from the reaction of 3-methyl-2-pyridinecarboxaldehyde **35** (0.02 M) with *N*-Ph NHC precursor **45** (0.02 M) under a triethylamine buffer (NEt_3:NEt_3·HCl, 2:1, 0.09 M) in CD_3OD at 25 °C.

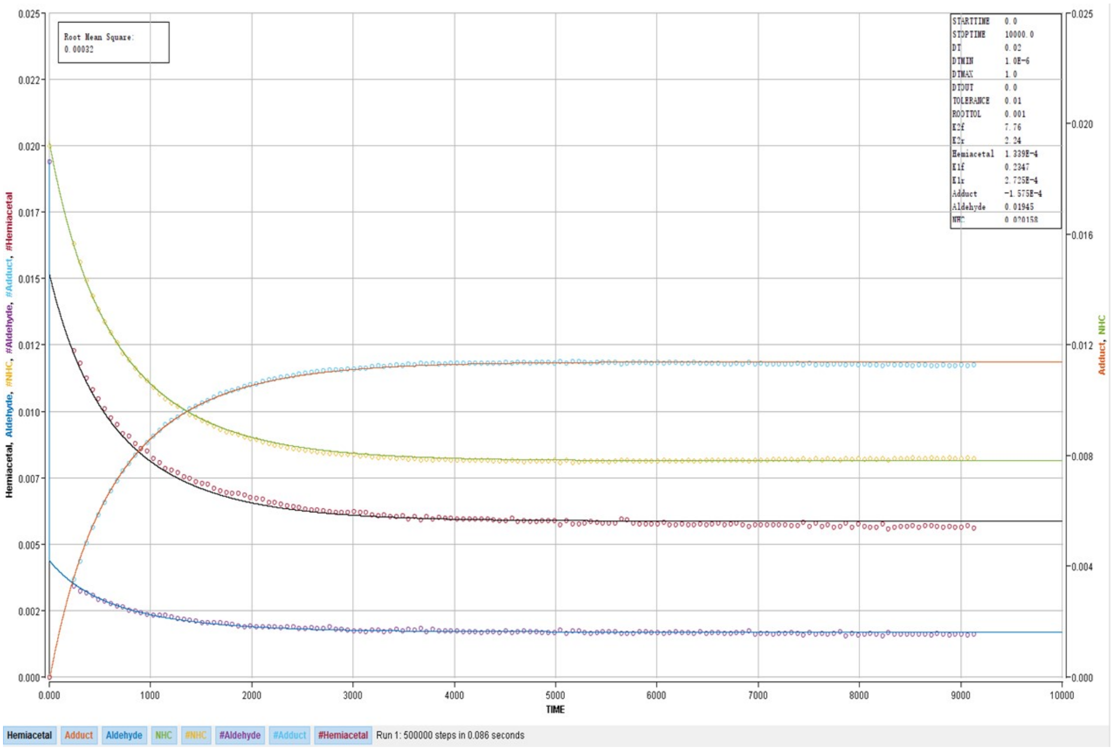

Figure S76. Global fitting profile for the reaction of 3-methyl-2-pyridinecarboxaldehyde **35** (0.02 M) with *N*-Ph NHC precursor **45** (0.02 M) under a triethylamine buffer (NEt_3:NEt_3·HCl, 2:1, 0.09 M) in CD_3OD at 25 °C. Open circles show the experimental data, with the solid line representing the fit to the kinetic model. Fitting data from t = 0 to t = 10000 s from Figure S74.

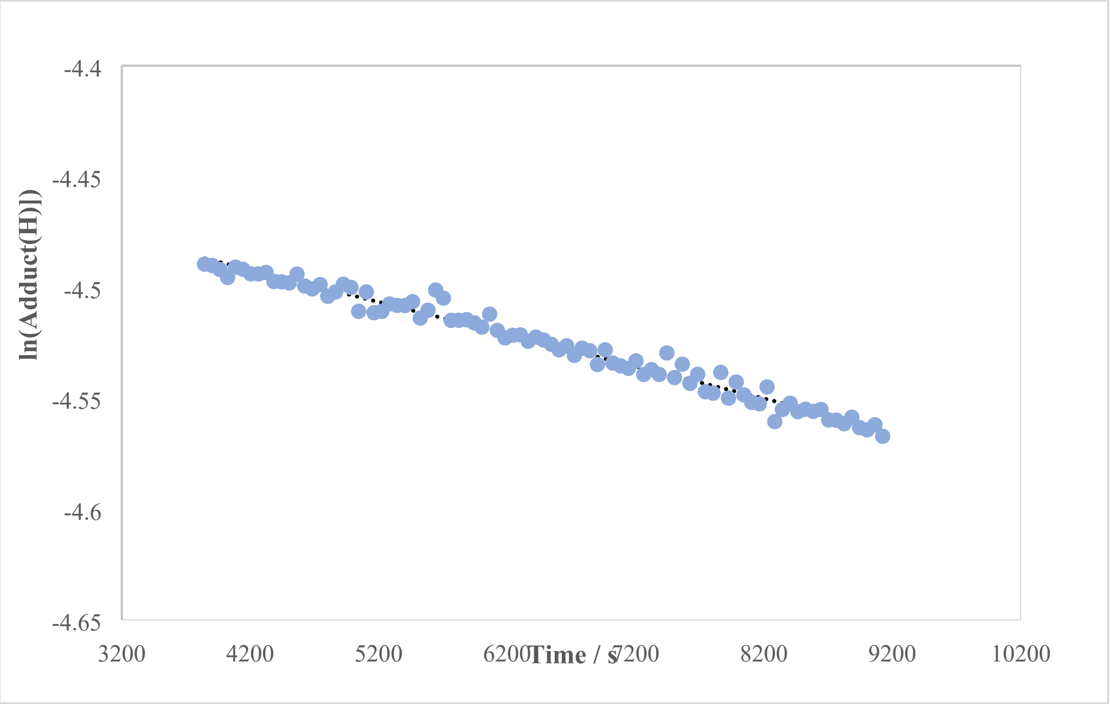

Figure S77. Semilogarithmic plots of [Adduct (H)] against time for the reactions of 3-methyl-2-pyridinecarboxaldehyde **35** (0.02 M) with *N*-Ph NHC precursor **45** (0.02 M) under a triethylamine buffer (NEt_3:NEt_3·HCl, 2:1, 0.09 M) in CD_3OD at 25 °C.

Table 1 Entry 19

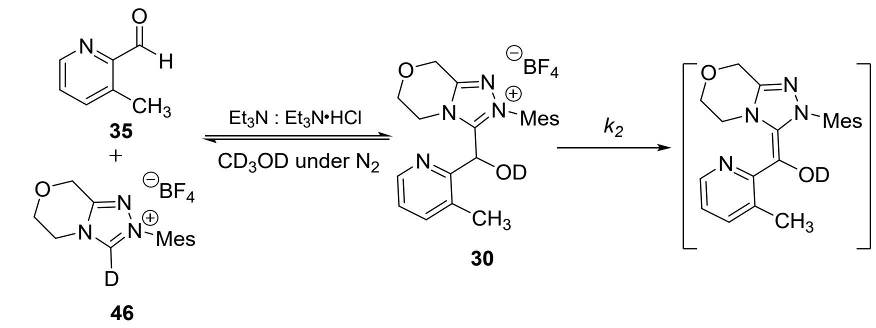

The reaction of aldehyde **35** and triazolium precatalyst **46** was monitored using ^{1}H NMR spectra, with representative NMR spectra over the course of the experiment given in Figure S78.

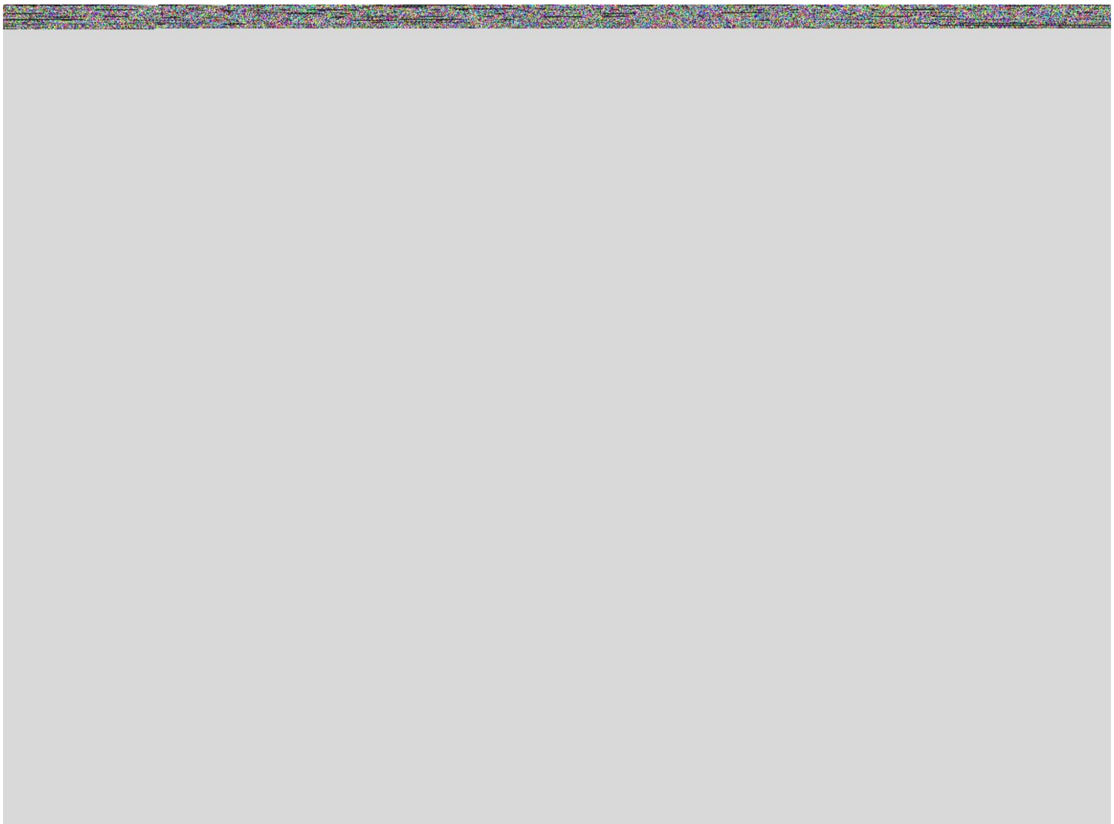

Figure S78. Representative ^{1}H NMR spectra (400 MHz) for reaction of 3-methyl-2-pyridinecarboxaldehyde **35** (0.02 M) with *N*-Mes NHC precursor **46** (0.02 M) under a triethylamine buffer (NEt_3:NEt_3·HCl, 2:1, 0.09 M) in CD_3OD at 25 °C. A = NHC precursor OC*H₂*C=, B1 =Ar*H*CHO, B2 =ArHC*H*O, C1 = Adduct OC*H₂*C=, C2 =Adduct C(α)*H,* D1 = Hemiacetal Ar*H*, D2 = Hemiacetal C*H*.

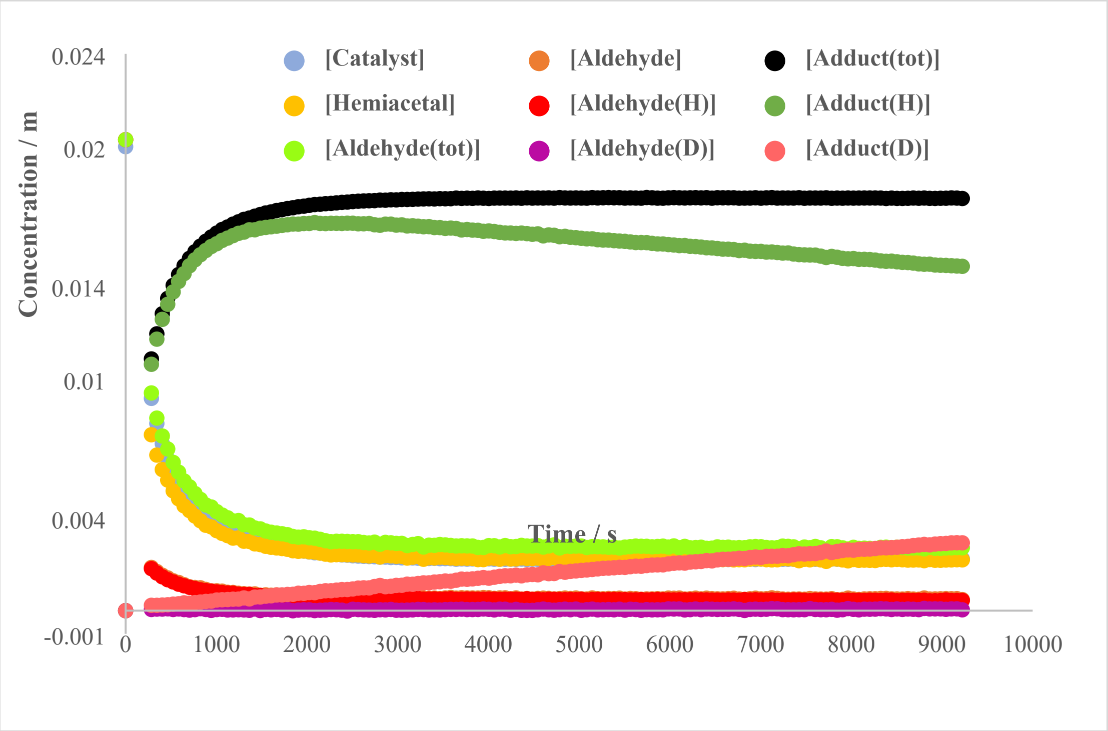

Figure S79. Concentration profile for the self-condensation of 3-methyl-2-pyridinecarboxaldehyde **35** (0.02 M) with *N*-Mes NHC precursor **46** (0.02 M) under a triethylamine buffer (NEt_3:NEt_3·HCl, 2:1, 0.09 M) in CD_3OD at 25 °C.

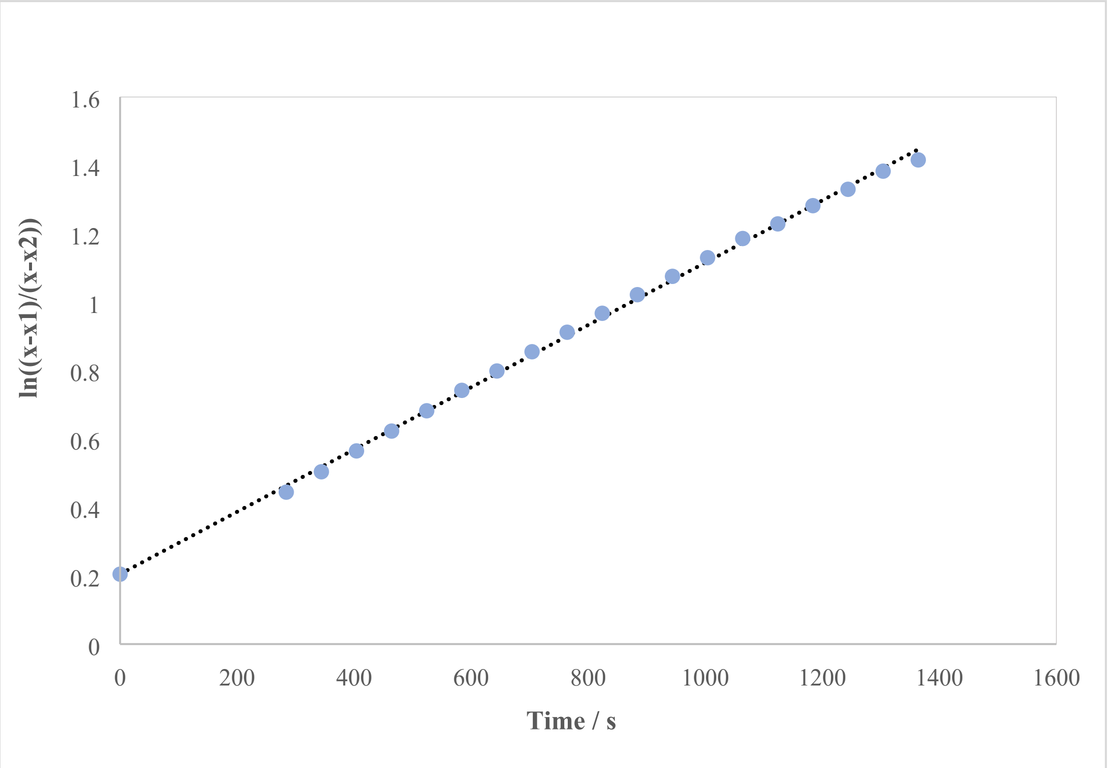

Figure S80. Semilogarithmic plots of $(x\text{-}x_1)/(x\text{-}x_2)$ against time, obtained from the reaction of 3-methyl-2-pyridinecarboxaldehyde **35** (0.02 M) with *N*-Mes NHC precursor **46** (0.02 M) under a triethylamine buffer (NEt_3:NEt_3·HCl, 2:1, 0.09 M) in CD_3OD at 25 °C.

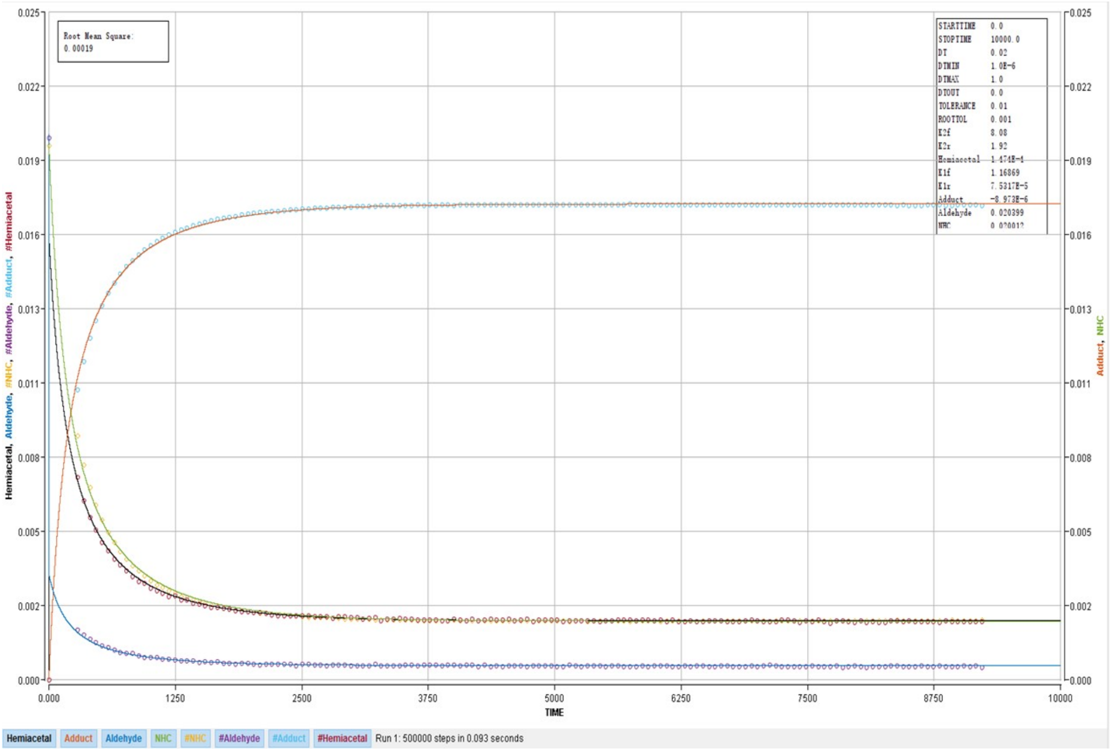

Figure S81. Global fitting profile for the reaction of 3-methyl-2-pyridinecarboxaldehyde **35** (0.02 M) with *N*-Mes NHC precursor **46** (0.02 M) under a triethylamine buffer (NEt_3:NEt_3·HCl, 2:1, 0.09 M) in CD_3OD at 25 °C. Open circles show the experimental data, with the solid line representing the fit to the kinetic model. Fitting data from t = 0 to t = 10000 s from Figure S79.

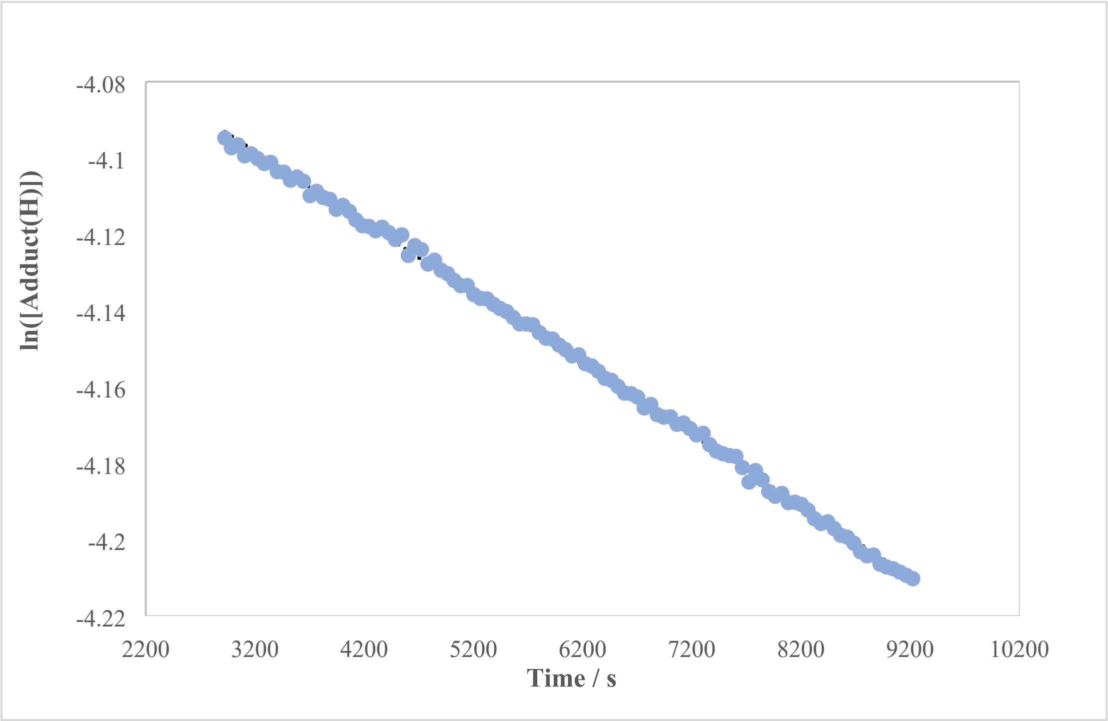

Figure S82. Semilogarithmic plots of [Adduct (H)] against time for the reactions of 3-methyl-2-pyridinecarboxaldehyde **35** (0.02 M) with *N*-Mes NHC precursor **46** (0.02 M) under a triethylamine buffer (NEt_3:NEt_3·HCl, 2:1, 0.09 M) in CD_3OD at 25 °C.

Unsuccessful samples (Adduct 16 and 22)

Data extraction for the following two samples was unsuccessful as the furfural concentration couldn't reach the equilibrium with side reactions.

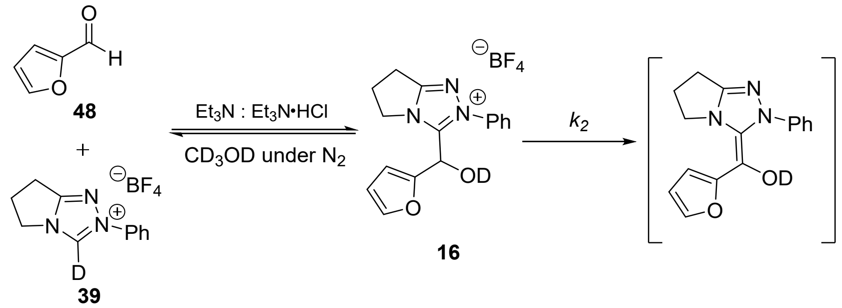

The reaction of furfural **48** and triazolium precatalyst **39** was monitored using ^1H NMR spectra, with representative NMR spectra over the course of the experiment given in Figure S83.

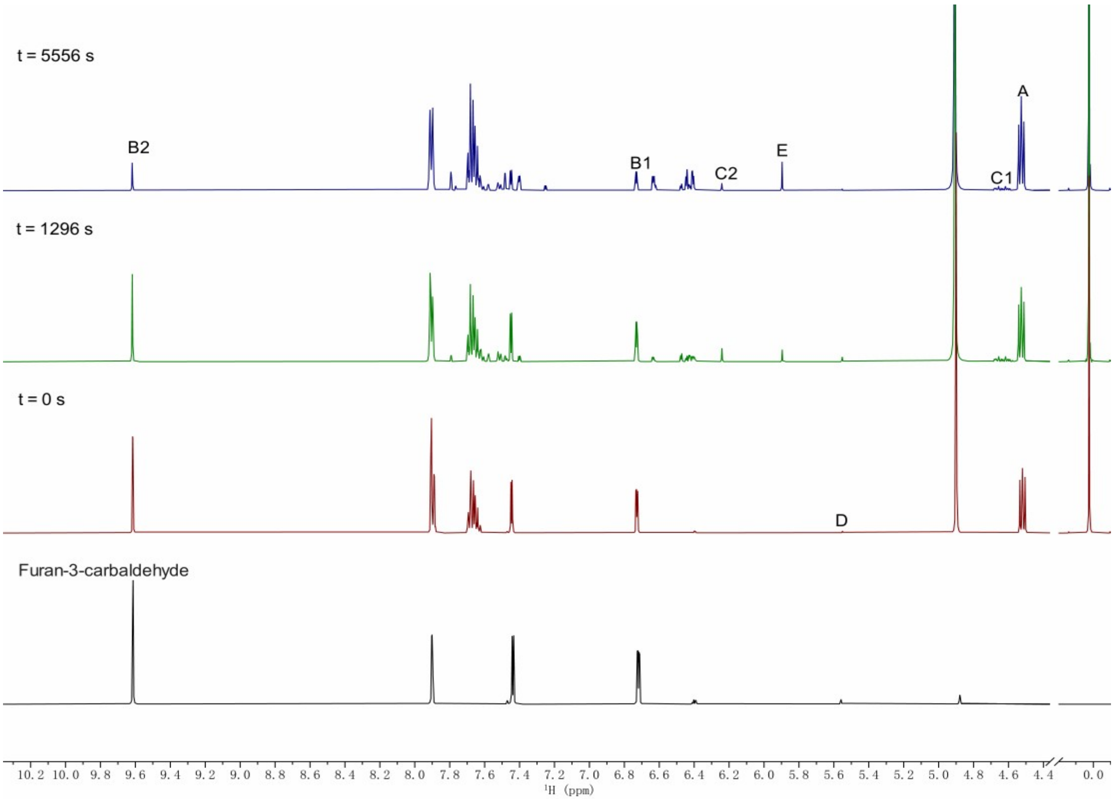

Figure S83. Representative ^1H NMR spectra (400 MHz) for reaction of furfural **48** (0.02 M) with *N*-Ph NHC precursor **39** (0.02 M) under a triethylamine buffer (NEt_3:NEt_3·HCl, 2:1, 0.09 M) in CD_3OD at 25 °C. A = NHC precursor NCH_2, B1 =Ar*H*CHO, B2 =ArHC*H*O, C1 = Adduct NCH_2, C2 =Adduct C(α)*H*, D = Hemiacetal C*H*, E = Benzoin C*H*.

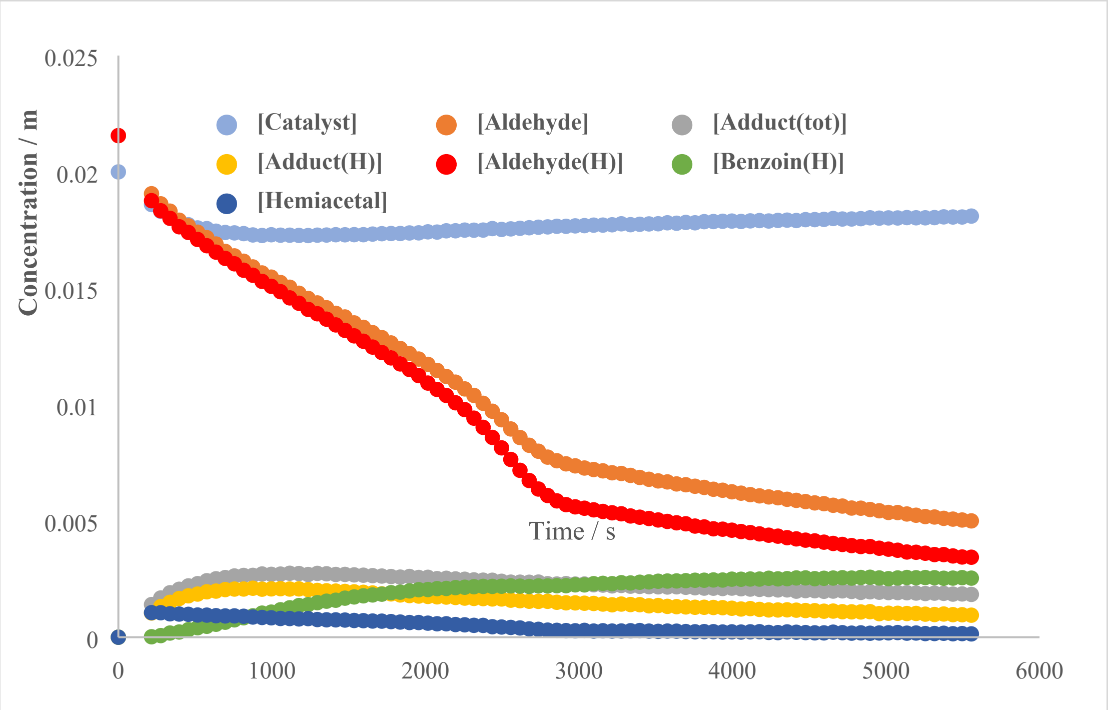

Figure S84. Concentration profile for the self-condensation of furfural **48** (0.02 M) with *N*-Ph NHC precursor **39** (0.02 M) under a triethylamine buffer (NEt_3:NEt_3·HCl, 2:1, 0.09 M) in CD_3OD at 25 °C.

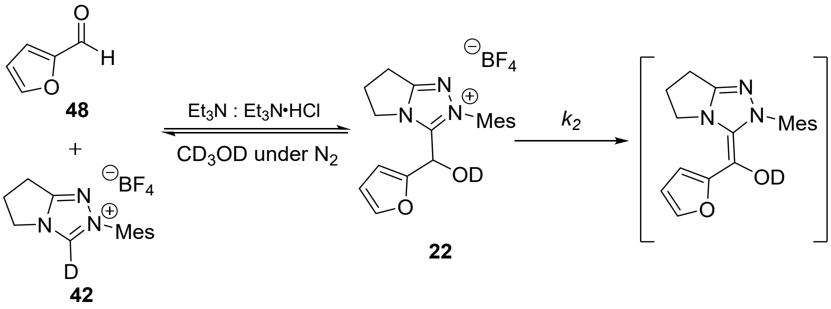

The reaction of furfural **48** and triazolium precatalyst **42** was monitored using ^{1}H NMR spectra, with representative NMR spectra over the course of the experiment given in Figure S85.

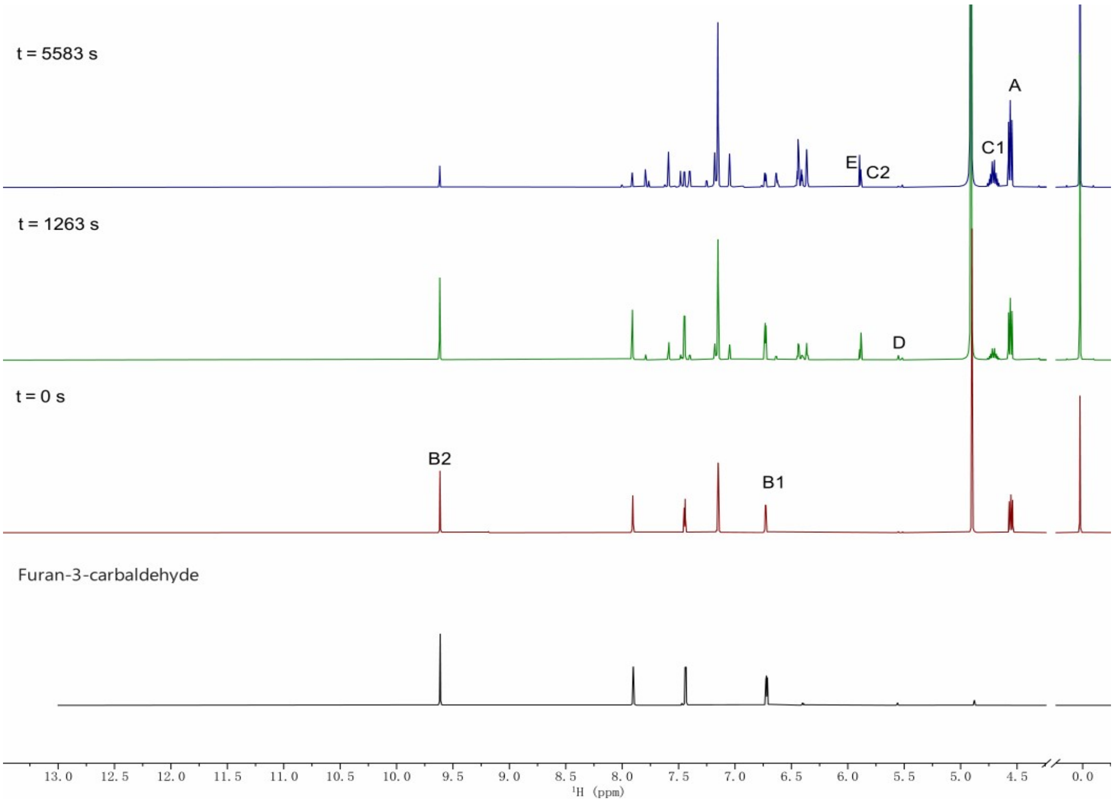

Figure S85. Representative ^{1}H NMR spectra (400 MHz) for reaction of furfural **48** (0.02 M) with *N*-Mes NHC precursor **42** (0.02 M) under a triethylamine buffer (NEt_3:NEt_3·HCl, 2:1, 0.09 M) in CD_3OD at 25 °C. A = NHC precursor NCH_2, B1 =Ar*H*CHO, B2 =ArHC*H*O, C1 = Adduct NC*H*$_2$, C2 =Adduct C(α)*H*, D = Hemiacetal C*H*, E = Benzoin C*H*.

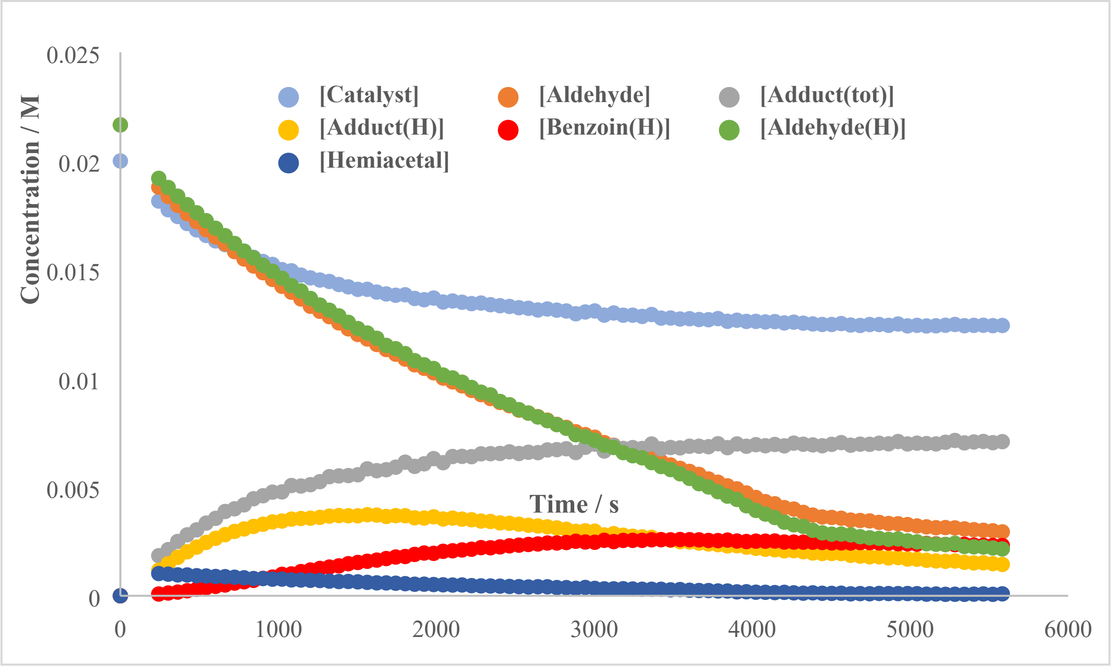

Figure S86. Concentration profile for the self-condensation of furfural **48** (0.02 M) with *N*-Mes NHC precursor **42** (0.02 M) under a triethylamine buffer (NEt_3:NEt_3·HCl, 2:1, 0.09 M) in CD_3OD at 25 °C.

4. Determination of Rate and Equilibrium Constants: Decay of 3-(Hydroxybenzyl)triazolium Adducts to Equilibrium

From experiments using an initial adduct concentration of 0.02 M, monitoring the initial decrease of adduct concentration at 25 °C, it was possible to determine the decay towards equilibrium. The equilibrium constant (K_{diss}, M) for this process is described by Equation **21**, assuming that $[\text{aldehyde(tot)}]_{eq} = [\text{NHC}]_{eq}$.

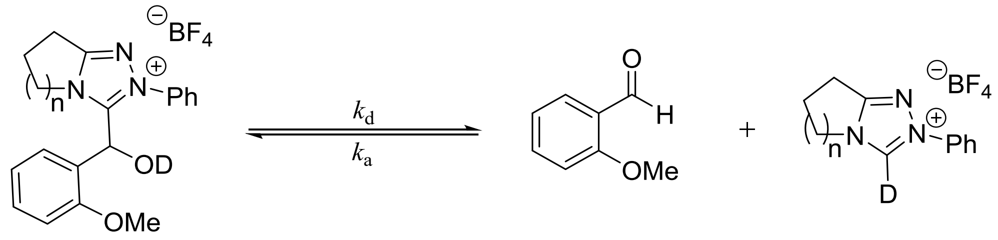

Scheme 4. Base catalysed 3-(hydroxybenzyl)triazolium salt equilibrium.

$$K_{diss} = \frac{k_d}{k_a} = \frac{[cat]_e \times [ald]_e}{[add\ (tot)]_e} = \frac{[cat]_e[ald(tot)]_e \times f_{ald}}{[add\ (tot)]_e}$$ **Equation 21**

The concentration of adduct can be expressed in Equation **22**, assuming [NHC] = [aldehyde(tot)], and may be written as in Equation **23**.

$$\frac{d[add]}{dt} = -\,k_d[add] + k_a[cat][ald]$$ **Equation 22**

$$\frac{d[add]}{dt} = -\,k_d[add] + k_a[cat][ald(tot)] \times f_{ald}$$ **Equation 23**

$$\frac{d[add]}{dt} = -\,k_d[add] + k_d\frac{[add]_e}{\left([add]_0 - [add]_e\right)^2}\left([add]_0 - [add]\right)^2$$ **Equation 24**

If the initial concentrations are $[add]_0 = a$ and $[cat]_0 = [ald]_0 = 0$, then Equation **3** may be integrated to give the rate equation shown in Equation **24**, where $x = ([add]_0 – [add])$ and $x_e = ([add]_0 – [add]_e)$. Thus, a value of k_d may be obtained from the slope of a semilogarithmic plot of $[(ax_e + x(a – x_e))/(a(x_e – x))]$ against time.

$$\frac{x_e}{(2a - x_e)}\ln\frac{ax_e + x(a - x_e)}{a(x_e - x)} = k_d t$$ **Equation 25**

Table 2 Entry 1

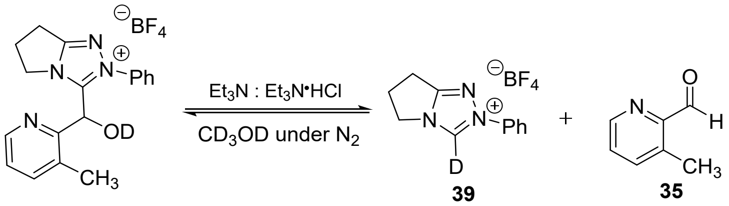

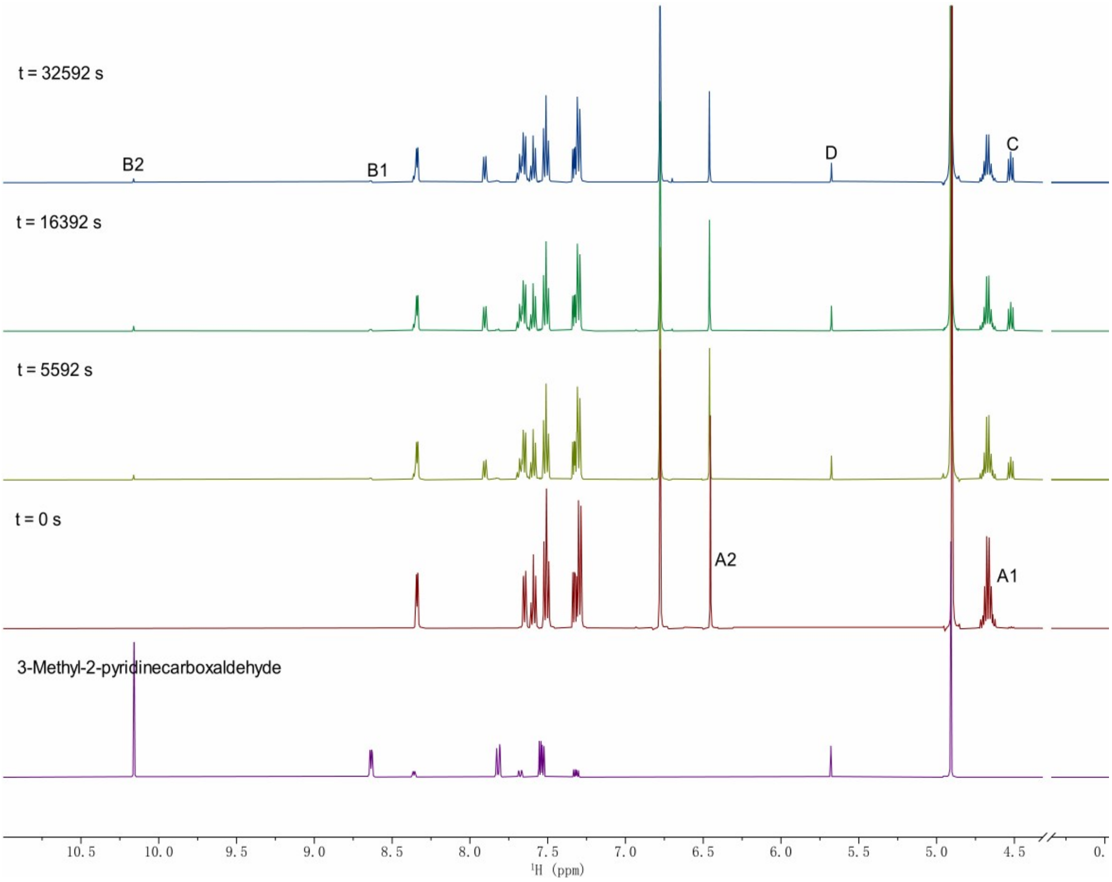

Figure S87. ^{1}H NMR spectra for dissociation of 3-(hydroxybenzyl)triazolium adduct **12** (0.02 M) under a triethylamine buffer (NEt_3:NEt_3·HCl, 2:1, 0.09 M) in CD_3OD at 25 °C. A1 = Adduct NCH_2, A2 = Adduct C(α)H, B1 =ArHCHO, B2 =ArHCHO, C = NHC precursor NCH_2, D = Hemiacetal CH.

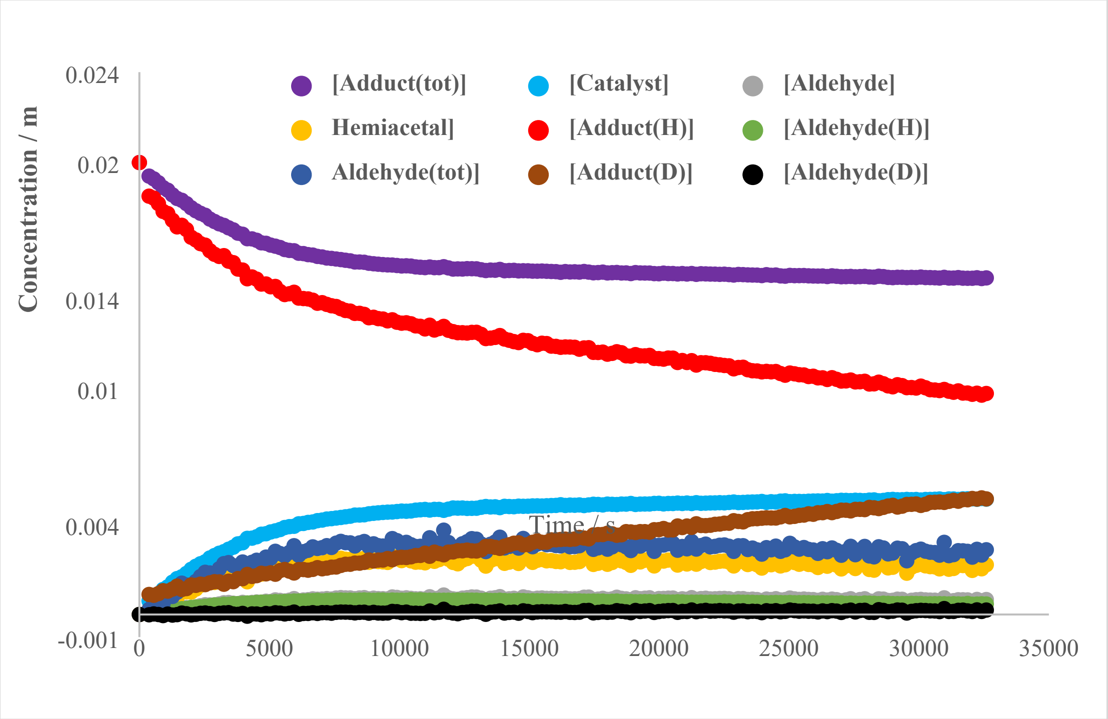

Figure S88. Reaction profile for dissociation of 3-(hydroxybenzyl)triazolium adduct **12** (0.02 M) under a triethylamine buffer (NEt_3:NEt_3·HCl, 2:1, 0.09 M) in CD_3OD at 25 °C.

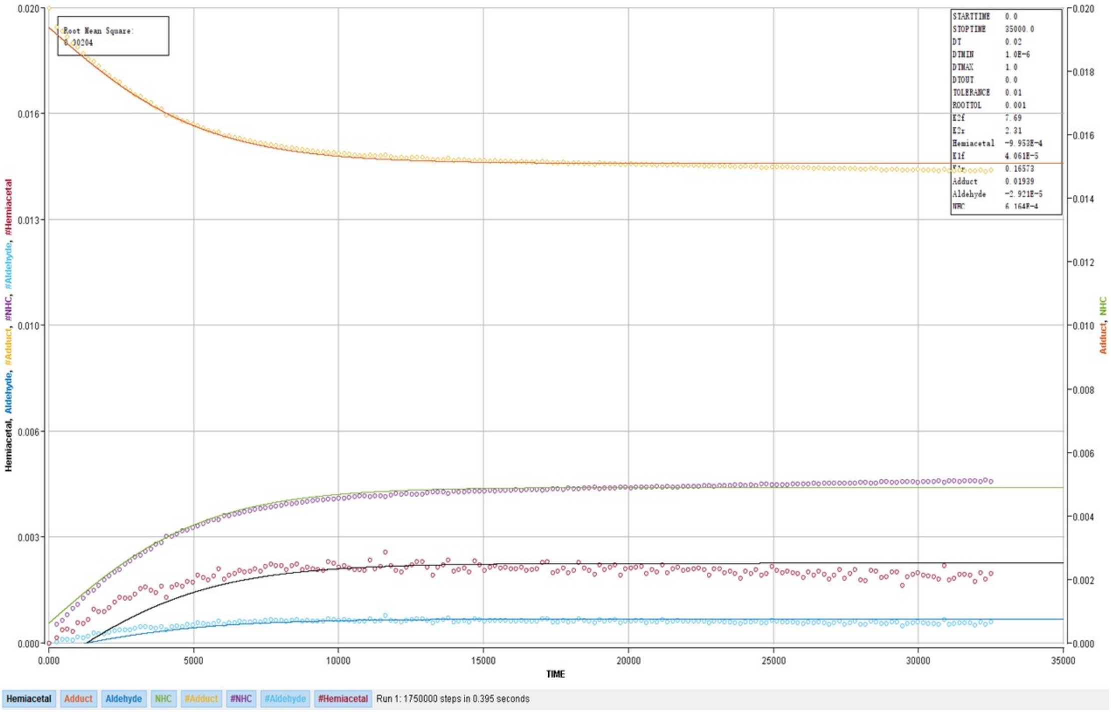

Figure S89. Plot showing the reaction of 3-(hydroxybenzyl)triazolium adduct **12** (0.02 M) under a triethylamine buffer (NEt_3:NEt_3·HCl, 2:1, 0.09 M) in CD_3OD at 25 °C up to the equilibrium concentrations. Open circles show the experimental data, with the solid line representing the fit to the kinetic model. Fitting data from t = 0 to t = 25000 s from Figure S84.

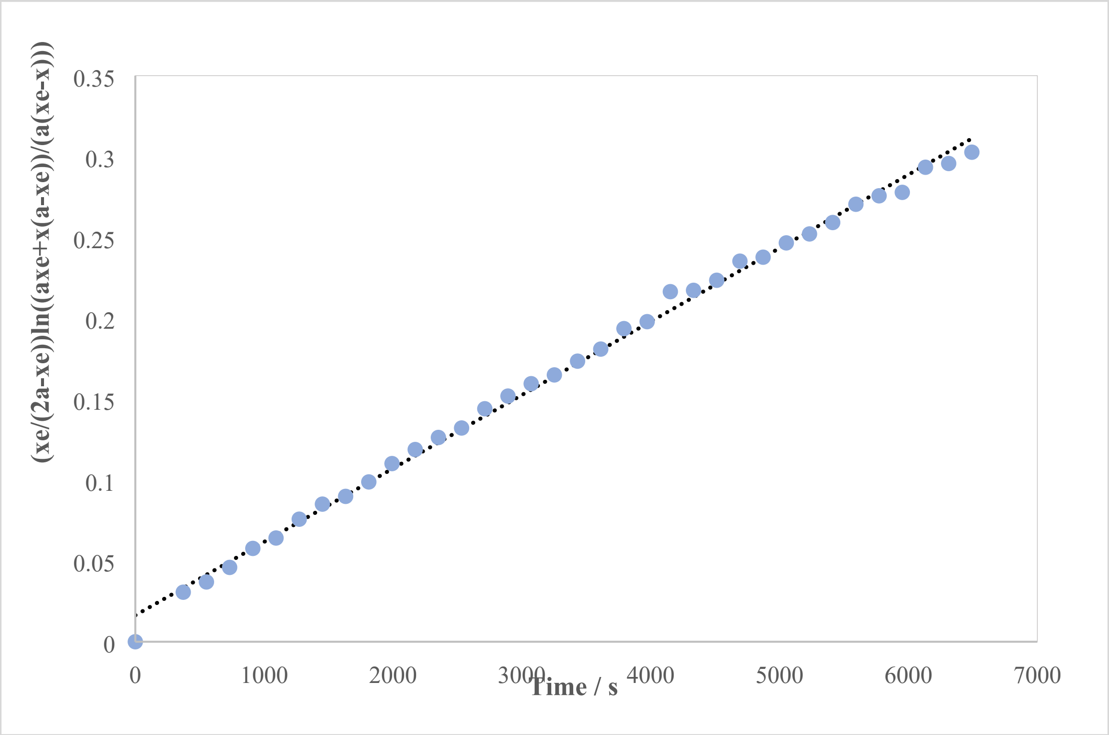

Figure S90. Semilogarithmic plots of $(ax_e+x(a–x_e))/(a(x_e–x))$ against time, obtained from dissociation of 3-(hydroxybenzyl)triazolium adduct **12** (0.02 M) under a triethylamine buffer (NEt_3:NEt_3·HCl, 2:1, 0.09 M) in CD_3OD at 25 °C.

Table 2 Entry 2

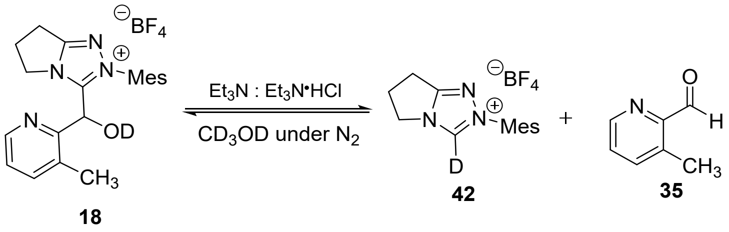

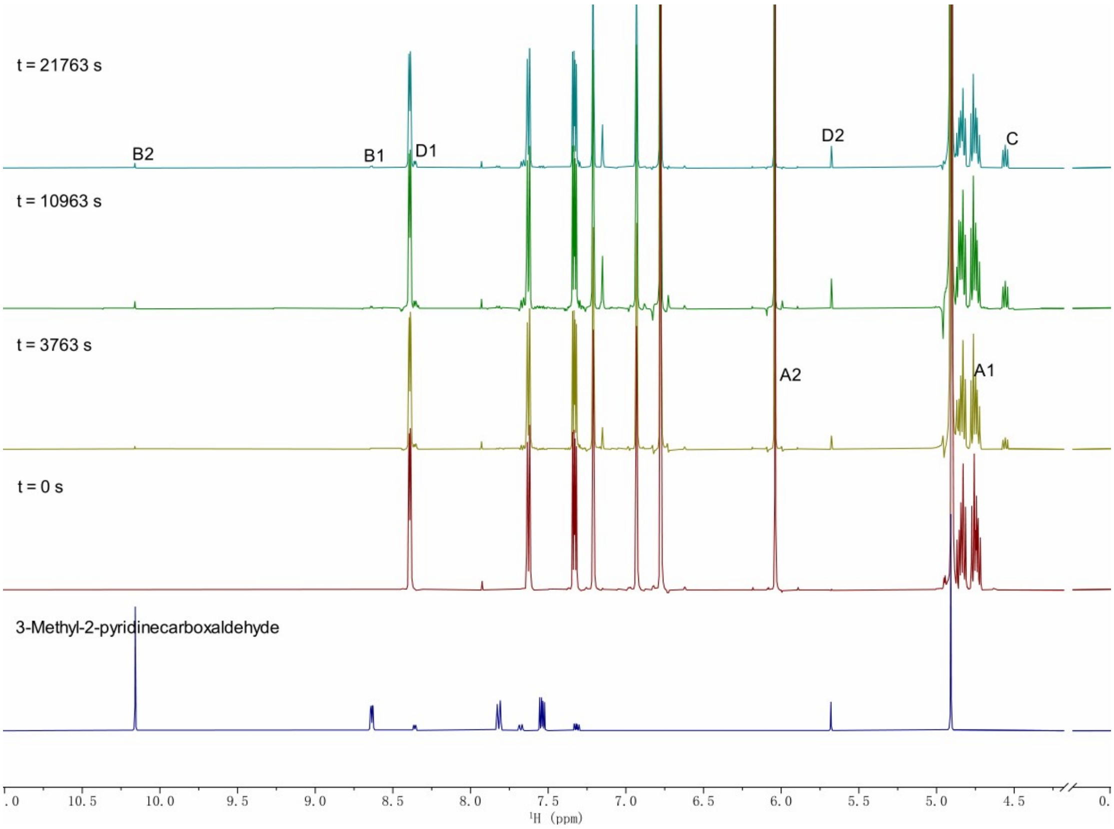

Figure S91. ^{1}H NMR spectra for dissociation of 3-(hydroxybenzyl)triazolium adduct **18** (0.02 M) under a triethylamine buffer (NEt$_3$:NEt$_3$·HCl, 2:1, 0.09 M) in CD$_3$OD at 25 °C. A1 = Adduct NCH_AH$_B$, A2 = Adduct C(α)H, B1 =ArHCHO, B2 =ArHCHO, C = NHC precursor NCH_2, D1 = Hemiacetal ArH, D2 = Hemiacetal CH.

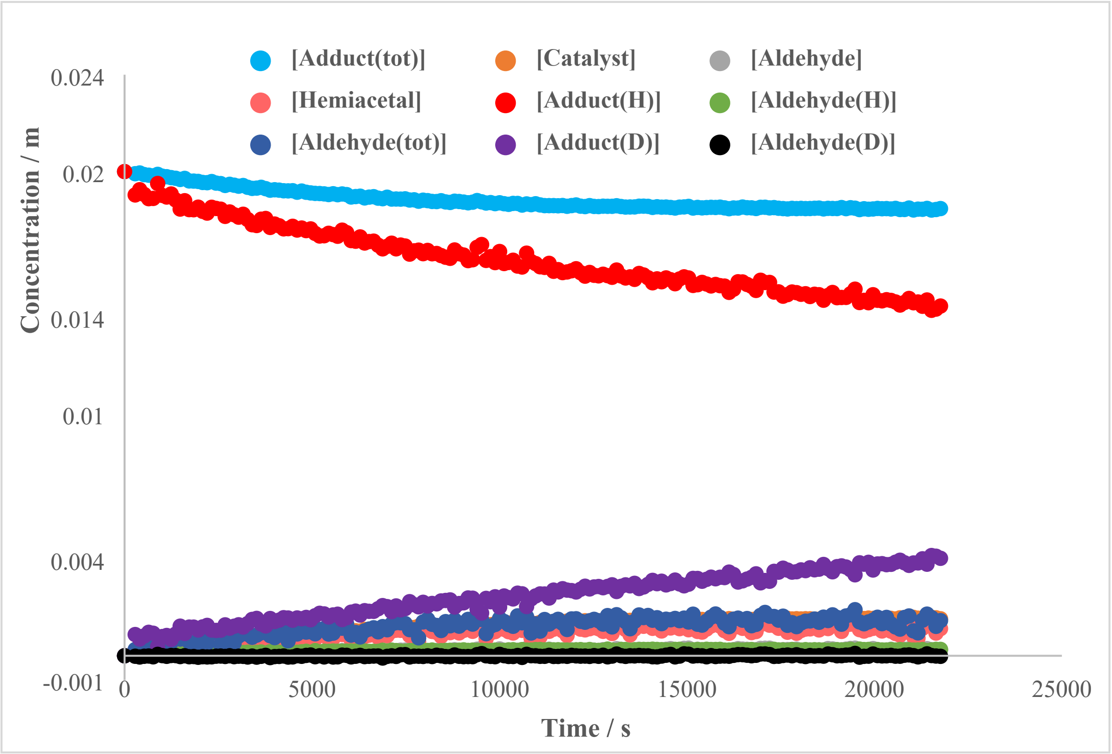

Figure S92. Reaction profile for dissociation of 3-(hydroxybenzyl)triazolium adduct **18** (0.02 M) under a triethylamine buffer (NEt_3:NEt_3·HCl, 2:1, 0.09 M) in CD_3OD at 25 °C.

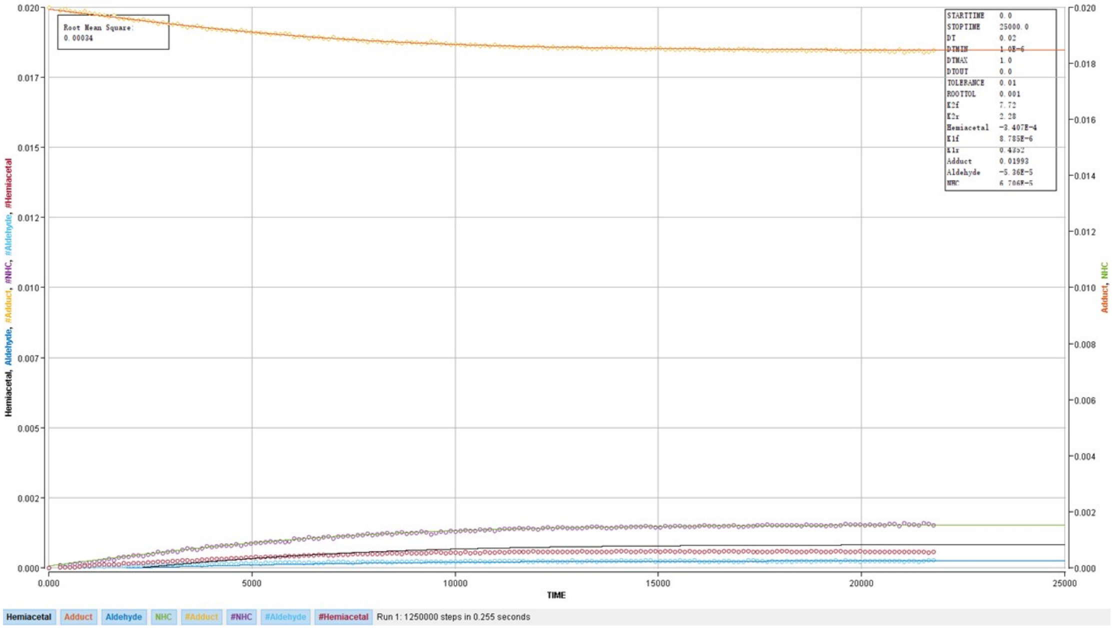

Figure S93. Plot showing the reaction of 3-(hydroxybenzyl)triazolium adduct **18** (0.02 M) under a triethylamine buffer (NEt_3:NEt_3·HCl, 2:1, 0.09 M) in CD_3OD at 25 °C. up to the equilibrium concentrations. Open circles show the experimental data, with the solid line representing the fit to the kinetic model. Fitting data from t = 0 to t = 25000 s from Figure S88.

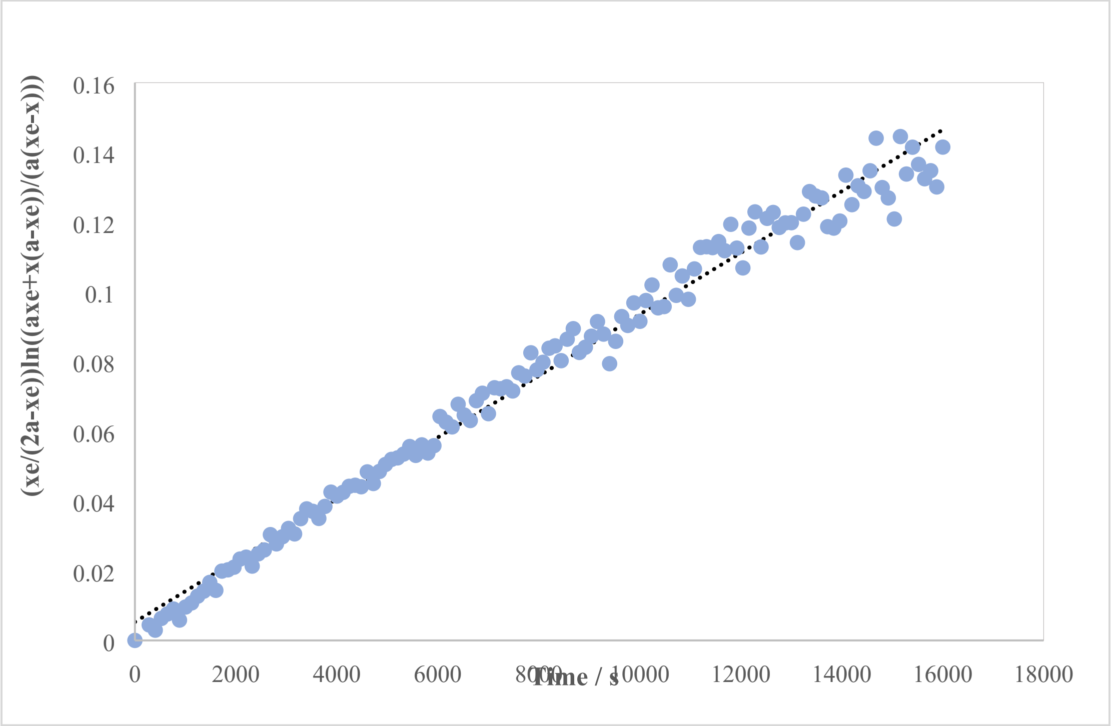

Figure S94. Semilogarithmic plots of $(ax_e+x(a–x_e))/(a(x_e–x))$ against time, obtained from dissociation of 3-(hydroxybenzyl)triazolium adduct **18** (0.02 M) under a triethylamine buffer

(NEt_3:NEt_3·HCl, 2:1, 0.09 M) in CD_3OD at 25 °C.

Table 2 Entry 3

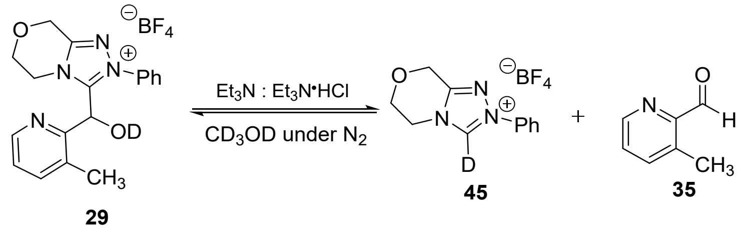

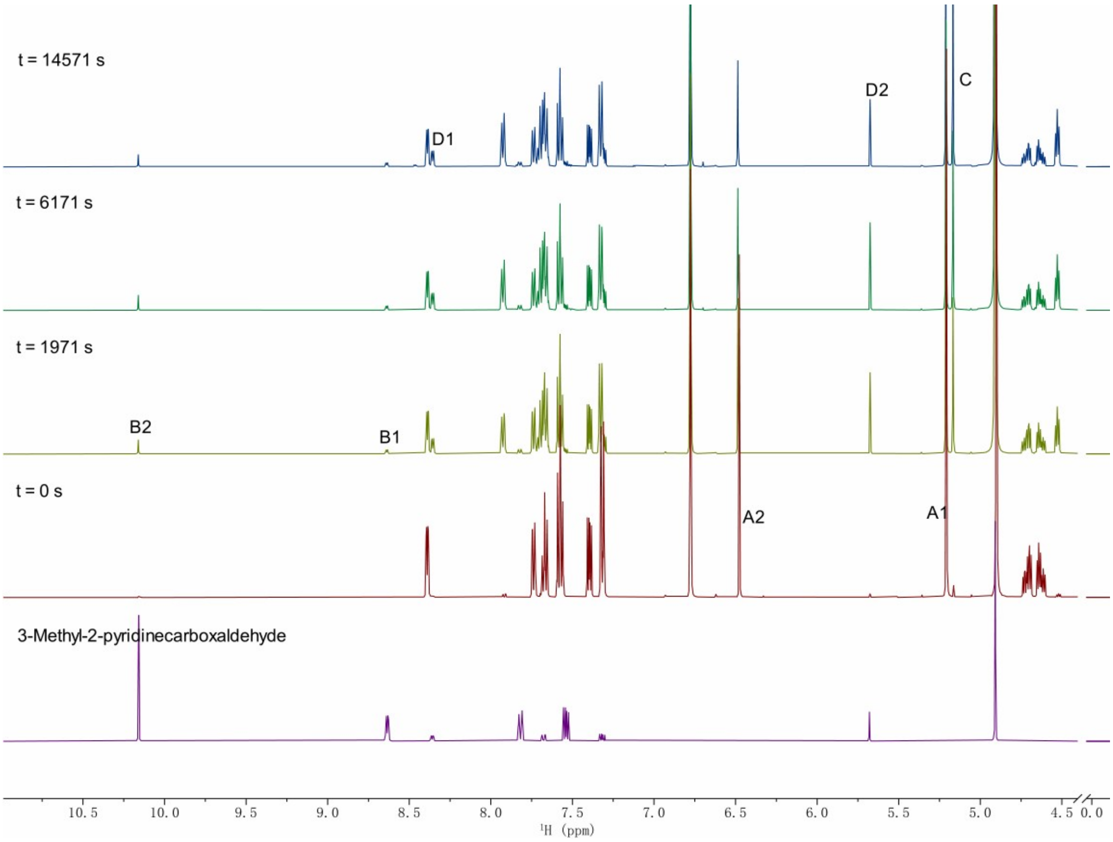

Figure S95. ^{1}H NMR spectra for dissociation of 3-(hydroxybenzyl)triazolium adduct **29** (0.02 M) under a triethylamine buffer (NEt_3:NEt_3·HCl, 2:1, 0.09 M) in CD_3OD at 25 °C. A1 = Adduct OCH_2C=, A2 = Adduct C(α)H, B1 =ArHCHO, B2 =ArHCHO, C = NHC precursor OCH_2C=, D1 = Hemiacetal ArH, D2 = Hemiacetal CH.

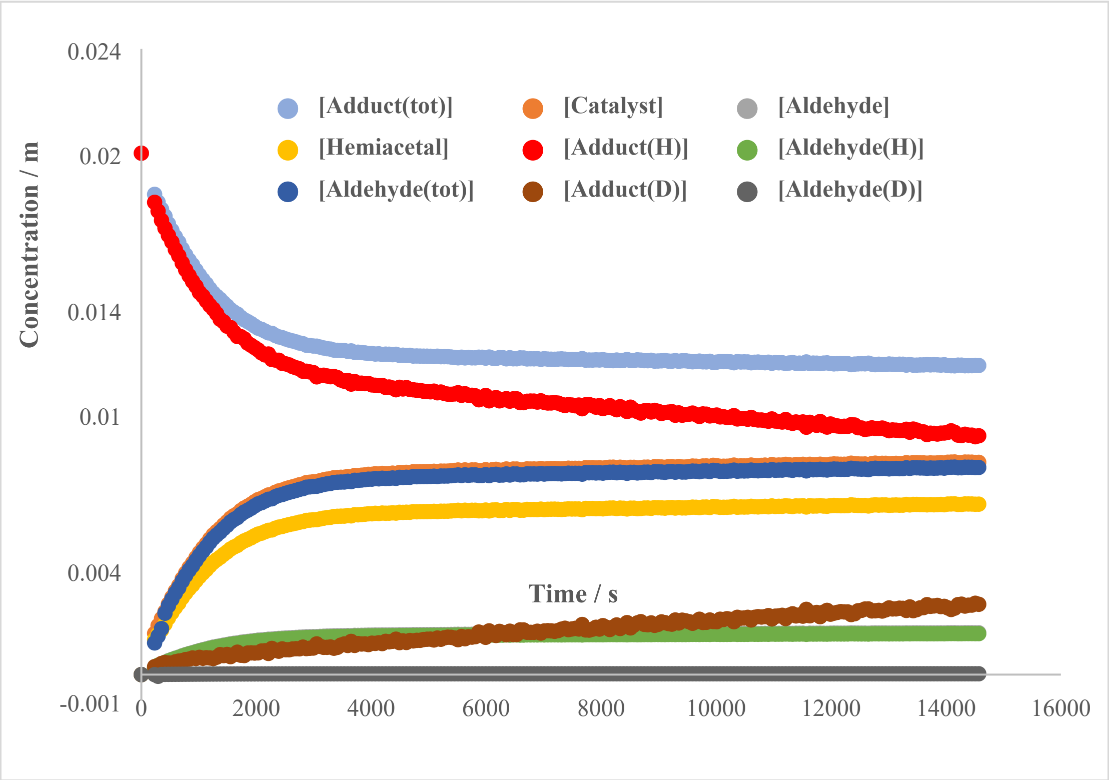

Figure S96. Reaction profile for dissociation of 3-(hydroxybenzyl)triazolium adduct **29** (0.02 M) under a triethylamine buffer (NEt_3:NEt_3·HCl, 2:1, 0.09 M) in CD_3OD at 25 °C.

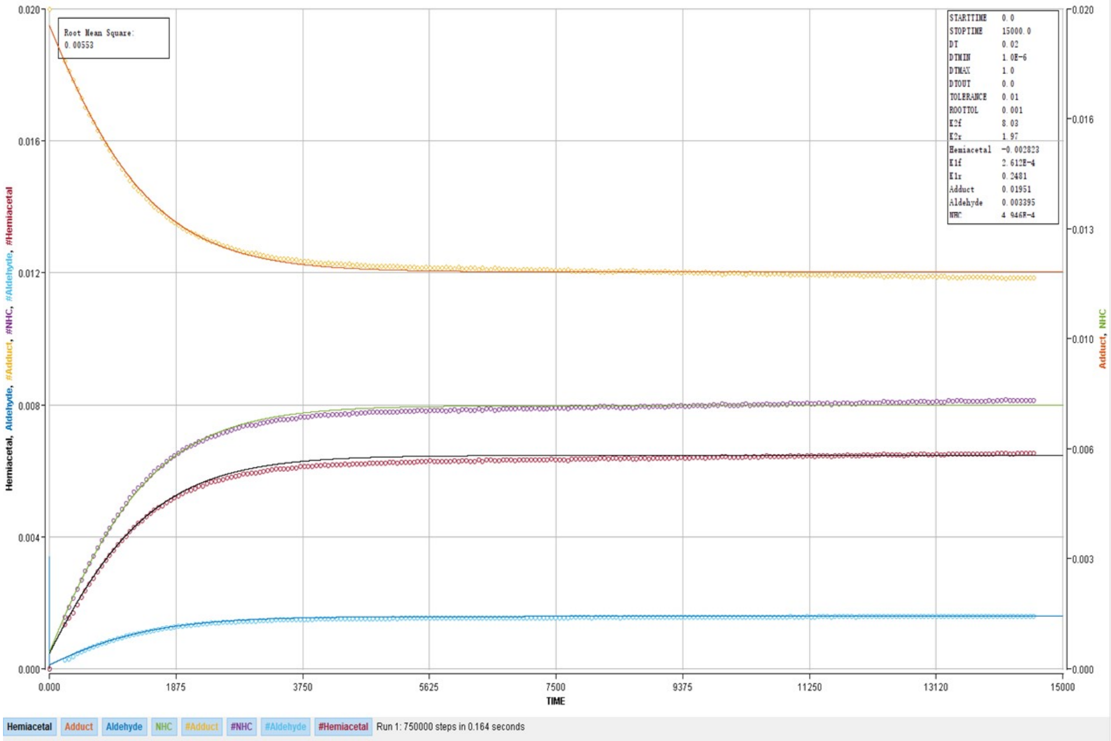

Figure S97. Plot showing the reaction of 3-(hydroxybenzyl)triazolium adduct **29** (0.02 M) under a triethylamine buffer (NEt_3:NEt_3·HCl, 2:1, 0.09 M) in CD_3OD at 25 °C up to the equilibrium concentrations. Open circles show the experimental data, with the solid line representing the fit to the kinetic model. Fitting data from t = 0 to t = 15000 s from Figure S92.

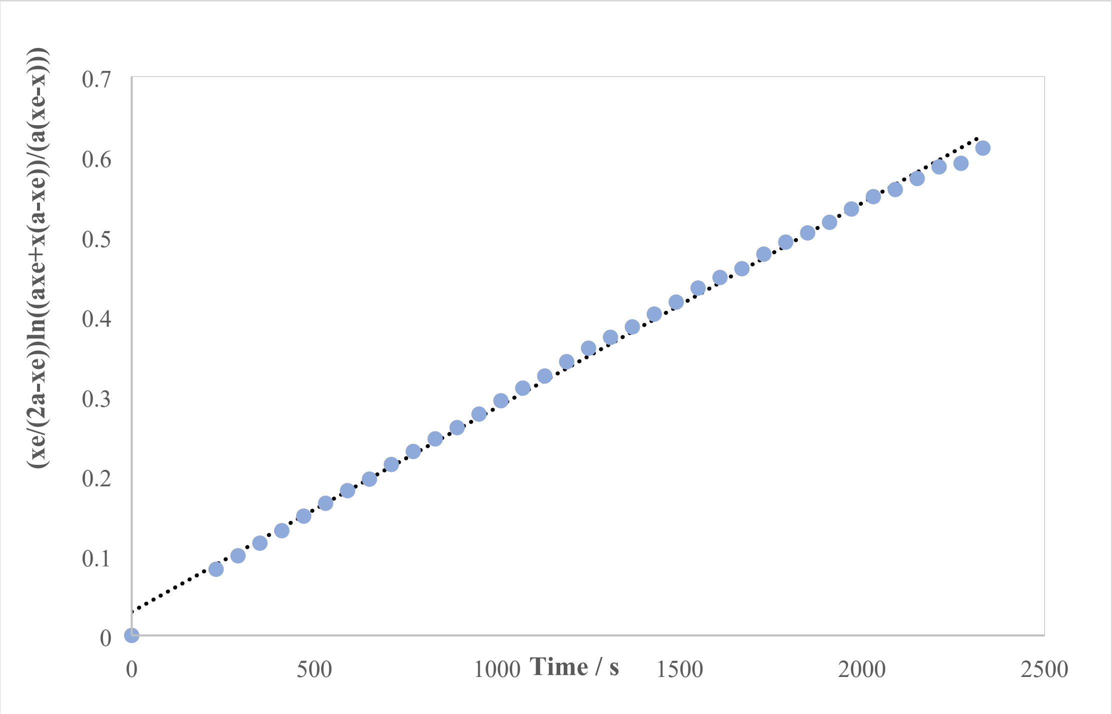

Figure S98. Semilogarithmic plots of $(ax_e+x(a-x_e))/(a(x_e-x))$ against time, obtained from dissociation of 3-(hydroxybenzyl)triazolium adduct **29** (0.02 M) under a triethylamine buffer (NEt_3:NEt_3·HCl, 2:1, 0.09 M) in CD_3OD at 25 °C.

Table 2 Entry 4

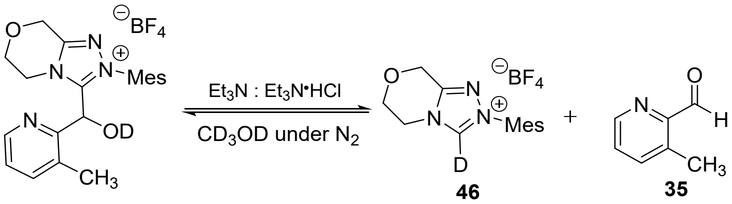

30 **46** **35**

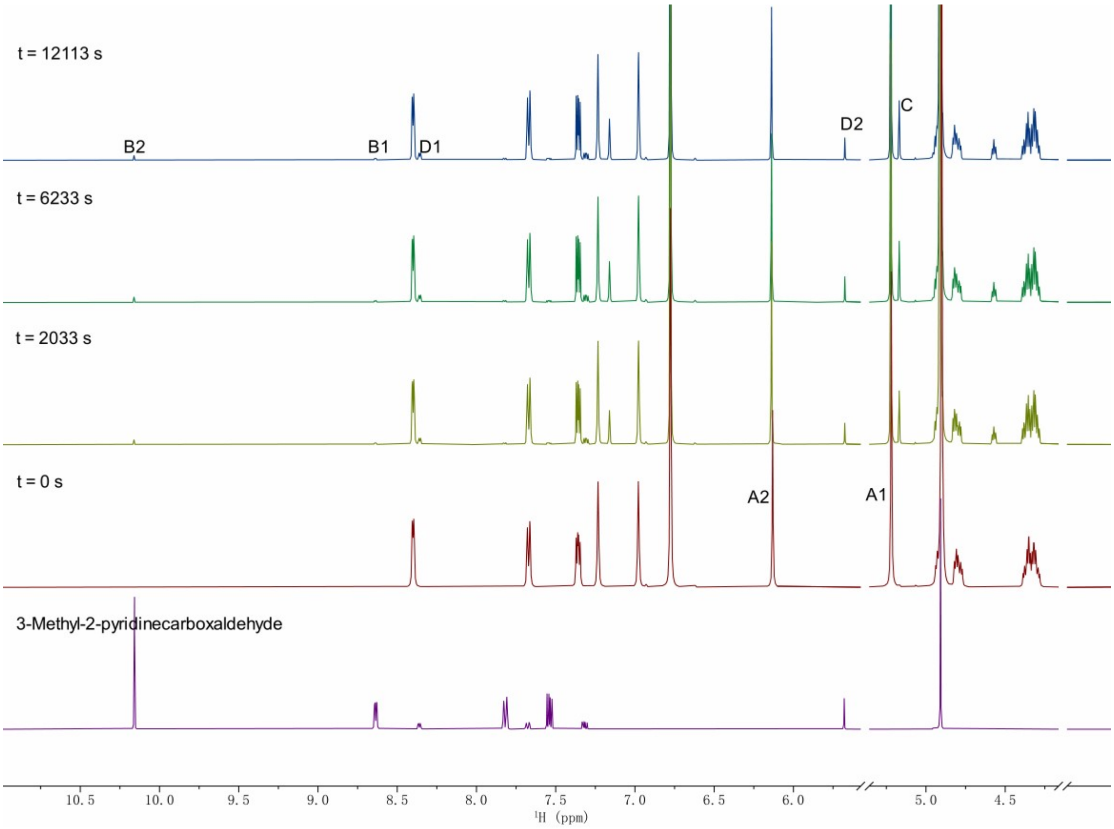

Figure S99. ^{1}H NMR spectra for dissociation of 3-(hydroxybenzyl)triazolium adduct **30** (0.018 M) under a triethylamine buffer (NEt_3:NEt_3·HCl, 2:1, 0.09 M) in CD_3OD at 25 °C. A1 = Adduct OCH_2C=, A2 = Adduct C(α)*H*, B1 =Ar*H*CHO, B2 =ArHC*H*O, C = NHC precursor OCH_2C=, D1 = Hemiacetal Ar*H*, D2 = Hemiacetal C*H*.

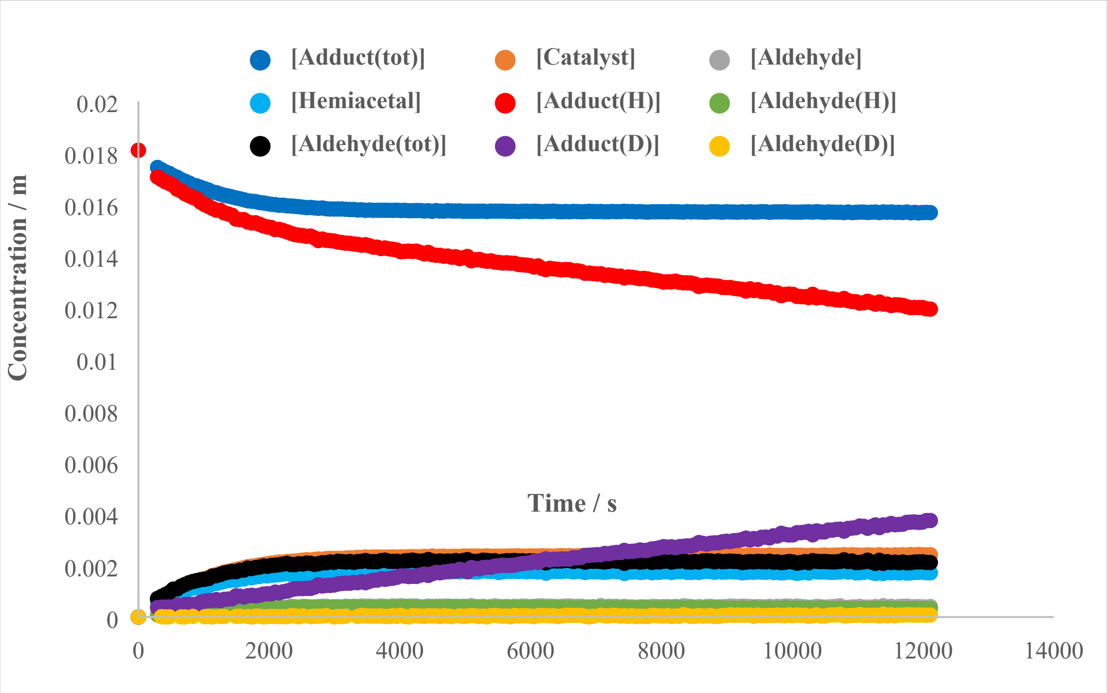

Figure S100. Reaction profile for dissociation of 3-(hydroxybenzyl)triazolium adduct **30** (0.018 M) under a triethylamine buffer (NEt_3:NEt_3·HCl, 2:1, 0.09 M) in CD_3OD at 25 °C.

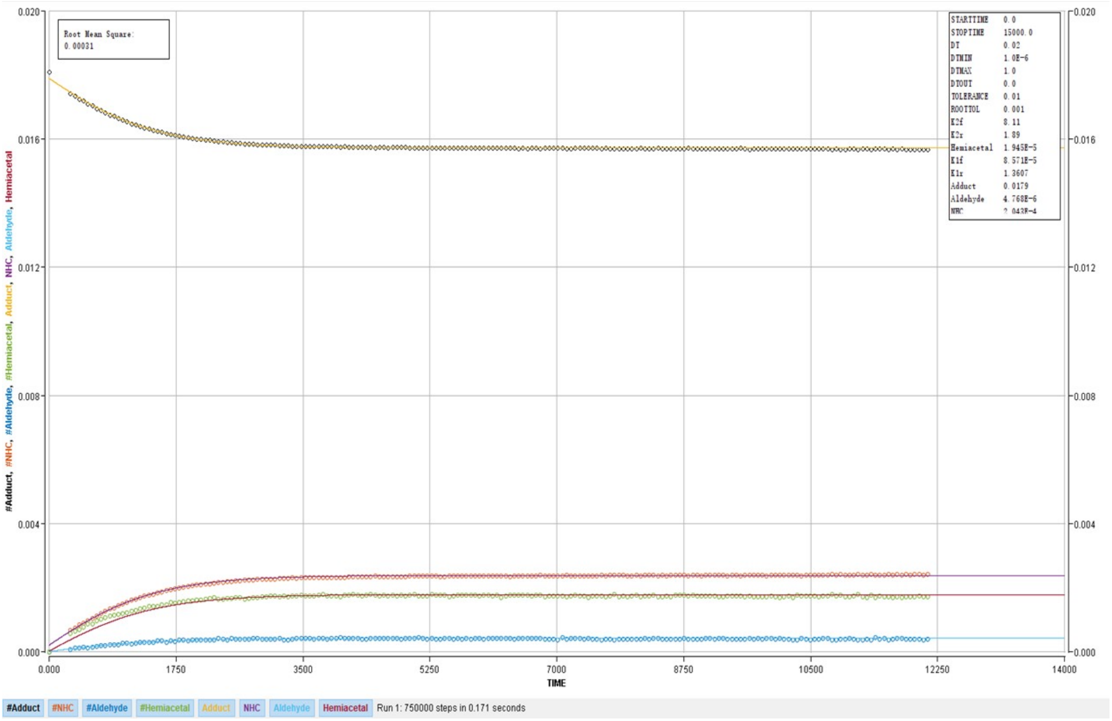

Figure S101. Plot showing the reaction of 3-(hydroxybenzyl)triazolium adduct **30** (0.018 M) under a triethylamine buffer (NEt_3:NEt_3·HCl, 2:1, 0.09 M) in CD_3OD at 25 °C up to the equilibrium concentrations. Open circles show the experimental data, with the solid line representing the fit to the kinetic model. Fitting data from t = 0 to t = 14000 s from Figure S96.

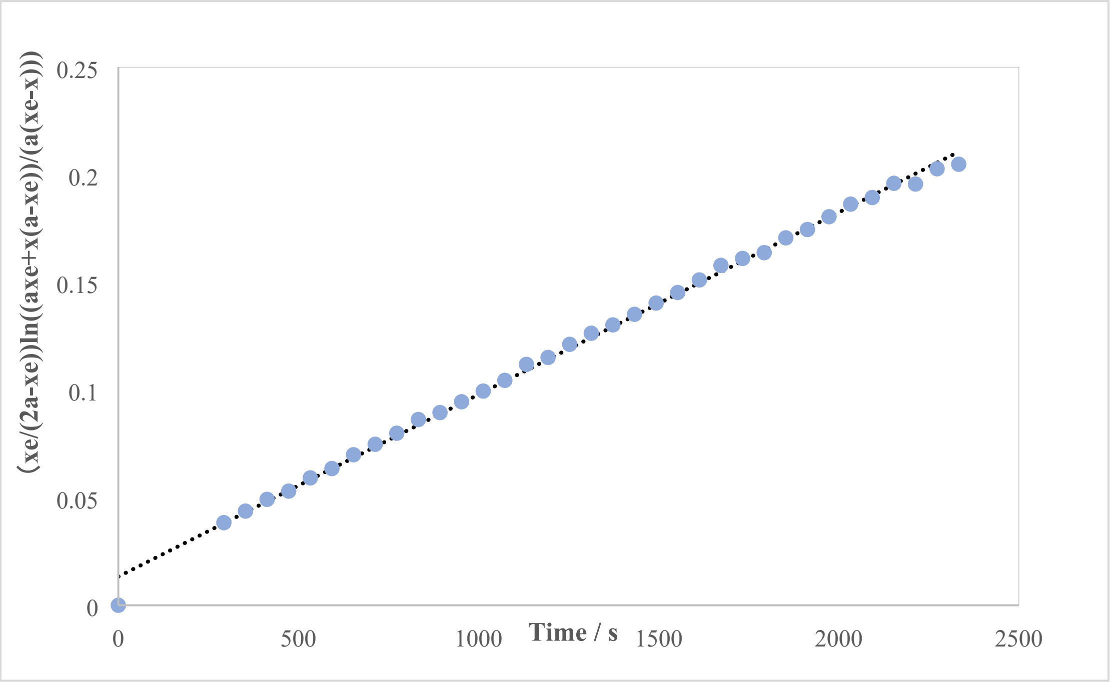

Figure S102. Semilogarithmic plots of $(ax_e+x(a–x_e))/(a(x_e–x))$ against time, obtained from dissociation of 3-(hydroxybenzyl)triazolium adduct **30** (0.018 M) under a triethylamine buffer (NEt_3:NEt_3·HCl, 2:1, 0.09 M) in CD_3OD at 25 °C.

X-ray Crystal Structure

X-ray diffraction data were collected at 173 K using a Rigaku Rigaku SCX mini diffractometer using graphite monochromated Mo-Ka radiation (λ = 0.71075 Å). Data were collected using CrystalClear[13] and processed (including correction for Lorentz, polarization and absorption) using CrysAlisPro.[14] Structures were solved by dual-space (SHELXT[15]), direct (SIR2011[16]) or charge-flipping (Superflip[17]) methods and refined by full-matrix least-squares against F^2 (SHELXL-2018/3[18]). Non-hydrogen atoms were refined anisotropically, and all hydrogen atoms were refined using a riding model. All calculations were performed using the CrystalStructure[19] interface.

3-(hydroxybenzyl)triazolium adduct **13**	
CCDC	2209647
empirical formula	$C_{17}H_{16}BBrF_4N_4O$
fw	459.0496
crystal description	colourless prism
crystal size [mm]	0.18×0.14×0.03
space group	$P2_12_12_1$(#19)
a [Å]	9.7229(6)
b [Å]	11.8712(8)
c [Å]	15.4592(10)
vol [Å]^3	1784.3(2)
Z	4
ρ (calc) [g/cm^3]	1.709
μ [cm^{-1}]	23.670
F(000)	920.00
reflections collected	18551
independent reflections (R_{int})	4098 (0.0515)
data/parameters	4098/257
GOF on F^2	0.97
R_1 [$I > 2\sigma(I)$]	0.0301
wR_2 (all data)	0.0626
largest diff. peak/hole [e/Å^3]	0.43, −0.41
Flack parameter	−0.015(5)

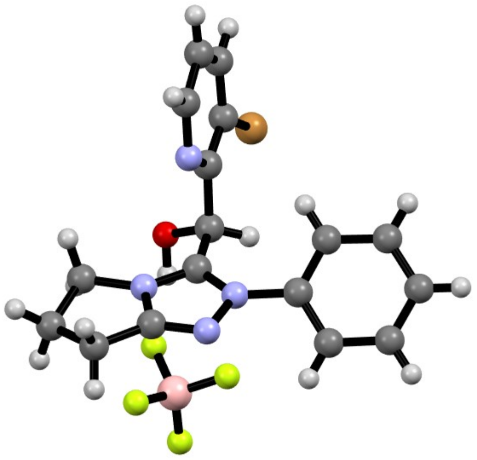

References

1. C. J. Collett, R. S. Massey, J. E. Taylor, O. R. Maguire, A. C. O'Donoghue and A. D. Smith, *Angewandte Chemie International Edition*, 2015, **54**, 6887-6892.
2. J. Zhu, Durham University, 2019.
3. M. S. Kerr, J. Read de Alaniz and T. Rovis, *The Journal of Organic Chemistry*, 2005, **70**, 5725-5728.
4. S. M. Langdon, M. M. D. Wilde, K. Thai and M. Gravel, *Journal of the American Chemical Society*, 2014, **136**, 7539-7542.
5. R. S. Massey, J. Murray, C. J. Collett, J. Zhu, A. D. Smith and A. C. O'Donoghue, *Organic & Biomolecular Chemistry*, 2021, **19**, 387-393.
6. N. Konstandaras, M. H. Dunn, M. S. Guerry, C. D. Barnett, M. L. Cole and J. B. Harper, *Organic & Biomolecular Chemistry*, 2020, **18**, 66-75.
7. K. B. Ling and A. D. Smith, *Chemical Communications*, 2011, **47**, 373-375.
8. J. Zhu, I. Moreno, P. Quinn, D. S. Yufit, L. Song, C. M. Young, Z. Duan, A. R. Tyler, P. G. Waddell, M. J. Hall, M. R. Probert, A. D. Smith and A. C. O'Donoghue, *J Org Chem*, 2022, **87**, 4241-4253.
9. C. J. Collett, R. S. Massey, J. E. Taylor, O. R. Maguire, A. C. O'Donoghue and A. D. Smith, *Angew Chem Weinheim Bergstr Ger*, 2015, **127**, 6991-6996.
10. R. Breslow and E. McNelis, *Journal of the American Chemical Society*, 1959, **81**, 3080-3082.
11. J. Henrique Teles, J.-P. Melder, K. Ebel, R. Schneider, E. Gehrer, W. Harder, S. Brode, D. Enders, K. Breuer and G. Raabe, *Helvetica Chimica Acta*, 1996, **79**, 61-83.
12. M. J. White and F. J. Leeper, *The Journal of Organic Chemistry*, 2001, **66**, 5124-5131.
13. CrystalClear-SM Expert v2.1. Rigaku Americas, The Woodlands, Texas, USA, and Rigaku Corporation, Tokyo, Japan, **2015**.
14. CrysAlisPro v1.171.38.46. Rigaku Oxford Diffraction, Rigaku Corporation, Oxford, U.K. **2015**.
15. G. M. Sheldrick, *Acta Crystallogr., Sect. A.* **2015**, *71*, 3–8.
16. M. C. Burla, R. Caliandro, M. Camalli, B. Carrozzini, G. L. Cascarano, C. Giacovazzo, M. Mallamo, A. Mazzone, G. Polidori, R. Spagna, *J. Appl. Cryst.* **2012**, *45*,357-361.
17. L. Palatinus, G. Chapuis, *J. Appl. Cryst.* **2007**, *40*, 786–790.
18. G. M. Sheldrick, *Acta Crystallogr., Sect. C.* **2015**, *71*, 3–8.
19. CrystalStructure v4.3.0. Rigaku Americas, The Woodlands, Texas, USA, and Rigaku Corporation, Tokyo, Japan, **2018**.

^{1}H, $^{13}C\{^{1}H\}$ and $^{19}F\{^{1}H\}$ NMR Spectra

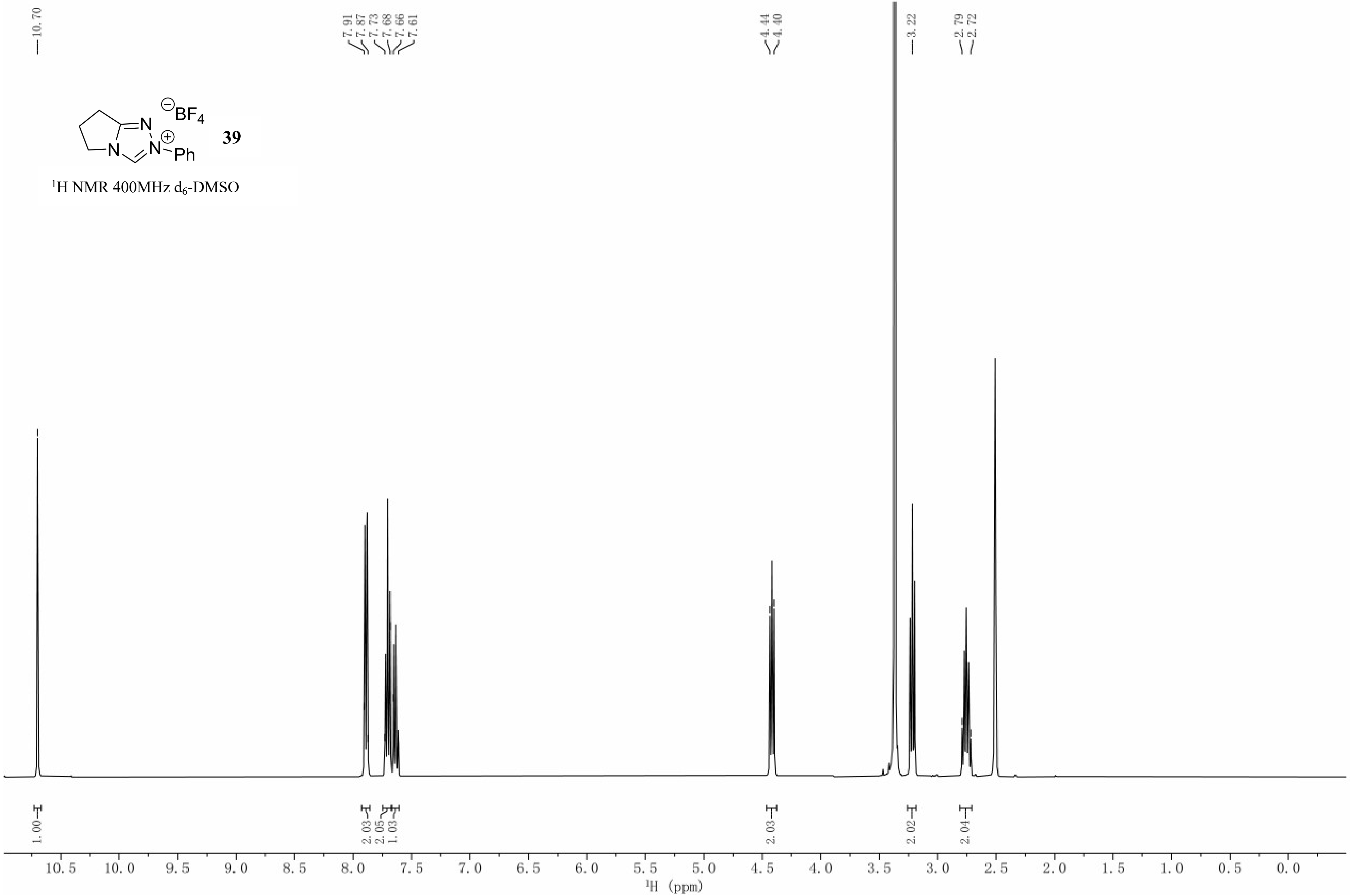

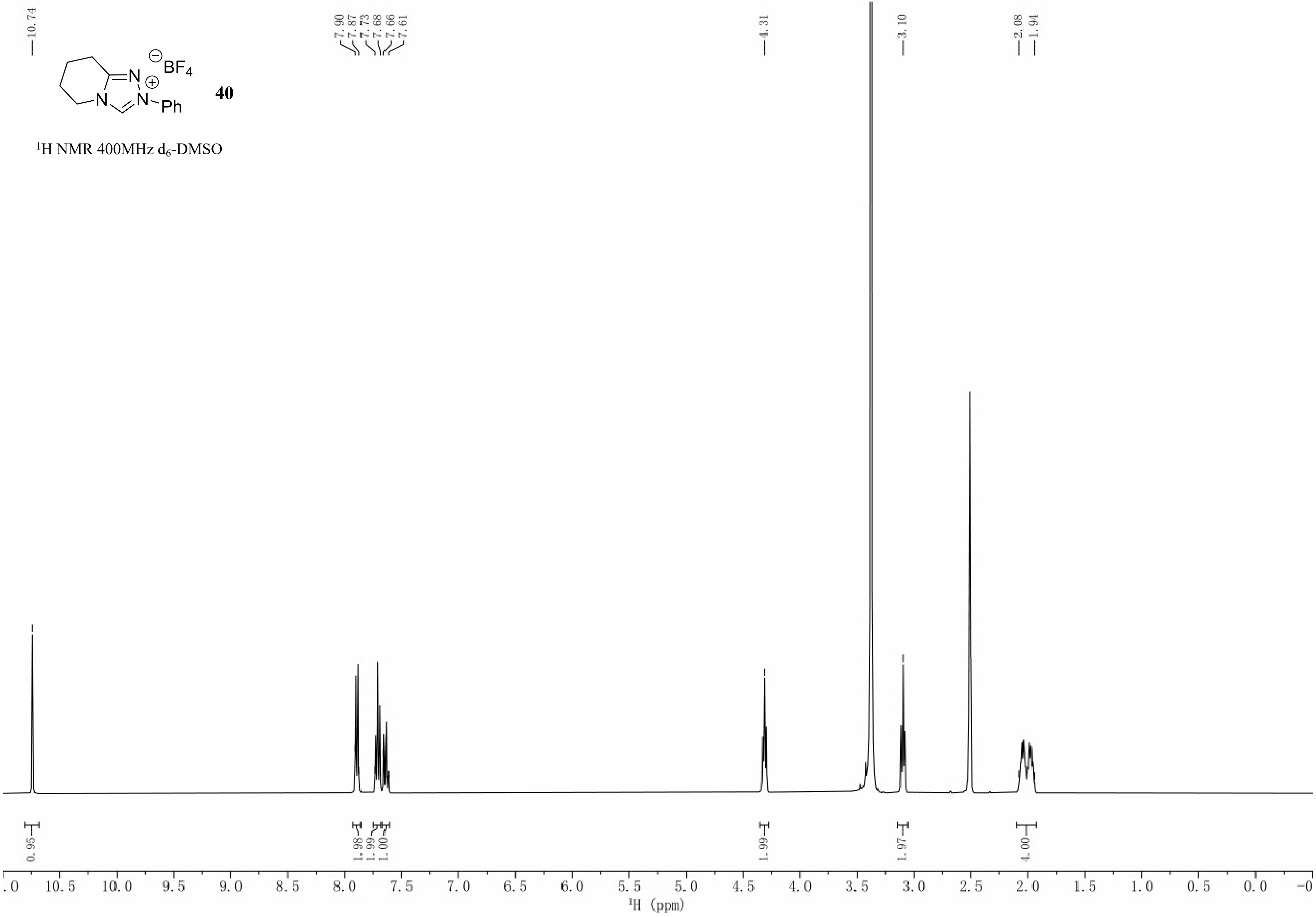

40

^{1}H NMR 400MHz d_6-DMSO

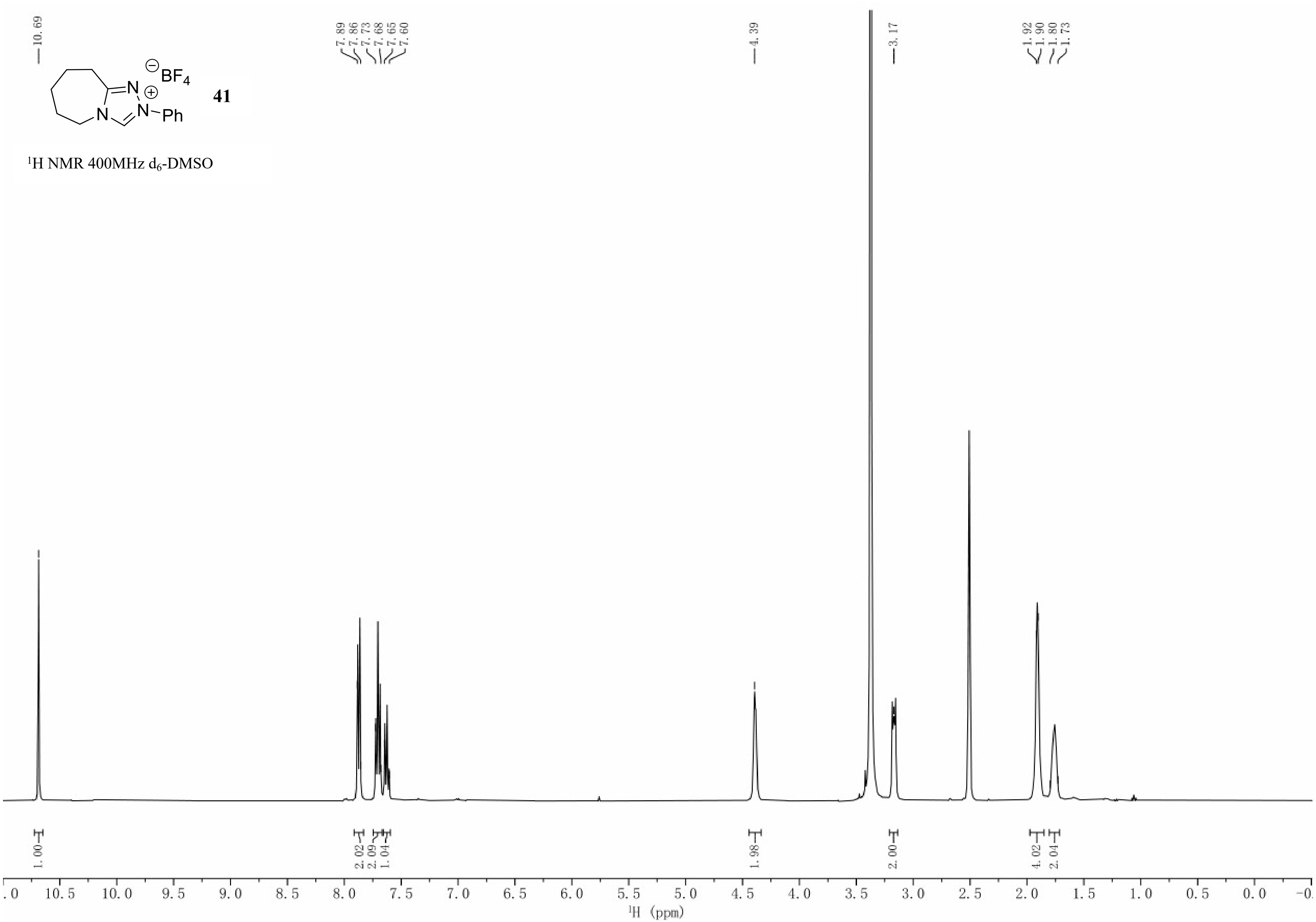

BF4
N
N
N
Ph
41
1H NMR 400MHz d6-DMSO
10.69
7.89
7.86
7.73
7.68
7.65
7.60
4.39
3.17
1.92
1.90
1.80
1.73
1.00
2.02
2.09
1.04
1.98
2.00
4.02
2.04
10.5 10.0 9.5 9.0 8.5 8.0 7.5 7.0 6.5 6.0 5.5 5.0 4.5 4.0 3.5 3.0 2.5 2.0 1.5 1.0 0.5 0.0
1H (ppm)

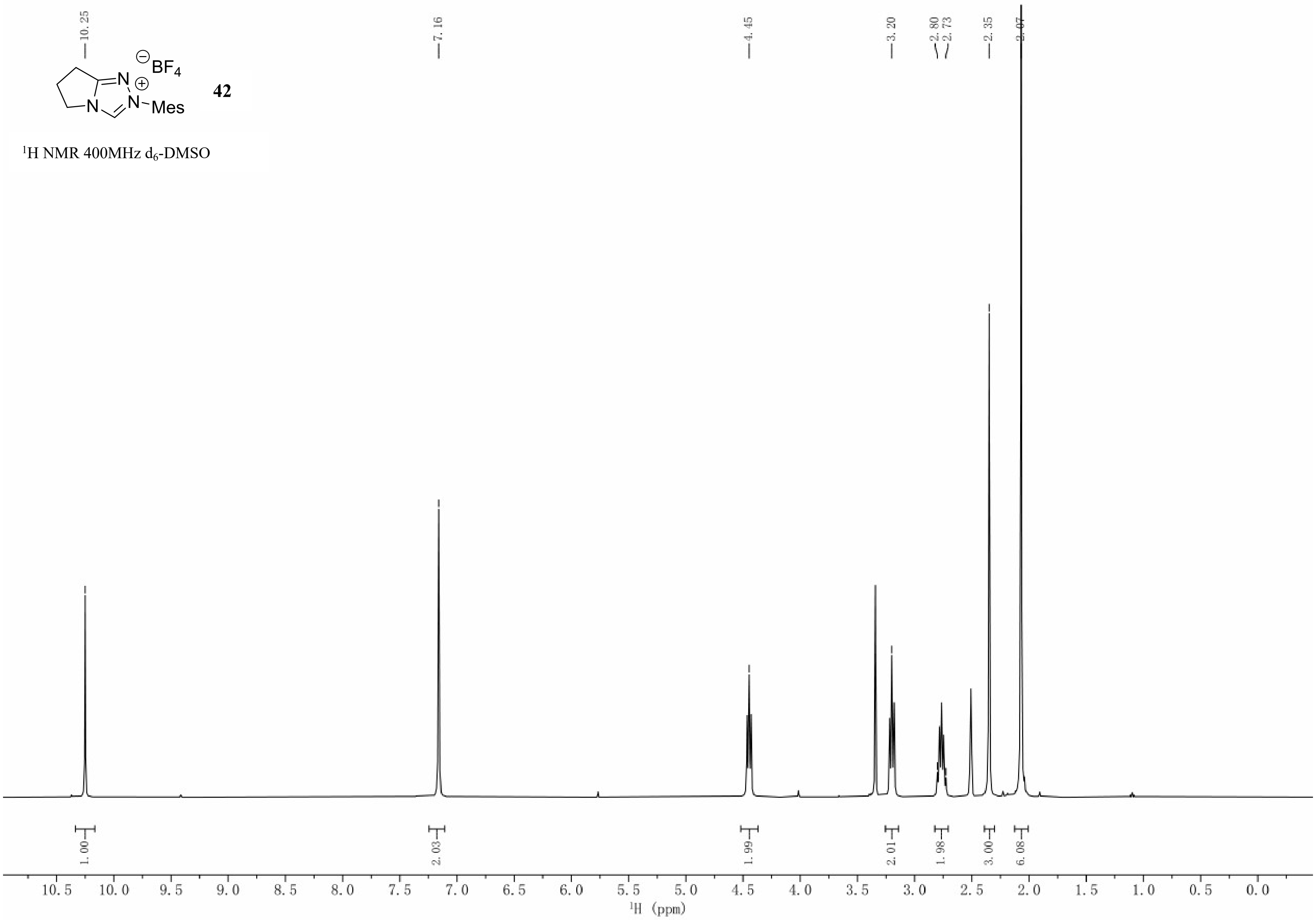

BF4
N
N
N
Mes
42
1H NMR 400MHz d6-DMSO
10.25
7.16
4.45
3.20
2.80
2.73
2.35
2.07
1.00
2.03
1.99
2.01
1.98
3.00
6.08
10.5 10.0 9.5 9.0 8.5 8.0 7.5 7.0 6.5 6.0 5.5 5.0 4.5 4.0 3.5 3.0 2.5 2.0 1.5 1.0 0.5 0.0
1H (ppm)

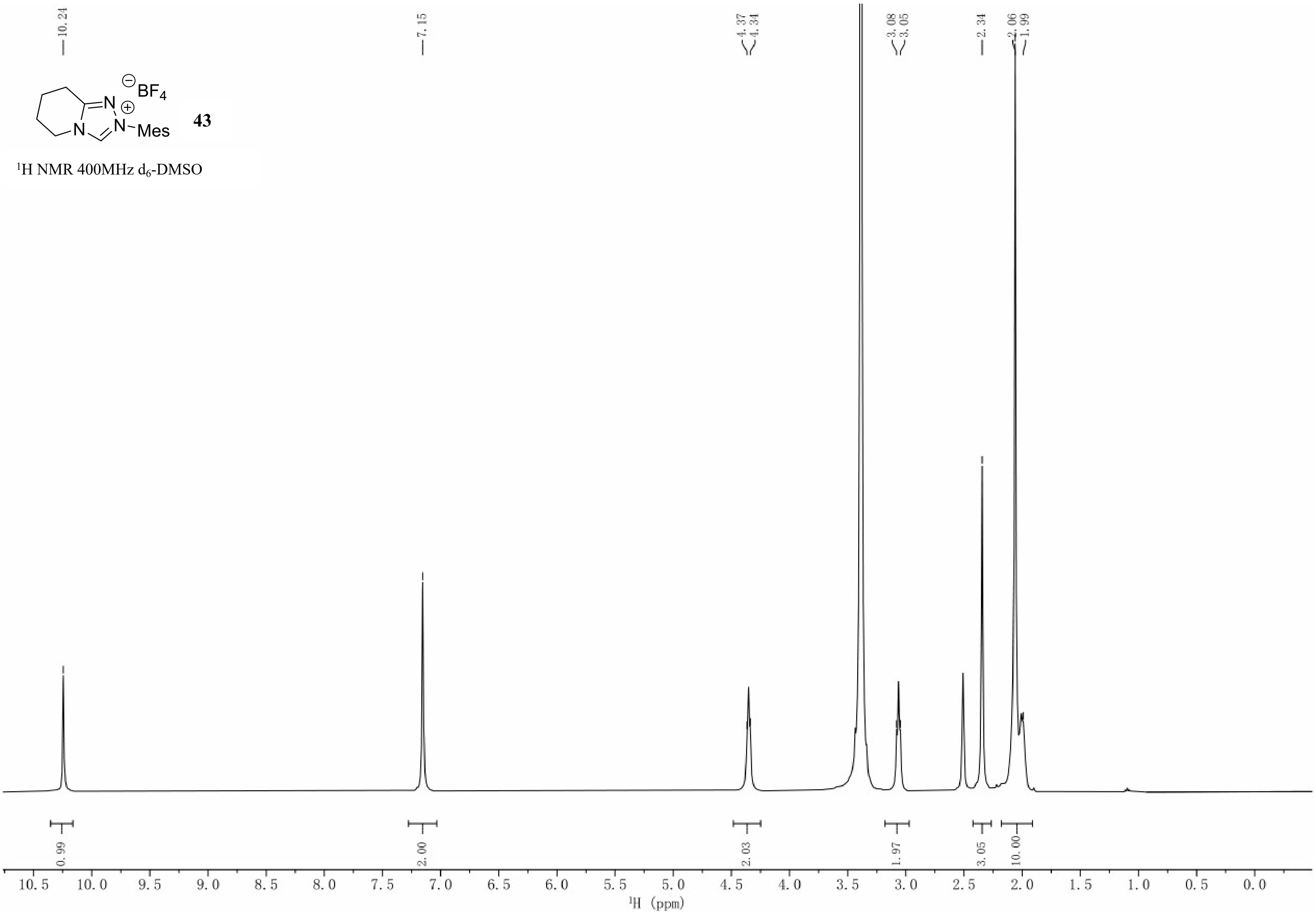

BF4
N
Mes
43
1H NMR 400MHz d6-DMSO
10.24
7.15
4.37
4.34
3.08
3.05
2.34
2.06
1.99
0.99
2.00
2.03
1.97
3.05
10.00
10.5
10.0
9.5
9.0
8.5
8.0
7.5
7.0
6.5
6.0
5.5
5.0
4.5
4.0
3.5
3.0
2.5
2.0
1.5
1.0
0.5
0.0
1H (ppm)

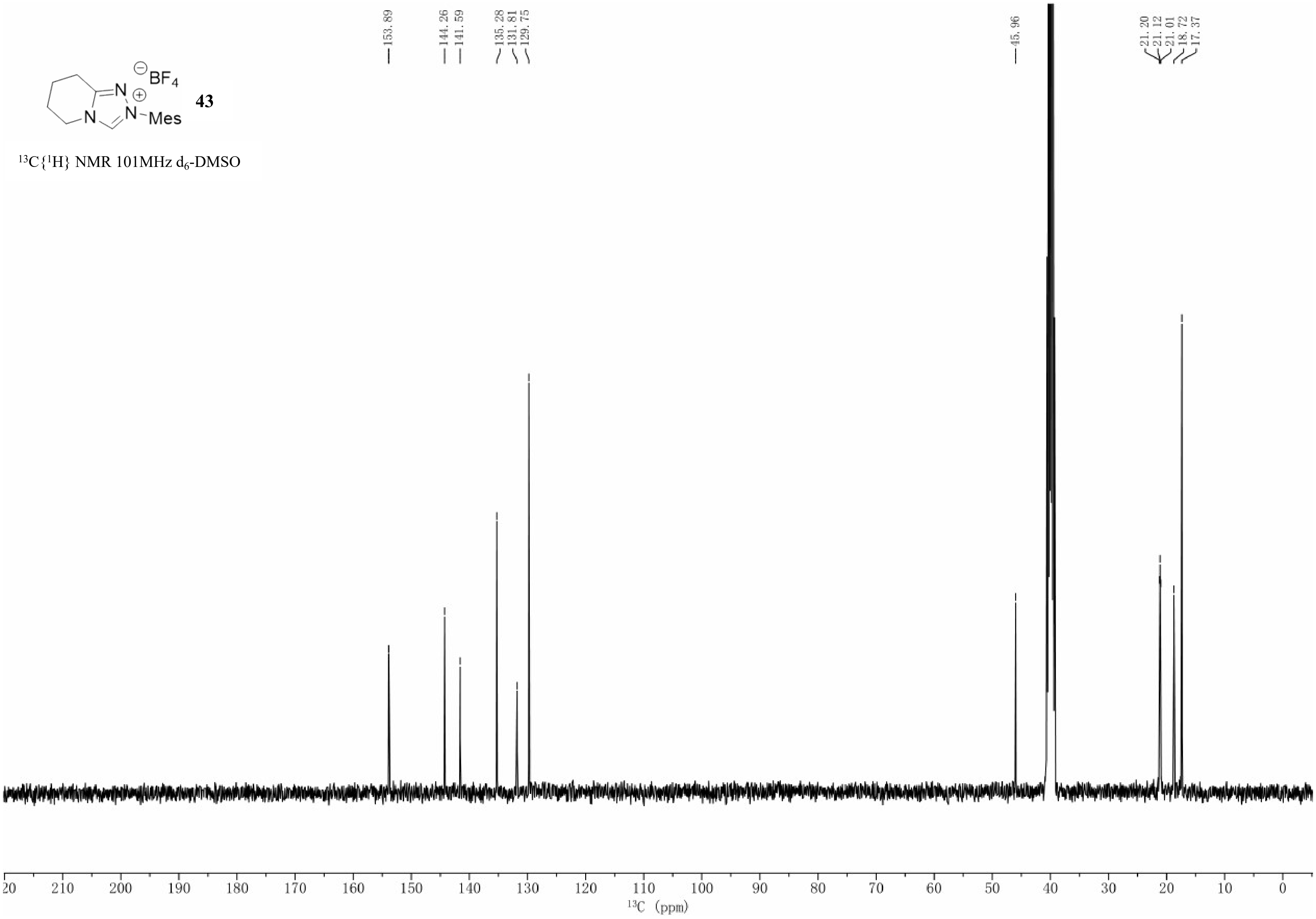

BF4
N
N
N
Mes
43
$^{13}C\{^{1}H\}$ NMR 101MHz d_6-DMSO
153.89
144.26
141.59
135.28
131.81
129.75
45.96
21.20
21.12
21.01
18.72
17.37
220
210
200
190
180
170
160
150
140
130
120
110
100
90
80
70
60
50
40
30
20
10
0
13C (ppm)

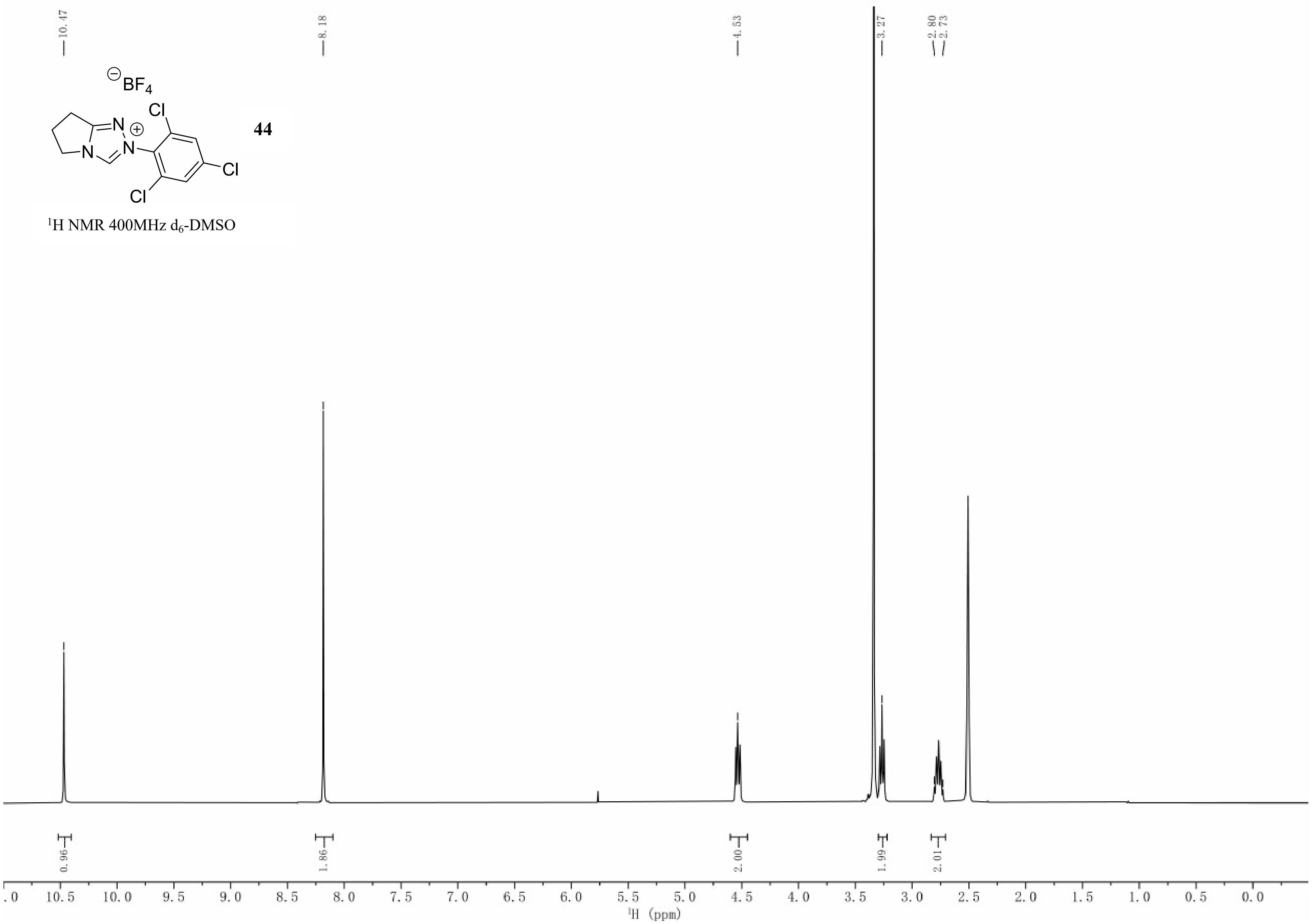
10.47
8.18
4.53
3.27
2.80
2.73
BF4
Cl
Cl
Cl
N
N
N
44
1H NMR 400MHz d6-DMSO
0.96
1.86
2.00
1.99
2.01
10.5 10.0 9.5 9.0 8.5 8.0 7.5 7.0 6.5 6.0 5.5 5.0 4.5 4.0 3.5 3.0 2.5 2.0 1.5 1.0 0.5 0.0
1H (ppm)

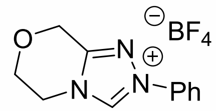

45

^{1}H NMR 400MHz d_6-DMSO

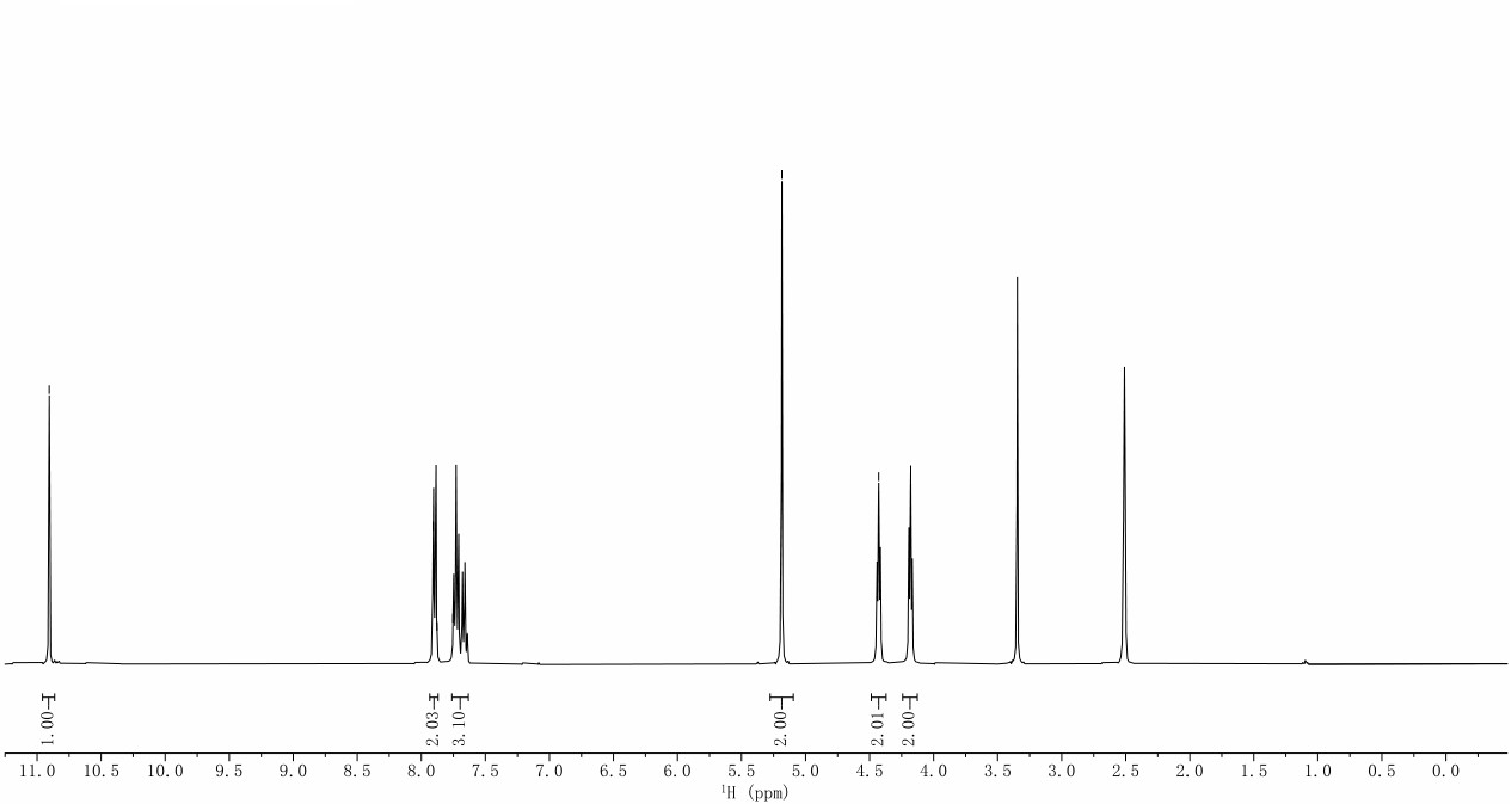

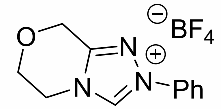

45

$^{13}C\{^1H\}$ NMR 101MHz d_6-DMSO

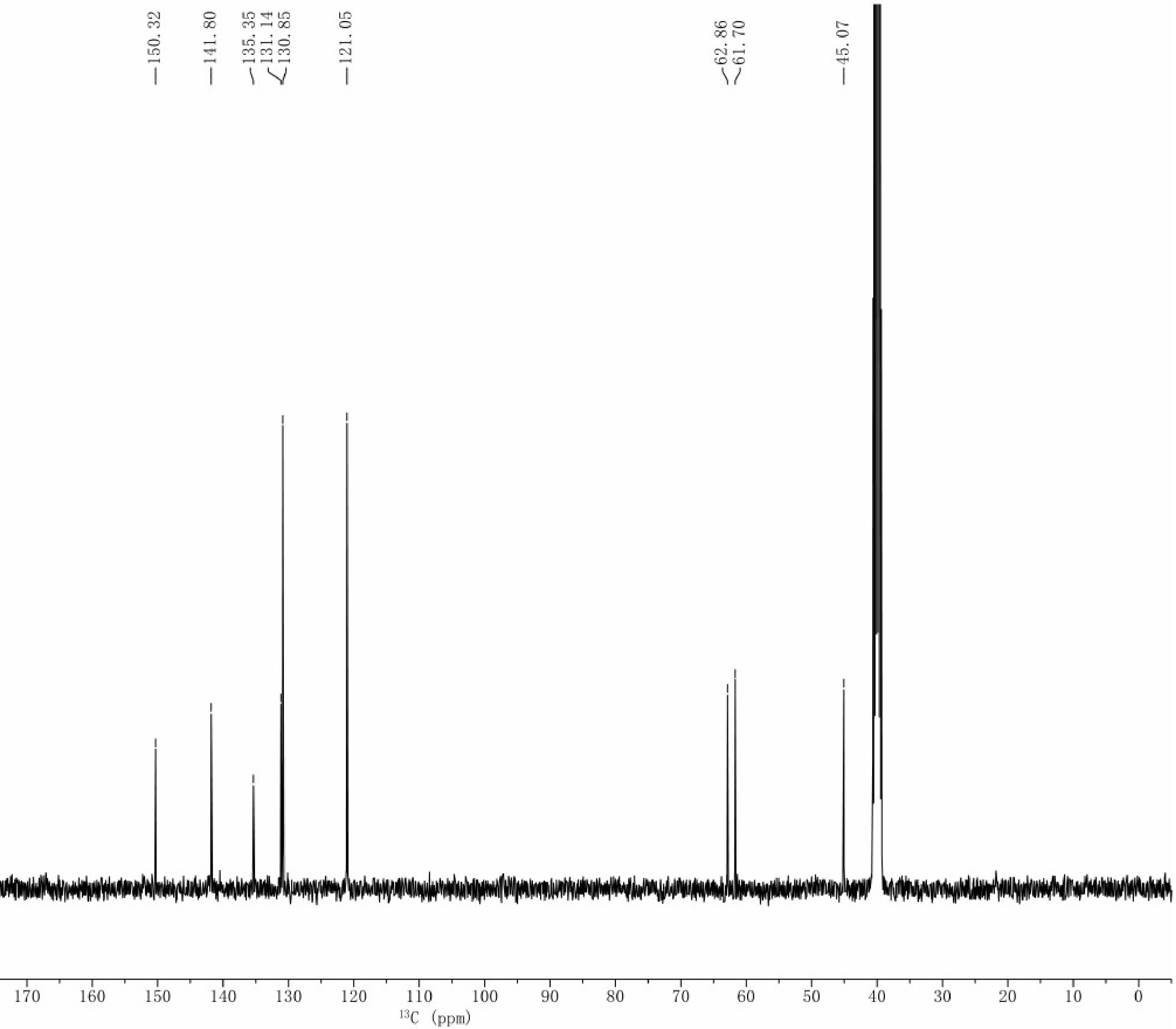

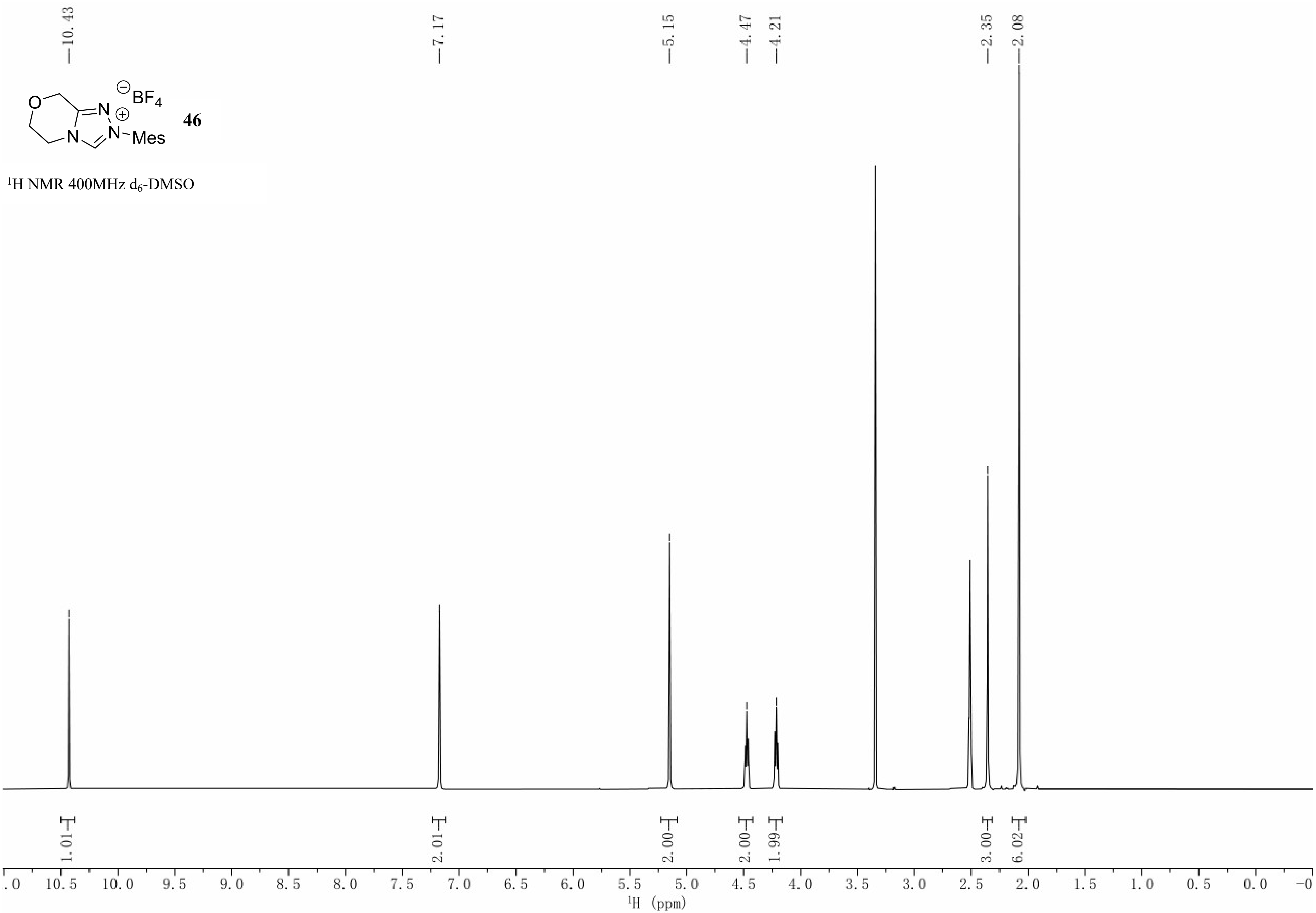

10.43
7.17
5.15
4.47
4.21
2.35
2.08
BF4
N
N
N-Mes
O
46
1H NMR 400MHz d6-DMSO
1.01
2.01
2.00
2.00
1.99
3.00
6.02
1H (ppm)

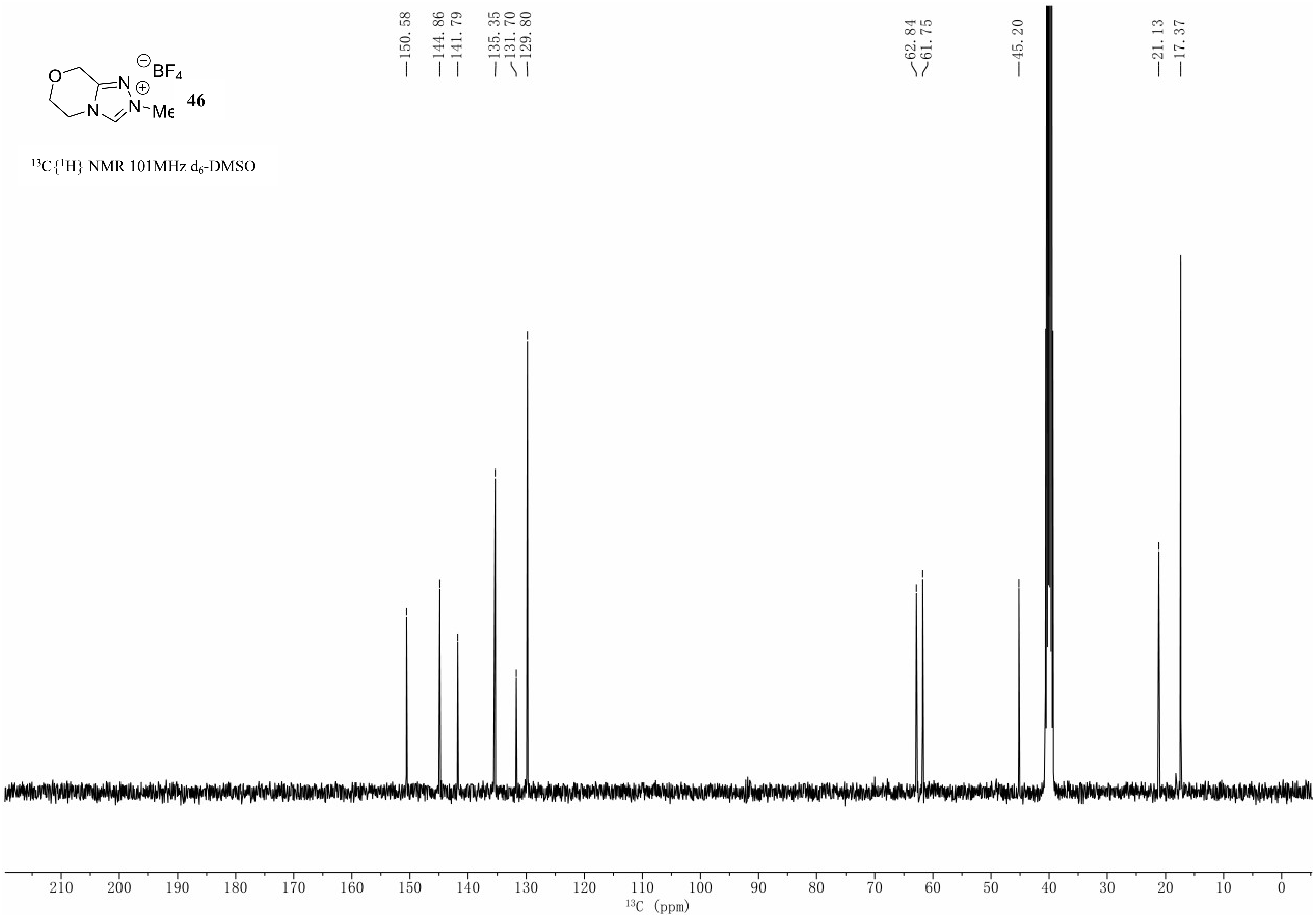

46

$^{13}C\{^1H\}$ NMR 101MHz d_6-DMSO

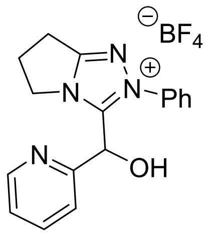

11

^{1}H NMR 400MHz d_4-CD_3OD

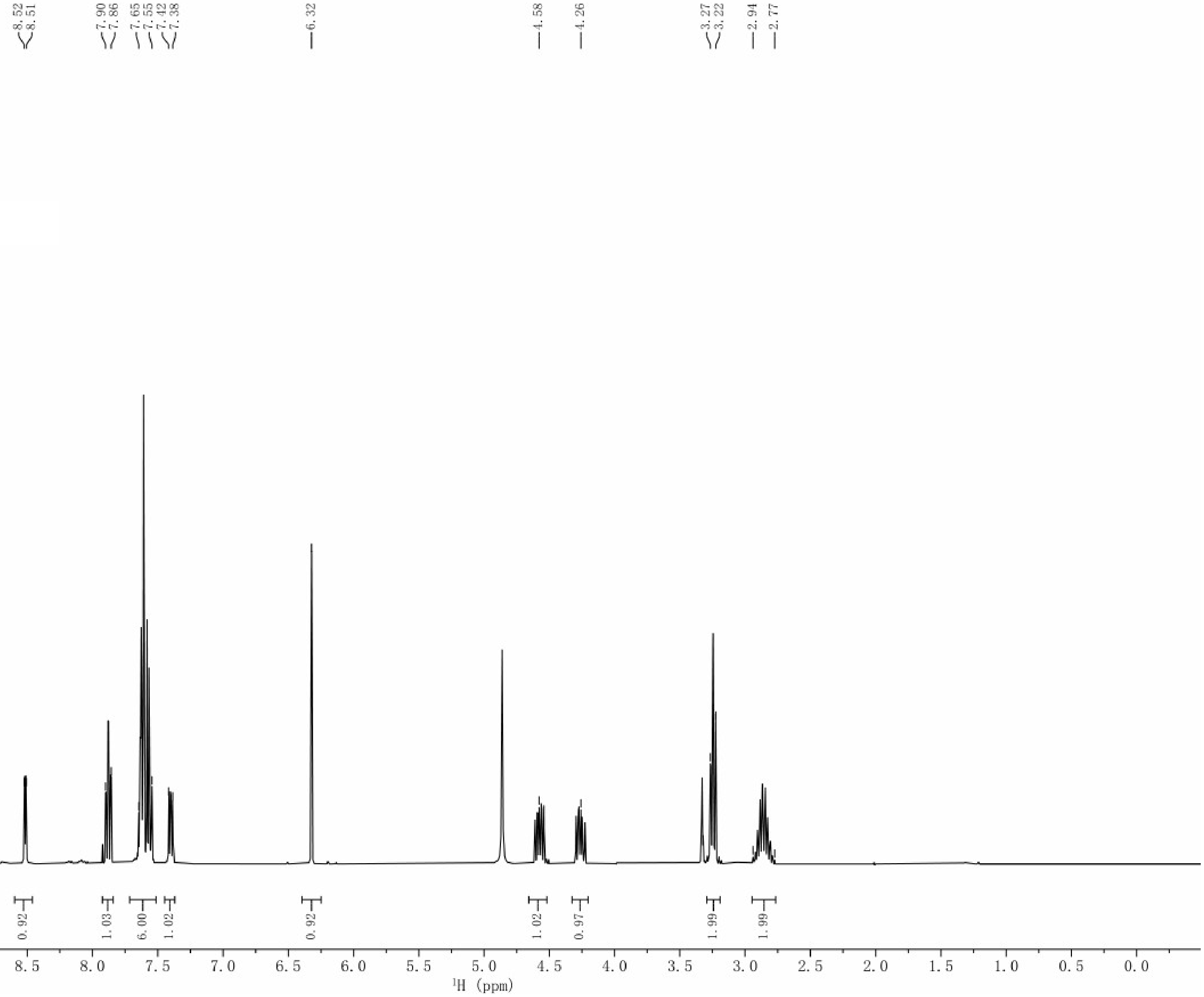

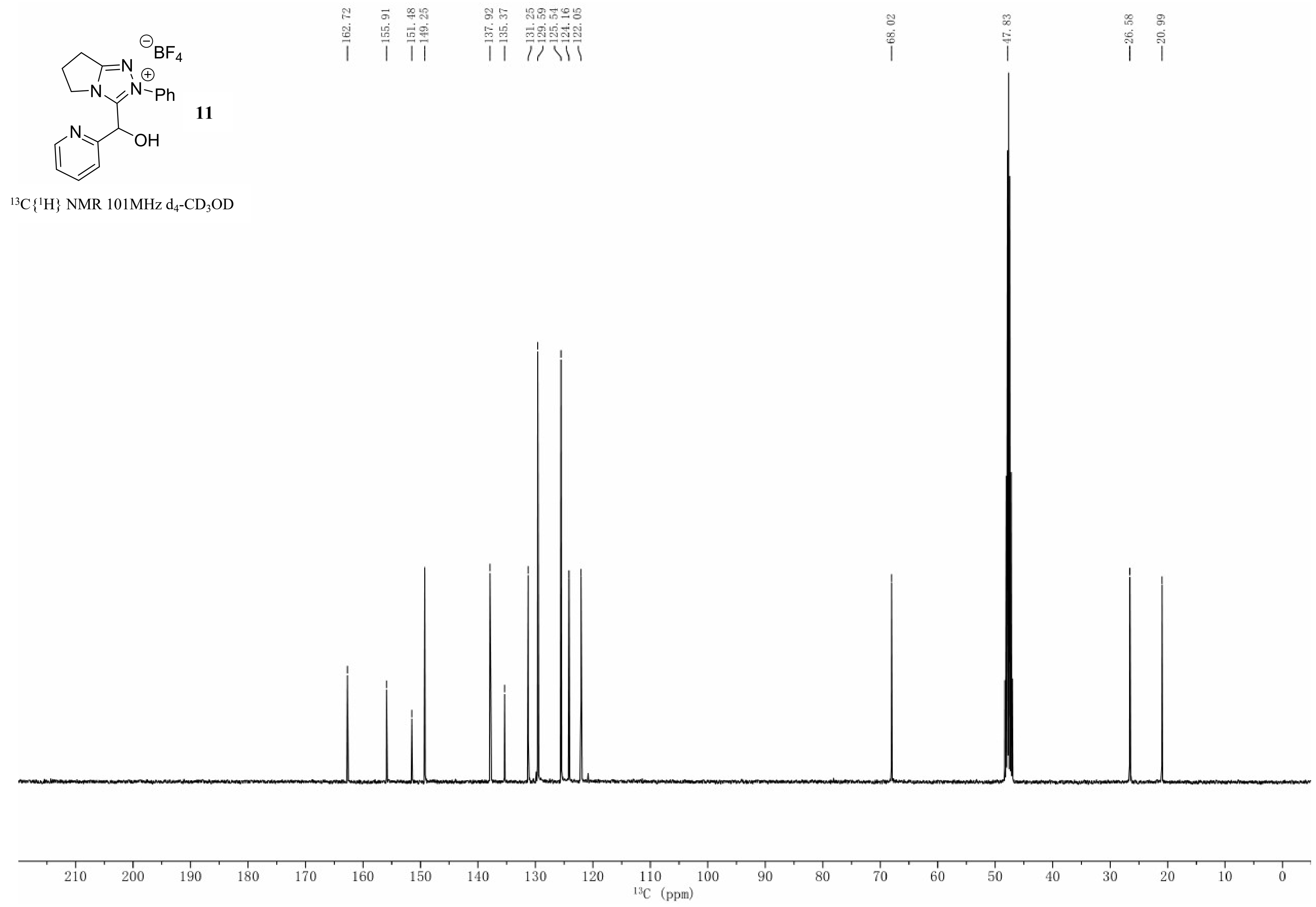

BF4
N
N
Ph
N
N
OH
11
13C{1H} NMR 101MHz d4-CD3OD
162.72
155.91
151.48
149.25
137.92
135.37
131.25
129.59
125.54
124.16
122.05
68.02
47.83
26.58
20.99
210
200
190
180
170
160
150
140
130
120
110
100
90
80
70
60
50
40
30
20
10
0
13C (ppm)

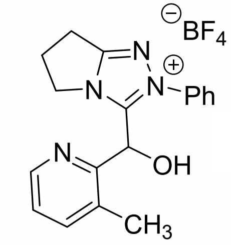

12

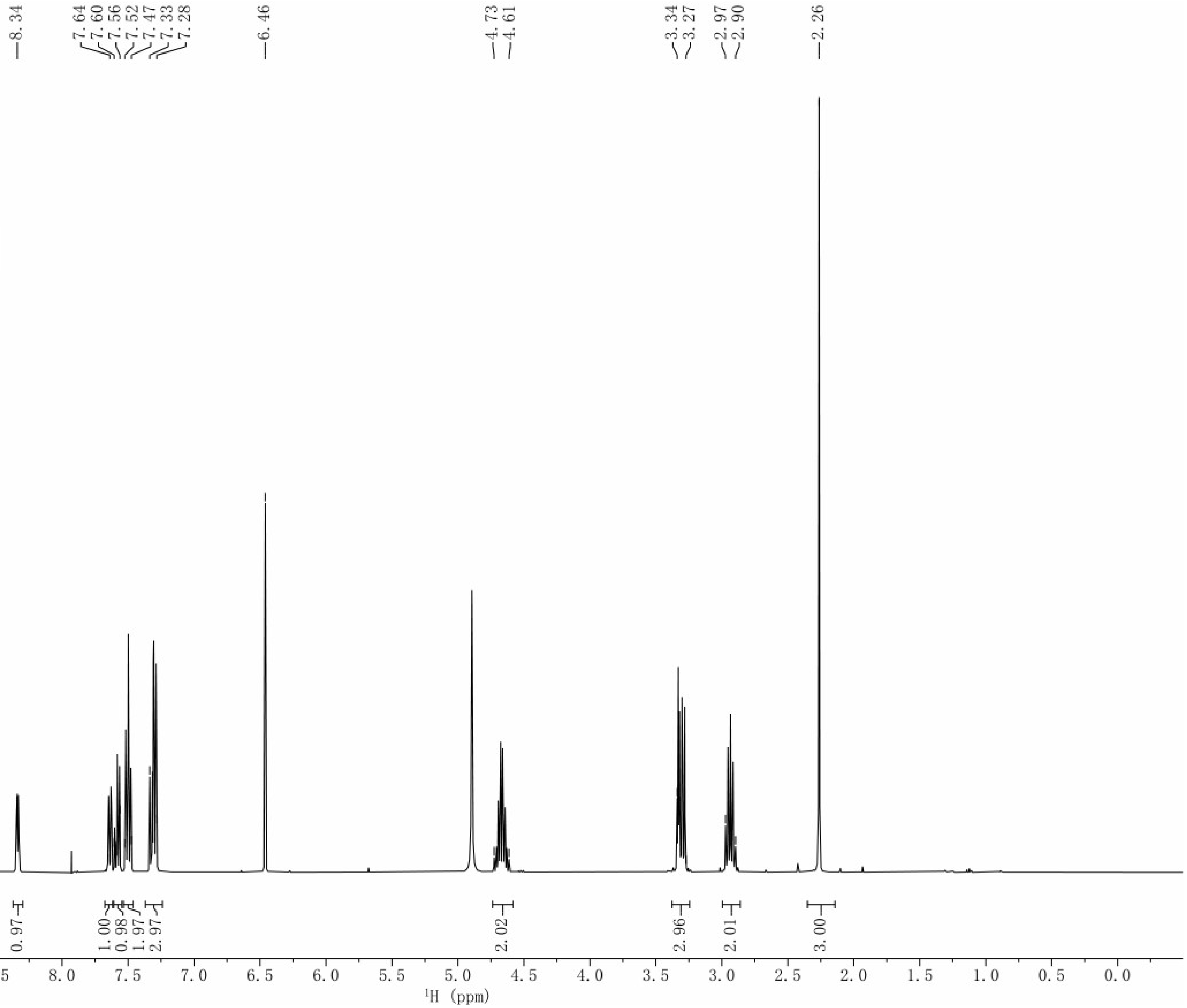

^{1}H NMR 400MHz d_4-CD_3OD

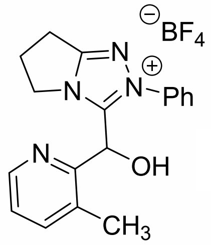

12

$^{13}C\{^1H\}$ NMR 101MHz d_4-CD_3OD

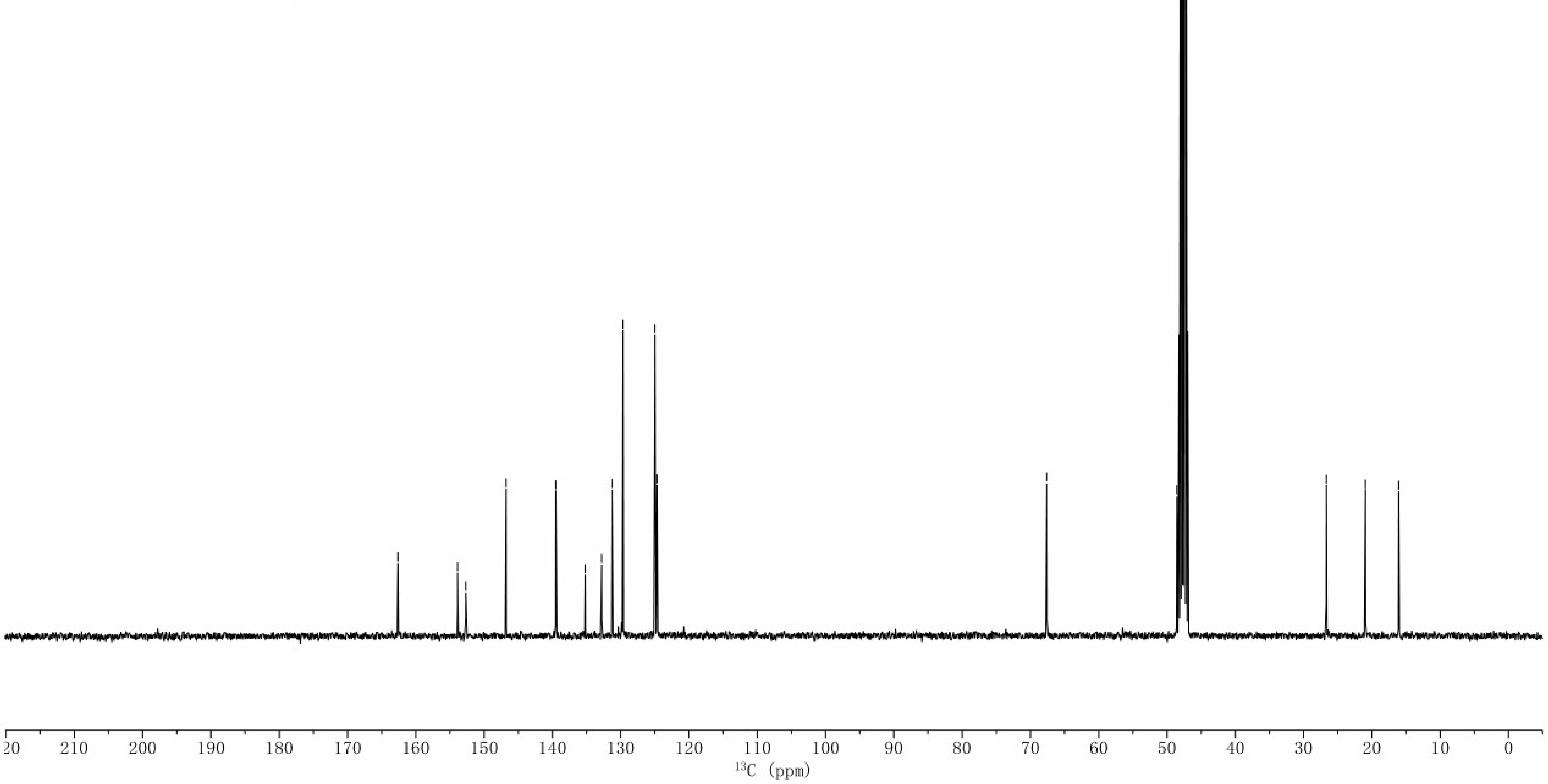

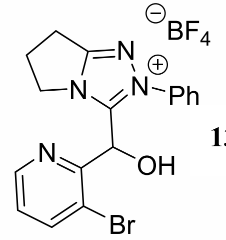

13

^{1}H NMR 400MHz d_4-CD_3OD

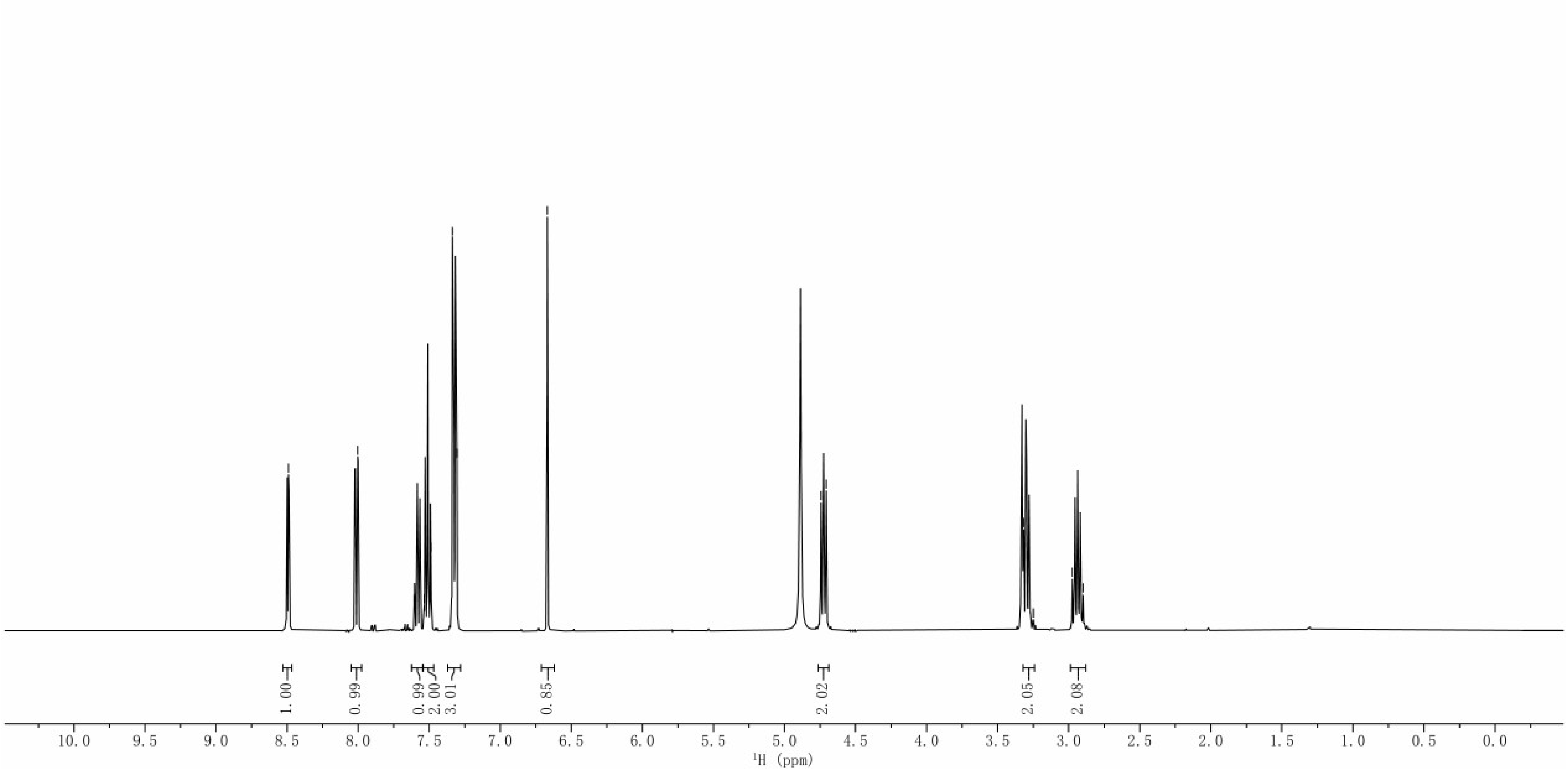

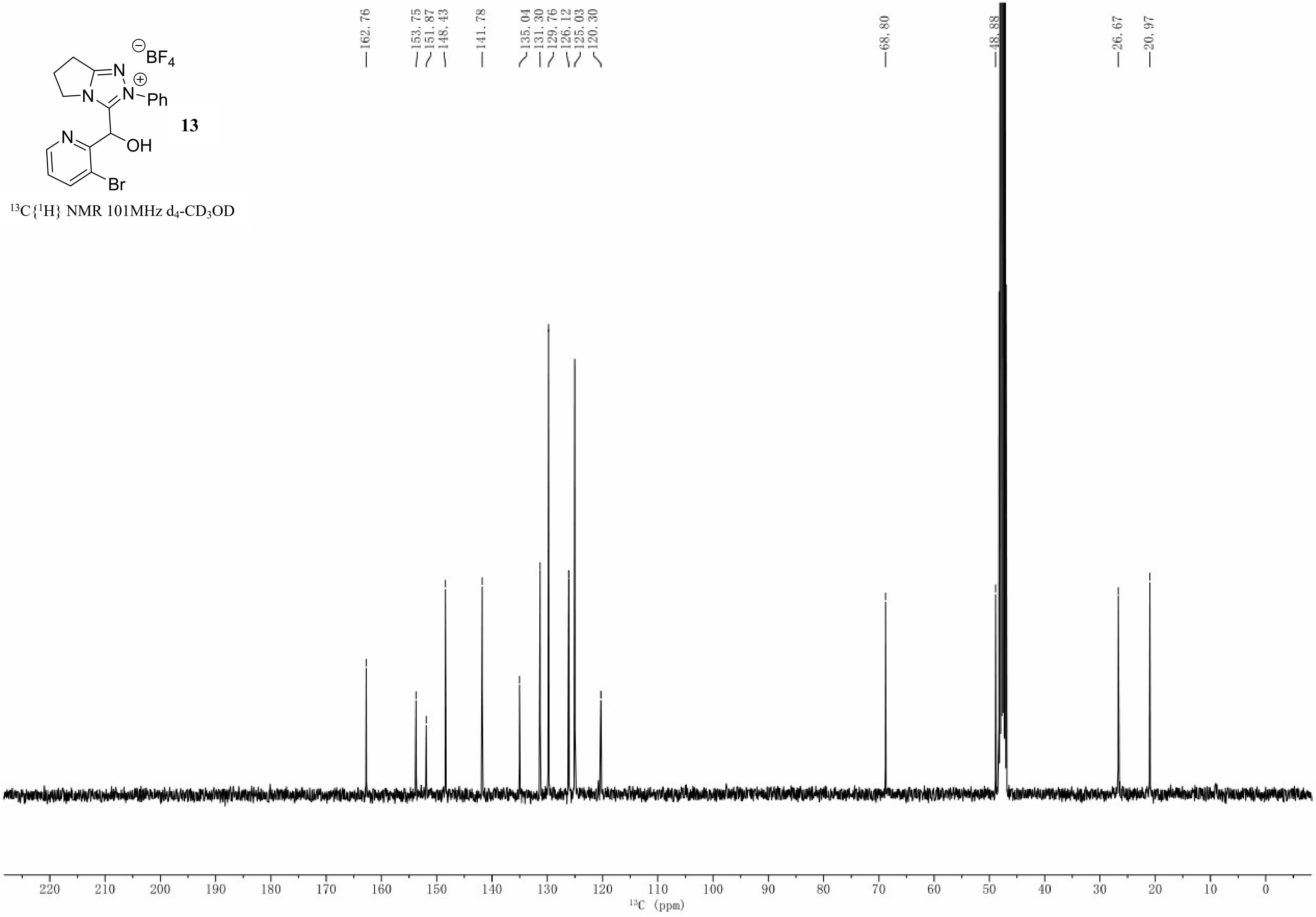
162.76
153.75
151.87
148.43
141.78
135.04
131.30
129.76
126.12
125.03
120.30
68.80
48.88
26.67
20.97
BF4
N
N
N
Ph
N
OH
Br
13
13C{1H} NMR 101MHz d4-CD3OD
220 210 200 190 180 170 160 150 140 130 120 110 100 90 80 70 60 50 40 30 20 10 0
13C (ppm)

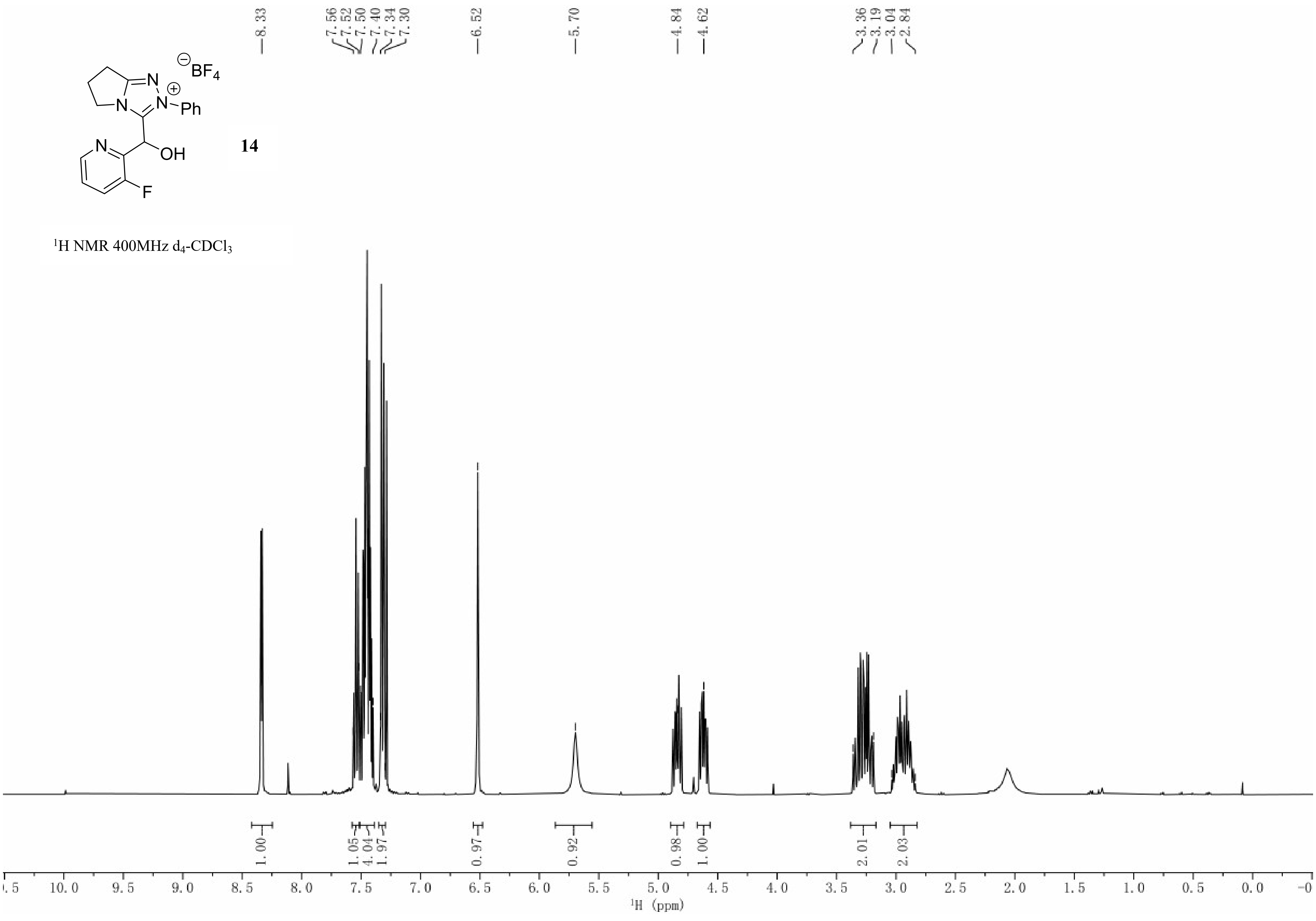

14
^{1}H NMR 400MHz d$_4$-CDCl$_3$
8.33
7.56
7.52
7.50
7.40
7.34
7.30
6.52
5.70
4.84
4.62
3.36
3.19
3.04
2.84
1.00
1.05
4.04
1.97
0.97
0.92
0.98
1.00
2.01
2.03
^{1}H (ppm)

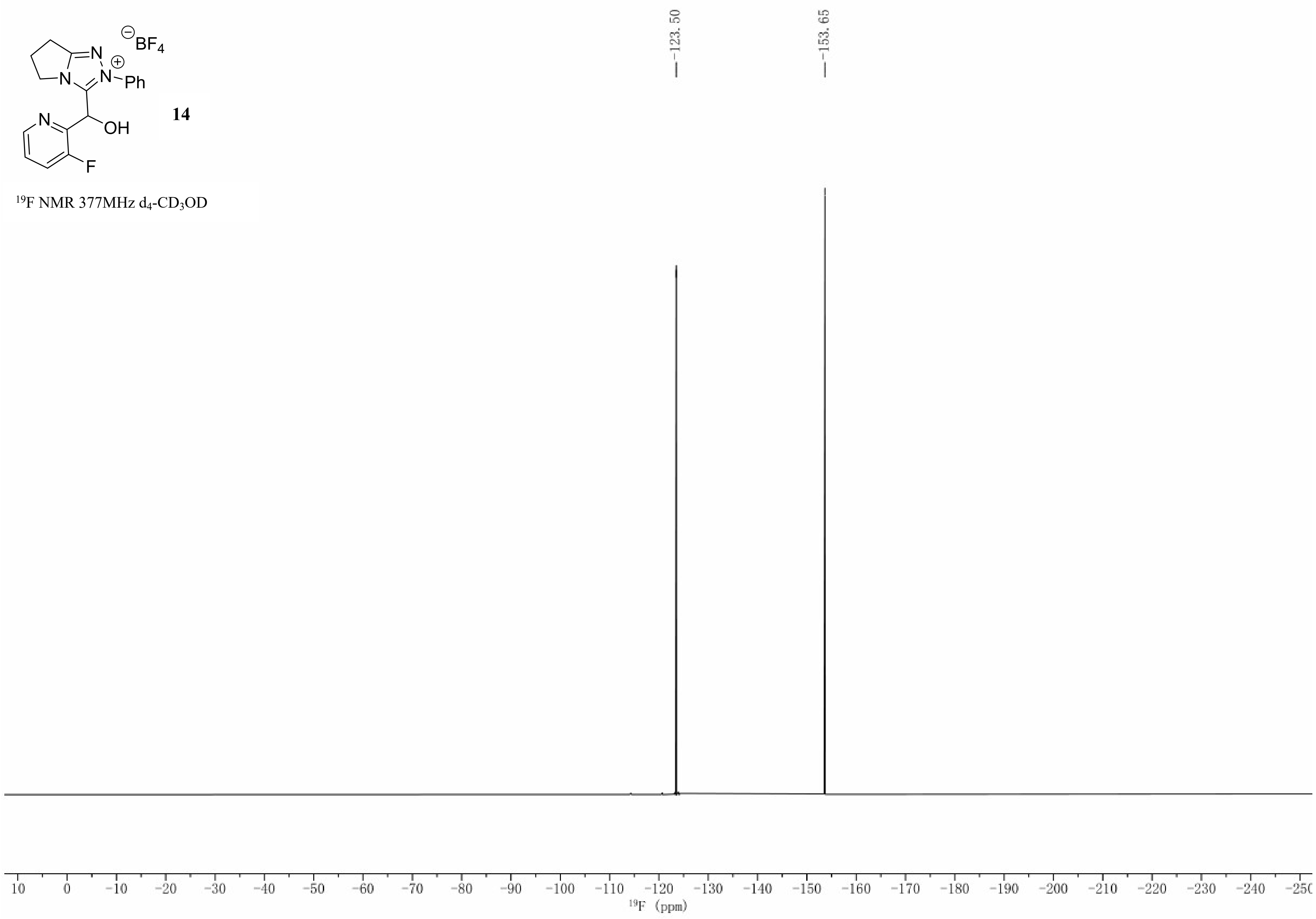

BF4
N
N
N
Ph
OH
F
14
19F NMR 377MHz d4-CD3OD
-123.50
-153.65
10 0 -10 -20 -30 -40 -50 -60 -70 -80 -90 -100 -110 -120 -130 -140 -150 -160 -170 -180 -190 -200 -210 -220 -230 -240 -250
19F (ppm)

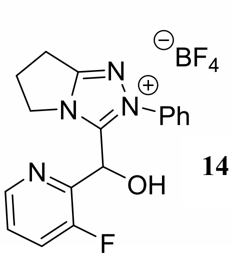

14

$^{13}C\{^1H\}$ NMR 101MHz d_4-$CDCl_3$

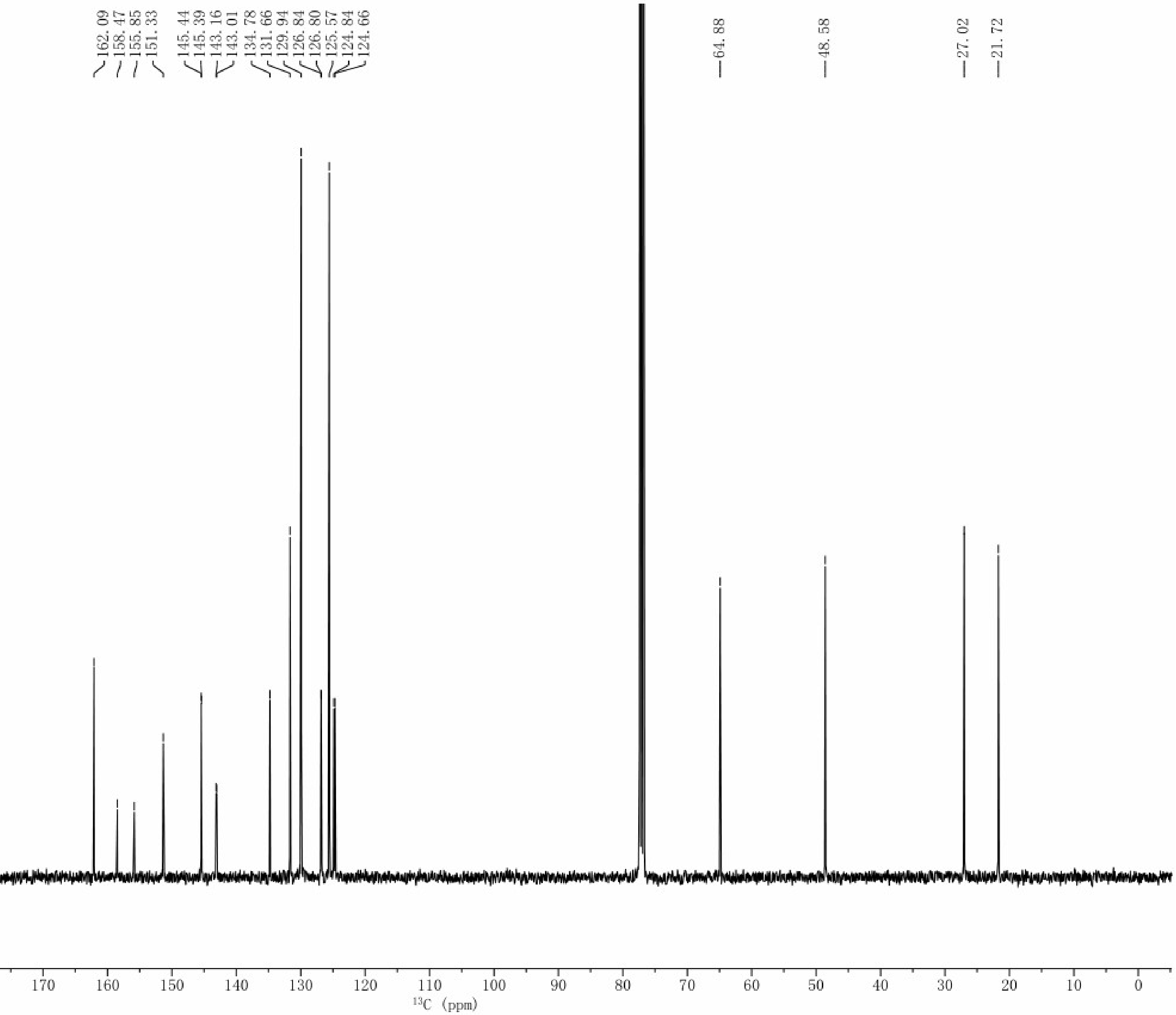

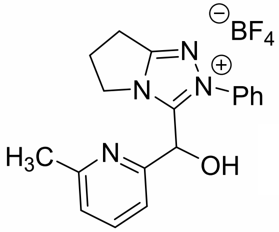

15

^1H NMR 400MHz d_4-CD_3OD

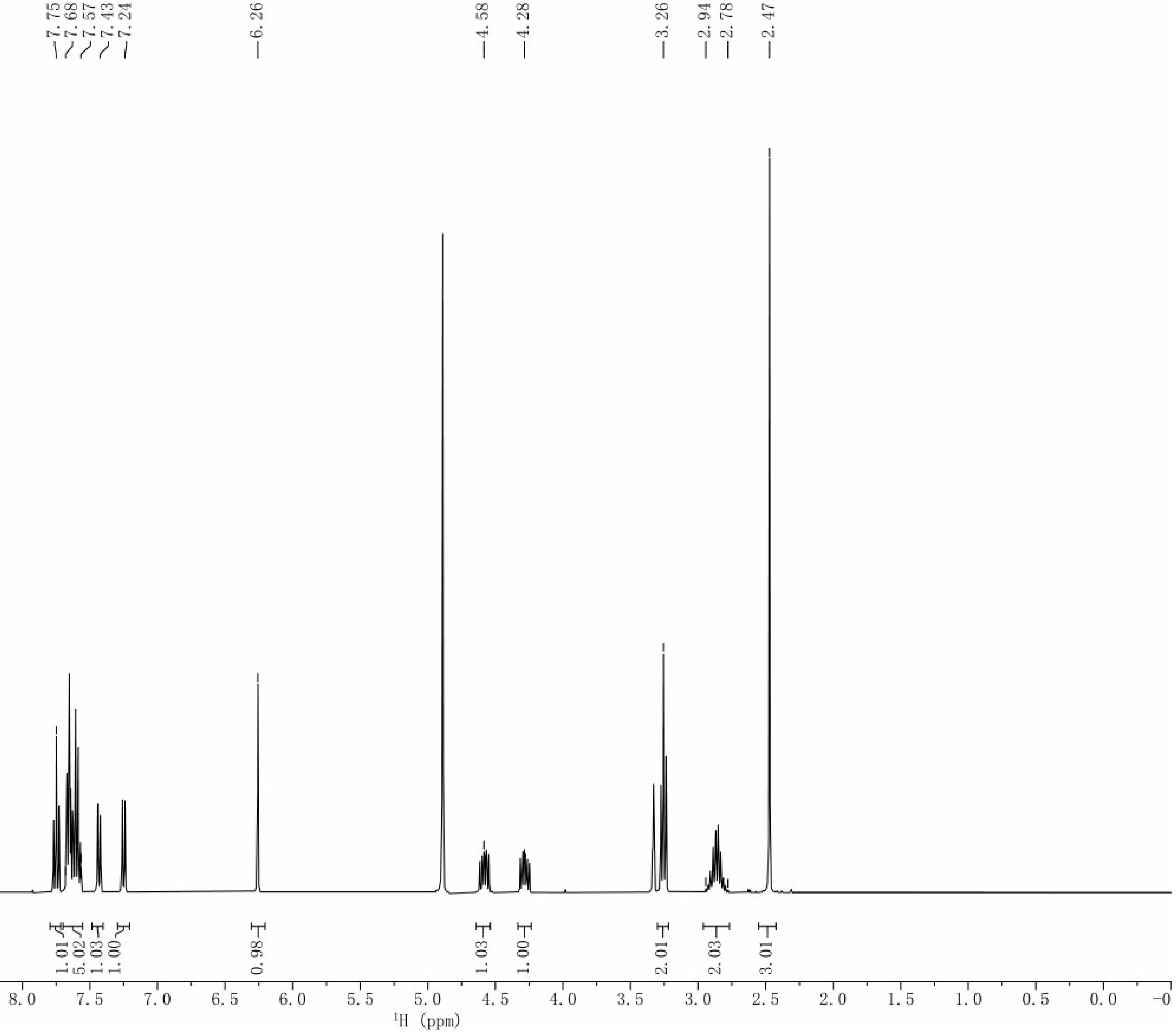

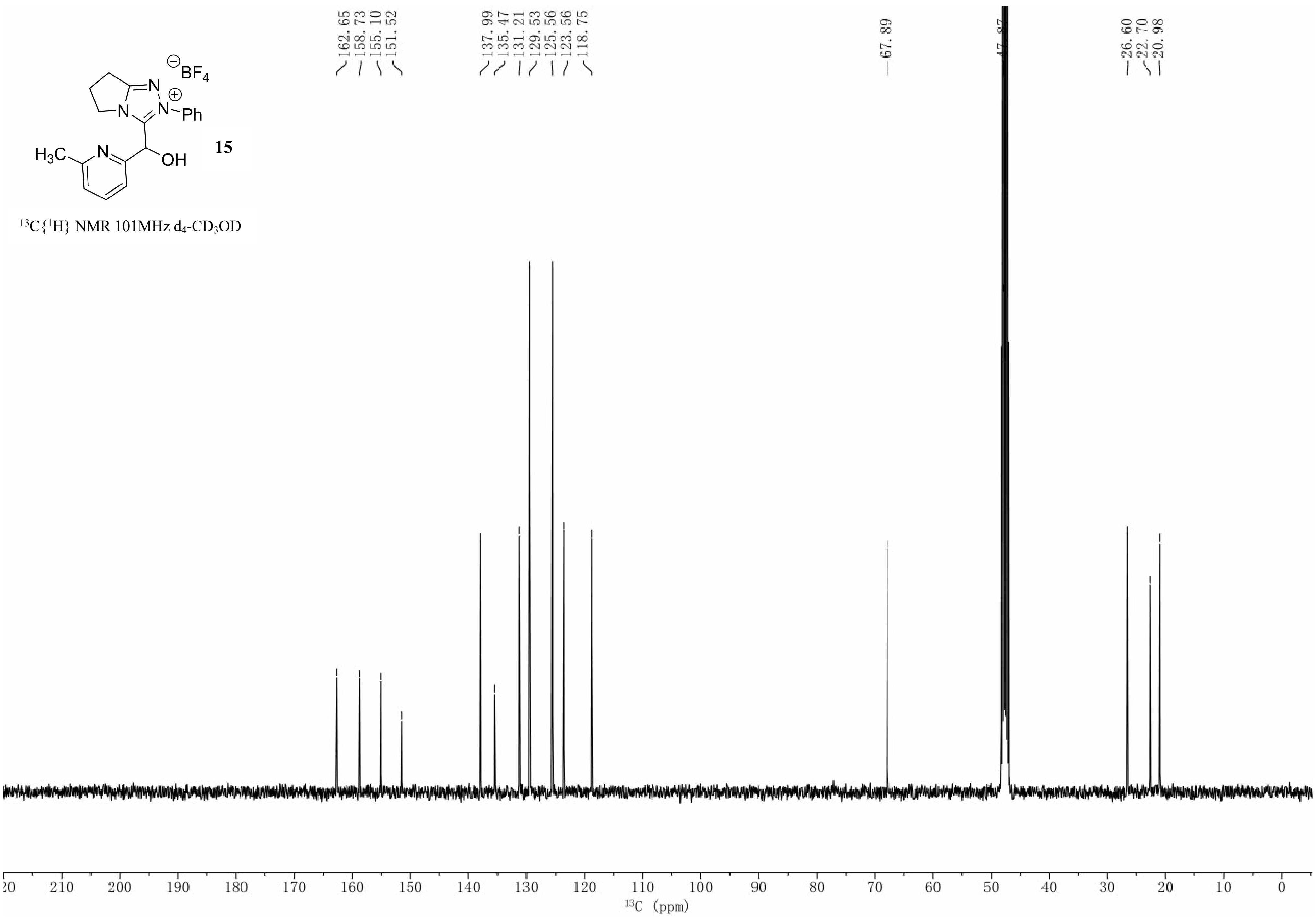

162.65
158.73
155.10
151.52
137.99
135.47
131.21
129.53
125.56
123.56
118.75
67.89
26.60
22.70
20.98
BF4
N
N
N
Ph
H3C
N
OH
15
$^{13}C\{^{1}H\}$ NMR 101MHz d_4-CD_3OD
210
200
190
180
170
160
150
140
130
120
110
100
90
80
70
60
50
40
30
20
10
0
^{13}C (ppm)

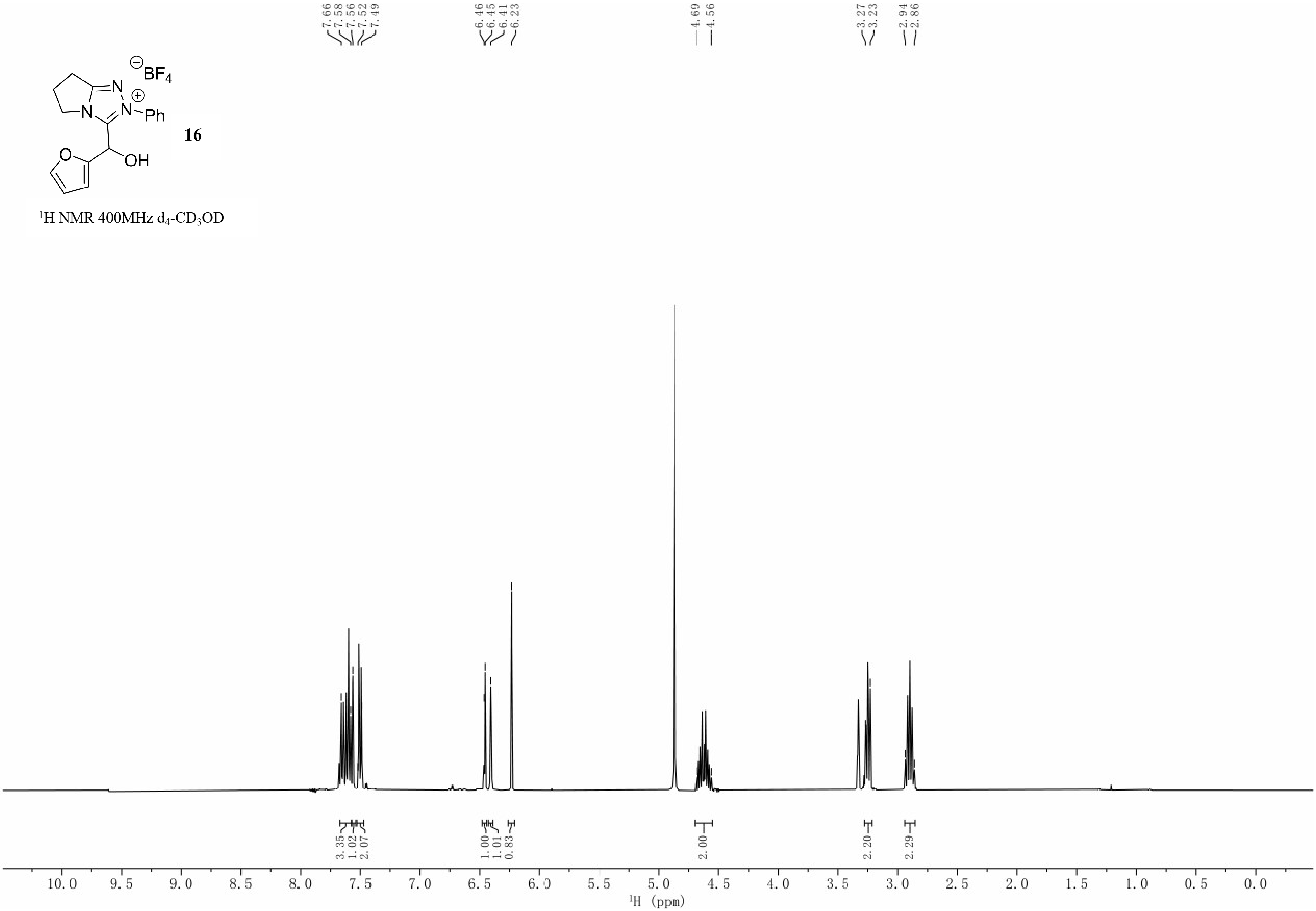
7.66
7.58
7.56
7.52
7.49
6.46
6.45
6.41
6.23
4.69
4.56
3.27
3.23
2.94
2.86
BF4
N
N
Ph
OH
O
16
1H NMR 400MHz d4-CD3OD
3.35
1.02
2.07
1.00
1.01
0.83
2.00
2.20
2.29
10.0
9.5
9.0
8.5
8.0
7.5
7.0
6.5
6.0
5.5
5.0
4.5
4.0
3.5
3.0
2.5
2.0
1.5
1.0
0.5
0.0
1H (ppm)

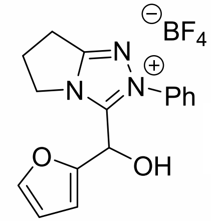

16

$^{13}C\{^{1}H\}$ NMR 101MHz d_4-CD_3OD

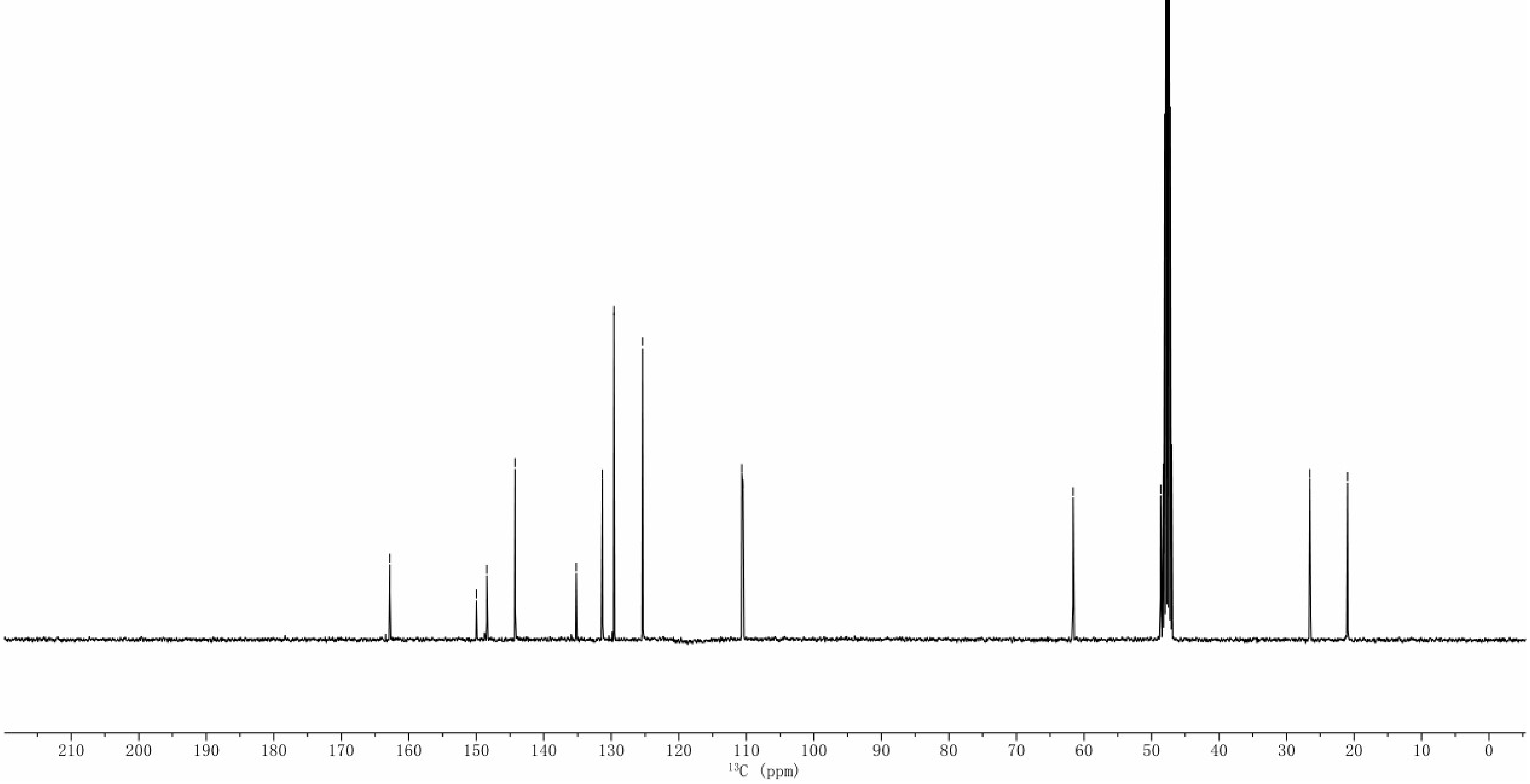

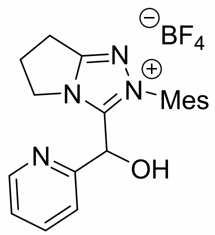

17

^{1}H NMR 400MHz d_4-CD_3OD

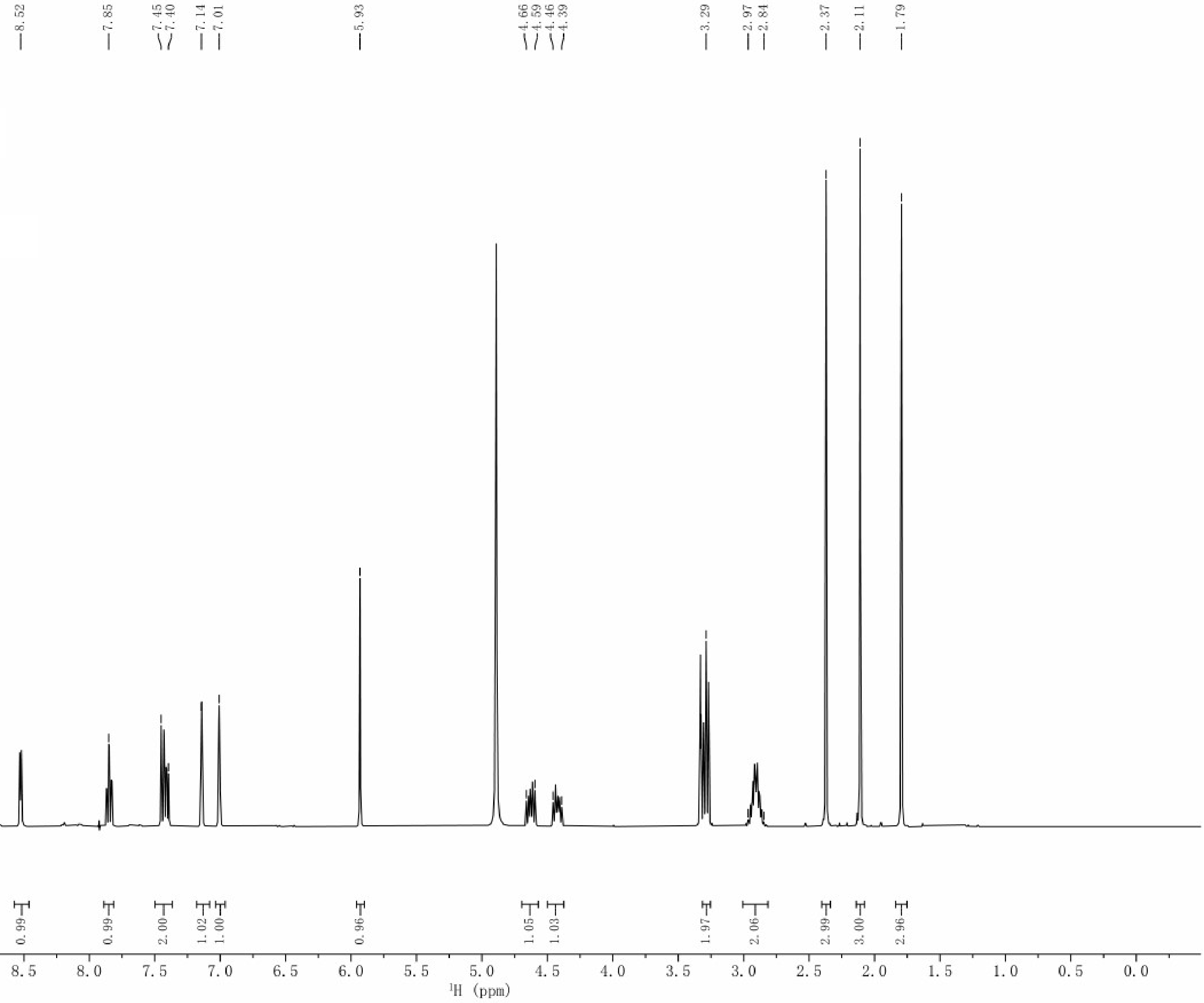

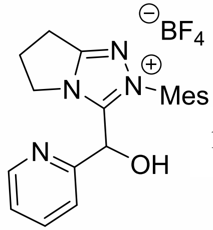

17

$^{13}C\{^{1}H\}$ NMR 101MHz d_4-CD_3OD

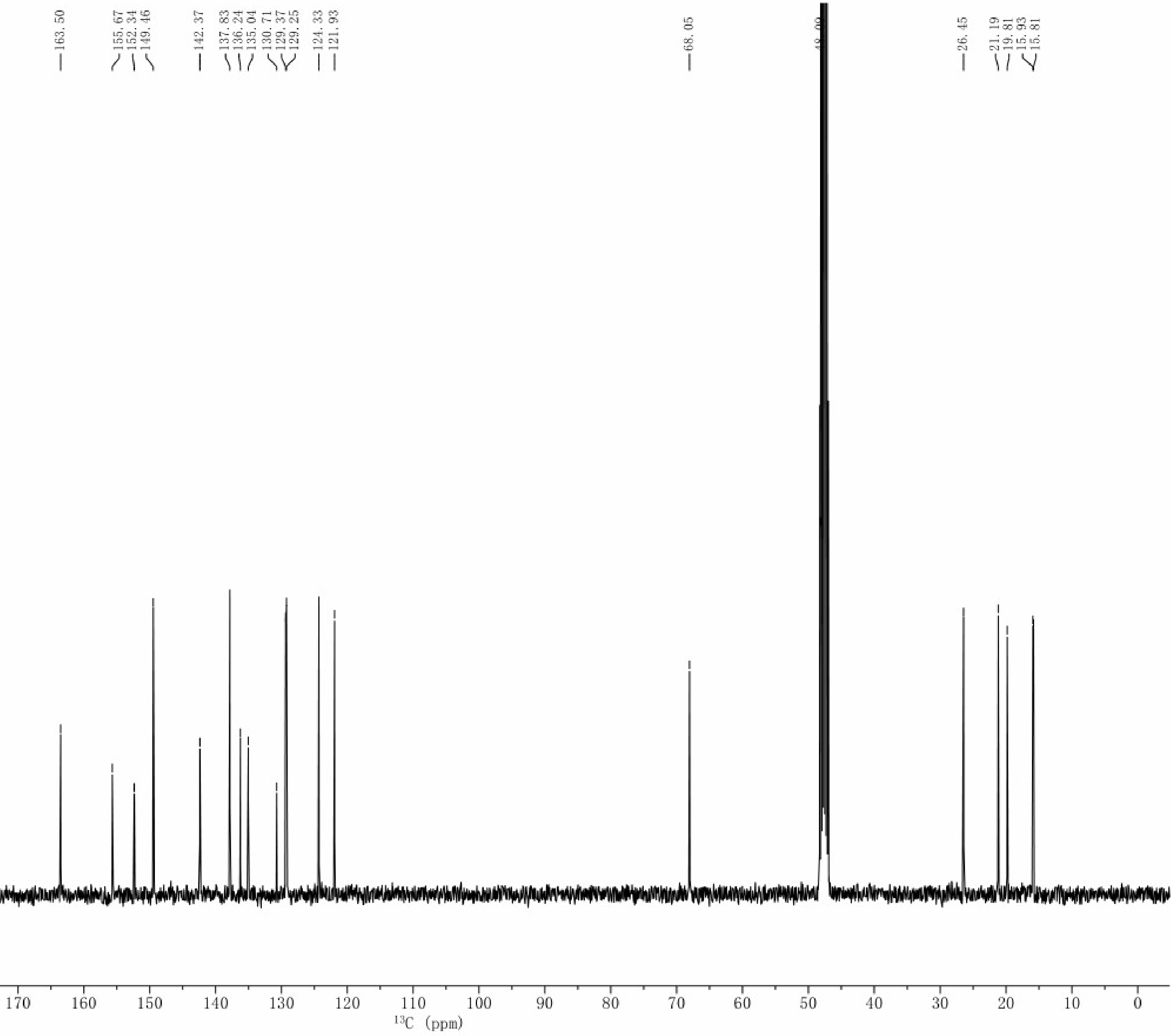

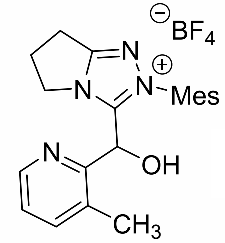

18

^{1}H NMR 400MHz d_4-CD_3OD

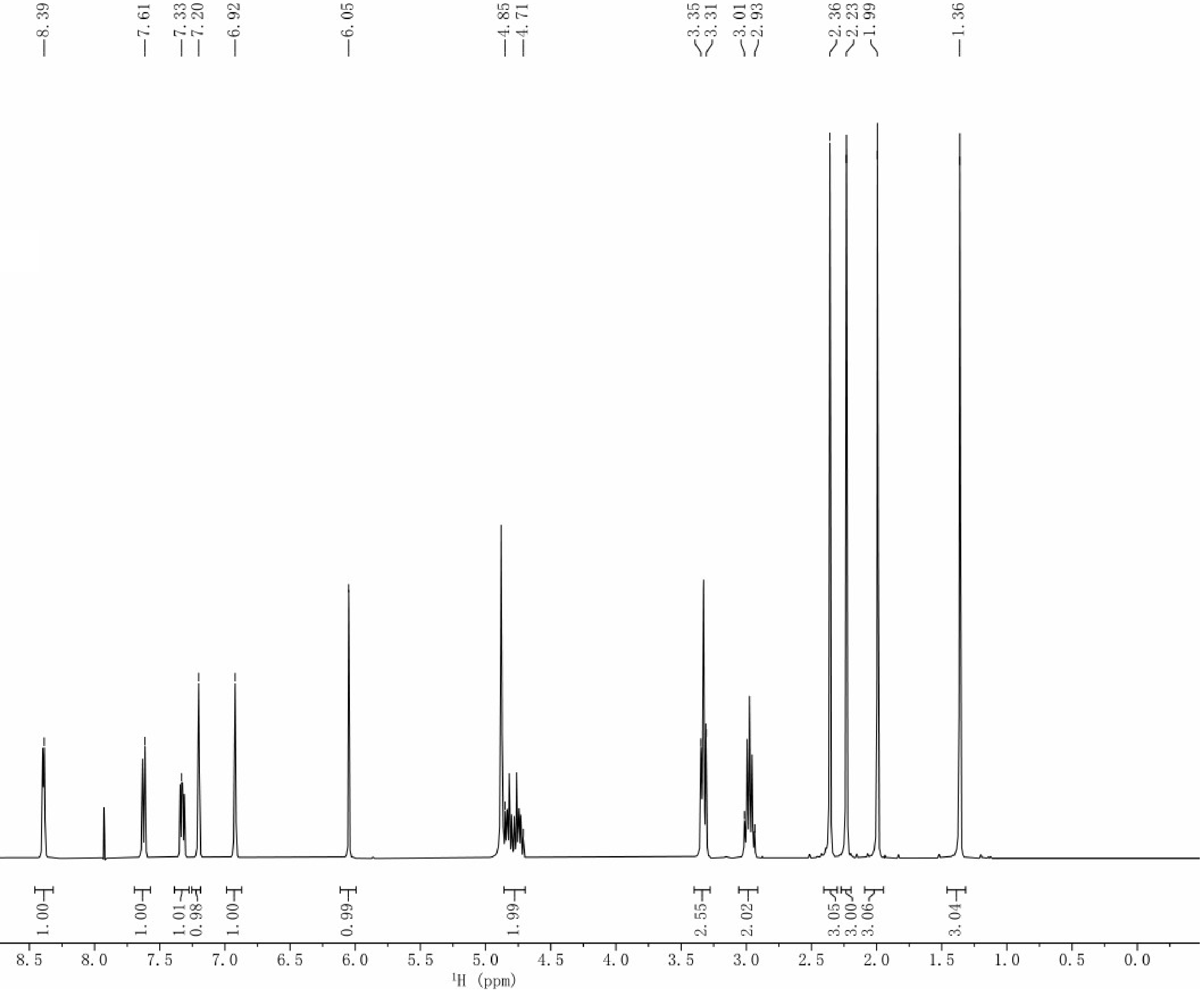

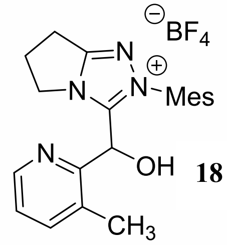

18

$^{13}C\{^{1}H\}$ NMR 101MHz d_4-CD_3OD

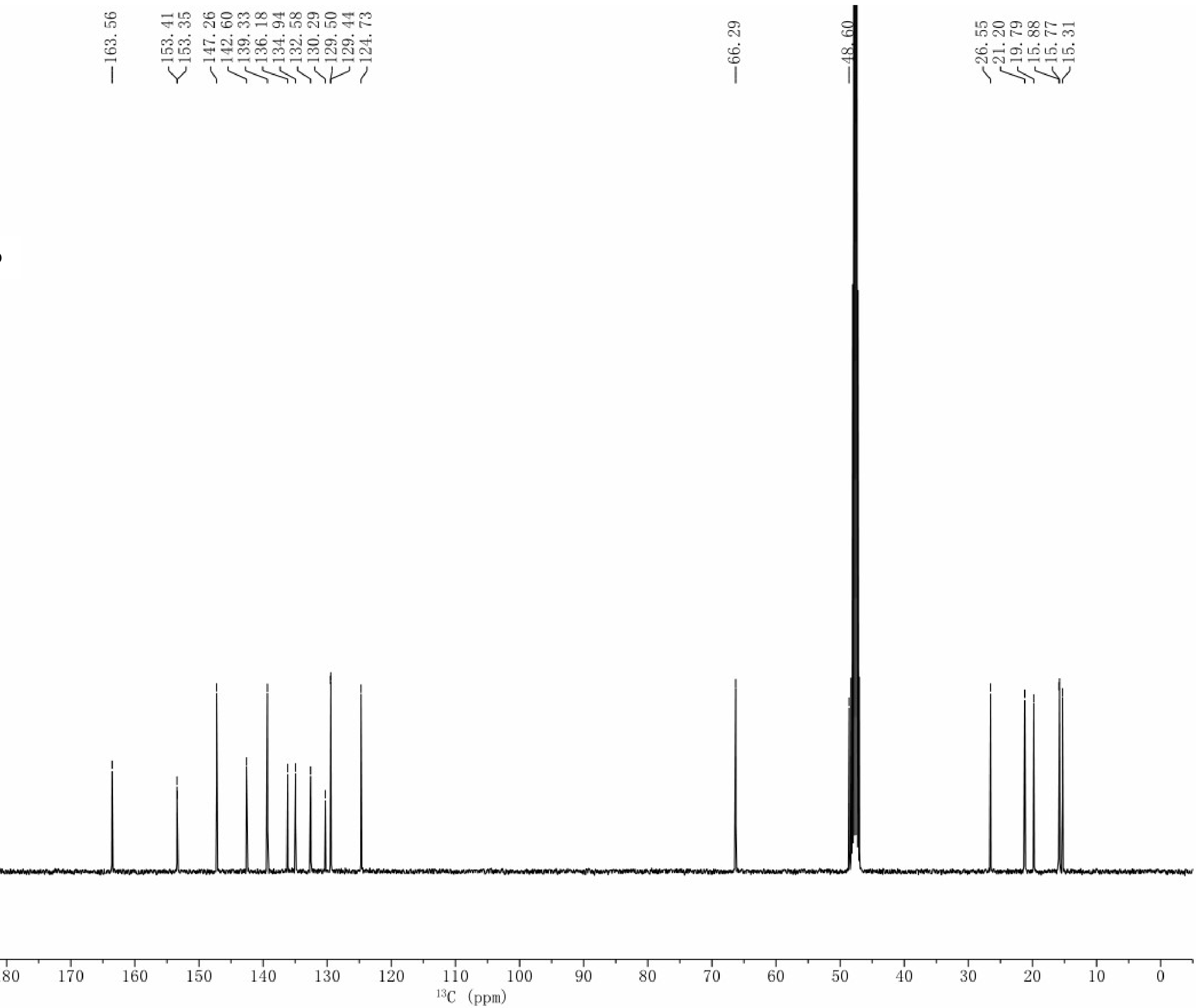

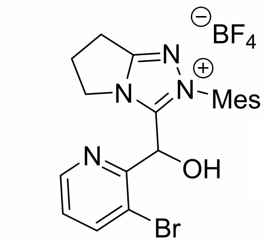

19

^{1}H NMR 400MHz d_4-CD_3OD

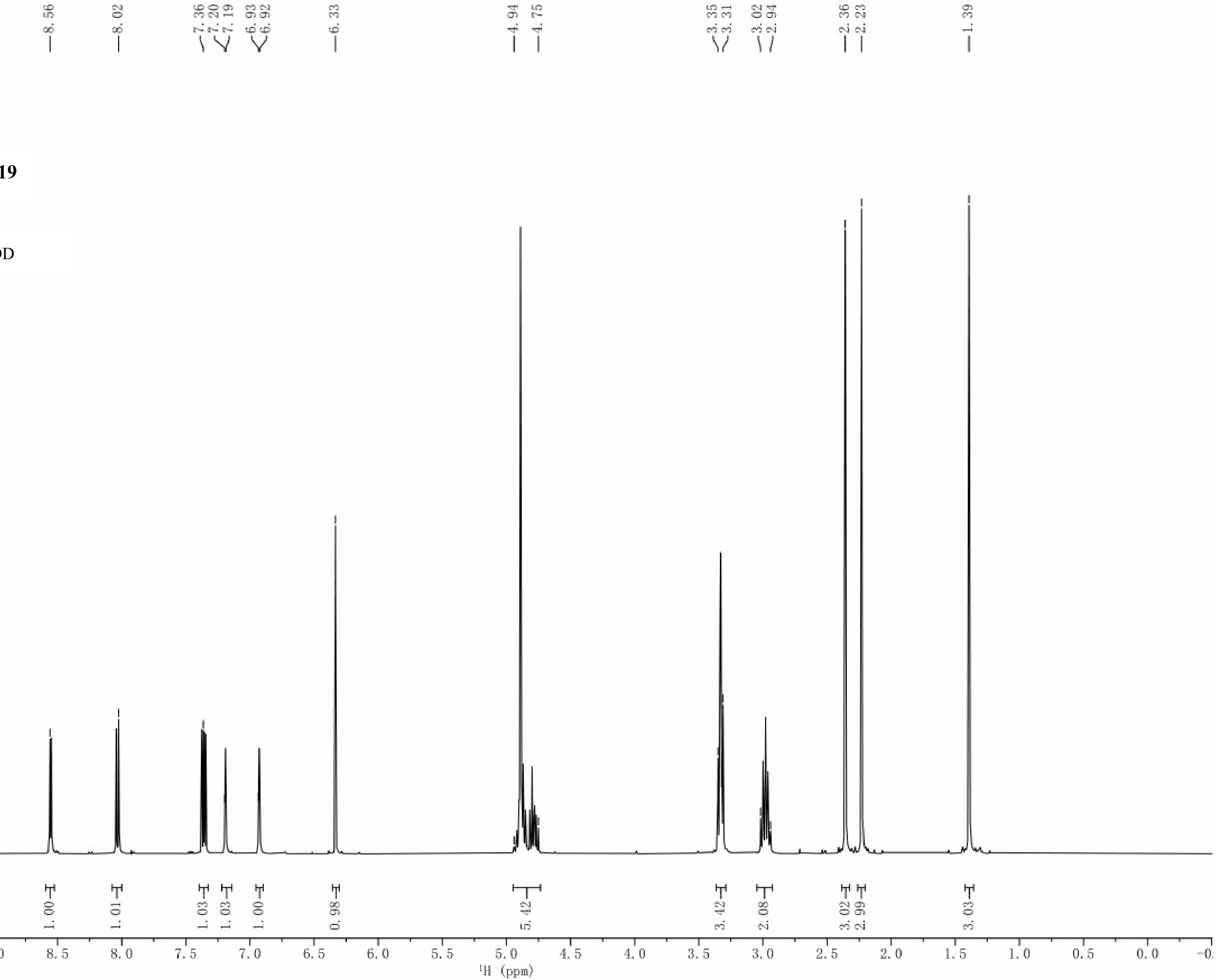

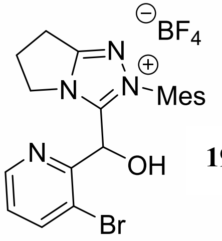

19

$^{13}C\{^{1}H\}$ NMR 101MHz d_4-CD_3OD

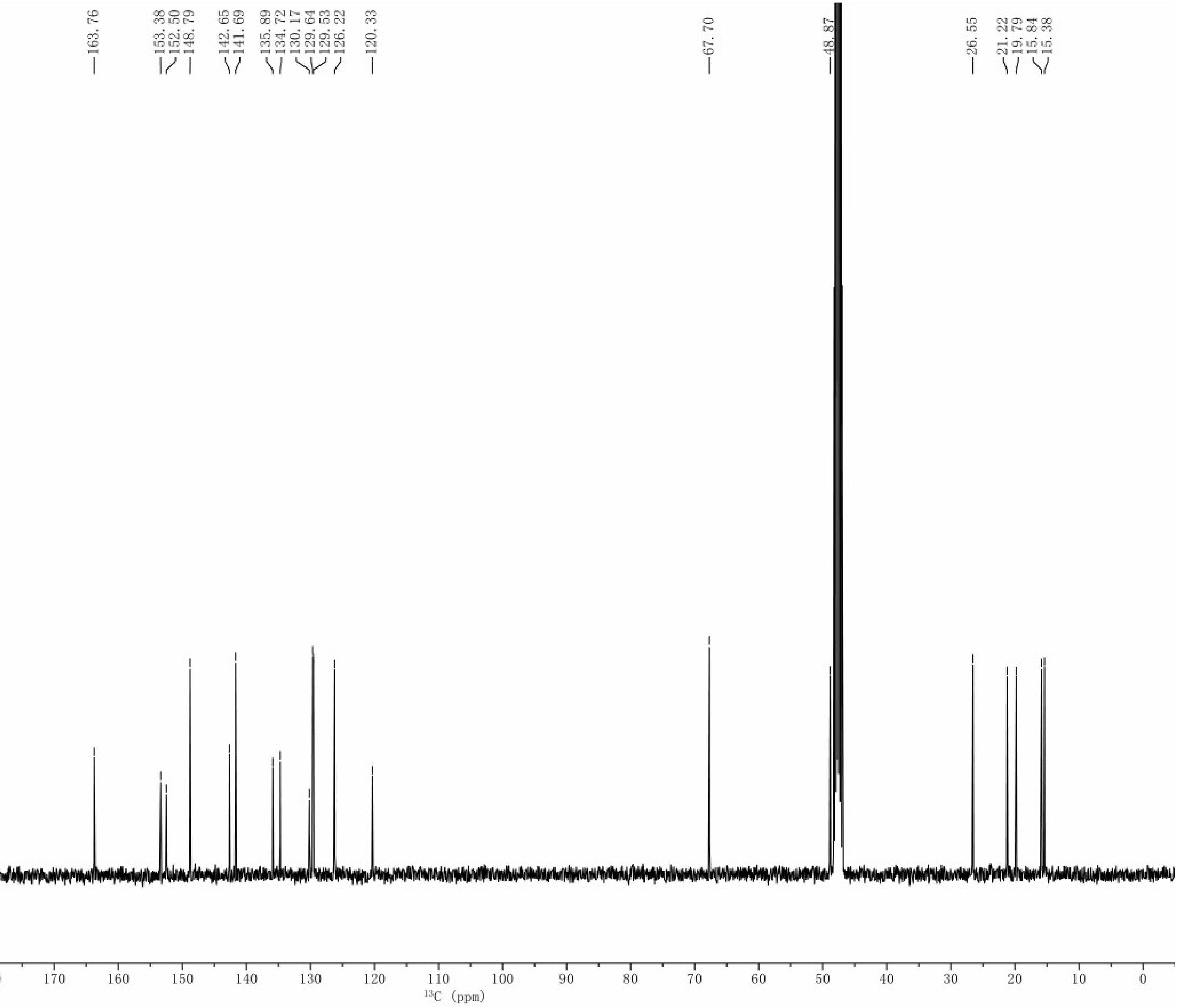

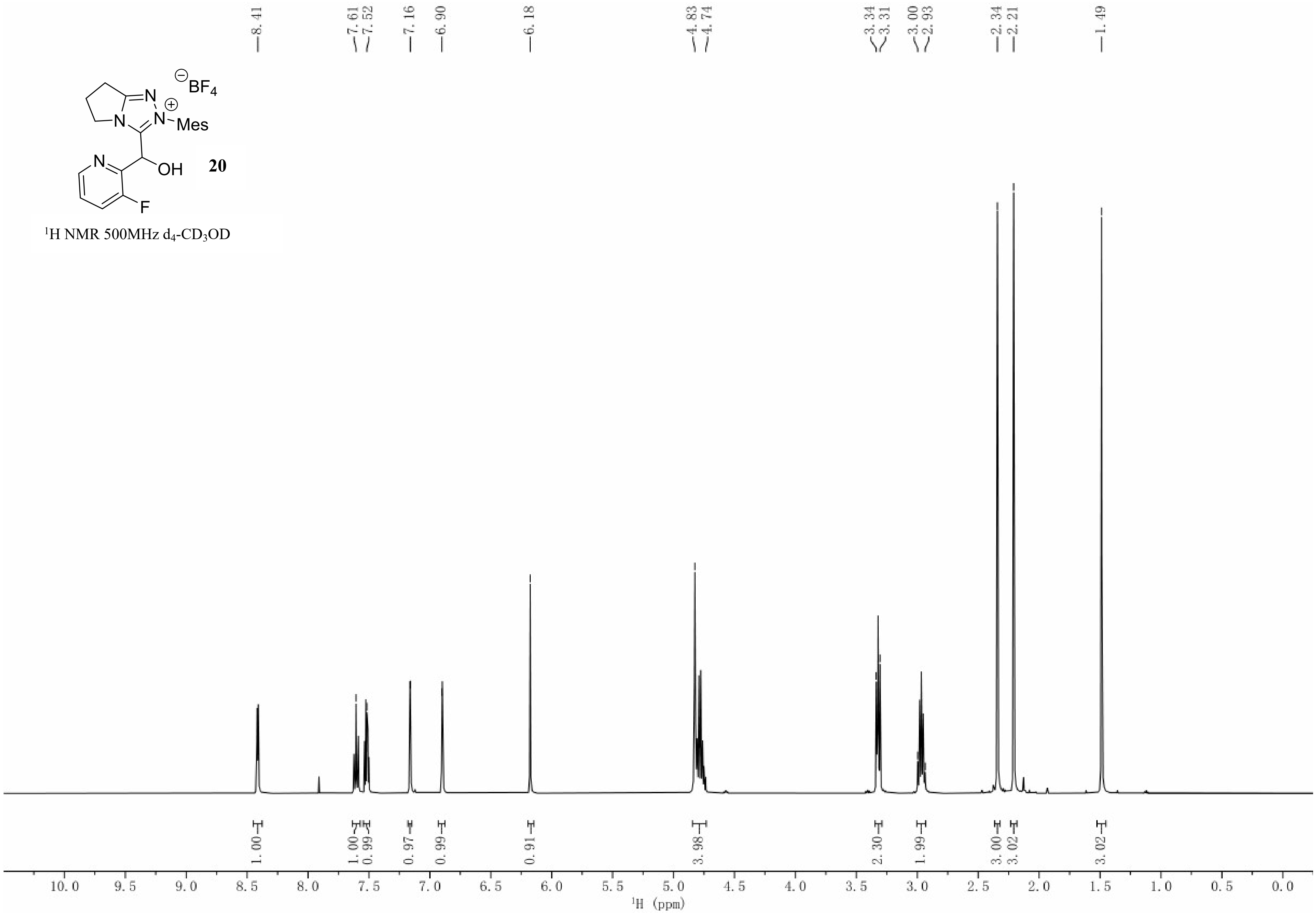

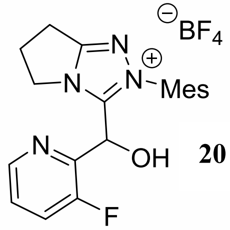

20

^{19}F NMR 470MHz d_4-CD_3OD

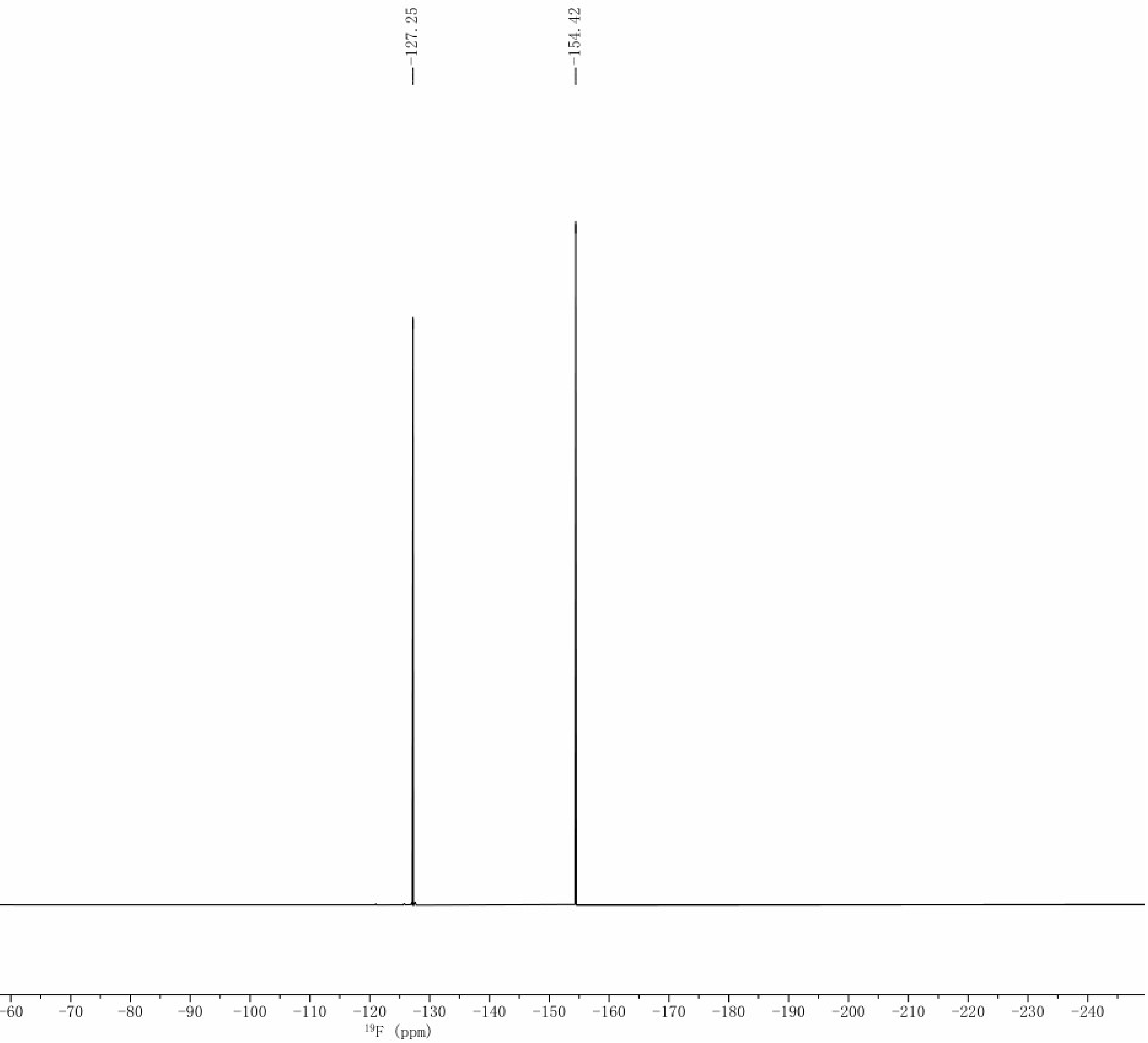

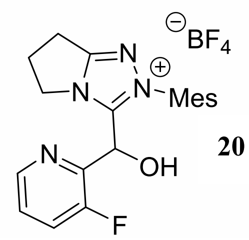

20

$^{13}C\{^1H\}$ NMR 126MHz d_4-CD_3OD

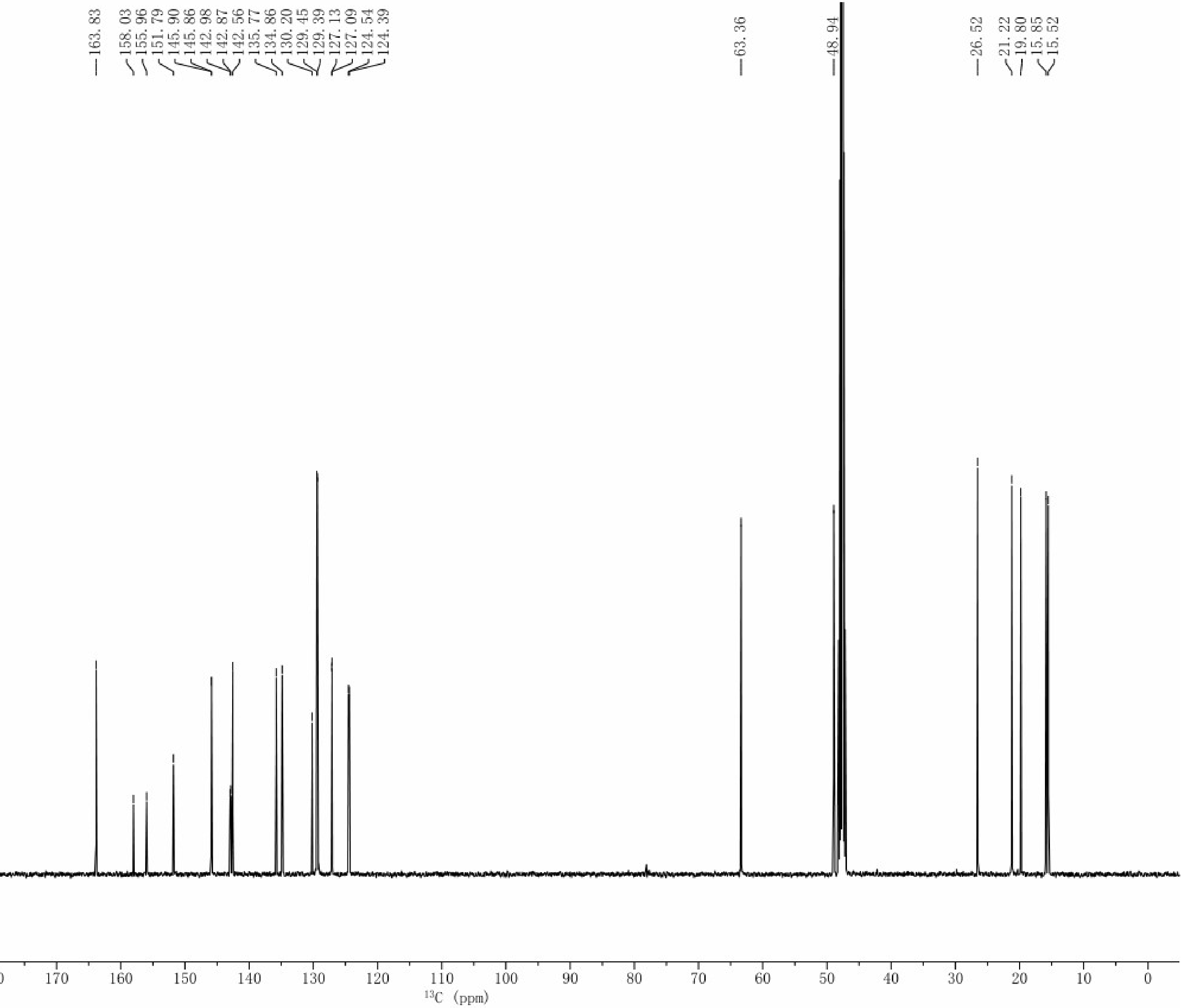

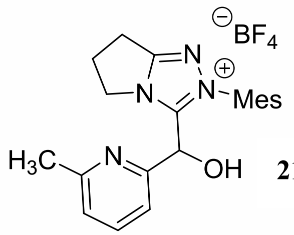

21

^{1}H NMR 400MHz d_4-CD_3OD

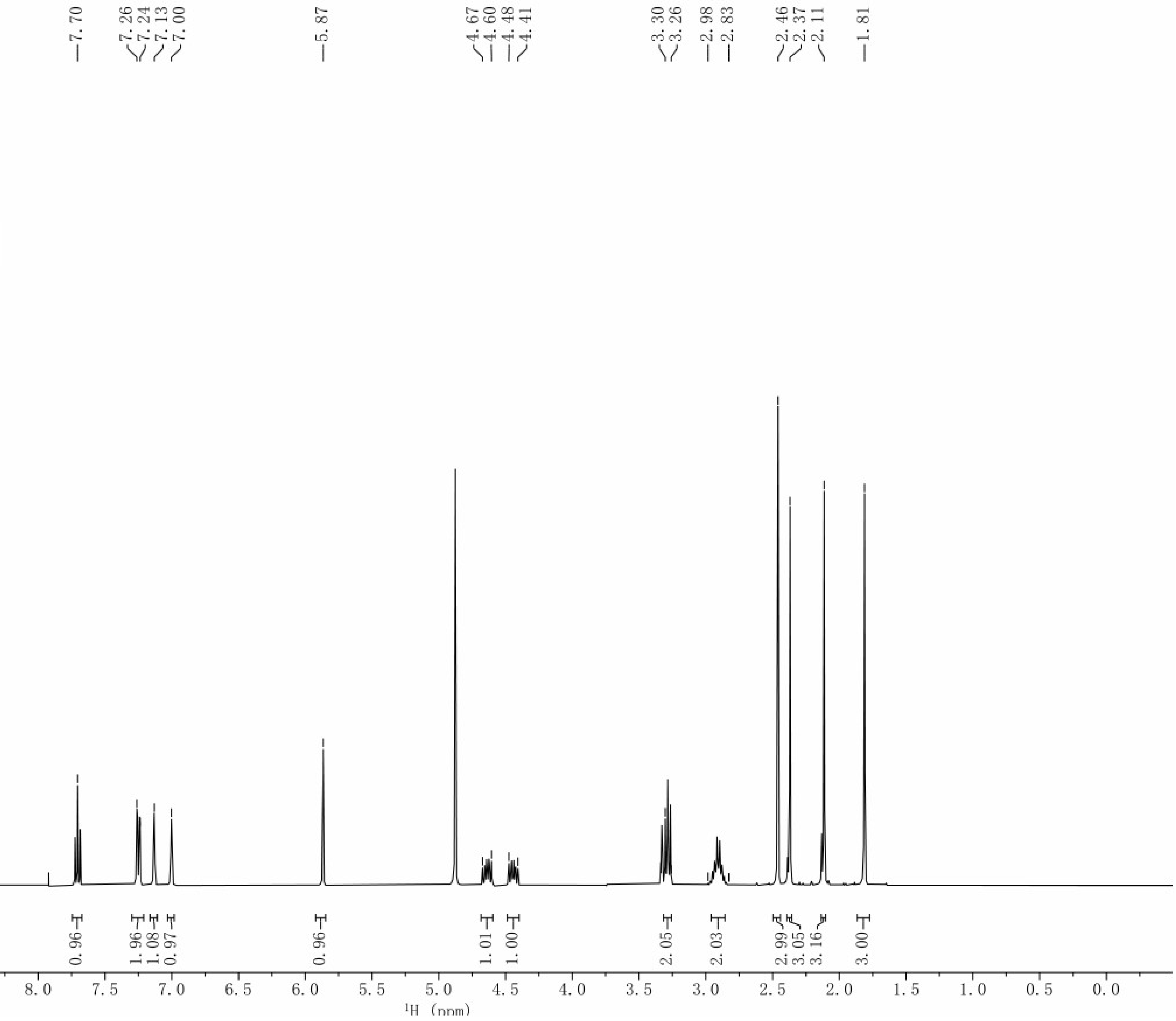

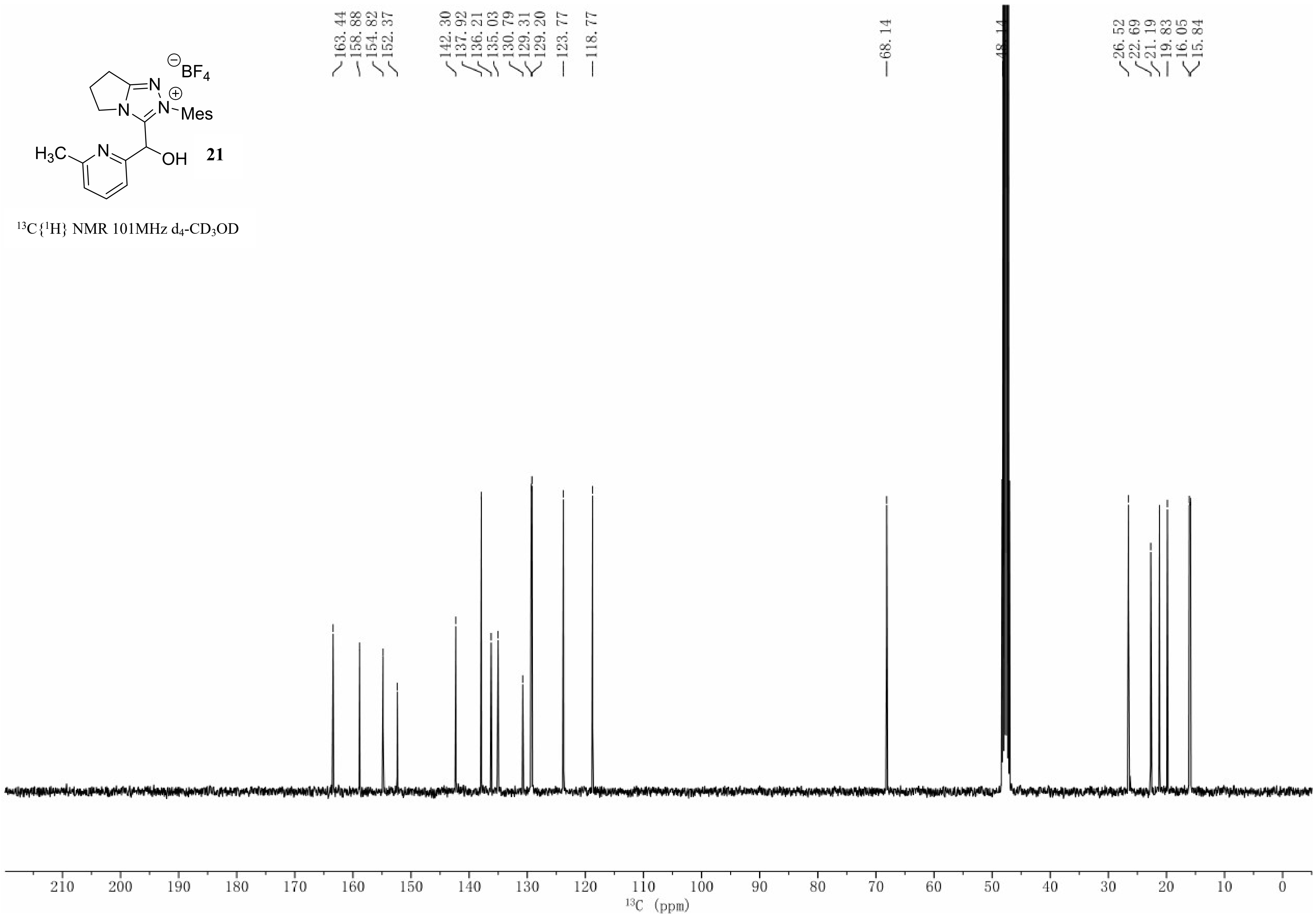

BF4
N
N
Mes
N
H3C
N
OH
21
13C{1H} NMR 101MHz d4-CD3OD
163.44
158.88
154.82
152.37
142.30
137.92
136.21
135.03
130.79
129.31
129.20
123.77
118.77
68.14
48.14
26.52
22.69
21.19
19.83
16.05
15.84
210 200 190 180 170 160 150 140 130 120 110 100 90 80 70 60 50 40 30 20 10 0
13C (ppm)

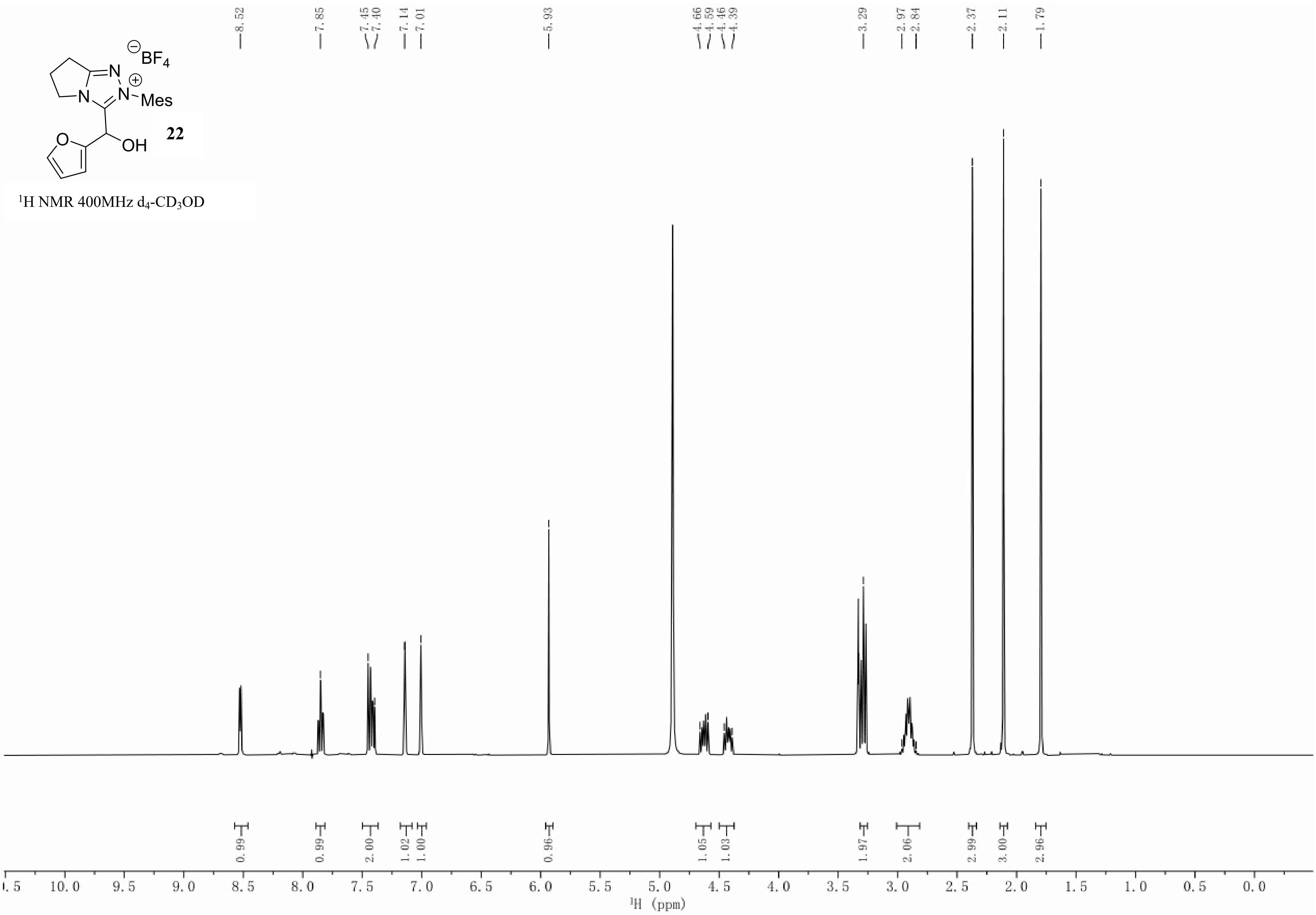

22

^{1}H NMR 400MHz d_4-CD_3OD

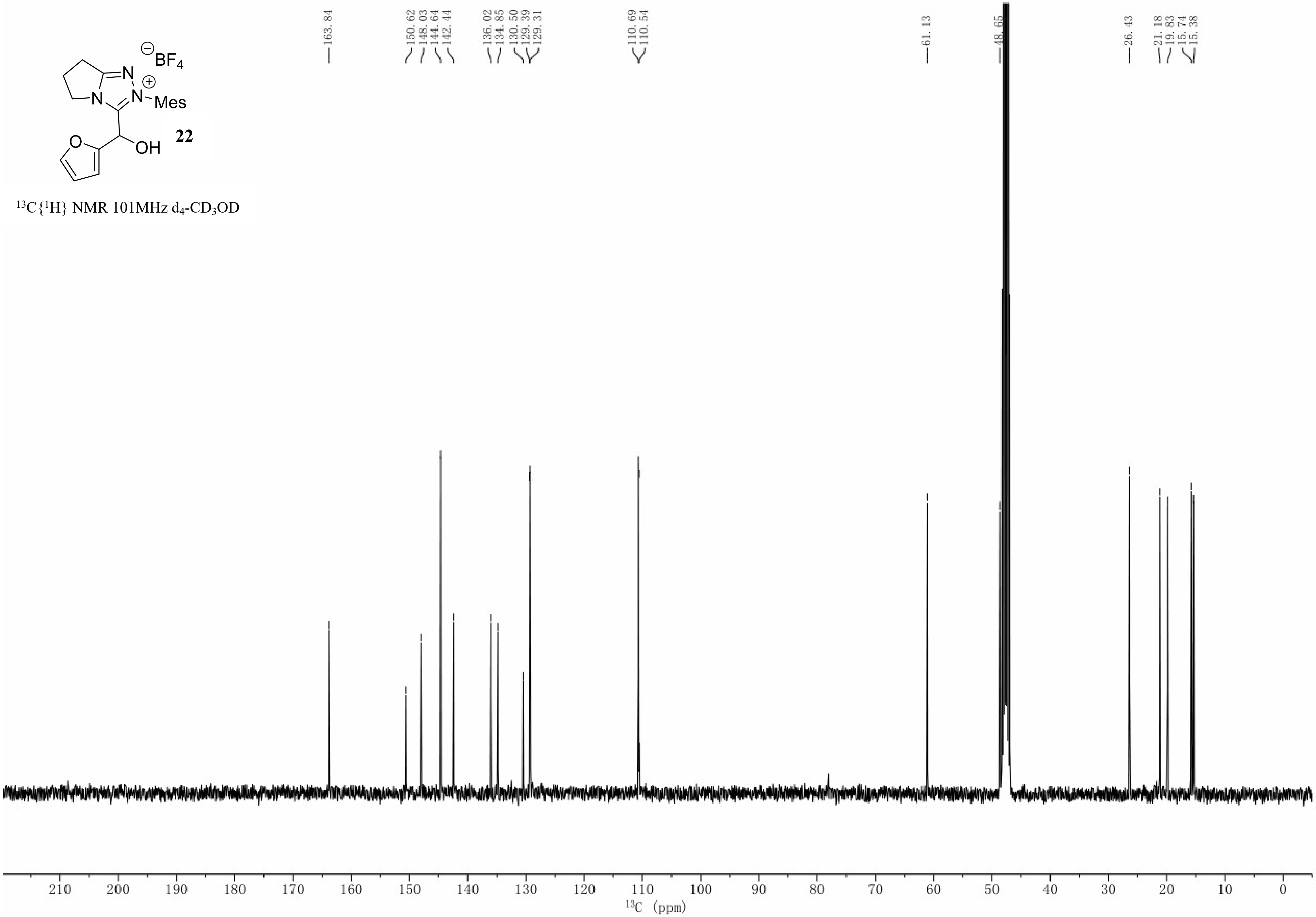

$^{13}C\{^{1}H\}$ NMR 101MHz d_4-CD_3OD

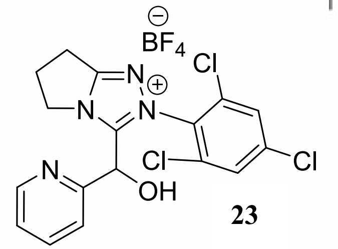

23

^{1}H NMR 400MHz d_4-CD_3OD

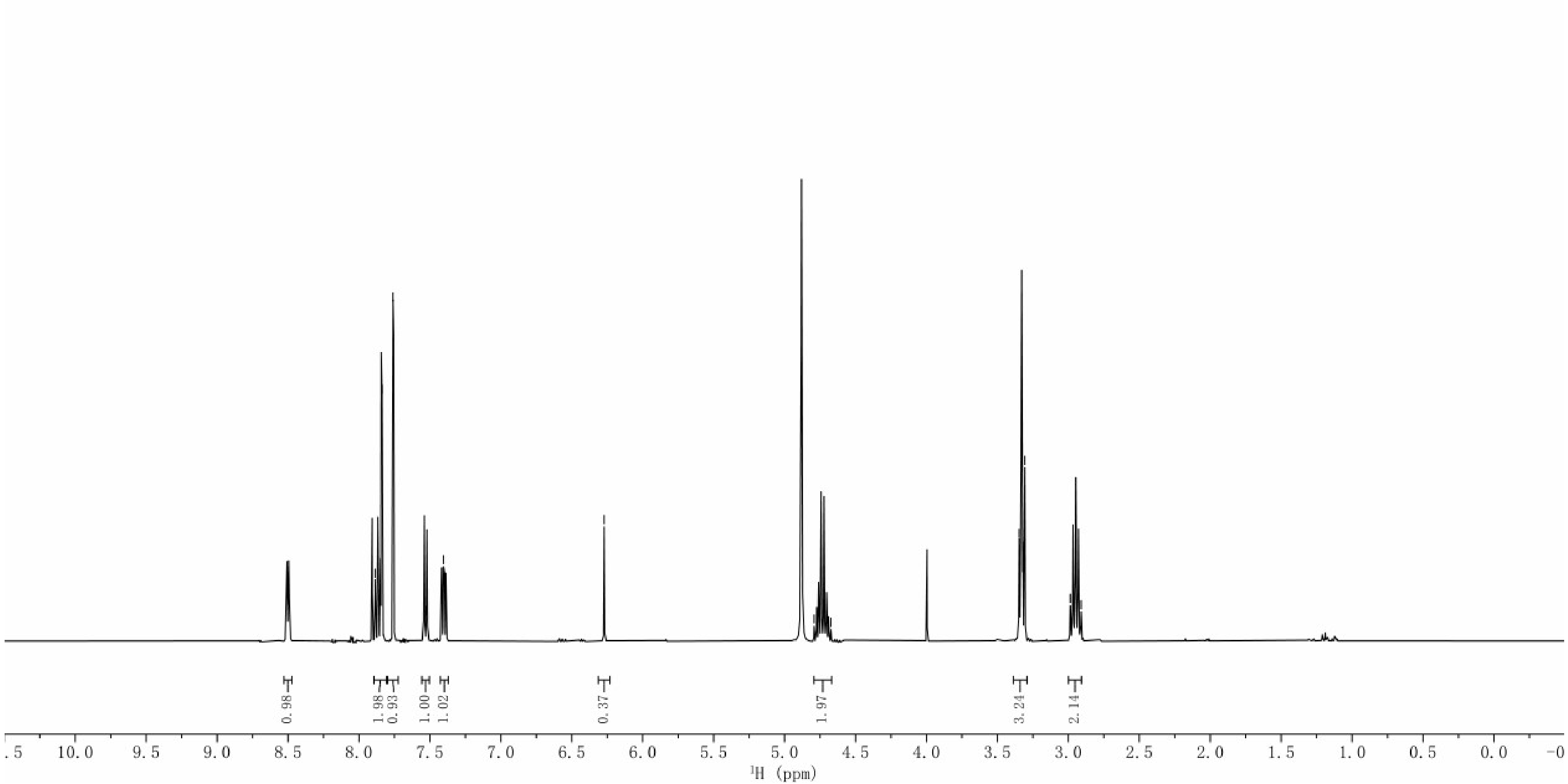

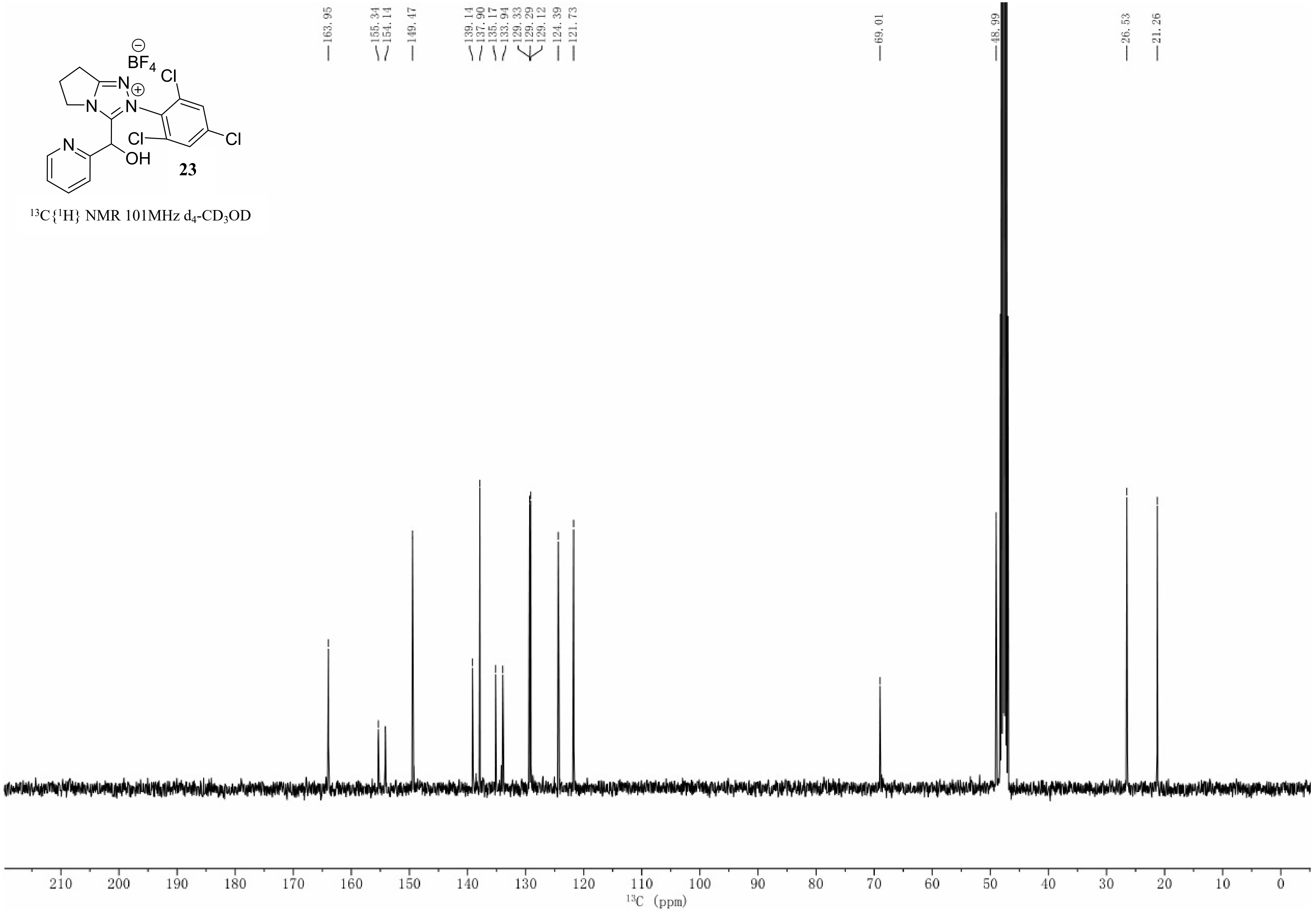

23

$^{13}C\{^{1}H\}$ NMR 101MHz d_4-CD_3OD

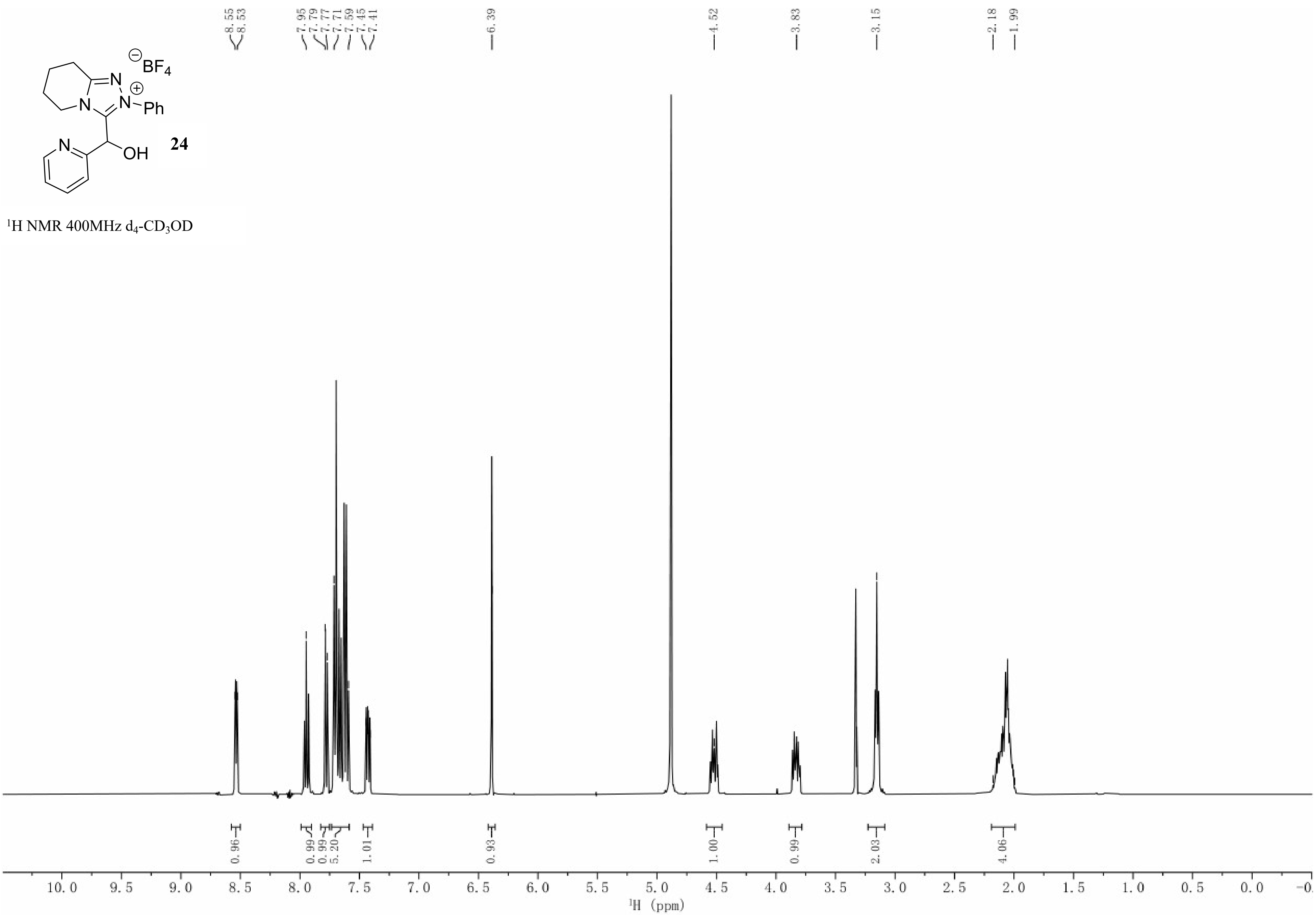
BF4
N
N
N
Ph
N
OH
24
1H NMR 400MHz d4-CD3OD
8.55
8.53
7.95
7.79
7.77
7.71
7.59
7.45
7.41
6.39
4.52
3.83
3.15
2.18
1.99
0.96
0.99
0.99
5.20
1.01
0.93
1.00
0.99
2.03
4.06
10.0 9.5 9.0 8.5 8.0 7.5 7.0 6.5 6.0 5.5 5.0 4.5 4.0 3.5 3.0 2.5 2.0 1.5 1.0 0.5 0.0
1H (ppm)

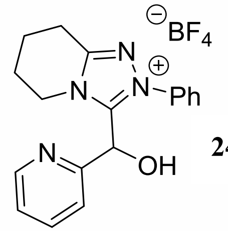

24

$^{13}C\{^{1}H\}$ NMR 101MHz d_4-CD_3OD

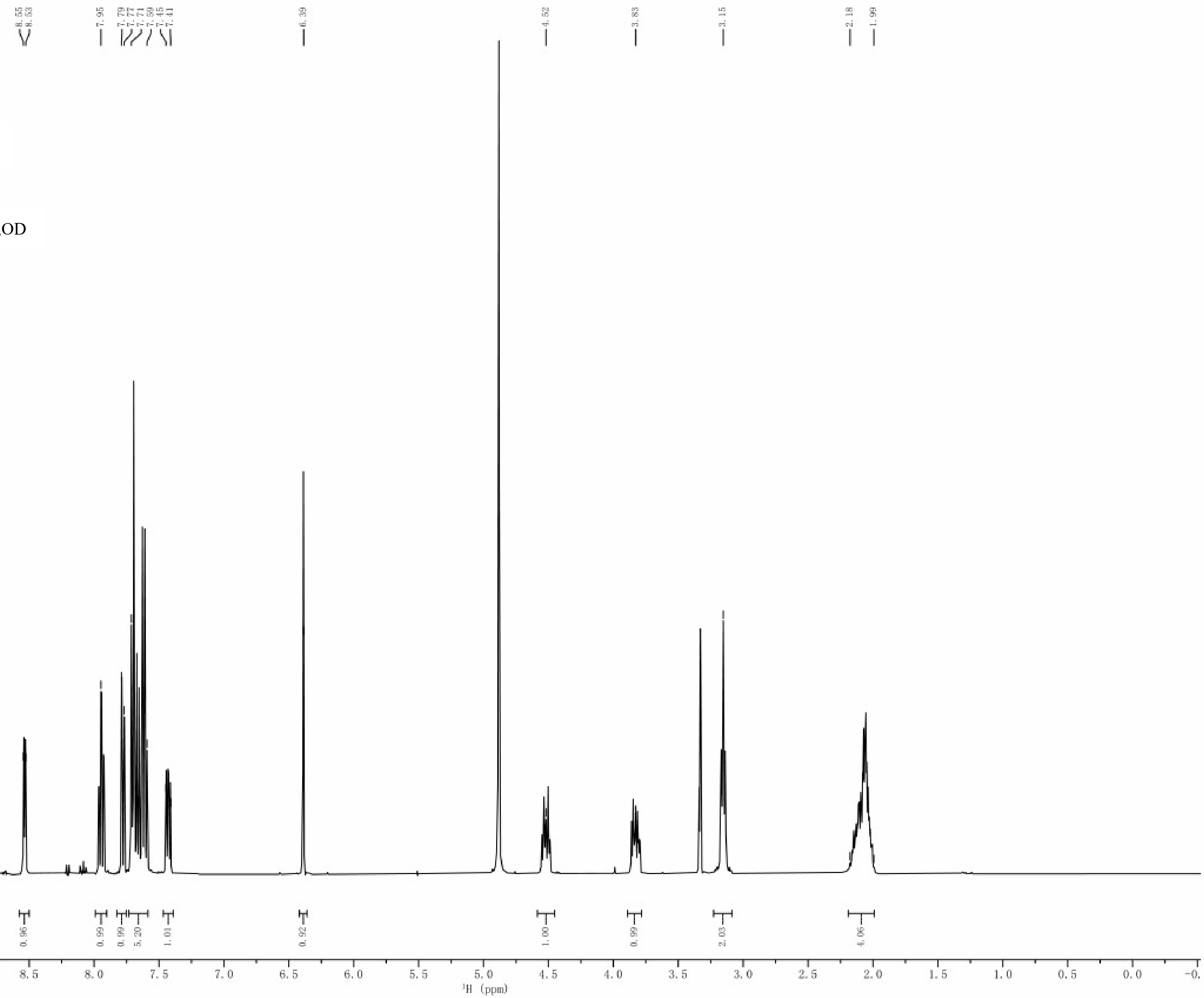

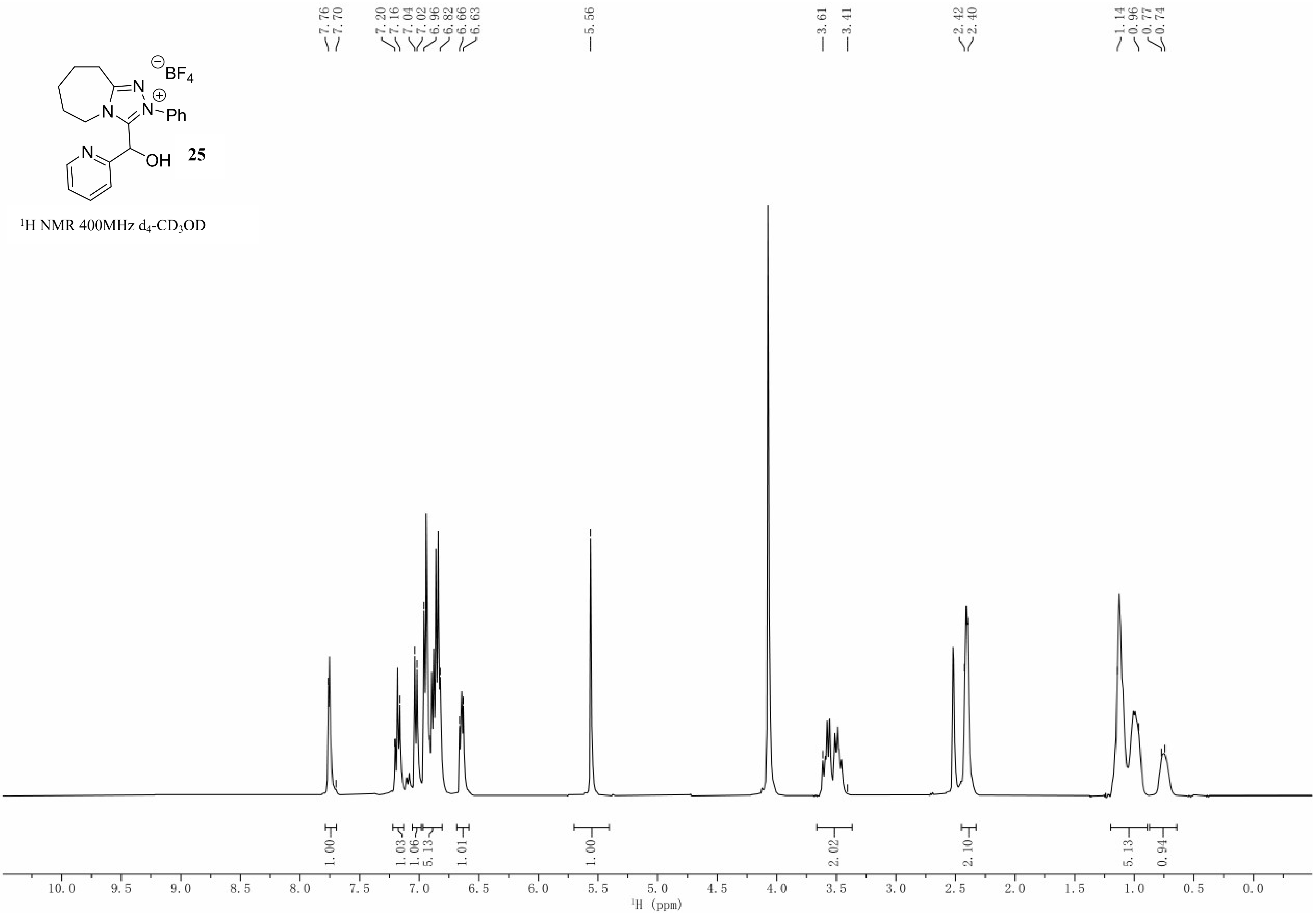
7.76
7.70
7.20
7.16
7.04
7.02
6.96
6.82
6.66
6.63
5.56
3.61
3.41
2.42
2.40
1.14
0.96
0.77
0.74
BF4
N
N
Ph
N
N
OH
25
1H NMR 400MHz d4-CD3OD
1.00
1.03
1.06
5.13
1.01
1.00
2.02
2.10
5.13
0.94
10.0
9.5
9.0
8.5
8.0
7.5
7.0
6.5
6.0
5.5
5.0
4.5
4.0
3.5
3.0
2.5
2.0
1.5
1.0
0.5
0.0
1H (ppm)

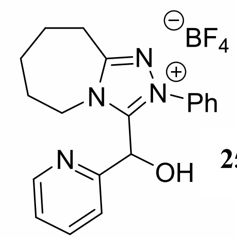

25

$^{13}C\{^1H\}$ NMR 101MHz d_4-CD_3OD

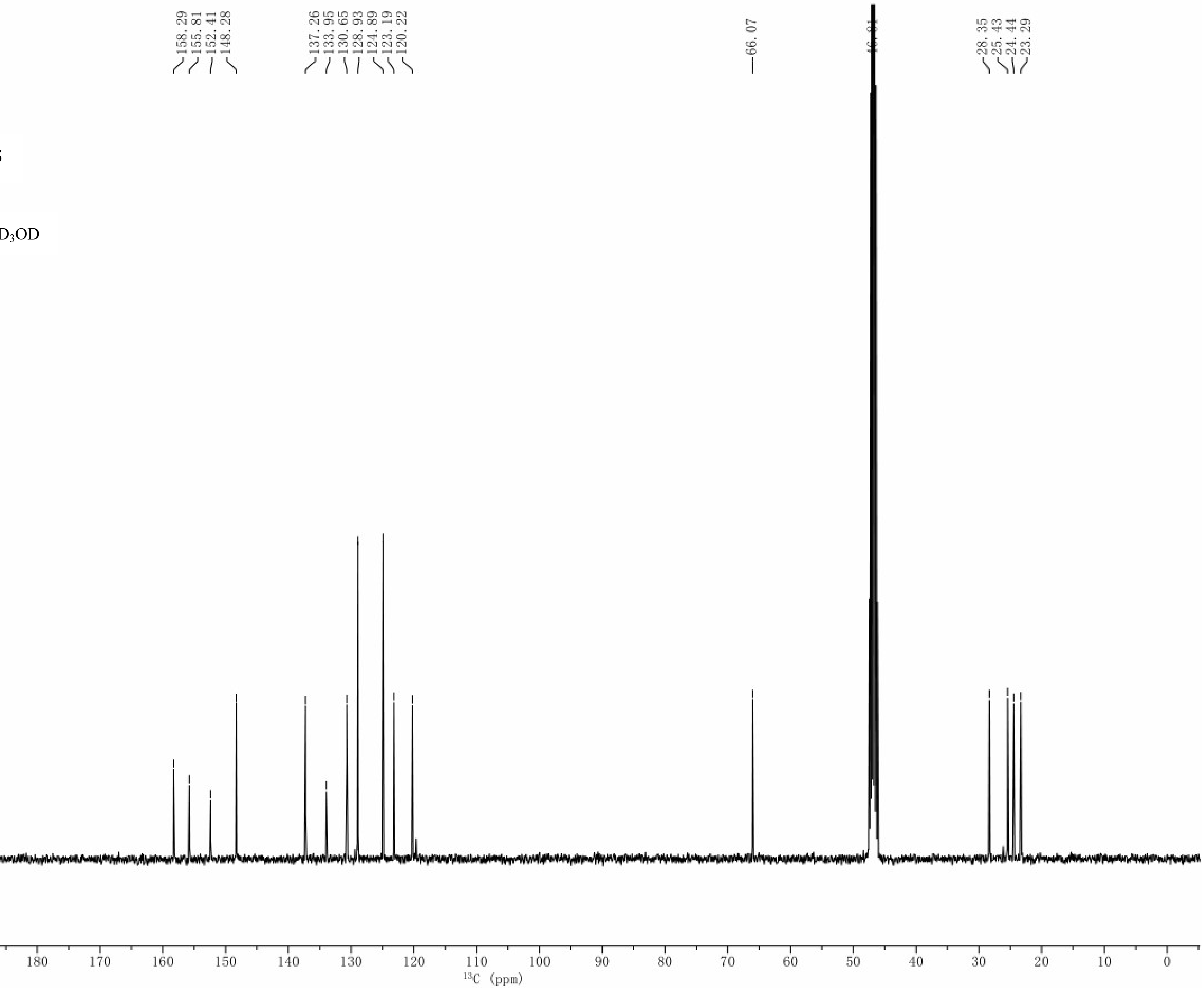

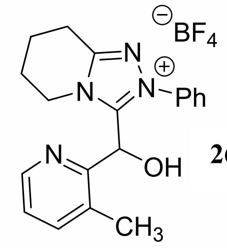

26

^{1}H NMR 400MHz d_4-CD_3OD

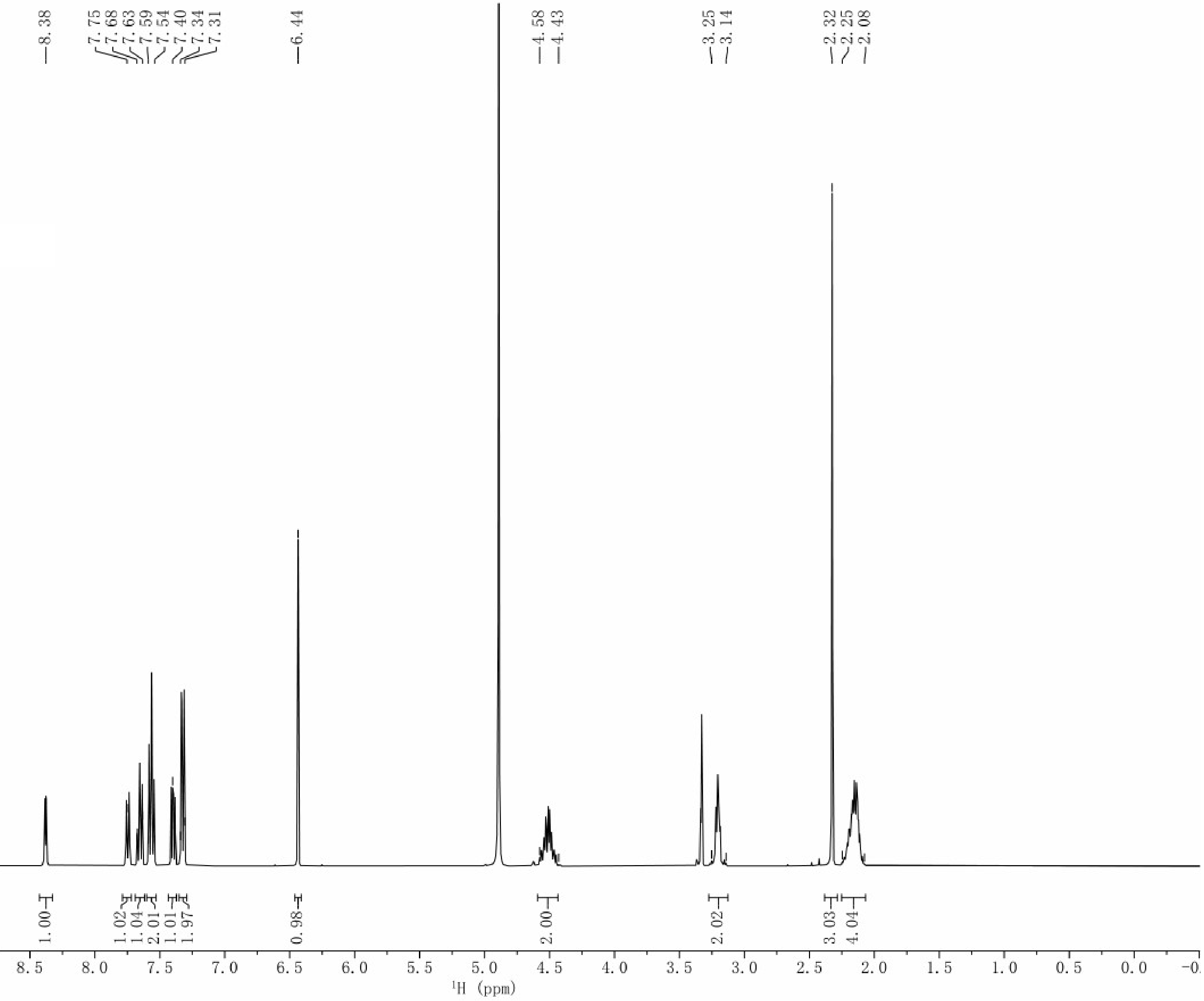

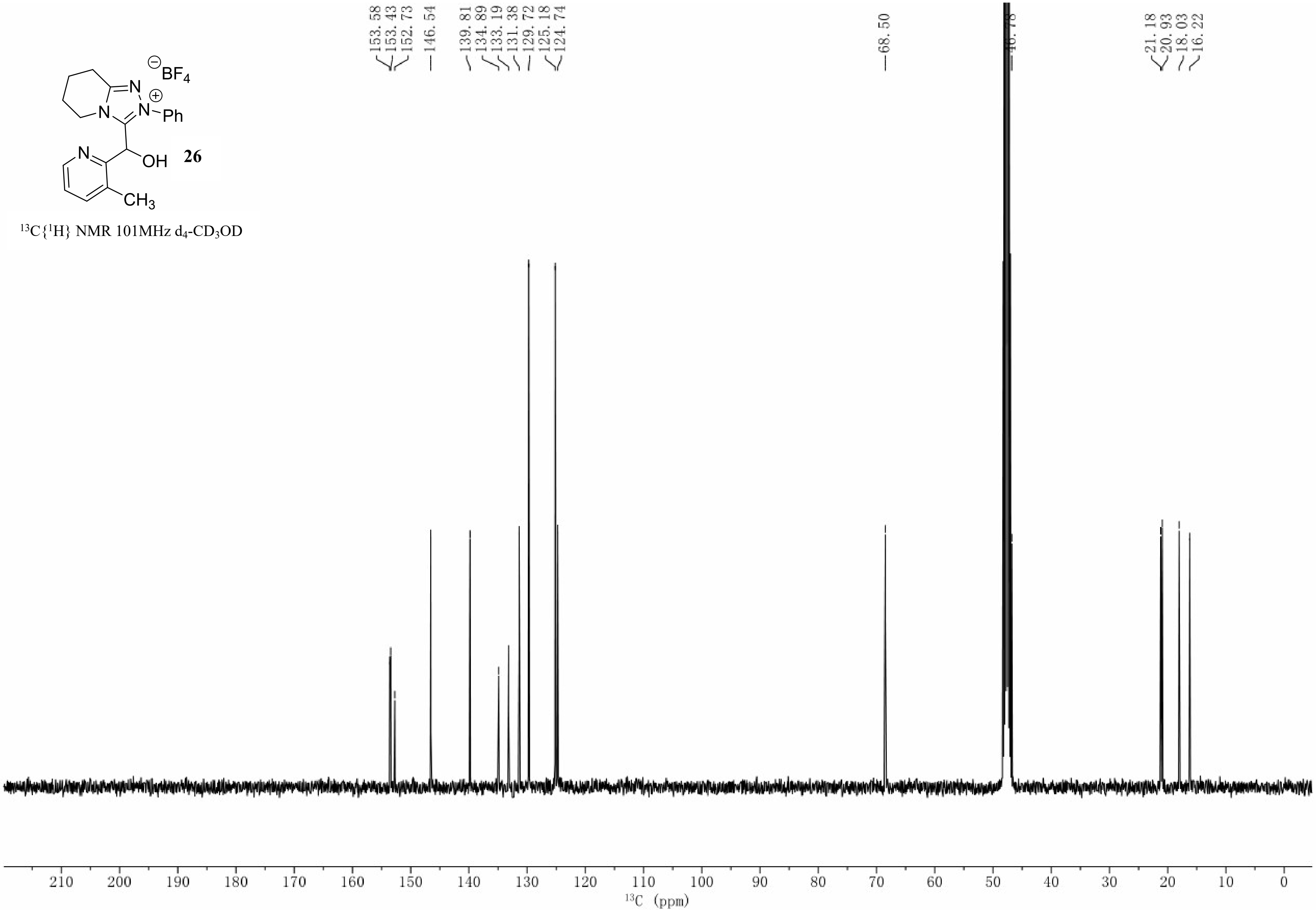
BF4
N
N
N
Ph
N
OH
CH3
26
13C{1H} NMR 101MHz d4-CD3OD
153.58
153.43
152.73
146.54
139.81
134.89
133.19
131.38
129.72
125.18
124.74
68.50
21.18
20.93
18.03
16.22
210
200
190
180
170
160
150
140
130
120
110
100
90
80
70
60
50
40
30
20
10
0
13C (ppm)

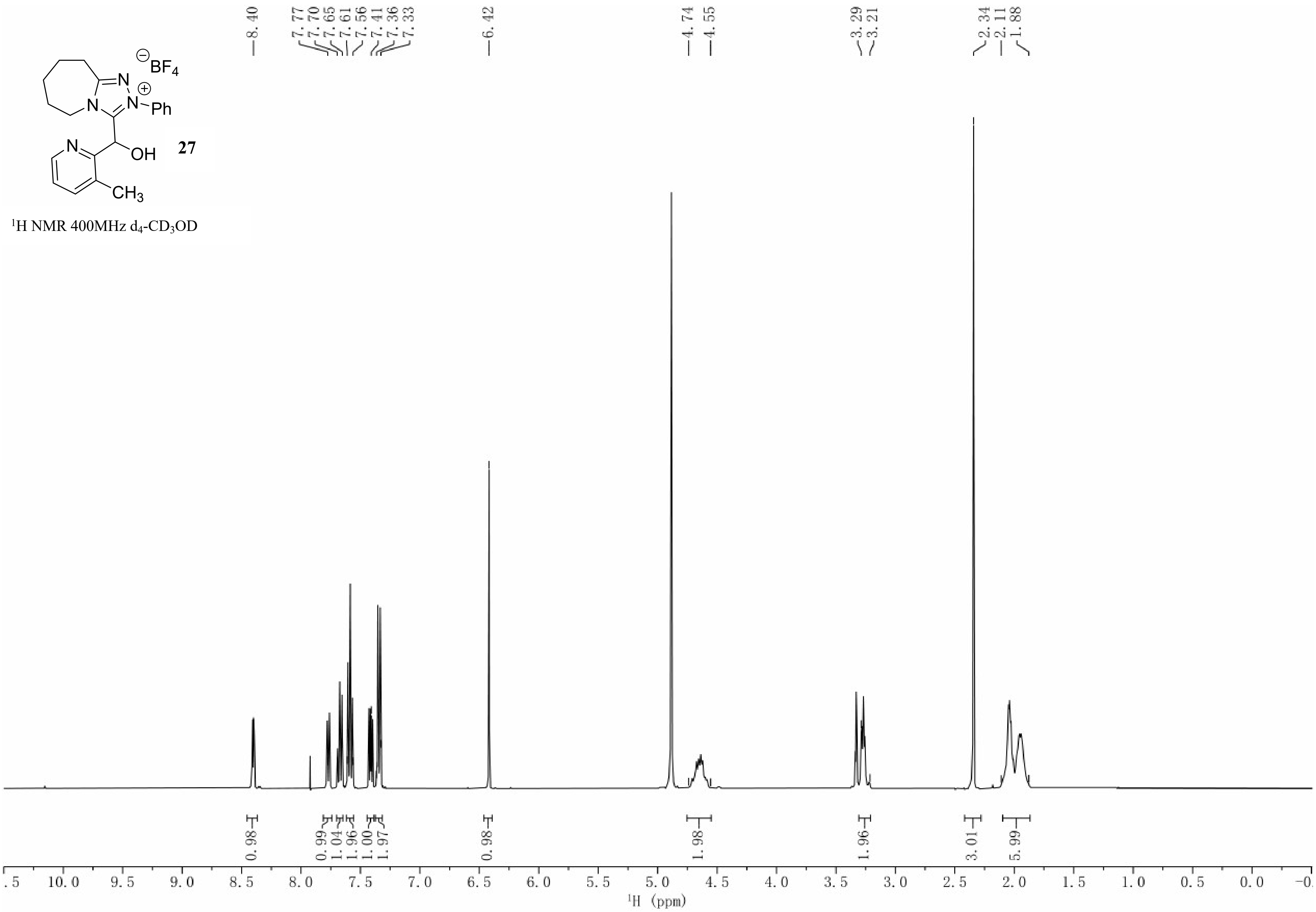

BF4
N
N
N
Ph
N
OH
CH3
27
1H NMR 400MHz d4-CD3OD
8.40
7.77
7.70
7.65
7.61
7.56
7.41
7.36
7.33
6.42
4.74
4.55
3.29
3.21
2.34
2.11
1.88
0.98
0.99
1.04
1.96
1.00
1.97
0.98
1.98
1.96
3.01
5.99
.5
10.0
9.5
9.0
8.5
8.0
7.5
7.0
6.5
6.0
5.5
5.0
4.5
4.0
3.5
3.0
2.5
2.0
1.5
1.0
0.5
0.0
-0.
1H (ppm)

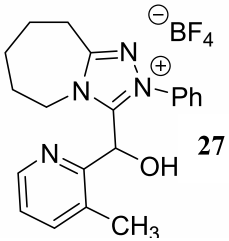

27

$^{13}C\{^{1}H\}$ NMR 101MHz d_4-CD_3OD

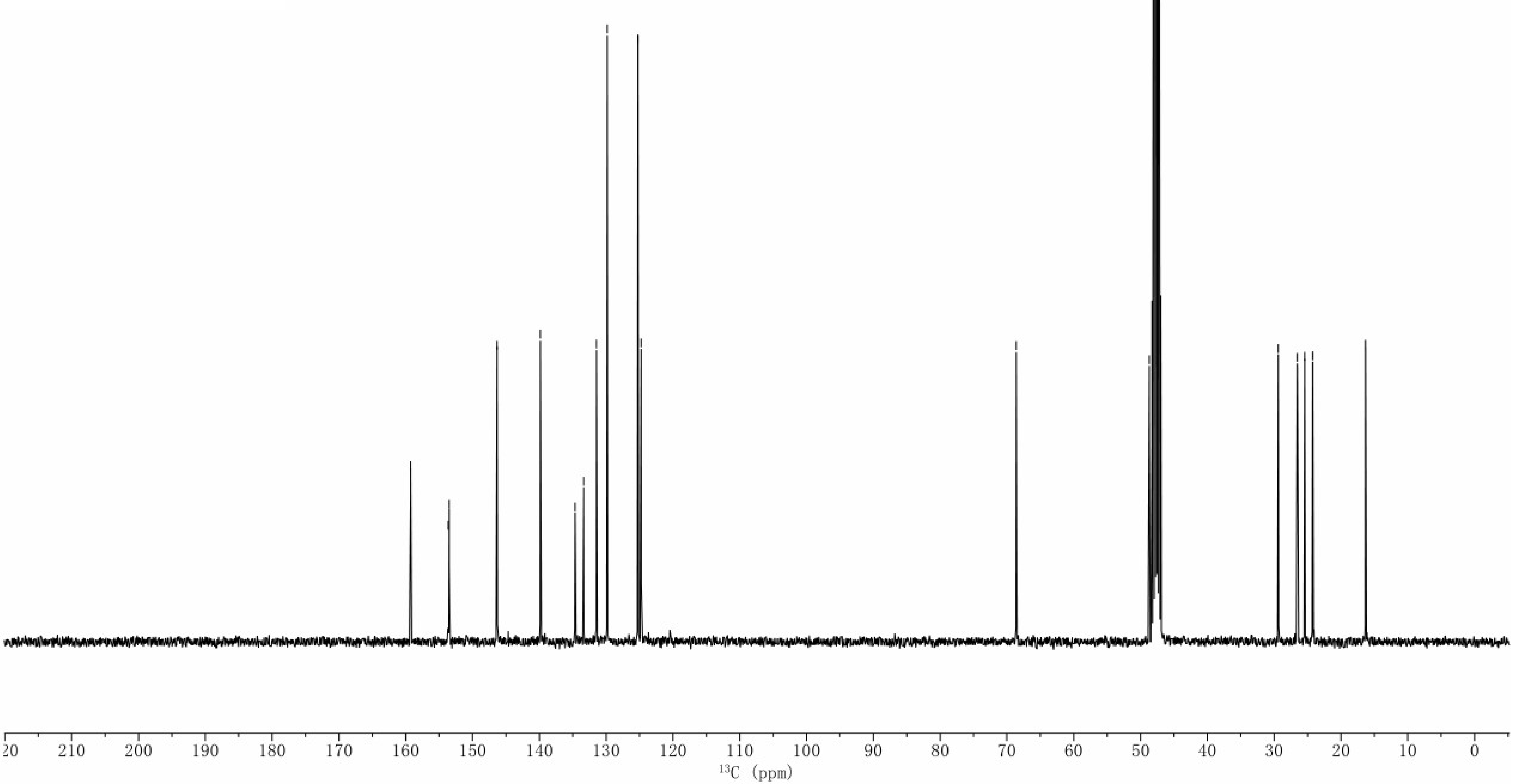

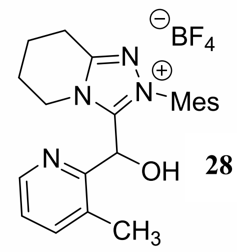

28

^{1}H NMR 400MHz d_4-CD_3OD

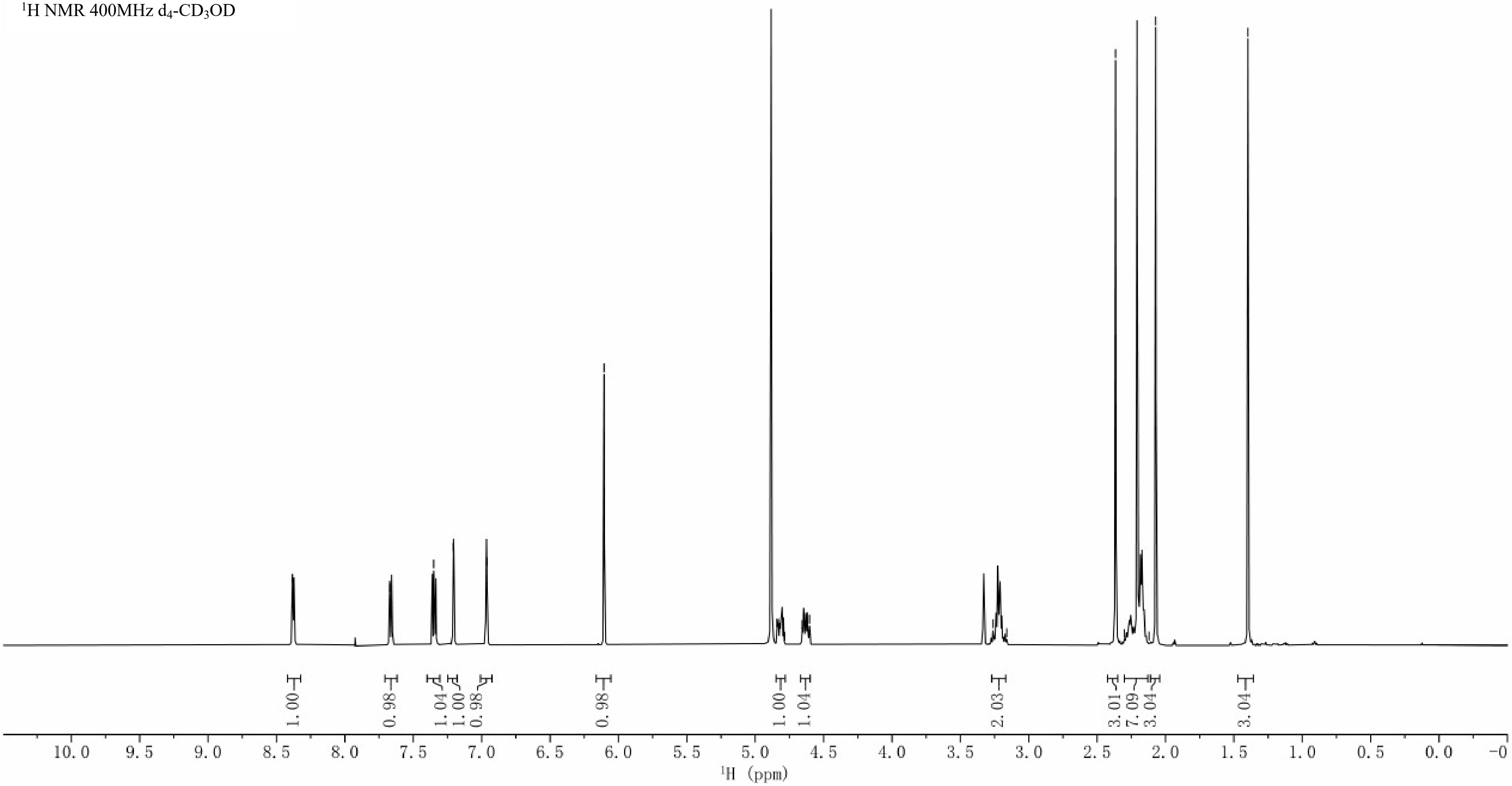

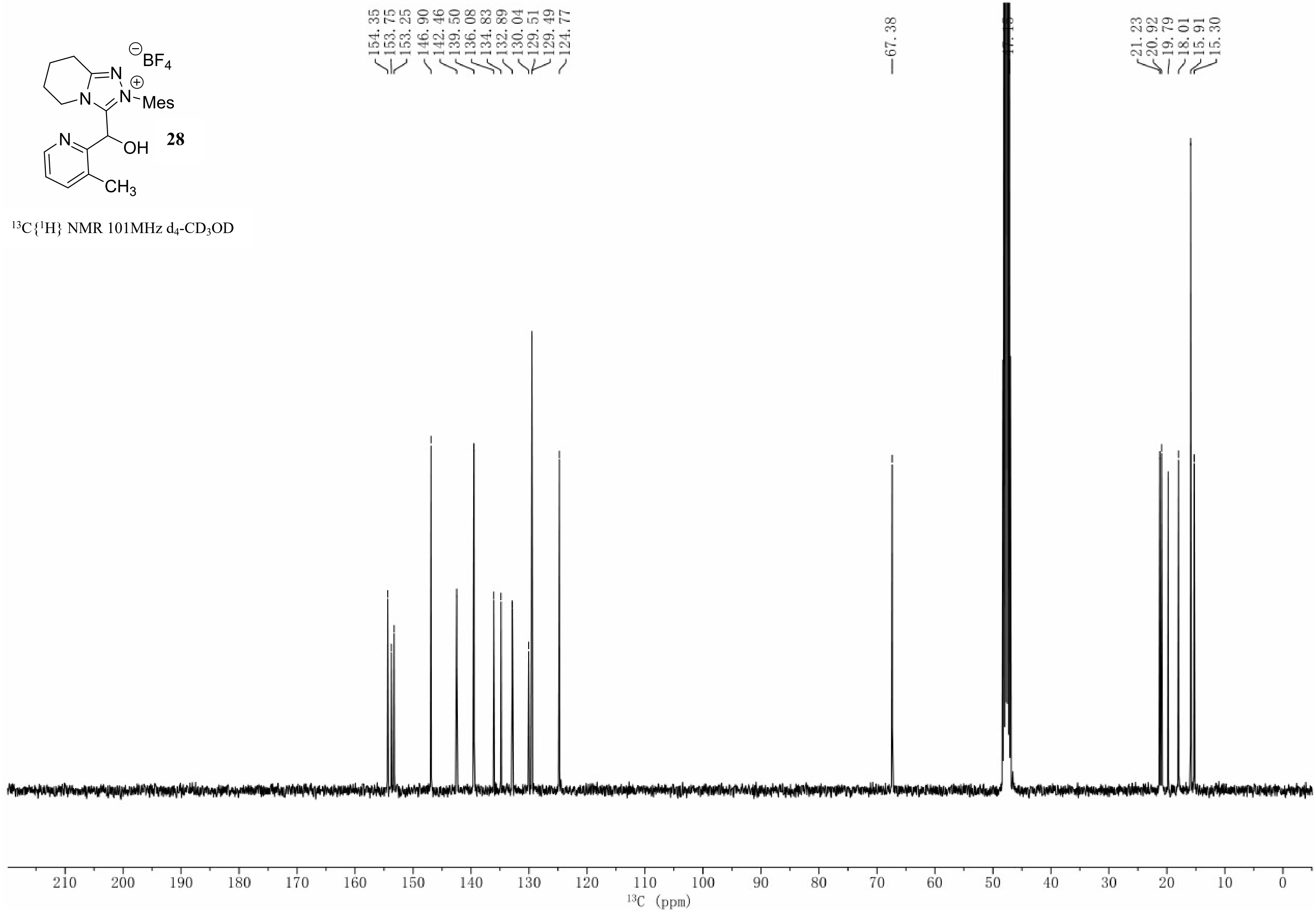

BF4
N
N
Mes
N
OH
CH3
28
$^{13}C\{^{1}H\}$ NMR 101MHz d_4-CD_3OD
154.35
153.75
153.25
146.90
142.46
139.50
136.08
134.83
132.89
130.04
129.51
129.49
124.77
67.38
21.23
20.92
19.79
18.01
15.91
15.30
210 200 190 180 170 160 150 140 130 120 110 100 90 80 70 60 50 40 30 20 10 0
^{13}C (ppm)

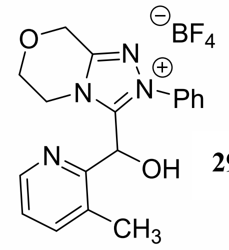

29

^{1}H NMR 400MHz d_4-CD_3OD

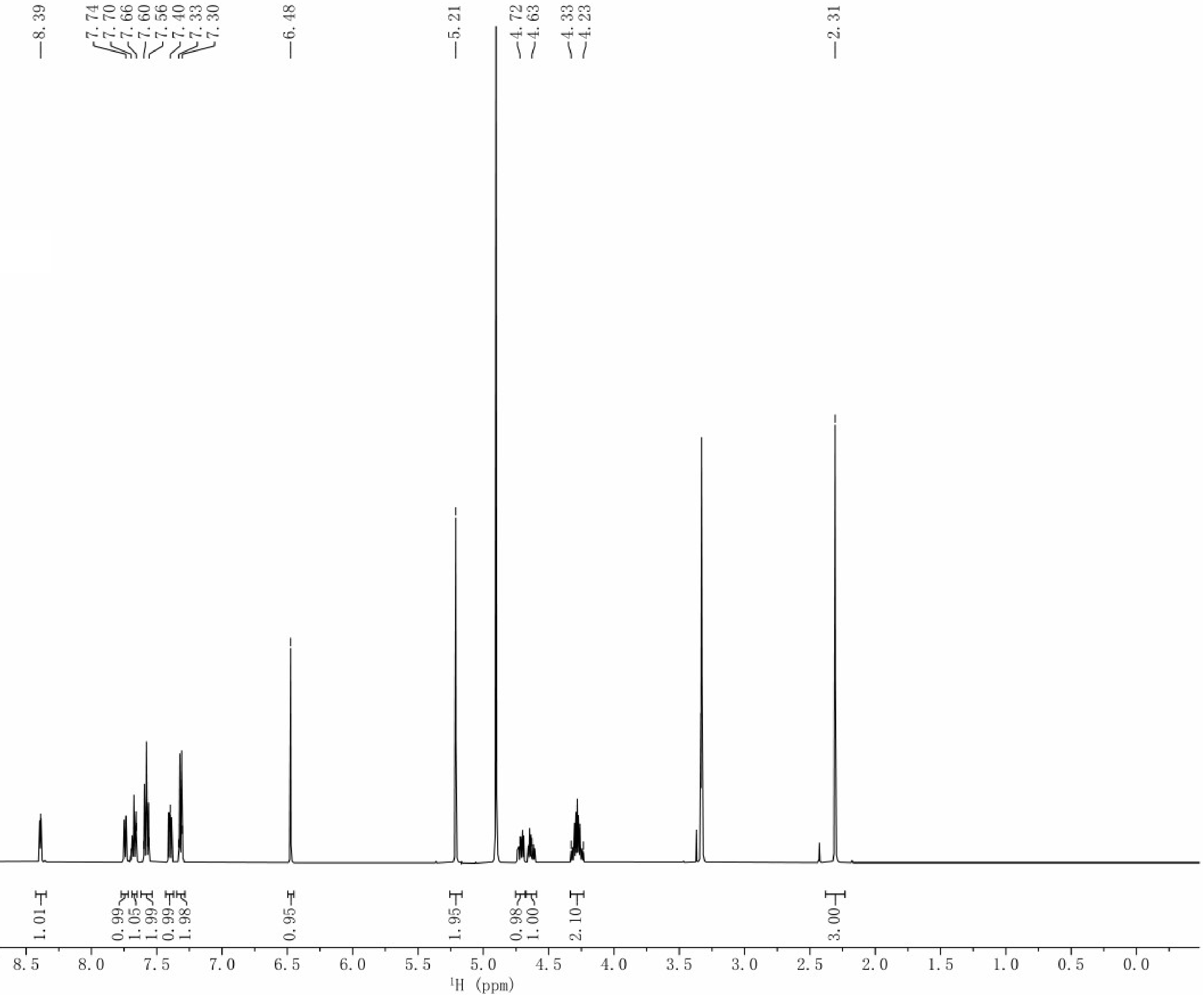

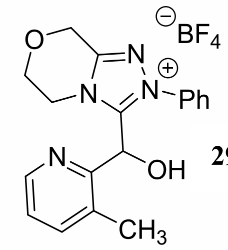

29

$^{13}C\{^{1}H\}$ NMR 101MHz d_4-CD_3OD

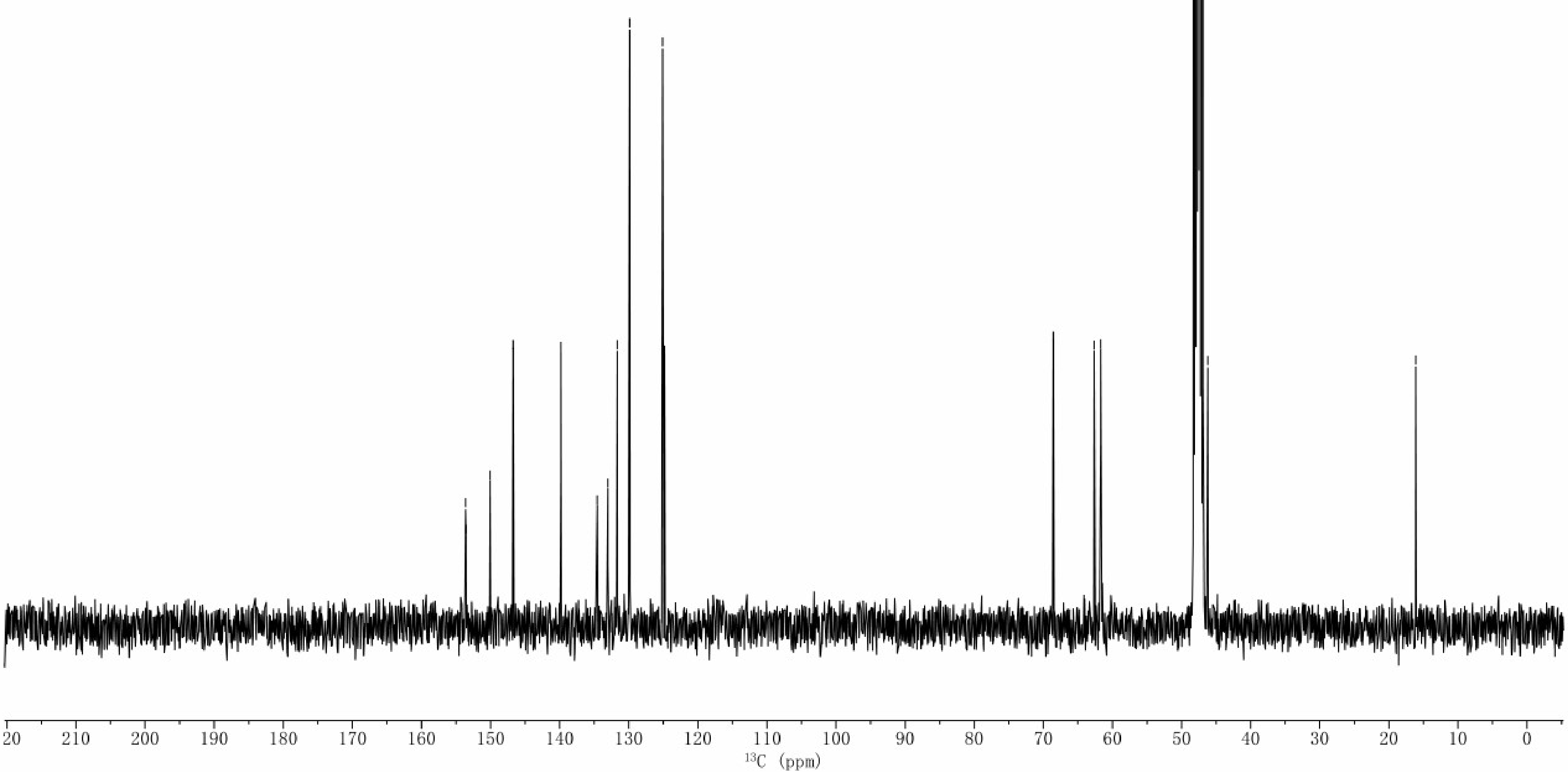

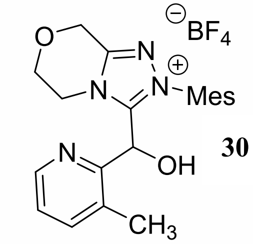

30

^{1}H NMR 400MHz d_4-CD_3OD

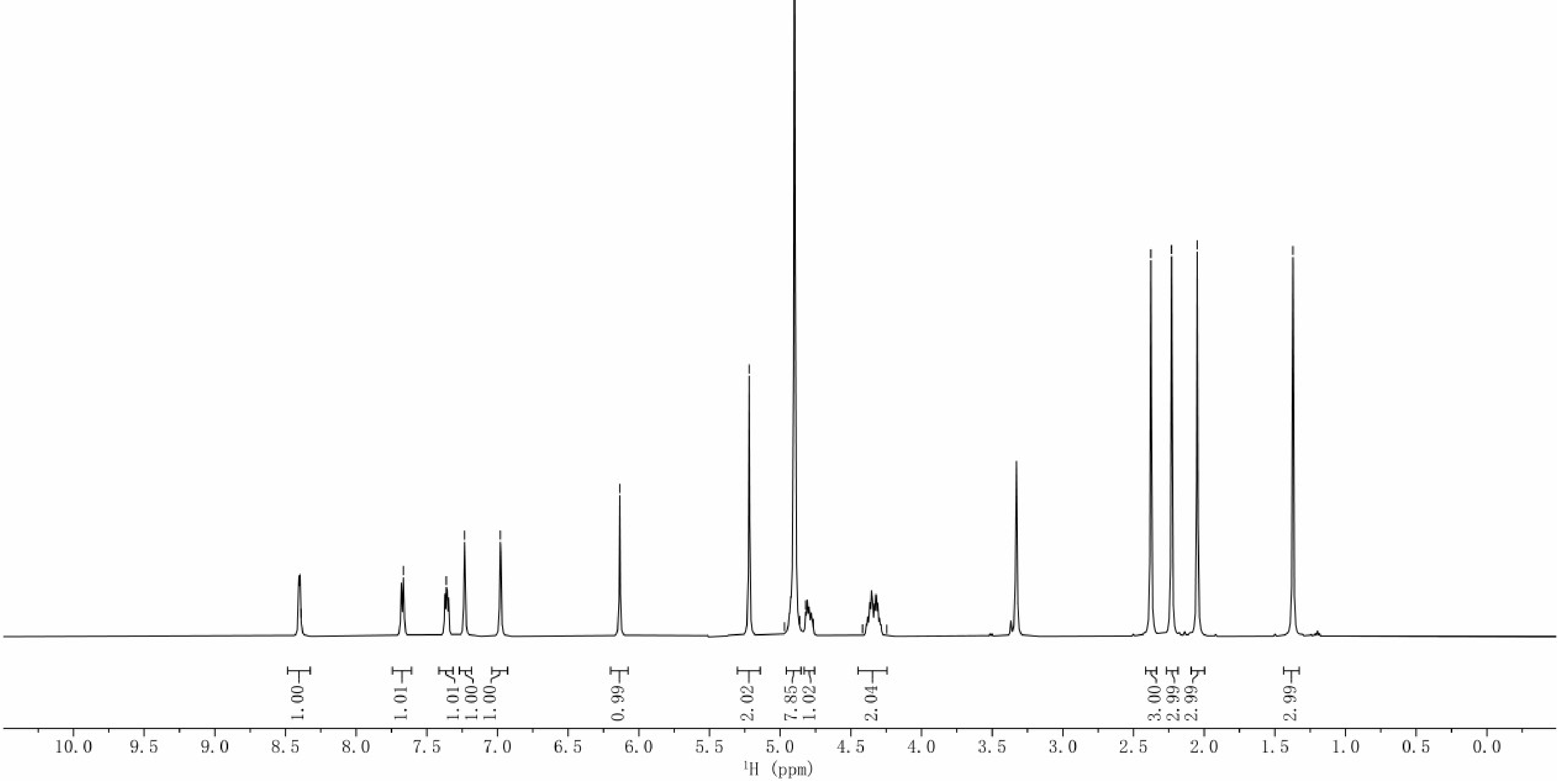

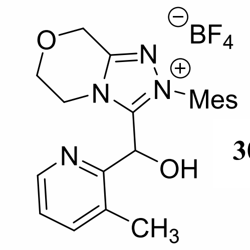

30

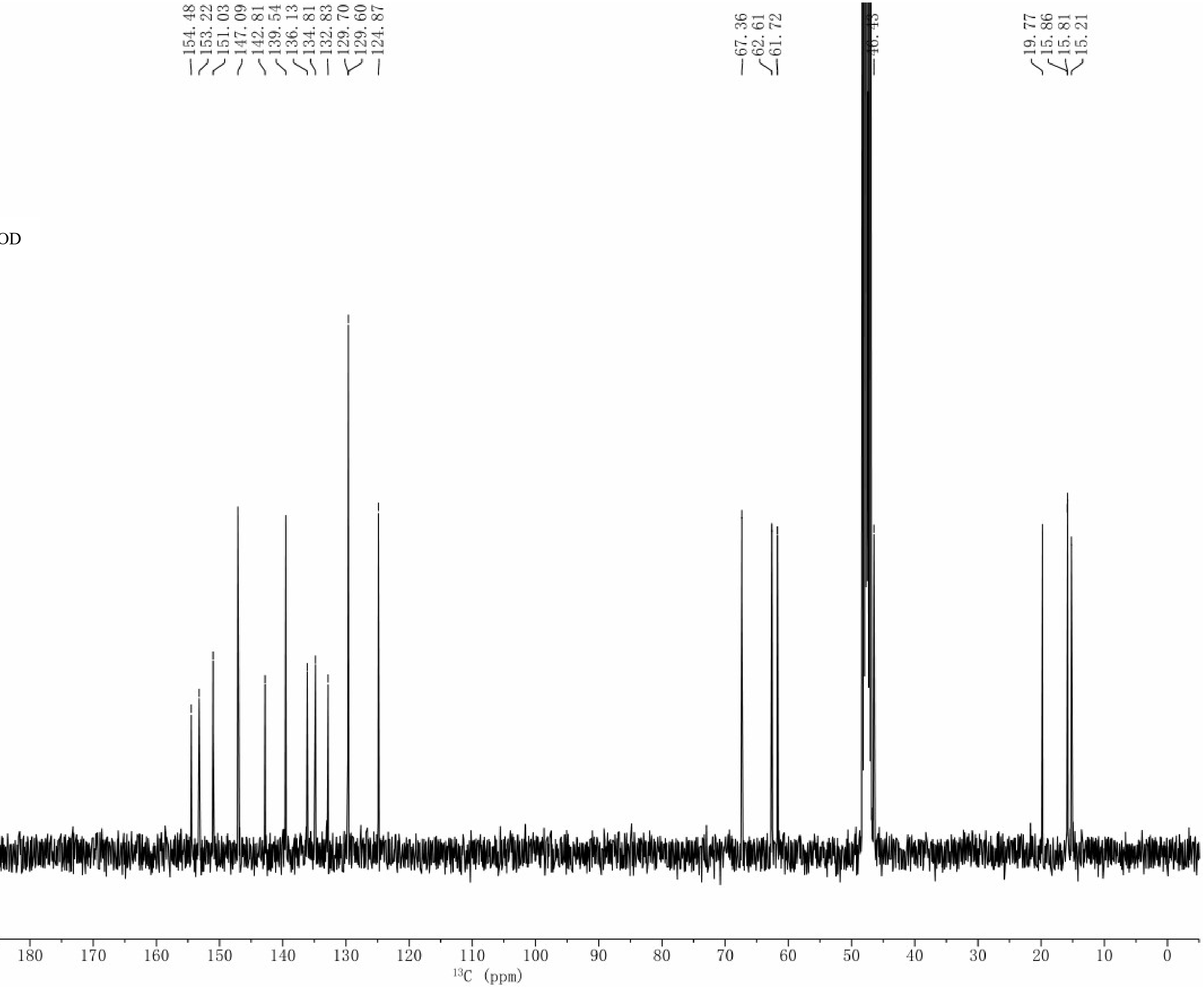

$^{13}C\{^1H\}$ NMR 101MHz d_4-CD_3OD
